# Characterizing genetic transmission networks among newly diagnosed HIV-1 infected individuals in eastern China: 2012–2016

**DOI:** 10.1371/journal.pone.0269973

**Published:** 2022-06-16

**Authors:** Xiaobei Ding, Antoine Chaillon, Xiaohong Pan, Jiafeng Zhang, Ping Zhong, Lin He, Wanjun Chen, Qin Fan, Jun Jiang, Mingyu Luo, Yan Xia, Zhihong Guo, Davey M. Smith

**Affiliations:** 1 Department of AIDS and STD Control and Prevention, Zhejiang Provincial Centers for Disease Control and Prevention, Hangzhou, China; 2 Department of Medicine, University of California, San Diego, California, United States of America; 3 Department of AIDS and STD Control and Prevention, Shanghai Municipal Centers for Disease Control and Prevention, Shanghai, China; University of Cincinnati College of Medicine, UNITED STATES

## Abstract

We aimed to elucidate the characteristics of HIV molecular epidemiology and identify transmission hubs in eastern China using genetic transmission network and lineage analyses. HIV-TRACE was used to infer putative relationships. Across the range of epidemiologically-plausible genetic distance (GD) thresholds (0.1–2.0%), a sensitivity analysis was performed to determine the optimal threshold, generating the maximum number of transmission clusters and providing reliable resolution without merging different small clusters into a single large cluster. Characteristics of genetically linked individuals were analyzed using logistic regression. Assortativity (shared characteristics) analysis was performed to infer shared attributes between putative partners. 1,993 persons living with HIV-1 were enrolled. The determined GD thresholds within subtypes CRF07_BC, CRF01_AE, and B were 0.5%, 1.2%, and 1.7%, respectively, and 826 of 1,993 (41.4%) sequences were linked with at least one other sequence, forming 188 transmission clusters of 2–80 sequences. Clustering rates for the main subtypes CRF01_AE, CRF07_BC, and B were 50.9% (523/1027), 34.2% (256/749), and 32.1% (25/78), respectively. Median cluster sizes of these subtypes were 2 (2–52, n = 523), 2 (2–80, n = 256), and 3 (2–6, n = 25), respectively. Subtypes in individuals diagnosed and residing in Hangzhou city (OR = 1.423, 95% CI: 1.168–1.734) and men who have sex with men (MSM) were more likely to cluster. Assortativity analysis revealed individuals were more likely to be genetically linked to individuals from the same age group (AI_age_ = 0.090, *P*<0.001) and the same area of residency in Zhejiang (AI_city_ = 0.078, *P*<0.001). Additionally, students living with HIV were more likely to be linked with students than show a random distribution (AI _student_ = 0.740, *P*<0.01). These results highlight the importance of Hangzhou City in the regional epidemic and show that MSM comprise the population rapidly transmitting HIV in Zhejiang Province. We also provide a molecular epidemiology framework for improving our understanding of HIV transmission dynamics in eastern China.

## Introduction

In China, 659,990 people were living with HIV as of November 2016, based on the China AIDS Response Progress Report [1]. Men who have sex with men (MSM) account for an increasing proportion of new national HIV/AIDS infections, from 2.5% in 2006 to 25.8% in 2014 [2]. The risk of acquiring HIV is 26-fold higher among gay men and other MSM [3]. Blued is a dating app for gay males, believed to be the largest dating app for MSM in mainland China. Using data from Blued app’s online and offline recruitment surveys, the MSM population size was estimated at 0.87–1.49% of the male population, with approximately 63.6% of the MSM registered in the Blued app from Zhejiang Province. Migrants are defined by the Chinese State Bureau of Statistics as individuals who are away from their registered residence for more than six of the previous 12 months. Here, migration was defined as movement between provinces within China. We defined inflow ratio as the number of immigrants to the province divided by the number of emigrants from the province and used this to predict the impact of mobility on the local MSM population. Zhejiang Province has one of the highest ratios of migrant inflow from other provinces in China [4], and the migration of MSM may increase HIV prevalence in migrant inflow regions. From 2004 to 2013, 4307 MSM HIV/AIDS cases were diagnosed in Zhejiang Province, of which the immigrants to Zhejiang province accounted for 46.9% [5]. However, the characteristics of HIV-1 transmission in these regions are currently poorly understood.

Identifying and monitoring HIV-1 transmission networks can improve our understanding of the origins and spread of the epidemic [6]. Recent advances in molecular epidemiology using HIV-1 *pol* sequences generated from routine drug resistance screening have greatly enhanced our ability to characterize the dynamics and structures of HIV transmission networks over space and time. Studies have identified risk factors for HIV transmission in China, including MSM, unprotected anal intercourse, multiple sexual partners, low rates of condom use, illegal drug use, migration, and a history of other sexually transmitted diseases (STDs) [7–10]. The use of geosocial networking apps might also increase sexual activity and high-risk sexual behavior among individuals living, working, or interacting in reasonably close proximity. Besides individual-level risk characteristics, few studies have evaluated shared characteristics between persons and used genetic linkage to explain assortativity and regional transmission in high-risk populations [11]. In China, network structure and neighborhood-level factors might be stronger predictors of HIV transmission than individual behaviors [12]. Assortativity refers to the pattern of mixing in the population with respect to a given characteristic [13]. Understanding assortativity patterns can help to better explain transmission networks, and may assist with identifying critical factors necessary for geographical allocation of interventions. For example, although individuals in shared clusters lived closer to each other than genetically unlinked individuals in Cologne-Bonn Region, Germany, they were less likely to be linked to individuals from the same zip code [14].

Previous studies, which focused on individual risk factors, identified the risk of HIV transmission based on phylogenetic analyses of viruses collected from individuals from Shanghai, Beijing, and Shenzhen [9, 15, 16]. The current study analyzed the shared characteristics between clusters of individuals and their linked partners. We investigated the molecular and epidemiological characteristics of HIV-1 strains in Zhejiang between 2012 and 2016 to infer local HIV transmission networks, identify high-risk areas in this region, and infer shared characteristics between linked individuals and their potential partners. Results from this study will help to further optimize targeted efforts necessary for HIV prevention in this region.

## Materials and methods

### Sample collection

Blood samples (5 mL) were collected by the staff of the local Centre for Disease Control and Prevention (CDC) for the routine measurement of CD4 cell counts prior to antiviral treatment. This study included subjects from HIV-infected individuals newly diagnosed between 2012 and 2016 in Zhejiang province whose blood samples remained more than 0.2ml after CD4 cell counts. All the eligible remaining blood samples were collected by the Center of Zhejiang HIV/AIDS confirmative laboratory. Epidemiological information was also collected by the staff of the local CDC. Most information, including age, occupation, transmission route, marital status, education background, residence at diagnosis, and current residence, had been reported via the Chinese HIV/AIDS Comprehensive Response Information Management System (CRIMS).

### HIV-1 *pol* gene sequence analysis

A fragment of HIV-1 *pol* gene (HXB2 position 2147–3462) was sequenced using DNA samples extracted from blood plasma. The sequences were assembled using Sequencher v5.0 (Gene Codes Corporation, Ann Arbor, MI, USA), aligned against all HIV-1 group M reference sequences available in the Los Alamos HIV Sequence Database (www.hiv.lanl.gov/content/sequence.html), and subsequently edited manually using BioEdit v7.2.0. The Subtype Classification tool COntext-based Modeling for Expeditious Typing (COMET) was used to subtype all sequences [17].

### Sensitivity analysis

HIV-TRACE (www.hiv-trace.org) has been used to infer transmission networks based on HIV subtype B [18]. However, more sensitive and specific methods are needed to identify recent transmission clusters and avoid spurious detection of subtype CRF01_AE and CRF07_BC clusters. Number of transmission clusters and cluster size are the key determinants of the sensitivity and specificity of molecular cluster inference. HIV transmission cluster amount and maximum cluster size with a range of genetic distance (GD) thresholds (0.1–2.0%) among main subtypes were calculated to determine the optimal GD threshold. Pairwise GDs were computed. HIV *pol* sequences tend to not diverge more than 0.01 substitutions/site from the baseline sequence in the first 10 years of infection [19], and the total sequence divergence tends to be less than 2.0%. Therefore, we explored the effect of using either conservative or liberal distance thresholds ranging between 0.1% and 2.0% [20]. We calculated the number of transmission clusters and the largest cluster sizes for subtypes CRF01_AE, CRF07_BC, and B.

HIV-TRACE was used to construct transmission clusters. HIV-1 *pol* sequences generated from each individual were used to infer the transmission network. All sequences were aligned to the *HXB2* (GenBank accession: K03455) reference sequence to correct for possible frameshifts and sequencing errors. A putative link between two individuals was considered whenever the distance between two sequences (Tamura-Nei 93 model) was below the GD threshold [21]. The evolutionary conservation in this region permitted pairwise alignment, and the pairwise Tamura-Nei 93 (TN 93) model is the most complex evolutionary model and can be computed rapidly via a closed form solution [19–21]. When calculating GDs between sequences, we resolved all IUPAC-defined nucleotide ambiguities (i.e. non-ATCG) to the corresponding nucleotide in the other sequences (i.e., Y is zero distance from both T and C). A phylogenetic test of conditional independence was used on each triangle in the network to remove spurious transitive connections. Moreover, 37 codons associated with major resistance in protease and reverse transcriptase were stripped from the alignment [19].

### Assortativity analysis

The assortativity Index (AI) was calculated from the district mixing matrix, a matrix comprised of the proportion of relationships between clustering individuals. The AI ranges from -1.0 to 1.0, with AI > 0 indicating that clustered individuals are more likely to be linked with individuals from the same category, AI < 0 indicating that clustered individuals are more likely to be linked with individuals from a different category, and 0 indicating that the relationship between clustered individuals is not influenced by category. We computed Newman’s assortativity index to describe the mixing patterns in our dataset using R package igraph [22, 23].

### Statistical analysis

Statistical analyses were performed using Statistical Product and Service Solutions (SPSS) v19.0 (IBM, Armonk, NY, USA). Non-parametric comparisons were assessed using Person’s χ^2^ tests or Fisher’s exact tests. All *P*-values < 0.05 were considered significant. Characteristics between clustered and non-clustered individuals in the transmission network were compared using logistic regression analysis. Odds ratios (ORs) and 95% confidence intervals were reported to show the direction and strength of associations. Student’s t-tests were performed to analyze means of independent groups.

## Ethical approval and informed consent

This study was part of mandated routine analysis of demographic surveillance data reported to the CRIMS. Individuals were assigned identification numbers unique to the study and could be linked back to the original data by authorized personnel only. The raw data did not contain any personally identifying information linked to particular individuals and was anonymized before its use. This study and its protocols were approved by the Ethical Review Committee of Chinese Center for Disease Control and Prevention (X140617334). The consent was waived by the ethics committee. All the procedures were carried out in accordance with approved guidelines and regulations.

## Results

### Study population

In total, 19,678 individuals were diagnosed with HIV-1 between 2012 and 2016 in Zhejiang province and 1,993 (10.1%) were enrolled in this study. All eleven prefectures in Zhejiang Province were represented in the study. The number of study subjects in each prefecture was as follows: Hangzhou (n = 804), Ningbo (n = 171), Wenzhou (n = 168), Huzhou (n = 68), Shaoxing (n = 73), Jinhua (n = 257), Quzhou (n = 34), Zhoushan (n = 25), Taizhou (n = 213), and Lishui (n = 43). In total, 88.5% (1764/1993) of the participants were male, compared to 82.6% (16256/19678) of all newly diagnosed patients (*X*^*2*^ = 53.730, *P*<0.01). 55.5% (1106/1993) were unmarried, compared to 41.5% (8159/19678) of all newly diagnosed patients (*X*^*2*^ = 185.982, *P*<0.01). The main age groups: 18–25 years and 26–35 years, accounted for 31.8% (633/1993) and 30.8% (613/1993) of study participants, compared to 22.0% (4322/19678) and 28.5% (5608/19678) of the diagnosed individuals, respectively. More than half of the population had senior high school or above educational background (n = 1,026, 51.5%; Table 1). Homosexual contact was the most frequently reported risk factor (n = 1,173, 58.9%), followed by heterosexual contact (n = 796, 39.9%). Most individuals (n = 1,701, 85.3%) lived in Zhejiang province at the time of diagnosis. 48.5% (569/1173) of MSM were diagnosed in Hangzhou city, which was higher than the proportion of non-MSM (235/820; *X*^*2*^ = 76.730, *P*<0.01) diagnosed in the same city. Further, 92.4% (207/224) of students were MSM, a higher percentage than that of other populations (*X*^*2*^ = 117.338, *P*<0.001). The majority of subjects were unmarried (55.5%, 1106/1993), while 11 pairs of infected individuals in this study were spouses.

**Table 1:** Population characteristics between clustering and non-clustering individuals.

	study population, N(%)	Clustering, N(%)			Non-Clustering, N(%),	Odds ratio (95%CI)	*P*
		Clustering, N(%)	clustering in a dyad, N(%)	larger than a dyad, N(%)			
Year of diagnosed							
2012	339(17.0)	134(16.2)	33(15.1)	101(16.6)	205(17.6)	1.00	
2013	583(29.3)	285(34.5)	65(29.8)	220(36.2)	298(25.5)	1.38(1.04–1.84)	0.026
2014	374(18.8)	160(19.4)	43(19.7)	117(19.2)	214(18.3)	1.01(0.73–1.38)	0.972
2015	518(26.0)	192(23.2)	58(26.6)	134(22)	326(27.9)	0.81(0.60–1.09)	0.175
2016	179(9.0)	55(6.7)	19(8.7)	36(5.9)	124(10.6)	0.55(0.37–0.83)	0.004
Place where diagnosed and resident							
Hangzhou-Hangzhou	610(30.6)	290(35.1)	66(30.3)	224(36.8)	320(27.4)	1.42(1.17–1.73)	<0.001
Hangzhou-Other	194(9.7)	75(9.1)	19(8.7)	56(9.2)	119(10.2)	0.99(0.72–1.35)	0.949
Other-Hangzhou	22(1.1)	7(0.8)	4(1.8)	3(0.5)	15(1.3)	0.73(0.29–1.81)	0.501
Other-Other	1167(58.6)	454(55.0)	129(59.2)	325(53.5)	713(61.1)	1.00	
Age when diagnosed							
<18 years old	34(1.7)	16(1.9)	5(2.3)	11(1.8)	18(1.5)	1.74(0.86–3.53)	0.125
18–25	633(31.8)	286(34.6)	79(36.2)	207(34.0)	347(29.7)	1.61(1.23–2.11)	0.000
26–35	613(30.8)	271(32.8)	60(27.5)	211(34.7)	342(29.3)	1.55(1.18–2.03)	0.001
36–45	355(17.8)	132(16.0)	40(18.3)	92(15.1)	223(19.1)	1.16(0.85–1.57)	0.345
46-	358(18.0)	121(14.7)	34(15.6)	87(14.3)	237(20.3)	1.00	
Educational background							
Junior high school and below	967(48.5)	379(45.9)	106(48.6)	273(44.9)	588(50.4)	1.00	
Senior high school	438(22.0)	191(23.1)	44(20.2)	147(24.2)	247(21.2)	1.20(0.95–1.51)	0.119
College or above	588(29.5)	256(31.0)	68(31.2)	188(30.9)	332(28.4)	1.20(0.97–1.47)	0.091
Marital Status							
Married	543(27.2)	210(25.4)	57(26.1)	153(25.2)	333(28.5)	1.00	
Unmarried	1106(55.5)	505(61.1)	131(60.1)	374(61.5)	601(51.5)	1.33(1.08–1.64)	0.007
Divorced/widowed	344(17.3)	111(13.4)	30(13.8)	81(13.3)	233(20.0)	0.75(0.57–1.00)	0.053
ethnicity							
Han	1899(95.3)	791(95.8)	210(96.3)	581(95.6)	1108(94.9)	1.00	
Minority group	94(4.7)	35(4.2)	8(3.7)	27(4.4)	59(5.1)	0.83(0.54–1.27)	0.396
STI before diagnosis							
No	1335(67.0)	547(66.2)	157(72)	390(64.1)	788(67.5)	1.00	
Yes	433(21.7)	196(23.7)	40(18.3)	156(25.7)	237(20.3)	1.19(0.96–1.48)	0.116
NA	225(11.3)	83(10.0)	21(9.6)	62(10.2)	142(12.2)	0.84(0.63–1.13)	0.248
Subtype							
B	78(3.9)	25(3.0)	6(2.8)	19(3.1)	53(4.5)	1.00	
CRF01_AE	1027(51.5)	523(63.3)	116(53.2)	407(66.9)	504(43.2)	2.20(1.35–3.56)	0.002
CRF07_BC	749(37.6)	256(31.0)	88(40.4)	168(27.6)	493(42.2)	1.10(0.67–1.81)	0.706
CRF08_BC	110(5.5)	16(1.9)	8(3.7)	8(1.3)	94(8.1)	0.36(0.18–0.74)	0.005
Others	29(1.5)	6(0.8)	0(0)	6(1.0)	23(2.0)	0.55(0.20–1.53)	0.253
Transmission route							
HTS	796(39.9)	259(31.4)	83(38.1)	176(28.9)	537(46.0)	1.00	
MSM	1173(58.9)	566(68.5)	134(61.5)	432(71.1)	607(52.0)	1.59(1.30–1.94)	0.000
Other risk	24(1.2)	1(0.1)	1(0.5)	0(0)	23(2.0)	0.09(0.01–0.64)	0.017
Gender							
Female	229(11.5)	60(7.3)	25(11.5)	35(5.8)	169(14.5)	1.00	
Male	1764(88.5)	766(92.7)	193(88.5)	573(94.2)	998(85.5)	2.16(1.59–2.94)	<0.001
HIV infection status							
HIV	1481(74.3)	627(75.9)	169(77.5)	458(75.3)	854(73.2)	1.00	
AIDS	512(25.7)	199(24.1)	49(22.5)	150(24.7)	313(26.8)	0.77(0.61–0.96)	0.021

MSM, men who have sex with men; HTS, heterosexual. NA, not available. Clustering vs. non-clustering; non parametric

### Transmission network analysis

**Fig 1:**
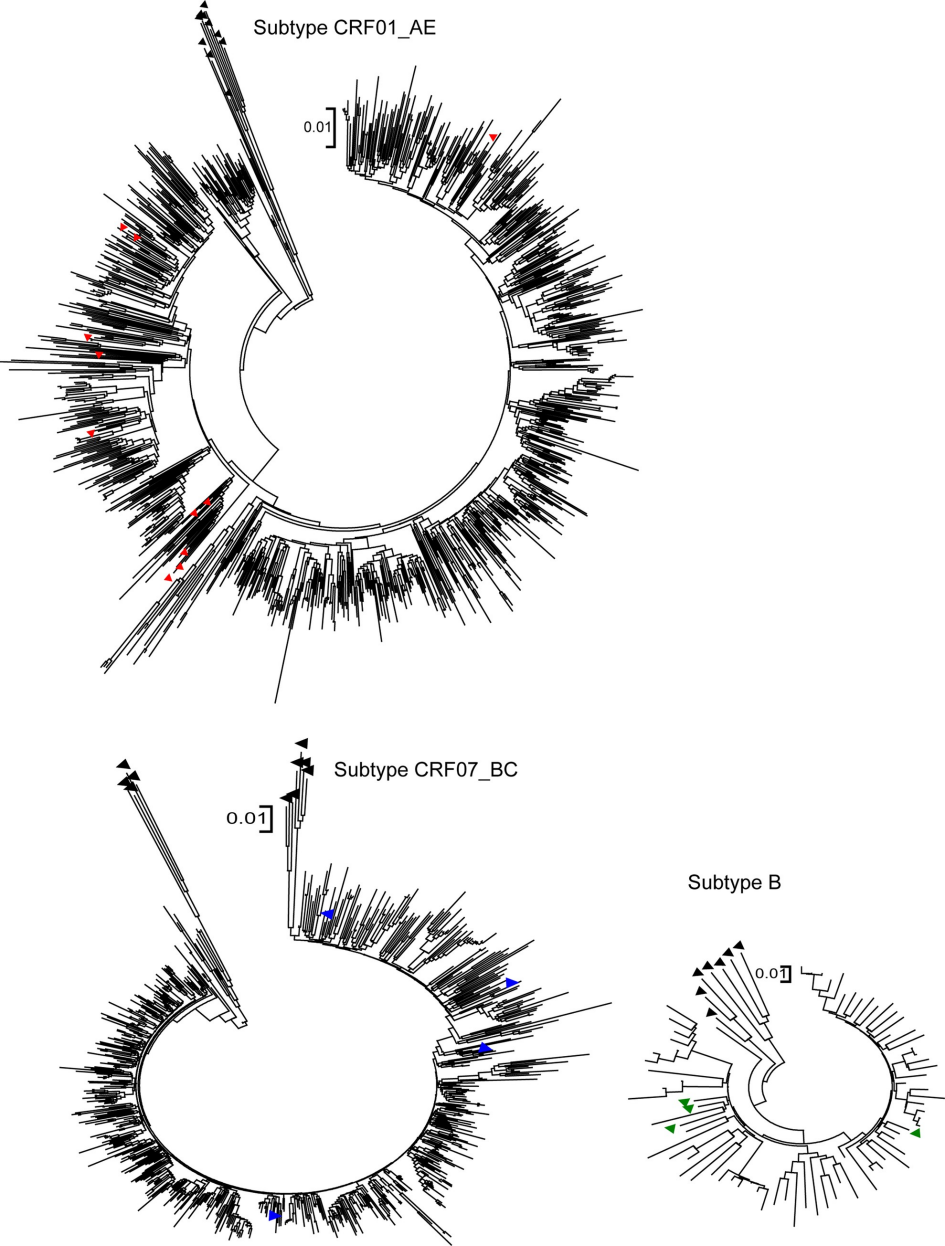
Phylogenetic trees for CRF01_AE, CRF07_BC and B. The phylogenetic tree is computed by Mega v 5.05. Neighbor-joining phylogenetic tree constructed based on partial pol genes from individuals who were newly diagnosed between 2012–2016 in Zhejiang. The reference sequences obtained from the Los Alamos HIV database are marked as solid triangle. The CRF01_AE, CRF07_BC, and B reference sequences are labeled with red, blue and green solid upward-pointing triangles (▲). The other reported reference sequences are labeled with a black solid upward-pointing triangle (▲).

The 1,993 sequences were classified into 10 subtypes, with the main subtypes being CRF01_AE (51.5%, 1025/1993) and CRF07_BC (37.6%, 749/1993), followed by CRF08_BC (5.5%, 110/1993), B (3.9%, 78/1993), C (0.9%, 17/1993), CRF03_AB (0.3%, 6/1993), CRF02_AG (0.1%, 2/1993), CRF04_CPX (0.1%, 2/1993), A (0.05%, 1/1993), and CRF18_CPX (0.05%, 1/1993) (See Fig 1 for phylogenetic trees). The mean GDs within CRF01_AE and CRF07_BC in the population were 0.047±0.012 and 0.028±0.010 substitutions per site, respectively.

**Fig 2:**
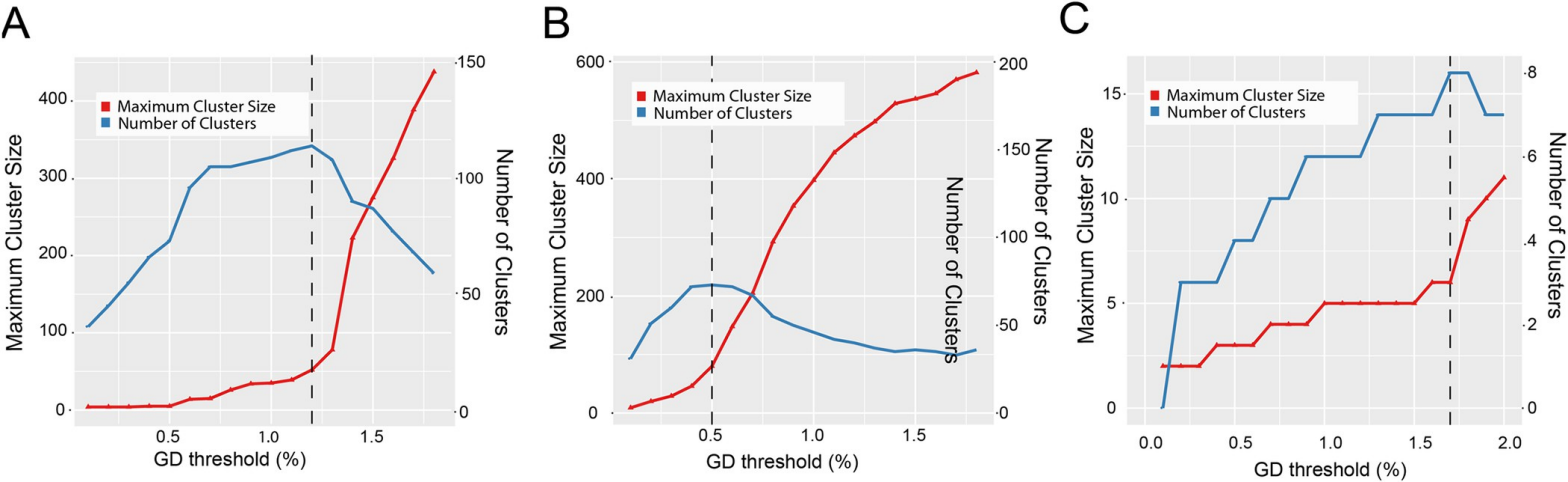
Genetic distance thresholds. (A–C) HIV transmission cluster amount and maximum cluster size with a range of GDs (0.1–2.0%) among subtypes CRF01_AE, CRF07_BC, and B. The red line depicts the maximum cluster size with GDs varying from 0.1% to 2.0%, whereas the blue line represents the number of clusters. The epidemiologically plausible range of thresholds for each subtype is highlighted with dashes.

The curves of HIV transmission cluster amounts and maximum cluster sizes with a range of GDs (0.1–2.0%) among subtypes CRF01_AE, CRF07_BC, and B were plotted (Fig 2). Among 78 subtype B sequences, a maximum of eight clusters were identified with cluster sizes ranging from two (n = 3 dyads) to six sequences, and the plausible GD threshold was 1.7% (Fig 2C). Among subtype CRF01_AE sequences, 114 clusters were identified with cluster sizes ranging from 2 (n = 58 dyads) to 52 sequences and an optimal GD threshold of 1.2% (Fig 2A). In the CRF07_BC network, a maximum 66 clusters were identified, with cluster sizes ranging from 2 (n = 44 dyads) to 80 sequences, yielding an optimal GD threshold of 0.5% (Fig 2B).

**Fig 3:**
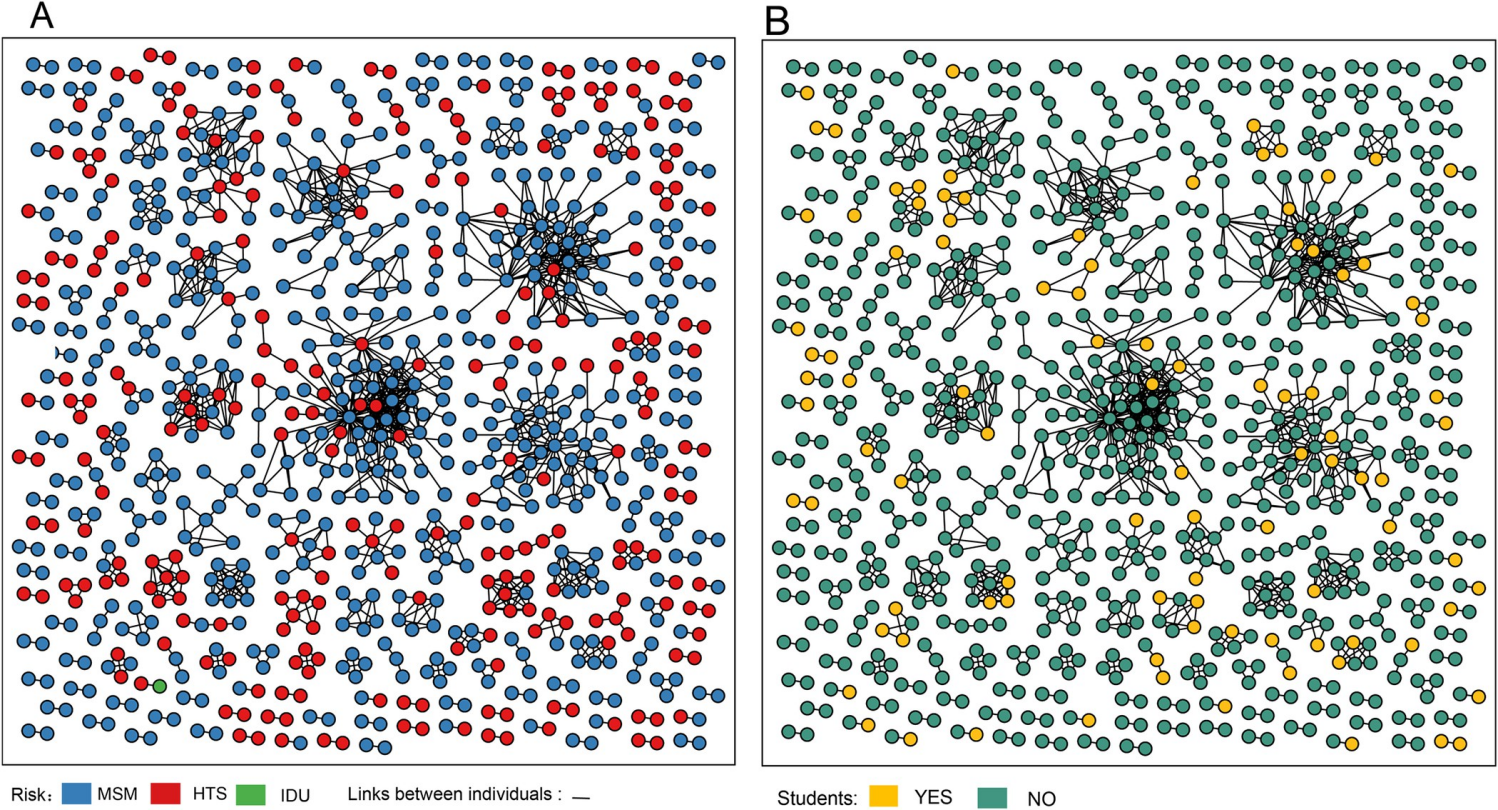
HIV transmission network. HIV transmission clustering in Zhejiang by risk group (A) and student status (B). Edges indicate genetic linkage (< optimal GD threshold).

The clustering rates for CRF01_AE, CRF07_BC, B, CRF08_BC, and C were 50.9% (523/1027), 34.2% (256/749), 14.5% (16/110), 32.1% (25/78), and 35.3%(6/17), respectively. The median cluster sizes of these subtypes were 2 (2–52, n = 523), 2 (2–80, n = 256), 3 (2–6, n = 25), 2 (2–6, n = 16), and 6 (n = 6), respectively. Using the selected GD threshold, 826 individuals were linked with at least one other individual, giving a total of 1,305 edges (Fig 3A).

Eleven pairs of spouses were identified among the clusters. All appeared in the same cluster with their spouses, and 63.6% (14/22) of them formed dyads. Individuals over 50 years of age made up 68.2% (15/22) of the spousal pairs. The infection routes for 11 women was heterosexual transmission. Nine of eleven male cases were infected via heterosexual behavior, one was infected via sexual encounter with other men, and the last was infected via injection drug use.

### Large size clusters

The largest cluster sizes in subtypes CRF07_BC, CRF01_AE, and B contained 80, 52, and 6 individuals, respectively. In these clusters, the proportions of gay men were 80% (64/80), 82.6% (43/52), and 100% (6/6), respectively, which were higher than in other clusters; 44.9% (62/138) of the individuals in these clusters were diagnosed in Hangzhou city. No significant differences in the region of diagnosis, marital status, STI history, HIV infection status, education background, or place of residence were observed among the clusters.

Three female cases were included in the large cluster infected with CRF07_BC, and all of them were infected via heterosexual transmission. All of their spouses were HIV-1-positive. However, no sequences were obtained from their spouses.

## Characteristics of genetically linked individuals

Population characteristics were compared between clustered and non-clustered individuals (Table 1). In univariate analysis, the following characteristics were significantly associated with clustering: individuals between 18 and 35 years at the time of HIV diagnosis, unmarried, infected with subtype CRF01_AE, MSM, male, diagnosed and residing in Hangzhou city, and prior HIV testing history (*P*<0.01). In multivariate analysis, the following characteristics were significantly associated with clustering: MSM and infected with subtype CRF01_AE (*P*<0.01).

### Assortativity analysis between genetically linked individuals

**Fig 4:**
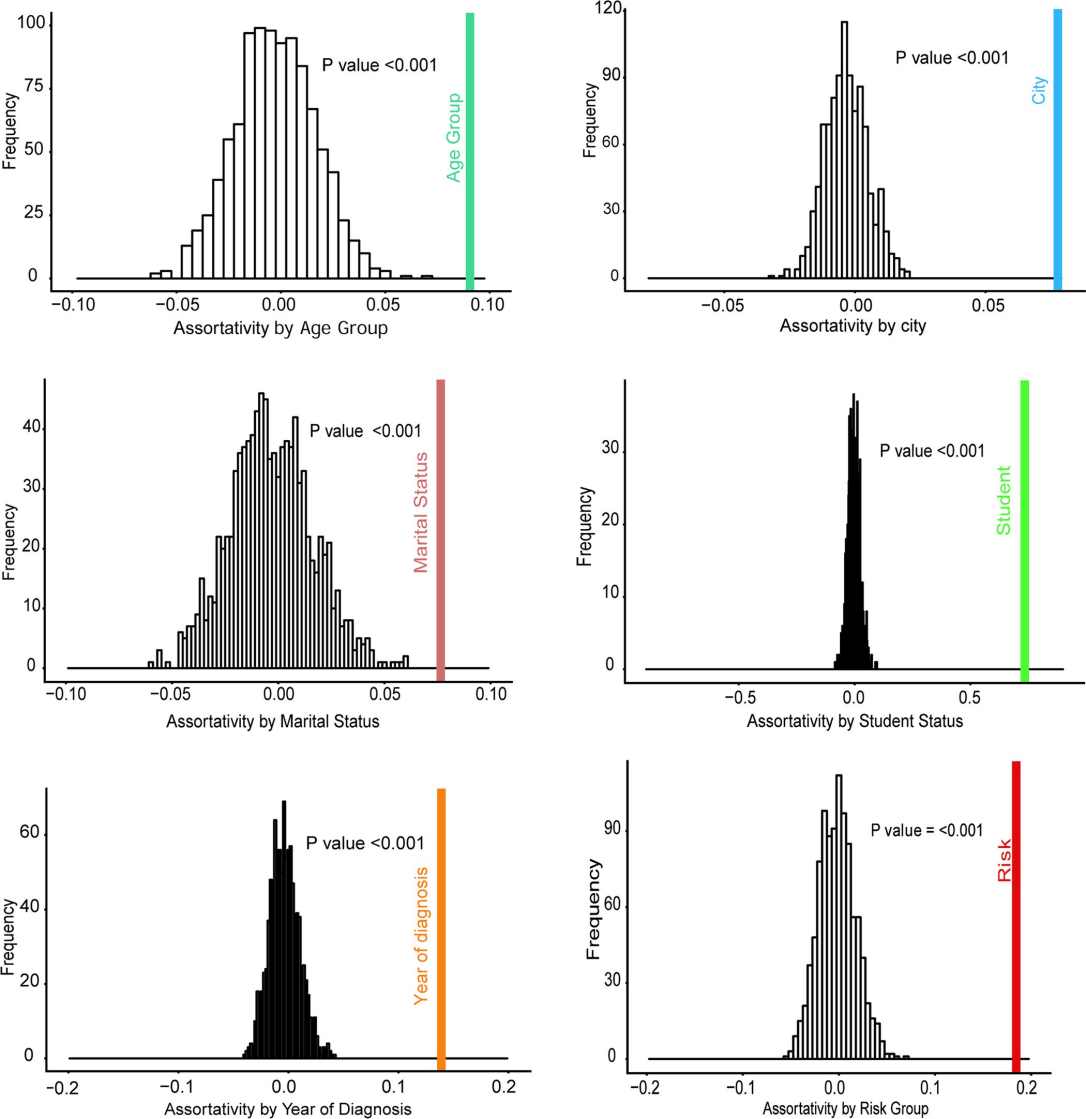
Assortativity analysis among linked individuals. The random value is 1000 times, and “1” indicates linked individuals originating from the same category, whereas “-1” indicated those from different category. Assortativity analysis by age group, student status, city of residence, marital status, and year of diagnosis, respectively. Assortativity index for above were AI_age_ = 0.090, AI_city_ = 0.078, AI_marital_ = 0.076, AI_student_ = 0.740, AI_year_ = 0.140, and AI_risk_ = 0.185.

To evaluate epidemic characteristics between genetically linked individuals (putative transmission pairs), we computed assortativity index based on individual demographics. Area of current residence, student status, risk group, age group, marital status, gender, STI and year of diagnosis between linked individuals was assortative (Fig 4). Clustering between individuals was not assortative by gender (AI_gender_ = 0.020, *P* = 0.199). Individuals were more likely to be genetically linked to individuals of the same age group (AI_age_ = 0.090, *P*<0.001), from the same area of residence in Zhejiang (AI_city_ = 0.078, *P*<0.001), diagnosed in the same year (AI_year_ = 0.140, *P*<0.001), have the same risk group(AI_risk_ = 0.185, *P*<0.001), and with the same marital status (AI_marital_ = 0.076, *P*<0.001). Students were more likely to be genetically linked with students (AI_student_ = 0.740, *P*<0.001). Clustering between individuals was not assortative by STI (AI_sti_ = -0.008, *P =* 0.589).

## Discussion

We analyzed the transmission networks of HIV-1 infections among 1,993 individuals newly diagnosed in Zhejiang province between 2012 and 2016 and determined plausible GDs to identify potential transmission partners. Based on our results, we arrived at three conclusions regarding the dynamics of HIV-1 epidemics. First, the GD within CRF07_BC was significantly lower than that within CRF01_AE and B in Zhejiang. Second, Hangzhou city, the capital of Zhejiang, was a hub for the HIV-1 transmission network in Zhejiang and was therefore the most important region for targeted intervention. MSM and Hangzhou city were risk factors for clustering. Third, students were more likely to link with other students than exhibit a random distribution.

Using sensitivity analysis, we determined that the GD threshold of the CRF07_BC subtype was much lower than that of other subtypes in Zhejiang. This result can be attributed to the dense sampling in the population and the faster rate of spread. CRF01_AE and CRF07_BC are major subtypes in Asia, and most recent studies have used phylogenetic analysis to investigate HIV transmission clusters in China [24, 25]. However, phylogenetics is insufficient for epidemiology analysis because it cannot identify recent infection events, and individuals in the cluster are treated as equally connected. Additionally, high support for the clade does not indicate that members of the clade are necessarily closely related to each other [26]. Here, we applied the GD threshold to identify transmission clusters and recent infection events. This method is thought to be sufficient for epidemiological purposes [27]. Lower distance thresholds (e.g. 0.5%) might be more appropriate for distinguishing rapidly growing clusters or populations where more rapid evolution (non-B subtypes) predominates [28]. This low GD may represent highly related and rapidly expanding transmission networks in Zhejiang, and such information could be important for public health control efforts. A GD threshold of 0.5% for CRF07_BC could serve as a useful proxy for epidemiological relatedness in a surveillance setting in eastern China. Subtype B is the main circulating subtype in the US and Western Europe, and the GD threshold of *pol* sequences ranges from 1.0% to 2.0% [28–30]. Although the proportion of the B subtype in China is low, we found that it showed a plausible GD with an acceptable cluster size. Thus, we recommend using a genetic threshold of 1.7% for the B subtype in China to identify clusters to balance sensitivity and specificity.

Hangzhou city is located in southern Zhejiang province, which is the provincial center for economy, culture, education, and tourism. This city accounts for 48.3% of newly diagnosed HIV-1 infections among MSM in Zhejiang province. Up to 73.1% of edges connected one node from Hangzhou to nodes from other regions in Zhejiang Province among MSM infections, inferring potential transmission between Hangzhou and other regions and suggesting that Hangzhou plays an important role in the HIV epidemic in Zhejiang province [10]. The workbook method estimated that 69.6% of MSM living with HIV/AIDS in Zhejiang resided in urban areas [31]. The urban areas contained more convenient sexual venues and more inflow populations, and were more attractive to MSM [32]. Thus, as a metropolitan area, Hangzhou city represents the dominant proportion, based on the risk of clustering in transmission network (Table 1) and possession of the largest cluster. Current laws and regulations, as well as social stigma, force MSM to hide their sexual orientation and behaviors in both their origin and destination residences [4]. Other studies have shown that populations of Chinese MSM are likely to hide their sexual identity and engage in sexual behaviors in areas other than their hometowns to avoid social stigma [33]. Thus, these findings are crucial for developing targeted interventions for MSM in Hangzhou and are expected to affect the entire province.

We found that city of residence, student status, risk group, age group, marital status, and year of diagnosis between linked individuals was assortative. The AI value for student status was very high, suggesting direct or indirect transmission between students. The questionnaire survey of college students in Qingdao showed that 42.7% of students liked to choose college students as sexual partners, and 86.0% of students looked for sexual partners through the internet [34]. In China, there was a certain spatial and temporal clustering among young MSM students aged 18 to 24 who were reported HIV infection [35]. Targeted prevention and control measures should be carried out in these hot spots and clustering areas to reduce the prevalence of HIV among students. In this study, 92.4% of students were young MSM. Young MSM were more likely to find sexual partners in bars and on the internet, whereas middle-aged men preferred public baths and parks. These differences may be related to the participants’ cultural and recreational environments [36]. MSM within the same age group and who engaged in non-internet-based intercourse easily formed closer relationships with strong ties [37], suggesting that different categories of MSM might have relatively separated networks. Alternatively, these differences could be driven by a lack of accessible same-age partners in the social settings in which they are primarily found [38]. China’s most popular gay app has grown to include 15 million users in only 2 years [39]. Among social app users, 36.4% met their last partner within 24 hours of the first message exchange [39]. Thus, we can infer that many MSM students find their sexual partners within short times and in close proximity. School sites acted as centers of sexual activity, and some students had sexual encounters with school classmates. In a cross-sectional study determining sexual behavior among 535 college students in Zhejiang Province, 88.9% of MSM students did not insist on using condoms in the previous year [40]. Education regarding potential health issues associated with unprotected sex might influence condom use and prevent the spread of diseases; therefore, it is necessary to strengthen public knowledge of HIV infection among MSM students in Zhejiang province. Moreover, social network platforms can be used by local AIDS publicity campaigns to ensure dissemination of effective health education.

Our results showed that CRF01_AE and CRF07_BC were the two major HIV subtypes among newly HIV-infected MSM between 2012 and 2016 in Zhejiang province. CRF01_AE was the main genotype, accounting for 56.0% of cases This is consistent with previous reports among MSM populations in Zhejiang province in 2009 (86.8%, 33/38) and 2011 (62.4%, 63/101) [41, 42], indicating a downward temporal trend. However, the proportion of CRF07_BC subtype among MSM in Zhejiang province accounted for 7.9% (3/38) of MSM in 2009, 31.7% (32/101) in 2011, and 42.0% in this study, indicating an upward temporal trend. These findings are consistent with a previous report which showed an increase in CRF07_BC among 18–25-year-old individuals in Zhejiang province from 2009 to 2013 and was also consistent with the trend of subtype diversification among populations of MSM in China [43]. CRF01_AE (53.8%) and CRF07_BC (36.5%) were the predominant genotypes among young MSM in 2012–2013 in Shanghai city (borders with Zhejiang) [18], similar to that in Zhejiang province. Moreover, another investigation among populations of MSM in Shenzhen city in 2012 showed that CRF01_AE and CRF07_BC accounted for 32.3% and 43.2% of cases, respectively [10], in contrast to the results observed in Zhejiang province. Therefore, infection transmission by MSM may be subject to geographical constraints.

Univariate comparison between clustering and non-clustering nodes revealed that linked individuals were significantly more likely to be between 18 and 35 years of age, unmarried, infected with subtype CRF01_AE, MSM, male, diagnosed and residing in Hangzhou city, and had prior HIV testing history. This multivariable model revealed that clustering individuals were more commonly MSM and infected with subtype CRF01_AE. The risk factors: unmarried, 18–35 years old, male, diagnosed and residing in Hangzhou city, and prior HIV testing history did not remain significant in the multivariable model. The reason may be that the independent variable MSM is associated with being male, unmarried, and 18–35 years old. Multivariable model can correct the influence of various confounding factors, and the results are often more reliable.

This study has some limitations. First, this study was subjected to sampling bias. Individuals were enrolled by convenience sampling without random selection. However, these data included 1,993 sequences reflecting HIV infection in Zhejiang. Additionally, some data, including drug use and STI history, were self-reported, and incorrect reporting of this information might have introduced bias into our model. The individuals in this study were newly diagnosed between 2012 and 2016, and it was not possible to make a more complete retrospective survey for individuals within transmission clusters. Moreover, these data could not be used to verify the direct or indirect transmission relationships among individuals within transmission clusters. However, the samples obtained from the newly reported MSM students in Zhejiang province in this study accounted for the majority of cases; therefore, the analysis results were thought to have good representation.

In conclusion, this study determined plausible GD thresholds for subtypes CRF07_BC, CRF01_AE, and B for genetic transmission network; these thresholds could serve as references in eastern China. Furthermore, we established a preliminary understanding of transmission in Zhejiang province, and also identified the characteristic of the clustering individuals, highlighting the role of Hangzhou in the transmission network. We found that transmission links were more likely between individuals from the same area of residence. Our findings highlight the importance of transmission network analysis to get a better understanding of regional transmission patterns. Further studies of molecular transmission networks should focus on the individual transmission relationships and individuals with high degrees of edges in the transmission network. Combined with epidemiological information, we can determine the high-risk factors for the population in the transmission network and further guide intervention measures against HIV transmission.

## Supporting information

S1 File Sequences in this study and reference sequences used in phylogenetic analyses. 14 reference sequences(1–14 sequences) and 717 sequences of CRF 07_BC in this study were listed together.
(DOC)
