# Supplementary material for: Characterizing genetic transmission networks among newly diagnosed HIV-1 infected individuals in eastern China: 2012–2016
Source: PLoS One. 2022 Jun 16;17(6):e0269973. doi: 10.1371/journal.pone.0269973 (PMC9202869; doi:10.1371/journal.pone.0269973)
Supplement: S1 File — 14 reference sequences(1–14 sequences) and 717 sequences of CRF 07_BC in this study were listed together. (DOC) [file pone.0269973.s001.doc]

>B.TH.90.BK132

CCTCAAATCACTCTTTGGCAACGACCCCTCGTCACAATAAAGATAGGGGGGCAACTAAAG

GAAGCTCTATTAGATACAGGGGCAGATGATACAGTATTAGAAGAAATGAATTTGCCAGGA

AGATGGAAACCAAAAATGATAGGGGGAATTGGAGGTTTTATCAAAGTAAGACAATATGAT

CAGATACTTGTAGAAATCTGTGGACATAAAGCTATAGGTACAGTATTAATAGGACCTACA

CCTGTCAACATAATTGGAAGAAATTTGTTGACTCAGCTTGGTTGCACTTTAAATTTTCCT

ATTAGTCCTATTGAAACTGTACCAGTAAAATTAAAGCCAGGAATGGATGGCCCAAAAGTT

AAACAATGGCCATTGACAGAAGAAAAAATAAAAGCATTAGTAGAAATTTGTACAGAAATG

GAAAAGGAAGGAAAAATTTCAAAAATTGGGCCTGAAAATCCATACAATACTCCAGTATTT

GCCATAAAGAAAAAAGACAGTACTAAATGGAGAAAATTAGTAGATTTCAGAGAACTTAAT

AAAAGAACTCAAGACTTCTGGGAAGTTCAATTAGGAATACCACATCCCGCAGGGTTGAAA

AAGAAAAAATCAGTAACGGTACTGGATGTGGGTGATGCATATTTTTCAGTTCCCTTAGAT

AAAGACTTCAGGAAATATACTGCATTTACCATACCGAGTACAAACAATGAGACACCAGGG

ATTAGATATCAGTACAATGTGCTTCCACAGGGATGGAAAGGATCACCAGCAATATTCCAA

TGTAGCATGACAAAAATCTTAGAGCCTTTTAGAAAACAAAATCCAGACATAGTTATCTAT

CAATACATGGATGATTTGTATGTAGGATCTGACTTAGAAATAGGGCAGCATAGAACAAAA

ATAGAGGAACTGAGACAACATCTGTTGAGGTGGGGATTTACCACACCAGATAAAAAACAT

CAGAAAGAACCTCCATTCCTTTGGATGGGTTATGAACTCCATCCTGATAAATGGACAGTA

CAACCTATAGTGCTGCCGGAAAAAGACAGCTGGACTGTCAATGACATACAGAAGTTAGTG

GGAAAATTGAATTGGGCAAGTCAGATTTACCCAGGGATTAAAGTAAAGCAGTTATGTAAA

CTCCTTAGGGGAACCAAAGCACTAACAGAAGTAGTACCACTAACAAAAGAGGCAGAGCTA

GAACTGGCAGAAAACAGGGAAATTCTAAAAGAAACAGTACATGGAGTG

>01-AE.CM240

CCTCAAATCACTCTTTGGCAACGACCCCTTGTCACAGTAAAAATAGGAGGACAGCTGAAA

GAAGCTCTATTAGATACAGGAGCAGATGATACAGTATTAGAAGATATAAATTTGCCAGGA

AAATGGAAACCAAAAATGATAGGGGGAATTGGAGGTTTTATCAAGGTAAAGCAATATGAT

CAGATACTTATAGAAATCTGTGGAAAAAAGGCTATAGGTACAGTATTAGTAGGACCTACA

CCTGTCAACATAATTGGACGAAATATGTTGACTCAGATTGGTTGTACTTTAAATTTCCCA

ATTAGTCCTATTGACACTGTACCAGTAACATTAAAGCCAGGAATGGATGGACCAAAGGTT

AAACAGTGGCCATTGACAGAAGAAAAAATAAAAGCATTAACAGAAATTTGTAAAGAGATG

GAAGAGGAAGGAAAAATCTCAAAAATTGGGCCTGAAAATCCATACAATACTCCAGTATTT

GCTATAAAGAAAAAGGACAGCACCAAATGGAGGAAATTAGTAGATTTCAGAGAGCTCAAT

AAAAGAACTCAGGACTTTTGGGAAGTTCAATTAGGAATACCGCATCCAGCAGGTTTAAAA

AAGAAAAAATCAGTAACAGTACTAGATGTGGGAGATGCATATTTTTCAGTTCCTTTAGAT

GAAAGCTTTAGAAAGTATACTGCATTCACCATACCTAGTATAAACAATGAGACACCAGGA

ATCAGATATCAGTACAATGTGCTGCCACAGGGATGGAAAGGATCACCGGCAATATTCCAG

AGTAGCATGACAAAAATCTTAGAGCCCTTTAGAATAAAAAATCCAGAAATGGTTATCTAT

CAATACAAGGATGACTTGTATGTAGGATCTGATTTAGAAATAGGGCAGCACAGAACAAAA

ATAGAGGAGCTAAGAGCTCATCTATTGAGCTGGGGATTTACTACACCAGACAAAAAGCAT

CAGAAGGAACCTCCATTCCTTTGGATGGGATATGAACTCCATCCTGACAGATGGACAGTC

CAGCCTATAGAACTGCCAGAAAAAGACAGCTGGACTGTCAATGATATACAGAAATTAGTG

GGAAAACTAAATTGGGCAAGTCAAATTTATGCAGGGATTAAGGTAAAGCAACTGTGTAAA

CTCCTCAGGGGAGCTAAAGCACTAACAGACATAGTACCACTGACTGAAGAAGCAGAATTA

GAGTTGGCAGAGAACAGGGAGATTCTAAAAACCCCTGTGCATGGAGTA

>02-AG.NG

CCTCAAATCACTCTTTGGCAACGACCCTTAGTTACAGTAAGAATAGAGGGACAGCTAATA

GAAGCCCTATTAGACACAGGAGCAGATGATACAGTATTAGAAGACATAAATTTACCAGGA

AAATGGAAACCAAAAATGATAGGGGGAATTGGAGGTTTTATCAAAGTAAGACAATATGAT

CAAATACTTATAGAAATTTGTGGAAAAAAGGCCATAGGTACAGTATTAGTAGGACCTACA

CCTGTCAACATAATTGGACGAAATATGTTGACTCAGATTGGTTGTACTTTAAATTTTCCA

ATTAGTCCTATTGAAACTGTGCCAGTAAAATTAAAGCCAGGAATGGATGGCCCAAAGGTT

AAACAATGGCCATTGACAGAAGAAAAAATAAAAGCATTAACAGACATTTGTACAGAGATG

GAAAAGGAAGGAAAAATTTCAAAAATTGGACCTGAAAATCCATACAATACTCCAGTATTT

GCCATAAAGAAAAAGGATAGTACTAAATGGAGAAAATTAGTAGATTTCAGAGAACTCAAT

AAGAGAACTCAAGATTTCTGGGAGGTCCAGTTAGGAATACCTCATCCCGCGGGATTAAAA

AAGAAAAAATCAGTAACAGTGCTAGATGTGGGGGATGCATATTTTTCAGTTCCCTTAGAT

AAAGACTTTAGAAAGTATACTGCATTTACTATACCTAGTGTAAATAATGAGACACCAGGG

ATTAGATACCAGTACAATGTGCTTCCACAGGGATGGAAAGGGTCACCGGCAATATTTCAG

GCAAGCATGACAAAAATCTTAGAGCCCTTTAGAACAAAAAATCCAGAGATAGTGATCTAC

CAATACATGGATGATTTATATGTAGGATCTGATTTAGAAATAGGGCAGCATAGAGCAAAA

ATAGAGGAGTTGAGAGGACATCTACTGAAATGGGGATTTACCACACCAGACAAAAAGCAT

CAGAAAGAACCTCCATTTCTTTGGATGGGTTATGAACTCCATCCTGATAAATGGACAGTC

CAGCCTGTAGAACTACCAGAAAAAGACAGCTGGACTGTCAATGATATACAGAAATTAGTG

GGAAAACTAAATTGGGCAAGTCAGATTTATGCAGGAATTAAAATAAAGCAACTGTGTAGA

CTCCTCAGGGGAGCCAAAGCACTAACAGATATAGTAGCACTGACTGAGGAAGCAGAATTA

GAATTGGCAGAGAACAGGGAAATTCTAAAAGAACCTGTACATGGGGTA

>06-cpx.ML.95

CCTCAGATCACTCTTTGGCAACGACCCCTAGTCACAGTAAGAATAGGGGGACAGCTAATA

GAAGCCCTATTAGACACAGGAGCAGATGATACAGTATTAGAAGACATAAATTTACCAGGA

AAATGGAAACCAAAGATGATAGGGGGAATTGGAGGTTTTATCAAAGTAAGACAGTATGAT

CAAATACTTATAGAAATTTGTGGAAAAAGGGCTATGGGTACAGTATTAGTGGGACCTACA

CCTGTCAACATAATTGGACGAAACATGTTGACCCAGATTGGTTGTACTTTAAATTTTCCA

ATTAGTCCTATTGAAACTGTACCAGTAAAATTAAAGCCAGGAATGGATGGCCCAAAGGTT

AAACAATGGCCATTGACAGAAGAGAAAATAAAAGCATTAACAGAAATTTGTACAGAAATG

GAAAAAGAAGGAAAAATTTCAAGAATTGGGCCTGAAAATCCATACAATACTCCAATATTT

GCTATAAAGAAAAAAGATAGTACTAAATGGAGAAAATTAGTAGATTTCAGAGAACTTAAT

AAAAGAACTCAAGATTTTTGGGAAGTTCAATTAGGAATACCACATCCTGCTGGGCTGAAA

AAGAAAAAATCAGTAACAGTATTGGATGTGGGGGATGCGTATTTTTCAATTCCCTTAGAT

AAAGACTTTAGGAAGTATACTGCATTCACTATACCTAGTACAAATAATGAGACACCAGGG

ATTAGATATCAGTACAATGTACTTCCACAAGGATGGAAAGGATCACCAGCAATATTTCAG

TGTAGCATGACAAAAATCTTAGATCCCTTTAGAATAAAAAATCCAGAACTGGTGATCTAC

CAATACATGGATGATTTATATGTAGGATCTGACTTAGAAATAGGGCAACATAGAGCAAAA

ATAGAGGAGTTAAGAGAACATCTATTAAAATGGGGATTTACCACACCAGATAAAAAACAT

CAGAAAGAACCCCCATTTCTTTGGATGGGGTATGAACTCCATCCTGACAAATGGACCGTG

CAGCCTATACAACTGCCAAACAAGGAGAGCTGGAGTATCAATGATATACAGAAATTAGTG

GGAAAACTAAATTGGGCAAGTCAGATTTATTCAGGGATTAAAGTAAGACAATTATGTAAA

CTCCTCAGGGGAGCAAAAGCACTAACAGACATAGTACCACTAACTGCAGAAGCAGAATTA

GAATTGGCAGAGAACAGGGAAATTCTAAAAGAACCAGTACATGGGGCA

>07-BC.97CN001

CCTCAAATCACTCTTTGGCAACGACCCCTCGTCACAATAAAGATAGGGGGGCAATTAAAG

GAAGCTCTATTAGATACAGGAGCAGATGATACAGTATTAGAAGACCTGAATTTGCCAGGG

AAATGGAAACCAAAAATGATAGGGGGAATTGGAGGTTTTATCAAAGTAAGACAGTATGAA

CAGATACCCATAGAAATTTGCGGACACAAAGCTATAGGTACAGTATTAGTAGGACCTACA

CCTGTCAACATAATTGGAAGAAATCTGTTGACTCAGCTTGGTTGCACTTTAAATTTTCCA

ATCAGTCCCATTGAAACTGTACCAGTAAAATTAAAGCCAGGAATGGATGGCCCAAAGGTT

AAACAATGGCCATTGACAGAAGAGAAAATAAAAGCATTAACAGCAATTTGTGATGAAATG

GAGAAAGAAGGAAAAATTACAAAAATTGGGCCTGAAAATCCATATAACACTCCAATATTT

GCCATAAAAAAGAAGGACAGTACTAAGTGGAGAAAGTTAGTAGATTTCAGGGAACTCAAT

AAAAGAACTCAAGATTTTTGGGAAGTTCAATTAGGAATACCACACCCAGCAGGGTTAAAA

AAGAAAAAATCAGTGACAGTACTGGATGTGGGGGATGCATATTTTTCAATTCCTTTATAT

GAAGACTTCAGGAAGTATACTGCATTCACCATACCTAGTAGAAACAATGAAACACCAGGG

ATTAGGTATCAGTACAATGTACTTCCACAGGGATGGAAAGGATCACCAGCAATATTCCAA

AGTAGCATGACAAAAATCTTAGAGCCTTTTAGAAAACAAAATCCAGACATAGTTATCTAT

CAATACATGGATGATTTGTATGTAGGATCTGACTTAGAGATAGGGCAGCATAGAACAAAA

ATAGAGGAACTGAGACAACATTTGTTGAGGTGGGGATTTACCACACCAGACAAGAAACAT

CAGAAAGAACCTCCATTTCTTTGGATGGGGTATGAACTCCATCCTGACAAATGGACAGTA

CAGCCTATACAGCTGCCAGAAAAAGATAGCTGGACTGTCAATGATATACAAAAGTTAGTG

GGAAAATTAAACTGGGCAAGTCAGATTCATCCTGGAATTAAAGTAAGGCAACTTTGTAAA

CTCCTTAGGGGGGCCAAAGCACTAACAGACATAGTACCACTAACTGAAGAAGCAGAATTA

GAATTGGCAGAAAACAGGGAAATTCTAAAAGAACCAGTACATGGAGTA

>08-BC.97CN6F

CCTCAGATCACTCTTTGGCAGCGACCCCTTGTCTCAATAAAAGTAGGGGGCCAGATAAAA

GAGGCTCTCTTAGACACCGGAGCAGATGATACAGTATTAGAAGAAGTAAATTTGCCAGGA

AAATGGAAACCAAAAATGATAGGAGGAATTGGAGGTTTTATCAAAGTAAGACAATATGAG

CAAATACCTATAGAAATTTGTGGAAAAAAGGCTATAGGTACAGTATTAGTGGGACCCACA

CCTGTCAACATAATAGGAAGAAATATGTTGACCCAGCTTGGATGCACACTAAATTTTCCA

ATCAGTCCCATTGAAACTGTACCAGTAAAATTAAAGCCAGGAATGGATGGCCCAAAGGTT

AAACAATGGCCATTGACAGAAGAAAAAATAAAAGCATTAACAGCAATTTGTGATGAAATG

GAGAAGGAAGGAAAAATTACAAAAATTGGGCCTGACAATCCATATAACACTCCAATATTT

GCCATAAGAAAGAAGGACAGTAGTAAGTGGAGAAAATTAGTAGATTTCAGGGAACTCAAT

AAAAGAACTCAAGATTTTTGGGAAGTTCAATTAGGAATACCACACCCAGCAGGGTTAAAA

AAGAAAAAATCAGTAACAGTCCTGGATGTGGGTGATGCATATTTCTCAGTTCCTTTAGAT

AAAGACTTCAGGAAGTATACTGCATTTACCATACCTAGTGTAAACAATGAGACACCAGGG

ATTAGATATCAGTACAATGTGCTTCCACAGGGATGGAAAGGATCACCAGCAATATTCCAA

TGTAGCATGACAAAAATCTTAGAGCCTTTTAGAAAACAAAATCCAGACATAGTTATCTAT

CAATACATGGATGACTTGTATGTAGGATCTGACTTAGAAATAGGGAAGCATAGAACAAAA

ATAGAGGAACTGAGAGAACATCTGTTAAAGTGGGGATTTACCACACCAGACAAGAAACAT

CAGAAAGAACCTCCATTTCTTTGGATGGGGTATGAACTCCATCCTGACAAATGGACAGTA

CAGCCTATACAGCTGCCAGAAAAGGATAGCTGGACTGTCAATGATATACAGAAGTTAGTG

GGAAAATTAAACTGGGCAAGTCAGATTTACCCAGGAATTAAAGTAAGGCAACTTTGTAAA

CTCCTTAGGGGGGCCAAAGCACTAACAGACATAGTACCACTAACTGAAGAAGCAGAATTA

GAATTAGCGGAAAACAGGGAAATTTTAAAAGAACCAGTACATGGAGCA

>B.RL42

CCTCAAATCACTCTTTGGCAACGACCCCTCGTCACAATAAAGGTAGGAGGGCAATTAAAG

GAAGCTCTATTAGATACAGGAGCAGATGATACAGTATTAGAAGACATGAATTTGCCAGGA

AGATGGAAACCAAAAATGATAGGGGGAATTGGAGGTTTTATCAAAGTAAGACAGTATGAT

CAGATACCCATAGAAATCTGCGGACACAAAGCTATAGGTACAGTATTAGTAGGACCTACA

CCTGTCAACATAATTGGAAGAAATCTGTTGACTCAGCTTGGTTGCACTTTAAATTTTCCC

ATTAGTCCTATTAAAACTGTACCAGTAAAATTAAAACCAGGAATGGATGGCCCAAAAGTT

AAACAATGGCCATTGACAGAAGAAAAAATAAAAGCATTAGTAGAAATTTGTACAGAAATG

GAAAAGGAAGGGAAAATTTCAAAAATCGGGCCTGAAAATCCATACAATACTCCAGTATTT

GCCATAAAGAAAAAAGACAGTACTAAATGGAGAAAATTAGTAGATTTCAGGGAACTTAAT

AAAAGAACTCAAGACTTCTGGGAAGCTCAATTAGGAATACCACATCCCGCAGGGTTAAAA

AAGAAAAAATCCGTAACAGTCCTGGATGTGGGTGATGCATATTTCTCAGTTCCTTTAGAT

AAAGACTTCAGGAAGTATACTGCATTTACCATACCTAGTGTAAACAATGAGACACCAGGG

ATTAGATATCAGTACAATGTGCTTCCACAGGGATGGAAAGGATCACCAGCAATATTCCAA

TGTAGCATGACAAAAATCTTAGAGCCTTTTAGAAAACAAAATCCAGACATAGTTATCTAT

CAATACATGGATGATTTGTATGTAGGATCTGACTTAGAAATAGGGCAGCATAGAGCAAAA

ATAGAGGAACTGAGACAACATCTGTTGAGGTGGGGATTTACCACACCAGACAAAAAACAT

CAGAAAGAACCTCCATTCCTTTGGATGGGTTATGAACTCCATCTTGATAAATGGACAGTG

CAGCCTATAATGCTGCCAGAAAAAGACAGCTGGACTGTCAATGACATACAGAAGTTAGTG

GGAAAGTTGAATTGGGCAAGTCAGATTTATGCAGGGATTAAGGTAAAGGAATTATGTAAA

CTCCTTAGGGGAACCAAAGCACTAACAGAAGTAATACCACTAACAGAAGAAGCAGAGCTA

GAACTGGCGGAAAACAGGGAAATTCTGAAAGAATCAGTACATGGAGTG

>B.FR.HXB2

CCTCAGGTCACTCTTTGGCAACGACCCCTCGTCACAATAAAGATAGGGGGGCAACTAAAG

GAAGCTCTATTAGATACAGGAGCAGATGATACAGTATTAGAAGAAATGAGTTTGCCAGGA

AGATGGAAACCAAAAATGATAGGGGGAATTGGAGGTTTTATCAAAGTAAGACAGTATGAT

CAGATACTCATAGAAATCTGTGGACATAAAGCTATAGGTACAGTATTAGTAGGACCTACA

CCTGTCAACATAATTGGAAGAAATCTGTTGACTCAGATTGGTTGCACTTTAAATTTTCCC

ATTAGCCCTATTGAGACTGTACCAGTAAAATTAAAGCCAGGAATGGATGGCCCAAAAGTT

AAACAATGGCCATTGACAGAAGAAAAAATAAAAGCATTAGTAGAAATTTGTACAGAGATG

GAAAAGGAAGGGAAAATTTCAAAAATTGGGCCTGAAAATCCATACAATACTCCAGTATTT

GCCATAAAGAAAAAAGACAGTACTAAATGGAGAAAATTAGTAGATTTCAGAGAACTTAAT

AAGAGAACTCAAGACTTCTGGGAAGTTCAATTAGGAATACCACATCCCGCAGGGTTAAAA

AAGAAAAAATCAGTAACAGTACTGGATGTGGGTGATGCATATTTTTCAGTTCCCTTAGAT

GAAGACTTCAGGAAGTATACTGCATTTACCATACCTAGTATAAACAATGAGACACCAGGG

ATTAGATATCAGTACAATGTGCTTCCACAGGGATGGAAAGGATCACCAGCAATATTCCAA

AGTAGCATGACAAAAATCTTAGAGCCTTTTAGAAAACAAAATCCAGACATAGTTATCTAT

CAATACATGGATGATTTGTATGTAGGATCTGACTTAGAAATAGGGCAGCATAGAACAAAA

ATAGAGGAGCTGAGACAACATCTGTTGAGGTGGGGACTTACCACACCAGACAAAAAACAT

CAGAAAGAACCTCCATTCCTTTGGATGGGTTATGAACTCCATCCTGATAAATGGACAGTA

CAGCCTATAGTGCTGCCAGAAAAAGACAGCTGGACTGTCAATGACATACAGAAGTTAGTG

GGGAAATTGAATTGGGCAAGTCAGATTTACCCAGGGATTAAAGTAAGGCAATTATGTAAA

CTCCTTAGAGGAACCAAAGCACTAACAGAAGTAATACCACTAACAGAAGAAGCAGAGCTA

GAACTGGCAGAAAACAGAGAGATTCTAAAAGAACCAGTACATGGAGTG

>B.US

CCTCAAATCACTCTTTGGCAACGACCCATCGTCACAGTAAAGATAGGGGGGCAATTAAAG

GAAGCTCTATTAGATACAGGAGCAGATGATACAGTATTAGAAGAAATGAATTTGCCAGGA

AAATGGAAACCAAAAATGATAGGGGGAATTGGAGGTTTTATCAAAGTGAGACAGTATGAT

CAAATACTCATAGAAATCTGTGGACATAAAGCTATAGGTACAGTATTAGTAGGACCTACA

CCTGTCAACATAATTGGAAGAAATCTGTTGACTCAGATTGGTTGCACTTTAAATTTTCCC

ATTAGTCCTATTGAAACTGTACCAGTAAAATTAAAGCCAGGAATGGATGGCCCAAAAGTT

AAACAATGGCCATTGACAGAGGAAAAAATAAAAGCATTGGTAGAAATTTGTACAGAAATG

GAAAAGGAAGGAAAAATTTCCAAAATTGGGCCTGAAAATCCATACAATACTCCAGTATTT

GCCATAAAGAAAAAAGACAGTACTAAATGGAGAAAATTAGTAGATTTCAGAGAACTTAAT

AAGAGAACTCAAGACTTCTGGGAAGTTCAGTTAGGAATACCACATCCTGCAGGGTTAAAA

AAGAAGAAATCAGTAACAGTATTGGATGTGGGTGATGCATATTTTTCAGTTCCCTTAGAT

AAAGAGTTCAGGAAGTATACTGCATTTACCATACCTAGTATAAACAATGAAACACCACGG

ATTAGATATCAGTACAATGTGCTTCCACAAGGGTGGAAAGGATCACCAGCAATATTCCAA

AGTAGTATGACAAAAATCTTAGAGCCTTTTAAAAAACAAAATCCAGAAATAGTTATCTAT

CAATACATGGATGATTTGTATGTAGGATCTGATTTAGAAATAGGGCAGCATAGAATAAAA

ATAGAGGAACTGAGAGAACATCTGTTAAAGTGGGGGTTTACCACACCGGACAAGAAACAT

CAGAAAGAACCTCCATTTCTTTGGATGGGTTATGAACTCCATCCTGATAAATGGACAGTA

CAGCCTATAGTGCTGCCAGAAAAAGACAGCTGGACTGTCAATGACATACAGAAGTTAGTG

GGAAAATTGAATTGGGCAAGTCAGATTTATGCAGGGATTAAAGTAAAGCAATTATGTAAA

CTCCTTAGGGGAACCAAAGCACTAACAGAAGTAGTACAACTAACAAAAGAAGCAGAGCTA

GAACTGGCAGAAAATAGGGAGATTCTAAAAGAACCAGTACATGGAGTG

>C.IN.98IN022

CCTCAAATCACTCTTTGGCAGCGACCCCTTGTCTCAATAAAAGTAGGGGGCCAAATAAAA

GAGGCTCTCTTAGGCACAGGAGCAGATGATACAGTATTAGAAGAAATAAATTTGCCAGGA

AAATGGAAACCAAAAATGATAGGAGGAATTGGAGGTTTTATCAAAGTAAGACAATATGAT

CAAATAACTATAGAAATTTGTGGAAAAAAGGCTATAGGTACAGTATTAGTAGGACCCACA

CCTGTCAACATAATTGGAAGAAATCTGTTGACTCAGCTTGGATGCACACTAAATTTTCCA

ATCAGTCCTATTGAAACTGTACCAGTAAAATTAAAGCCAGGAATGGATGGGCCAAAGGTT

AAACAATGGCCATTGACAGAAGAGAAAATAAAAGCATTAACAGAAATTTGCGAGGAAATG

GAGAAGGAAGGAAAAATTACAAAAATTGGGCCTGAAAATCCATATAACACTCCAATATTT

GCAATAAAAAAGAAGGACAGTACTAAGTGGAGAAAATTAGTAGATTTCAGGGAACTCAAT

AAAAGAACTCAAGATTTTTGGGAAGTTCAATTAGGAATACCACACCCAGCAGGGTTAAAA

AAGAAAAAATCAGTGACAGTACTGGATGTGGGGGATGCATATTTTTCAGTTCCTTTATAT

GAAGACTTCAGGAAGTATACTGCATTCACCATACCTAGTATAAACAATGAAACACCAGGG

ATTAGGTATCAATATAATGTGCTTCCACAGGGATGGAAAGGATCACCATCAATATTCCAG

GCTAGCATGACAAAAATCTTAGAGCCCTTTAGGACACAAAATCCAGAAATAGTCATCTAT

CAATATATGGATGACTTGTATGTAGGATCTGACTTAGAAATAGGGCAACATAGAGCAAAA

ATAGAGGAGTTAAGAGAACATCTGTTAAAGTGGGGATTTACCACACCAGACAAGAAACAT

CAGAAAGAACCTCCATTTCTGTGGATGGGGTATGAACTCCATCCTGACAAATGGACAGTA

CAGCCTATACAGCTGCCAGAAAAAGATAGCTGGACTGTCAATGATATACAGAAGTTAGTG

GGAAAATTAAACTGGGCAAGTCAGATTTACCCAGGAATTAAAGTAAGACAACTTTGTAAA

CTCCTTAGGGGGACCAAAGCACTAACAGATGTAGTACCACTAACTGAAGAAGCAGAATTA

GAATTGGCAGAGAACAGGGAAATTCTAAAAGAACCAGTACATGGAGTA

>G.SE6165

CCTCAAATCACTCTTTGGCAGCGACCCCTAGTCACAGTAAAAATAGGGGGACAGCTAATA

GAAGCCCTATTAGACACAGGAGCAGATGACACAGTATTAGAAGAAATAAATTTACCAGGA

AGATGGAAACCAAAAATGATAGGGGGAATTGGAGGTTTTATCAAAGTAAGACAATATGAT

CAAGTACCTATAGAAATTAGTGGAAAAAAGGCTATAGGGACGATATTAGTAGGACCTACA

CCTATCAACATAATTGGGAGAAATATGTTGACTCAGATTGGTTGCACTTTAAATTTTCCA

ATTAGTCCTATTGAAACTGTACCAGTAAAATTAAAGCCAGGAATGGATGGCCCAAGGGTT

AAACAATGGCCATTGACAGAAGAAAAAATAAAAGCATTAACAGAAATTTGTAAAGAAATG

GAAGAGGAAGGAAAAATTTCAAAAATTGGGCCTGAAAATCCATACAACACTCCAATATTT

GCCATAAAGAAAAAGGACAGTACTAAATGGAGAAAATTGGTAGATTTCAGAGAGCTCAAT

AAAAGAACTCAAGACTTCTGGGAGGTCCAATTAGGAATACCTCATCCTGCAGGGTTAAAA

AAGAAAAAATCAGTAACAGTACTAGATGTGGGGGATGCATATTTTTCAGTTCCCTTAGAT

GAAGACTTTAGAAAGTATACTGCATTCACTATACCTAGTATAAATAATGAGACACCAGGG

GTTAGATATCAGTACAATGTGCTTCCACAGGGATGGAAAGGATCACCAGCAATATTTCAG

AGTAGCATGACAAGAATCTTAGAGCCCTTTAGAGCAAATAATCCAGAAATGGTGATCTAC

CAATACATGGATGATTTATATGTAGGATCTGACTTAGAAATAGGGCAGCATAGAGCAAAA

ATAGAGGAGTTAAGAGAACATCTATTGAAATGGGGGTTTACCACACCAGATAAAAAACAT

CAGAAAGAACCTCCATTCCTTTGGATGGGATATGAGCTCCATCCTGACAAATGGACGGTA

CAACCTATACAGCTGCCAGACAAGGAAAGCTGGACTGTCAATGATATACAAAAGTTAGTG

GGAAAACTAAATTGGGCAAGTCAGATTTATCCAGGGATTAAAGTAACGCACTTATGTAAA

CTCCTTAGGGGGGCCAAAGCACTAACAGACATAGTATCACTGACAGCAGAAGCAGAAATG

GAGTTGGCAGAGAACAGGGAAATTCTAAGAGAACCTGTACATGGAGTC

>07_BC.CN.BJ.MSM.BJ070032-2007.5

CCTCAAATCACTCTTTGGCAACGACCCCTTGTTACAATAAAGATAGGGGGGCAACTAAAG

GAAGCTCTATTAGATACAGGAGCAGATGACACAGTATTAGAAGACATGAATTTGCCAGGG

AAATGGAAACCAAAAATGATAGGGGGAATTGGAGGTTTTATCAAAGTAAGACAGTATGAA

CAGATACCCATAGAAATCTGTGGACATAAAGCTATAGGTACAGTATTAGTAGGACCTACA

CCTGTCAACATAATTGGAAGAAATCTGTTGACTCAGCTTGGTTGTACTTTAAATTTTCCA

ATCAGTCCTATTGAAACTGTACCAGTAAAACTAAAGCCAGGAATGGATGGCCCAAAGGTT

AAACAATGGCCATTGACAAAAGAGAAAATAGAAGCATTAACAGCAATTTGTGATGAAATG

GAAAAGGAAGGAAAAATTACAAAAATTGGGCCTGAAAATCCATACAACACTCCAATATTT

GCCATAAAAAAGAAAGACAGTACTAAGTGGAGAAAATTAGTAGATTTCAGGGAACTCAAT

AAAAGAACTCAAGATTTTTGGGAAGTTCAATTAGGAATACCACACCCAGCAGGATTAAAA

AAGAAAAAATCAGTGACAGTGCTGGATGTGGGGGATGCATATTTTTCAGTTCCTTTATAT

GAAGACTTCAGGAAATATACTGCATTCACCATACCTAGTACAAACAATGAAACACCAGGG

ATTAGGTATCAGTACAATGTACTTCCACAGGGATGGAAAGGATCACCAGCAATATTTCAA

AGTAGCATGACAAAAATCTTAGAGCCTTTTAGAAAACAAAATCCAGACATAGTCATCTAT

CAATACATGGATGATTTGTACGTAGGATCTGACTTAGAGATAGGGCAGCATAGAACAAAA

ATAGAGGAACTGAGACAACATTTGTTGAGGTGGGGATTTACCACACCAGACAAAAAACAT

CAGAAAGAACCTCCATTTCTTTGGATGGGGTATGAACTCCATCCTGACAAATGGACAGTA

CAGCCTATACAGCTGCCAGTACAAGATAGCTGGACTGTCAATGATATACAAAAGTTAGTG

GGAAAATTAAACTGGGCAAGTCAGATTTATCCTGGAATTAAAGTAAGGCAACTTTGTAAA

CTCCTTAGGGGGGCCAAAGCACTAACAGACATAGTACCACTAACTGAAGAAGCAGAATTA

GAATTGGCAGAAAATAGGGAAATTCTAAAAGAATCAGTACATGGAGT-

>07_BC.CN.HEB.X.1114-2008.5

CCTCAAATCACTCTTTGGCAACGACCCCTTGTCACAATAAAGATAGGGGGGCAATTAAAG

GAAGCTCTATTAGATACAGGAGCAGATGATACAGTATTAGAAGACATGAATTTGCCAGGG

AAATGGAAACCAAAAATGATAGGGGGAATTGGAGGTTTTATCAAAGTAAGACAGTATGAA

CAGATACCCGTAGAAATTTGTGGACATAAAGCTATAGGTACAGTATTAGTAGGACCTACA

CCTGTCAACATAATTGGAAGAAATCTGTTGACTCAGCTTGGTTGCACTTTAAATTTTCCA

ATCAGTCCTATTGAAACTGTACCAGTAAAATTAAAGCCAGGAATGGATGGCCCAAAGGTA

AAACAATGGCCATTGACAAAAGAGAAAATAGAAGCATTAACAGCAATTTGTGATGAAATG

GAAAAGGAAGGAAAAATTACAAAAATTGGGCCTGAAAATCCATACAACACTCCAATATTT

GCCATAAAAAAGAAGGACAGTACTAAGTGGAGAAAATTAGTAGATTTCAGGGAACTCAAT

AAAAGAACTCAAGATTTCTGGGAAGTTCAGTTAGGAATACCACATCCAGCAGGGTTAAAA

AAGAAAAAATCAGTGACAGTACTGGATGTGGGGGATGCATATTTTTCAGTTCCTTTATAT

GAAGACTTCAGGAAATATACTGCATTCACCATACCTAGTATAAACAATGAAACACCAGGG

ATTAGGTATCAGTACAATGTACTTCCACAGGGATGGAAAGGATCACCAGCAATATTTCAA

AGTAGCATGACAAAAATCTTAGAGCCTTTTAGAAAACAAAATCCAGACATAGTCATCTAT

CAATACATGGATGATTTGTATGTAGGATCTGACTTAGAGATAGGGCAGCATAGAACAAAA

ATAGAGGAACTGAGACAACATTTGTTGAGGTGGGGATTTACCACACCAGACAAGAAGCAC

CAGAAAGAACCTCCATTTCTTTGGATGGGGTATGAACTCCATCCTGACAAATGGACAGTA

CAGCCTATACAGCTGCCAGTACAAGATAGCTGGACTGTCAATGATATACAAAAGTTAGTG

GGAAAATTAAACTGGGCAAGTCAGATTTATCCTGGAATTAAAGTAAGGCAACTTTGTAAA

CTCCTTAGGGGGGCCAAAGCACTAACAGACATAGTACCACTAACTGAAGAAGCAGAACTA

GAATTGGCAGAAAACAGGGAAATTCTAAAAGAACCGGTACATGGAGT-

>CRF07_BC.CN.05.XJDC6441.EF368370

CCTCAGATCACTCTTTGGCAACGACCCCTCGTCACAATAAAGATAGGGGGGCAGTTAAAA

GAAGCTCTATTAGATACAGGAGCAGATGATACAGTATTAGAAGACATGAATTTGCCAGGG

AAATGGAAACCAAAAATGATAGGGGGAATTGGAGGTTTTATCAAAGTAAGACAGTATGAC

CAGGTACCTATAGAAATTTGTGGACACAAAGCTATCGGTACAGTATTAGTAGGACCTACA

CCTGTCAACATAATTGGAAGAAATCTGTTGACTCAGCTTGGTTGTACTTTAAATTTTCCA

ATTAGTCCCATTGAAACTGTACCAGTAAAATTAAAGCCAGGAATGGATGGCCCAAAGGTT

AAACAATGGCCATTGACAGAAGAGAAAATAAAAGCATTAACAGCAATTTGTGATGAGATG

GAGAAGGAAGGAAAAATTACAAAAATTGGGCCTGAAAATCCATATAACACTCCAATATTT

GCCATAAAAAAGAAGGACAGTACTAAGTGGAGAAAGTTAGTAGATTTCAGGGAACTCAAT

AAAAGAACTCAAGACTTTTGGGAAGCTCAATTAGGAATACCACACCCAGCAGGGTTAAAA

AAGAAAAAATCAGTGACAGTACTGGATGTGGGGGATGCATATTTTTCAGTCCCTTTATAT

GAAGACTTCAGGAAATATACTGCATTCACCATACCTAGTATAAACAATGAAACACCAGGG

ATTAGGTATCAGTACAATGTACTTCCACAGGGATGGAAAGGATCACCAGCAATATTCCAA

AGTAGCATGACAAAAATCTTAGAGCCTTTTAGAAAACAAAATCCAGACATAGTTATCTAT

CAATACATGGATGATTTGTATGTAGGATCTGACTTAGAGATAGGGCAGCATAGAACAAAA

ATAGAGGAACTGAGACAACATTTGTTGAGGTGGGGATTTACCACACCAGACAAGAAACAT

CAGAAAGAACCTCCATTTCTTTGGATGGGGTATGAACTCCATCCTGACAAATGGACAGTT

CAGCCTATACTGTTGCCAGAAAAAGAAAGCTGGACTGTCAATGATATACAAAAGTTAGTG

GGAAAATTAAACTGGGCAAGTCAGATTTATCCTGGAATTAAAGTAAGGCAACTTTGTAAA

CTCCTTAGGGGGACCAAAGCACTAACAGACATAGTACCACTAACTGAAGAAGCAGAATTA

GAATTGGCAGAAAACAGGGAAATTCTAAAAGAACCAGTACATGGGGT-

>CRF07BC.1-12ZJ0556

cctcaaatcactctttggcaacgaccccttgttaccataaagataggagggcaattaaag

gaagctctattagatacaggagcagatgatacagtattagaagacattgatttgccaggg

aaatggaaaccaaaaatgataggaggaattggaggttttatcaaagtaagacagtatgaa

cagatacccatagaaatctgtggacataaagctataggtacagtattagtaggacctaca

cctgtcaacataattggaagaaatctgttgactcagcttggttgtactttaaattttcca

atcagtcctattgaaactgtaccagtaaaactaaagccaggaatggatggcccaaaggtt

aaacaatggccattgacaaaagagaaaatagaagcattaacagcaatttgtgatgaaatg

gaaaaggaaggaaaaattacaaaaattgggcctgaaaatccatacaacactccaatattt

gccataaaaaagaaagacagtactaagtggagaaaattagtagatttcagggaactcaat

aaaagaactcaagatttttgggaagttcaattaggaataccacacccagcaggattaaaa

aagaaaaaatcagtgacagtgctggatgtgggggatgcatatttttcagttcctttatat

gaagacttcaggaaatatactgcattcaccatacctagtataaacaatgaaacaccaggg

attaggtatcagtacaatgtacttccacagggatggaaaggatcaccagcaatatttcaa

agtagcatgacaaaaattttagagccttttagaaaacaaaatccagacatagtcatctat

caatacatggatgatttgtatgtaggatctgacttagaaatagggcagcatagaacaaaa

atagaggaactgagacaacatttgtttaggtggggatttaccacaccagacaagaaacat

cagaaagaacctccatttctttggatggggtatgaactccatcctgacaaatggacagta

cagcctatacagctgccagtacaagatagctggactgtcaatgatatacaaaagttagtg

ggaaaattaaactgggcaagtcagatttatcctggaattaaagtaaggcaactttgtaaa

ctccttaggggggccaaagcattaacagacatagtaccactaactgaagaagca------

------------------------------------------------

>CRF07BC.11-12ZJ0501

cctcagatcactctttggcaacgacccctcgtcccaataaaggtaggggggcaatyaaag

gaagctctattagatacaggagcagatgatacagtattagaagacatgaatttgccaggg

aaatggaaaccaaaaatgatagggggaattggaggttttatcaaagtaaaacaatatgaa

cagatatccatagaaatctrtggacacaaaactatcggtacagtattagtaggacctaca

cctgtcaacataattggaaggaatctgttaactcagcttggttgcactttaaattttcca

atyagtcctattgaaactgtaccagtaaaattaaagccaggaatggatggcccaaaggtt

aaacaatggccattgacagaagagaaaataaaagcattaacagcaatttgtgatgaaatg

gagaaggaagggaaaattacaaaaattgggcctgaaaatccatataacactccaatattt

gccataaaaaagaaggacagtactaagtggagaaaattagtagatttcagggaactcaat

aaaagaactcaagatttttgggaagttcaattaggaataccacacccagcagggttaaaa

aagagaaaatcagtgacagtactggatgtgggggatgcatatttttcagttcctttatat

gaagatttcaggaaatatactgcattcaccatacctagtgtaaacaatgaaacaccaggg

attaggtatcagtacaatgtacttccacagggatggaaaggatcaccagcaatattccaa

actagcatgacaaagatcttagagccttttagaaagcaaaatccagacatagttatctat

caatacatggatgatttgtatgtaggatctgacttagaaatagggcagcatagaacaaaa

atagaggaactgagacaacacttgttgaggtggggatttaccacaccagacaagaaacat

cagaaagaacctccatttctttggatggggtatgaactccaccctgacaaatggacagta

cagcctatacagctaccagaaaaagatagctggactgtcaatgatatacaaaagttagtg

ggaaaattaaattgggcaagtcagatttatcctggaattaaagtaaggcaactttgtaaa

ctccttaggggggccaaaacactaacagaaatagtaccactaactgaagaa---------

------------------------------------------------

>CRF07BC.11-TT120971

cctcaaatcactctttggcaacgaccccttgttacaataaagataggggggcaattaaag

gaggctctattagatacaggagcagatgatacagtattagaagatatgaatttgccaggg

aaatggaaaccaaaaatgatagggggaattggaggttttatcaaagtaagacagtatgaa

caggtacccatagaaatctgtggacataaagctataggtacagtattaataggacctaca

cctgtcaacataattggaagaaatctgttgactcagcttggttgtactttaaattttcca

atcagtcctattgaaactgtaccagtaaaactaaagccaggaatggatggcccaaaggtt

aaacaatggccattgacaaaagagaaaatagaagcattaacagcaatttgtgatgaaatg

gaaaaggaaggaaaaattacaaaaattgggcctgaaaatccatacaatactccaatattt

gccataaaaaagaaagacagtactaagtggagaaaattagtagatttcagggaactcaat

aaaagaactcaagatttttgggaagttcaattaggaataccacacccagcaggattaaaa

aagaaaaratcagtgacagtgctggatgtgggggatgcatatttttcagttcctttacat

gaagacttcaggaaatatactgcattcaccatacctagtacaaacaatgaaacaccaggg

attaggtatcagtacaatgtacttccacagggatggaaaggatcaccagcaatatttcaa

agtagcatgacaaaaatcttagagccttttagaaaacaaaatccagacatagtcatctat

caatacatggatgatttgtatgtaggatctgacttagagatagggcagcatagaacaaaa

atagaagaactgagacaacatttgttgaggtggggatttaccacaccagacaagaaacat

cagaaagaacctccatttctttggatggggtatgaactccatcctgacaaatggacagta

cagcctatacagctgccagtacaagatagctggactgtcaatgatatacaaaagttagtg

ggaaaattaaactgggcaagtcagatttatcctggaattaaagtaaggcaactttgtaaa

ctccttaggggggccaaagcactaacagacatagtaccactaactgaagaagcagaa---

------------------------------------------------

>CRF07BC.111409015913

cctcaaatcactctttggcaacgaccccttgtcacaataaagataggggggcaattaaag

gaagctctattagatacaggagcagatgatacagtattagaagacatgaatttgccaggg

aaatggaaaccaaaaatgatagggggaattggaggttttatcaaagtaagacagtatgaa

cagatacccatagaaatctgtggacakaaagctataggtacagtattagtaggacctaca

cctgtcaacataattggaagaaatctgttgactcagcttggttgtactttaaattttcca

atcagtcctattgaaactgtaccagtaaaactaaagccaggaatggatgggccaaaggtt

aaacaatggccattgacaaaagagaaaatagaagcattaacagcaatttgtgatgaaatg

gaaaaggaaggaaagattacaaaaattgggcctgaaaatccatacaatactccaatattt

gccataaaaaagaaagacagtactaartggagaaaattagtagatttcagggaactcaat

aaaagaactcaagatttttgggaagttcaattaggaataccacacccagcaggattaaaa

aagaaaaaatcagtgacagtgctggatgtgggggatgcatatttttcagttcctttacat

gaagacttcaggaaatatactgcattcaccatacctagtataaacaatgaaacaccaggg

attaggtatcagtacaatgtacttccacagggatggaaaggatcaccagcaatatttcaa

agtagcatgacaaaaatcttagagccttttagaaarcaaaatccagacatagtcatctat

caatacatggatgatttgtatgtaggatctgacttagagatagggcagcatagaacaaaa

atagaggaactgagacaacatttgttgaggtggggatttaccacaccagacaagaaacat

cagaaagaacctccatttctgtggatggggtatgaactccatcctgacaaatggacagta

cagcctatacagctaccagtacaagatagctggactgtcaatgatatacaaaagttagtr

ggaaaattaaactgggcaagtcaratttatcctggaattaaagtaaggcaactttgtaaa

ctccttaggggggccaaagcactaacagacatagtaccactaactgaagaagca------

------------------------------------------------

>CRF07BC.111415001059

cctcaaatcactctttggcaacgaccccttgttccaataaagatagggggacagttaaaa

gaagctctattagatacaggagcagatgatacagtattagaagacatgaatttgccaggg

aaatggaaaccaaaratgatagggggaattggaggttttatcaaagtaagacagtatgaa

cagatacccctagaaatctgtggacataaagctataggtacagtattagtaggacctaca

cckgtcaacataattggaaggaatctgttgactcagcttggttgtactttaaattttcca

atcagtcctattgaaactgtaccagtaaaactaaagccaggaatggatggcccaaaggtt

aaacaatggccattgacaaaagagaaaatagaagcattaacagcaatttgtgatgaaatg

gaaaaggaaggaaaaattacaaaaattgggcctgaaaatccatacaacactccaatattt

gccataaaaaagaaagacagtactaagtggagaaaattagtagatttcagggaactcaat

aaaagaactcaagatttttgggaggttcaattaggaataccacacccagcaggattaaaa

aggaaaaaatcagtgacagtgctggatgtgggggatgcatatttttcagttcctttatat

gaagacttcaggaaatatactgcattcaccatacctagtataaacaatgaaacaccaggg

attaggtatcagtacaatgtacttccacagggatggaaaggatcaccagcaatatttcaa

agtagcatgacaaaaatcttagagccttttagaaaacaaaatccagacatagtcatctat

caatacatggatgatttgtatgtaggatccgacttagagatagggcagcatagaacaaaa

atagaggaactgagacaacatttgttgaagtggggatttaccacaccagacaagaaacat

cagaaagaacccccatttctttggatggggtatgaactccatcctgacaaatggacagta

cagcctatacagctgccagtacaagatagctggactgtcaatgatatacaaaagttagtg

ggaaaattaaactgggcaagtcaaatttatcctggaattaaagtaaggcaactttgtaaa

ctccttaggggggccaaagcactaacagacatagtaccactaactgaagaagcagaa---

------------------------------------------------

>CRF07BC.111420000019

cctcaaatcactctttggcaacgaccccttgtcycaataargatagggggrcaattaaag

gaagctctattagatacaggagcagatgatacagtattagaagacatggatttgccaggg

aaatggaaaccaaaaatgatagggggaattggaggttttatcaaagtaagacagtatgaa

caratacccatagaaatctgtggacataaagctataggtacagtattartrggacctaca

cctgtcaacataattggaagaaatctgttgactcagcttggttgtactttaaattttcca

atcagtcctattgaaactgtaccagtaaaactaaagccaggaatggatggcccaaaggtk

aaacaatggccattgacaaaagaraaaatagaagcattaayrgcaatttgtgatgaaatg

gaaaaggaaggaaaaattacaaaaattgggcctgaaaatccatacaacactccaatattt

gccataaaaaagaargacagtactaagtggagaaaattagtagatttcagggaactcaat

aaaagaactcaagatttttgggargttcaattaggaataccacacccrgcaggattaaaa

aagaaaaaatcagtgacagtgctggatrtaggggatgcatatttttcagttcctttatat

gaagayttcaggaaatatactgcattcaccatacctagyataaacaatgaaacaccaggg

attaggtatcagtacaatgtacttccacagggatggaaaggatcaccagcaatatttcaa

agtagcatgacaaaaatcttagagccttttagaaaacaaaatccagacatagtcatctat

caatacatggatgatttgtatgtaggatctgacytagaaatagggcagcatagaacaaaa

atagaggaactgagacaacatttgttgargtggggayttaccacaccagacaagaaacay

cagaaagaacctccatttctttggatggggtatgarctccatcctgacaaatggacagta

cagcctatacagctgccagwacaagatagctggactgtcaatgatatacaaaarttagtg

ggaaaaytaaactgggcaagtcagatttatcctggaattaaagtaaggcaactttgtaaa

ctccttaggggggccaaagcactaacagacatagtaccactaactg--------------

------------------------------------------------

>CRF07BC.12-12ZS85

cctcaaatcactctttggcaacgacccctcgtcgccataaagataggggggcaagtaaag

gaagctctattagatacaggagcagatgatacagtattagaagacatgaatttgccaggg

aaatggaaaccaaaaatgatagggggaattggaggttttatcaaagtaagacagtatgaa

cagacacccatagaaatttgcgggcacaaagttataggtacagtattagtaggacctaca

cctgtcaacataattggaagaaatctattgactcagcttggttgcactttaaattttcca

atcagtcccattgaaactgttccagtaaaattaaagccaggaatggatggcccaaaggtt

aaacaatggccgttgacagaagagaaaataaaagcattaacagcaatttgtgatgaaatg

gagaaggaaggaaaaattacaaaaattggacctgaaaatccatataacactccaatattt

gcaataaaaaagaaggacagtactaagtggagaaaattagtggatttcagggaactcaat

aaaagaacccaggatttttgggaggttcaattaggaataccacacccagcagggttaaaa

aagagaaaatcagtgacagtactagatgtgggggatgcatatttttcagttcctttatat

gaagacttcaggaaatatactgcattcaccatacctagtataaacaatgaaacaccaggg

attaggtatcagtacaatgtacttccacagggatggaaaggatcaccagcaatattccaa

tgtagcatgacaaaaatcttagagccttttagaaaacaaaatccagacatagttatctat

caatacatggatgatttgtatgtaggctctgacttagagatcgggcagcatagaacaaaa

atagaagaactgagacaacatttgttgaagtggggacttaccacaccagacaagaaacat

cagaaagaacctccatttctttggatggggtatgagctccatcctgacaaatggacagta

cagcctatacagctgccagaaaaagatagctggactgtcaatgatatacaaaagttagtg

ggaaaattaaactgggcaagtcagatttatcctggagttaaggtaaagcaactttgtaaa

ctccttagaggggccaaagcactaacagacatagtaccactaactgaagaagcaga----

------------------------------------------------

>CRF07BC.13-LC004

cctcaaatcactctttggcaacgaccccttgttaccataaagataggggggcaattgaag

gaagctctattagatacaggagcagatgatacagtattagaagacatgaatttgccaggg

aaatggaaaccaaaaatgatagggggaattggaggttttatcaaagtaagacagtatgaa

cagatacccatagaaatctgtggacataaagctataggtacagtattaataggacctaca

cctgtcaacataattggaagaaatctgttgactcagcttggttgtactttaaattttcca

atcagtcctattgaaactgtaccagtaaaactaaagccaggaatggatggcccgaaggtt

aaacaatggccattgacaaaagagaaaatagaagcattaacagcaatttgtgatgaaatg

gaaaaggaaggaaaaattacaaaaattgggcctgaaaatccatacaacactccaatattt

gccataaaaaagaaagacagtactaagtggagaaaattagtagatttcagggaactcaat

aaaagaactcaagatttttgggaagttcaattaggaataccacacccagcaggattaaaa

aagaaaaaatcagtgacagtgctggatgtgggagatgcatatttttcagttcctttagat

aaagacttcaggaaatatactgcattcaccatacctagtataaacaatgaaacaccaggg

attagatatcagtacaatgtacttccacagggatggaaaggatcaccagcaatatttcaa

agtagcatgacaaaaatcttagagccttttagaaaacaaaatccagacatagtcatctat

caatacatggatgatttgtatgtaggatctgacttagagatagggcagcatagagcaaaa

atagaggaactgagacaacatttgttgaggtggggatttaccacaccagacaagaaacat

cagaaagaacctccatttctttggatggggtatgaactccatcctgacaaatggacagta

cagcctatacagctgccagtacaagatagctggactgtcaatgatatacaaaagttagtg

ggaaaattaaactgggcaagtcagatttatcctggaattagagtaagacaactttgtaaa

ctccttaggggggccaaagcactaacagacatagtaccactaactgaagaagcagaatta

gaattggcaga-------------------------------------

>CRF07BC.13BDZL720

cctcaaatcactctttggcaacgaccccttgtcacaataaagatagggggacaattaaag

gaagctctattagatacaggagcagatgatacagtattagaagacatgaatttgccaggg

aaatggaaaccaaaaatgatagggggaattggaggttttatcaaagtaagacagtatgaa

cagatacccatagaaatctgtggacataaagctataggtacagtattagtaggacctaca

cctgtcaacataattggaagaaatctgttgactcagcttggttgtactttaaattttcca

atcagtcctattgaaactgtaccagtaaaaytaaagccaggaatggatggcccaaaggtt

aaacaatggccattgacaaaagagaaaatagaagcattaacaacaatttgtgaggaaatg

gaaaaagaaggaaaaattacaaaaattgggcctgaaaatccatacaacactccaatattt

gccataaaaaagaaagacagtactaagtggagaaaattagtagatttcagrgaactcaat

aaaagaactcaagacttttgggaagttcaattaggaataccacacccagcgggattaaaa

aagaaaaaatcagtgacagtgctggatgtgggggatgcatatttttcagttcctttatat

gaagacttcaggaaatatactgcattcaccatacctagtataaacaatgaaacaccaggg

attaggtatcagtacaatgtacttccacaaggatggaaagggtcaccagcaatatttcaa

agtagcatgacaaaaatcttagagccttttagaaaacaaaatccagacatagtcatctat

caatacatggatgatttgtatgtaggatctgacttagagatagggcagcatagaacaaaa

atagagraactgagacaacatttgttaaggtggggatttaccacaccagacaagaaacat

cagaaagaacctccatttctttggatggggtatgaactccatcctgacaaatggacagta

cagcctatacagctgccagtacaagatagctggactgtcaatgatatacaaaaattagtg

ggaaaattaaactgggcaagtcagatttatccgggaattaaagtaagacaactttgtaaa

ctccttaggggggccaaagcactaacagacataataccactaactgaagaagcagaa---

------------------------------------------------

>CRF07BC.13WZ20130055

cctcaaatcactctttggcaacgacccctcgtcccaataaaggtaggagggcaagtgaag

gaagctctattagatacaggagcagatgatacagtattagaagacataaatttgccagga

aaatggaaaccaaaaatgatagggggaattggaggttttatcaaagtaagacagtatgaa

cagatatccatagaaatctgcggacacaaagctataggtacagtattagtaggacctaca

cctgtcaacataattggaagaaatctgttgactcagcttggttgcactttaaattttcca

attagtcccattgaaactgtaccagtaaaattaaagccaggaatggatggcccaagggtt

aaacaatggccattgacagaagagaaaataaaagcactaatggaaatttgtgatgaaatg

gaaaaggaaggaaaaattacaaaaattgggcctgaaaatccatataacactccaatattt

gccataaaaaagaaggacagtactaagtggagaaaattagtagacttcagggaactcaat

aaaagaactcaagatttttgggaagttcaattaggaataccacacccagcagggttaaaa

aagagaaaatcagtgacagtactggatgtgggggatgcatatttttcagttcctttatat

gaagacttcaggaaatatactgcattcaccatacctagtacaaacaatgaaacaccaggg

attaggtatcagtacaatgtacttccacagggatggaaaggatcaccagcaatattccaa

agtagcatgacaaaaatcttagagccttttagaaaacaaaatccagacataattatctat

caatacatggatgatttgtatgtaggatctgacttagagatagggcagcatagaacaaaa

atagaagaactgagacaacatttgttgaggtggggatttaccacaccagataagaaacat

cagaaagaacctccatttctttggatggggtatgaactccatcctgacaaatggacagta

cagcctatacagctaccagaaaaagatagctggactgtcaatgatatacaaaagttagtg

ggaaaattaaactgggcaagtcagatttatcctggaattaaagtaaggcaactttgtaaa

ctccttaggggggccaaagcactaacagacatagtaccactaactgaagaagcagaa---

------------------------------------------------

>CRF07BC.14-12ZJ0663

cctcaaatcactctttggcaacgaccccttgtcacaataaagataggggggcaattaaag

gaagctctattagatacaggagcagatgatacagtactagaagacatggatttgccaggg

aaatggaaaccaaaaatgatagggggaattggaggttttatcaaagtaagacagtatgaa

caaatacccatagaaatctgtggacataaagctataggtacagtattagtaggacctaca

cctgtcaacataattggaagaaatctgttgactcagcttggttgtactttaaattttcca

atcagtcctattgaaactgtaccagtaaaactaaagccaggaatggatggcccaaaggtt

aaacaatggccattgacaaaagagaaaatagaagcattaacagcaatttgtgatgaaatg

gaaaaggaaggaaaaattacaaaaattgggcctgaaaatccatacaacactccaatattt

gccataaaaaagaaagacagtactaagtggagaaaattagtagatttcagggaactcaat

aaaagaactcaagatttttgggaagttcaattaggaataccacacccagcaggattaaaa

aagaaaaaatcagtgacagtgctggatgtgggggatgcatatttctcagttcctttatat

gaagacttcaggaaatacactgcattcaccatacctagtgtaaacaatgaaacaccaggg

attaggtatcagtacaatgtacttccacagggatggaaaggatcaccagcaatatttcaa

agtagcatgacaaaaatcttagagccttttagaaagcaaaatccagacatagtcatctat

caatacatggatgatttgtatgtaggatctgacttagagatagggcagcatagaacaaaa

atagaggaactgagacaacatttgttggggtggggatttaccacaccagacaagaaacat

cagaaagaacctccatttctttggatggggtacgaactccatcctgacaaatggacagta

cagcctatacagctgccagtacaagacagctggactgtcaatgatatacagaagttagtg

ggaaaattaaactgggcaagtcagatttatcctggaattaaagtaaggcaactttgcaaa

ctccttagggggaccaaagcactaacagacatagtaccactaactgaagaagcagaa---

------------------------------------------------

>CRF07BC.14-HuZ12200

cctcaaatcactctttggcagcgaccccttgtcacaataaagataggggggcaattaaag

gaagctctcttagatacaggagcagatgatacagtaktagaagacatgaatttgccaggg

aaatggaaaccaaaaatgatagggggaattggaggttttatcaaagtaagacagtatgaa

cagatacccatagaaatctgtggacacaaagctatagggacagtattagtagggccaacg

cctgtcaacataattggaagaaatttgttgactcagattggttgcactytaaattttcca

atcagtcccattgaaactgtaccagtaaaattaaagccaggaatggatggcccaaaggtt

aaacaatggccattgacagaagagaaaataaaagcattaacagcaatttgtgargaaatg

gagaaggaaggaaaaattacaaaaattgggcctgaaaatccatataacactccaatattt

gcyataaaaaagaaagacagtactaagtggagaaagttagtagatttcagggaactcaat

aaaagaactcaagatttttgggaggttcaattaggaataccacatccagcagggttaaaa

aagaaaaaatcagtaacagtactggatgtgggrgatgcatatttttcagttcctttatat

gaagacttcaggaaatatactgcattcaccatacctagtataaacaatgaaacaccaggg

attaggtatcagtacaatgtacttccacagggatggaaaggatcaccagcaatattccaa

agtagcatgacaaaaatcttagagccttttagaaaacaaaatccagatatagttatctat

caatacatggatgatttgtatgtaggatctgacttagagatagggcagcatagaacaaaa

atagaggaactgagagaacatttgttgaggtggggatttaccacaccagacaagaaacat

cagaaagaacctccatttctttggatggggtatgaactccatcctgacaaatggacagta

cagcctatacatctgccagaaaaagatagctggactgtcaatgatatacaaaagttagtg

ggaaaattaaaytgggcaagtcagatttatcckggaattaaagtaagacaactttgtaaa

ctccttagggggaccaaagcactaacagacatagtaccactaactgaagaagcagaat--

------------------------------------------------

>CRF07BC.14-LC001

cctcaaatcactctttggcaacgaccccttgttaccataaagataggggggcaattaaag

gaagctctattagatacaggagcagatgatacagtattagaagacatgaatttgccaggg

aaatggaaaccaaaaatgatagggggaattggaggttttatcaaagtaagacagtatgaa

cagatacccatagaaatctgtggacataaagctataggtacagtattagtaggacctaca

cctgtcaacataattggaagaaatctgttgactcagcttggttgtactttaaattttcca

atcagtcctattgaaactgtaccagtgaaactaaagccaggaatggatggcccaaaggtt

aaacaatggccattgacaaaagagaaaatagaagcattaacagcaatttgtgatgaaatg

gaaaaggagggaaaaattacaaaaattgggcctgaaaatccatacaacactccaatattt

gccataaaaaagaaagacagtactaagtggagaaaattagtagatttcagggagctcaat

aaaagaactcaagatttttgggaagttcaattaggaataccacacccagcaggaytaaaa

aagaaaaaatcagtgacagtgctggatgtgggggatgcatatttttcagttcctttatat

gaagacttcaggaaatacactgcattcaccatacctagtacaaacaatgagacaccagga

attaggtatcagtacaatgtgctaccacagggatggaaaggatctccggcaatattccag

tgtagcatgacaaaaatcttagagccctttagaagaaaaaatccagagataattatctat

caatacatggatgacttgtatataggatctgatttagaaatagggcaacacagaacaaaa

ataraggagctaagagctcatctattgagctggggatttaccacaccagacaagaaacat

cagaaagaacctccatttctttggatggggtatgaactccatcctgacaaatggacagta

cagcctatacagctgccagtacaagatagctggactgtcaatgatatacaaaagttggtg

ggaaaattaaactgggcaagtcagatttatcctggaattaaagtaaggcaactttgtaag

ctccttaggggggccaaagcactaacagacatagtaccactaactgaagaagcagaa---

------------------------------------------------

>CRF07BC.14BDZL030

cctcaaatcactctttggcaacgaccccttgtcacaataaarataggrgrrcaattaara

gaagctctattagatacaggagcagatgatacagtattagaagayataaatttgccaggr

aaatggaaaccaaaaatgatagggggaattggaggytttatcaaagtaagrcartatgaw

cagataccyatagaaatytgtggamawmargytataggyacagtrttartaggacctaca

cctgtcaacataattggamgaaatmtgttgactcagcttggttgtactttaaattttcca

atcagtcctattgaaactgtaccagtaaaaytaaagccaggaatggatggmccaaaggtt

aaacaatggccattgacaaaagaaaaaatagaagcattaacagmaatttgtgawgaaatg

gaaaaggaaggaaaaattacaaaaattgggcctgaaaatccatacaayactccaatattt

gccataaaaaagaaggacagtactaagtggagaaaattagtagatttcagggaactcaat

aaaagaactcaagatttttgggaagttcaattaggaataccrcayccagcaggattaaaa

magaaaaaatcagtgacagtgctrgatgtrggggatgcatatttttcagttcctttacat

gaaractttagraartatactgcattcaccatacctagtataaacaatgaracaccaggr

atmagrtatcagtacaatgtrctwccacarggatggaaaggatcwccrgcaatattycar

wgtagcatgacaaraatcttagarycytttagaaaacagaatccagasatrgtyatytat

caatacatggatgayttgtatgtaggatctgayttagaratagggcagcatagaacaaaa

atagaggarctragasmwcatytrttgagstggggatttacyacaccagacaaraaacat

cagaargaacctccatttctttggatgggrtatgaactccatccwgayaratggacagtm

cagcctatasarctgccagwamaagatagctggactgtcaatgatatacaraarttagtg

ggaaaaytaaaytgggcaagtcaratttatccwggrattarrgtaargcaaytgtgtaaa

ctcctyaggggracyaaagcrctaacagacatagtaccactractgaagaagcagaatta

gaa---------------------------------------------

>CRF07BC.14BDZL1000

cctcaaatcactctttggcaacgacccctagttamaataaagataggggggcaattaaar

gaagctctattagatacaggagcagatgatacagtattagaagacatgaatttgccaggg

aaatggaaaccaaaaatgatagggggaattggaggttttatcaaagtaagacagtacgac

cagatacccatagaaatttgtggacataaagytataggtacagtattagtaggacctaca

cctgtcaacataattggaagaaatctgttaactcagcttggttgtactttaaattttcca

atcagycctattgaaactataccagtacaactaaagccaggaatggatggcccaaaggtt

aaacaatggccattgacaaaagagaaaatagaagcattaacagcaatttgtgatgaaatg

gaaaaggaaggaaaaattacaaaaattgggcctgaaaayccatacaacactccaatattt

gccataaaaaagaaagacagtactaagtggagaaaattagtagatttcagggaactcaat

aaaagaactcaagatttttgggaagttcaattaggaataccacacccagcaggattaaaa

aagaaaaartcagtgacagtrctrgatgtgggggatgcatatttttcagttcctttatat

gaagacttcaggaaatatactgcattcaccatacctagtacaaacaatgaaacaccaggg

attaggtatcagtacaatgtacttccacagggatggaaaggatcaccagcaatatttcaa

agtagcatgacaaaaatcttagagccttttagaaaacaaaatccagacatagtcatctat

caatacatggatgatttgtatgtaggatctgacttagagataggrcagcatagaataaaa

atagaggaactgagacaacatttgttgargtggggatttaccacaccagacaagaarcat

cagaaagaacccccatttctttggatggggtatgaactccatcctaacaaatggacagta

cagcctatacagytgccagtacaagatagctggactgtcaatgatatacaaaagttagtg

ggaaaattaaactgggcaagtcagatttatcctggaattaaagtaaggcaactttgtaag

ctccttcggggggccaaagcactaacagacatagtaccactaactgaagaagcagaatta

gaattggcaga-------------------------------------

>CRF07BC.14BDZL1021

cctcaaatcactctttggcaacgacccctcgtcacaataaagatagggggacaattaaag

gaagccttattagatacaggggcagatgatacagtattagaagacatagatttgccaggg

aaatggaaaccaaaaatgatagggggaattggaggttttatcaaagtaaaacagtatgaa

gaaatacctatagaaatctgtggacacaaagctataggtacagtattrgtaggacctaca

cctgtcaacataattggaagaaatctgttgactcaaattggttgcactttaaattttcca

attagtcccattgaractataccagtaaaattaaagccaggaatggatggcccaagggtt

aaacaatggccattatcagaagagaaaataaaagcattaacagcaatttgtgaggaaatg

gagaaggaaggaaaaattacaaaaattgggcctgaaaatccatataacactccaatattt

gccataaaaargaaggacagtactaagtggagaaaattagtagatttcagggaactcaat

aaaagaactcaagatttttgggaagttcaattaggaataccacacccagcagggttamaa

aagaaaaaatcaatgacagtactggatgtaggggatgcatatttttcagttcctttatat

gaagacttcaggaaatatactgcattcaccatacctagtatraacaatgaaacaccaggg

attaggtatcagtataatgtacttccacagggatggaaaggatcaccagcaatattccaa

agtagcatgacaaaaatcttagagccttttagaaaacaaaatccagatatagatatctrt

caatacrtggatgatttgtatgtaggatctgacttagagatagggcaacatagaacaaaa

atagaggaactgagacaacatttgctgaggygggggttcaccacaccagacaagaaacat

cagaaagaacctccatttctttggatgggatatgaactccatcctgacaaatggacagta

cagcctatacagctgccagaaaaagatagctggactgtcaatgacatacaaaagttagtg

ggaaagctaaattgggcaagtcagatttatcctggaattaaagtaagacaactttgtaaa

ctccttaggggagccaaagcactaacagaaatagtaccactaactgaagaagcagaatta

g-----------------------------------------------

>CRF07BC.14BDZL349

cctcaaatcactctttggcaacgacccatygtcacaataaagataggggggcaattaaag

gaagctctattagatacaggagcagatgatacagtattagaagacatgaatttgccaggg

aaatggaaaccaaaratgatagggggaattggaggttttatcaaagtaagacagtatgaa

cagatacccatagacatctgtggacacacagctataggtacagtattagtgggacctaca

cctgtcaatataattggaaggaatctgttgactcagcttgggtgcactttaaattttcca

atcagtcccattgaaactgtaccagtaaaattaaagccaggaatggatggcccaaaggtt

aaacaatggccattgacagaagagaaaataaaagcattaacagcaatttgtgaggaaatg

garaaggaaggaaaaattacaaaaattgggcctgaaaatccatataacactccaatattt

gccataaaaaagaaggacagtactaaatggagaaaactagtagatttcagggagctcaat

aaaagaactcaagatttctgggaagttcaattaggaataccacacccagcagggttaaaa

aagagaaaatcagtgacagtaytggatgtgggggatgcatatttttcagttcctttatat

gaagatttcagaaaatatactgcattcaccatacctagtataaacaatgaaacaccaggg

attaggtatcagtacaatgtacttccacagggatggaaaggatcaccagcaatattccaa

agtagcatgacaaaaatcttagagccttttagaaaacaaaatccagacatagwtatctat

caatacgtggatgatttgtatgtaggatctgacttagagatagggcagcatagaacaaaa

atagaggaactgagamaacatttgttgasgtggggrtttaccacaccagacaagaaacat

cagaaagaacctccatttctttggatggggtatgarctccatcctgacaartggacagta

cagcctataaagctaccagaaaaggatagctggactgtcaatgatatacaaaagytagtg

ggaaaattaaactgggcaagtcagatttatcctggaattaaagtaaggcaactttgyaaa

ctccttaggggggccaaagcactaacagacatagtaccactaactgaagaagcagaatta

g-----------------------------------------------

>CRF07BC.14BDZL350

cctcaaatcactctttggcaacgacccatcgtcacartaaagataggggggcaattaaag

gaagctctattagatacaggagcagatgatacagtattagaagacatgaatttgccaggg

aaatggaaaccaaaratgatagggggaattggaggttttatcaaagtaagacagtatgaa

cagatacccatagacatctgtggacacacmgctataggtacagtattagtgggacctaca

cctgtcaatataattggaaggaatctgttgactcagmttgggtgcactttaaattttcca

atcagtcccattgaaactgtaccagtaaaattaaagccaggaatggatggcccaaaggtt

aaacaatggccattgacagaagagaaaataaaagcattaacagcaatttgtgaggaaatg

gagaaggaaggaaaaattacaaaaattgggcctgaaaatccatataacactccaatattt

gccataaaaaagaaggacagtaccaaatggagaaaactagtagatttcagggagctcaat

aaaagaactcaagatttctgggaagttcaattaggaataccacacccagcagggttaaaa

aagagaaaatcagtgacagtactggatgtgggggatgcatatttttcagttcctttatat

gaagatttcaggaaatatactgcattcaccatacctagtgtaaacaatgaaacaccaggg

attaggtatcagtacaatgtacttccacagggatggaaaggatcaccagcaatattccaa

agtagcatgacaaaaatcttagagccttttagaaaacaaaatccagacatagttatctat

caatacatggatgatttgtatgtaggatctgacttagagatagggcagcatagaacaaaa

atagaggaactgagacaacatttgttgaggtggggatttaccacaccagacaagaaacat

cagaaagaacctccatttctttggatggggtatgagctccatcctgacaagtggacagta

cagcctataaagctaccagaaaaggatarctggactgtcaatgatatacaaaagttagtg

ggaaaattaaactgggcaagycagatttatcctggaattaaagtaaggcaactttgtaaa

ctccttaggggggccaaagcactaacagacatagtaccactaactgaagaagcagaatta

------------------------------------------------

>CRF07BC.14BDZL400

cctcaaatcactctttggcaacgaccccttgttaccataaagataggggggcaattaaag

gaagctctattagatacaggagcagatgatacagtattagaagacatggatttgccaggg

aaatggaaaccaaaaatgatagggggaattggaggttttatcaaagtaagacagtatgaa

gagatacccgtagaaatttgtggacataaagctataggtacagtattagtaggacctaca

cctgtcaacataattggaagaaatctgttgactcagattggttgtactttaaattttcca

atcagtcctattgaaactataccagtaaaactaaagccaggaatggatggcccaaaggtt

aaacaatggccattgacaaaagagaaaatagaagcattaacagcaatttgtgatgaaatg

gaaaaggaaggaaaaattacaaaaattgggcctgaaaatccatacaacactccaatattt

gccataaaaaagaaagacagtactaagtggagaaaattagtagatttcagggaactcaat

aaaagaactcaagatttttgggaagttcaattaggaataccacacccagcaggattaaaa

aagaaaaaatcagtgacagtgctggatgtgggggatgcatatttttcagttcctttacat

gaagacttcaggaaatatactgcattcaccatacctagtataaacaatgaaacaccaggg

attaggtatcagtacaatgtacttccacagggatggaaaggatcaccagcaatatttcaa

agtagcatgacaagaatcttagagccttttagaaaacaaaatccagacatagtcatatat

caatacatggatgatttgtatgtaggatctgatttagagatagggcagcatagaacaaaa

atagaggaactgagagaacatttgttaaggtggggatttaccacaccagacaagaaacat

cagaaagaacctccatttctttggatggggtatgaactccatcctgacaaatggacagta

cagcctatacagctgccagaacaagacagctggactgtcaatgatatacaaaagttagtg

ggaaaattaaactgggcaagtcagatttatcctggaattaaagtaaggcaactttgtaaa

ctccttaggggggccaaagcactaacagacatagtaccactaacagaagaagcagaa---

------------------------------------------------

>CRF07BC.14BDZL440

cctcaaatcactctttggcaacgacccctagtcaccataaagataggggggcaattaaag

gaagctctattagatacaggagcagatgatacagtattagaagacataaatttgccaggg

aaatggaaaccaaaaatgatagggggaattggaggttttatcaaagtaagacaatatgaa

caggtacccatagaaatttgcggacacaaagcaataggtacagtaytaataggacctaca

cctgtcaacataattggaagaaatctgttgactcagcttggttgcactttaaattttcca

atcagycccattgaaactgtaccagtaaaattaaagccaggaatggatggcccaaaggtt

aaacaatggccmttgacrgaagagaaaataaaagcattaacagcaatttgtgatgaaatg

gagaaggaaggaaaaattacaaaaattgggcctgaaaatccatataacactccaatattt

gccataaaaaagaaggacagtactaagtggagaaaactagtagatttcagggaactcaat

aaaagaactcaagatttttgggaggttcaattaggaataccacacccagcagggttaaaa

aagaaaaaatcagtgacagtactggatgtgggggatgcatatttttcagttcctttatat

gaagacttcaggaaatatactgcattcaccatacctagtacaaacaatgaaacaccaggg

attaggtatcagtayaatgtacttccacagggatggaaaggatcaccagcratattccaa

agtagcatgacaaaaattttagagccttttagaaaacaaaatccagacatagttatctat

caatatatggatgatttgtatgtaggatctgacttagagatagggcaacatagaacaaaa

atagaggaactgagacaacatttgttgaggtggggatttaccacaccagacaagaaacat

caraaagaacctccatttctttggatggggtatgaactccatcctgacaaatggacagta

cagcctatacagctgccagaaaaagatagctggactgtcaatgatatacaaaagttagtg

ggaaaattaaactgggcaagtcagatttatcctggaattaaagtaaggcaactttgtaaa

ctccttaggggggtcaaagcactaacagacatagtacccctractgaggaagcagaatta

------------------------------------------------

>CRF07BC.14BDZL889

cctcaaatcactctttggcaacgaccccttgtcccaataargataggggggcaattaaag

gaagctctattagatacaggagcagatgatacagtattagaagawatgaatttgccaggg

aaatggaaaccaaaaatgatagggggaattggaggttttatcaaagtaagacagtatgaa

cagatacccatagaaatctgtggacataaagctataggtacagtattaataggacctaca

cctgtcaacataattggaagaaatctgttgactcagcttggttgyactttaaattttcca

atcagtcctattgaaactgtaccagtaaaactaaagccaggaatggatggcccaaaggtt

aaacaatggccattgacaaaagagaaaatagaagcattaacagcaatttgtgatgaaatg

gaaaaggaaggaaaaattacaaaaattgggcctgaaaatccatacaacactccaatattc

gccataaaaaagaaagacagtactaagtggagaaaattagtagatttcagggaactcaat

aaaagaactcaagatttttgggaagttcaattaggaataccacacccagcaggattaaaa

aagaaaaaatcagtracagtgytggatgtgggggatgcatatttttcagtycctttagat

gaagacttcaggaaatatactgcattcaccatacctagtgtraacaatgaaacaccaggg

attaggtatcagtacaatgtacttccacagggatggaaaggatcaccagcaatatttcaa

agtagcatgacaaaaatcttagagccttttagaaarcraaatccagacatagtcatctat

caatacatggatgatttgtatgtaggatctgacttagagatagggcaacatagagcaaaa

atagaggaactgagacaacatttgttgargtggggatttaccacaccagacaagaaacat

cagaaagaacctccatttctttggatgggatatgaactccatcctgacaaatggacagta

cagcctatacagctgccagtacaagatagctggactgtcaatgatatacaaaagttagtg

ggaaaattaaactgggcaagtcagatttatcctggaattaaagtaaggcaactttgtaaa

ctccttagggggaccaaagcactaacagaaatagtaccactaactgaagaagcagaatta

gaaattggcag-------------------------------------

>CRF07BC.14ZS134

cctcaaatcactctttggcaacgacccattgtcacaataaagataggagggcaattaaag

gaagctctattagatacaggagcagatgatacagtattagaagacatgaatttgycaggg

aaatggaaaccaaaaatgatagggggaattggaggttttatcaaagtaagacagtatgaa

cagatacccatagaaatctgcggacacaaagctataggtacagtattagtaggacctaca

cctgtcaacataattggaagaaatctgttgactcagcttggttgcactttaaattttcca

atcagccacattgaaactgtaccagtaaaattaaagccagggatggatggcccaagggtt

aaacaatggccattgacagaagagaaaataaaagcattaacagcaatttgtgatgaaatg

gagaaggaaggaaaaattacaaaaattgggcctgaaaatccatataacactccaatattt

gccataaaaaagaaggacagtactaagtggagaaaattagtagatttcagggaactcaat

aaaagaactcaagatttttgggaagttcaattaggaataccacatccagcagggttaaaa

aagaaaaaatcagtgacagtactggatgtgggggatgcatatttttcagtacctttagat

gaagacttcaggaaatatactgcattcaccatacctagtacaaacaatgaaacaccaggg

attaggtatcagtacaatgtacttccacagggatggaaaggatcgccagcaatattccaa

agtagcatgataaaaatcttagagccttttagaaaacaaaatccagacatagttatctat

caatacatggatgatttgtatgtaggatctgatttagagatagggcagcatagaacaaag

gtagaggaactgagacaacatttgttgaggtggggacttaccacaccagacaagaaacat

cagaaagaacctccatttctttggatggggtatgagctccatcctgacaaatggacagta

cagcctatacagctgccagaaaaagatagctggactgtcaatgatatacaaaagttagtg

ggaaaattaaactgggcaagtcagatttatcctggaattaaagtaaggcaactttgtaaa

ctccttagaggggccaaagctctaacagacatagtaccactaaccgaa------------

------------------------------------------------

>CRF07BC.14ZS201

cctcaratcactctttggcaacgacccctcgtcacaataaagataggggggcaaataaag

gaagctctattagatacaggagcagatgatacagtattagaagacatgaatttgccaggg

aaatggaaaccaaaaatgatagggggaattggaggttttatcaaagtaagacagtatgaa

cagatacccatagaaatctgtggacacaaagctataggtacagtattaataggacctaca

cctgtcaacataattggacgaaatctgttgactcagcttggttgcactttaaattttcca

atcagtcctattgaaactgtaccagtaaarttaaagccaggaatggatggcccaagggtt

aaacagtggccattracagaagagaaaataaaagcattaacagcaatttgtgatgaaatg

gagaaggaaggaaaaattacaaaaattgggcctgaaaayccatataacactccaatmttt

gccataaaaaagaaggatagtactaagtggagaaaattagtagatttcagggaactcaat

aaaagaactcaagatttttgggaagttcaattaggaataccacacccagcagggttaaaa

aagaaaaaatcagtgacagtactggatgtgggrgatgcatatttttcagttcctttatat

gaagacttcaggaaatatactgcattcaccatacctagtataaataatgaaacaccaggg

attagrtatcagtacaatgtrcttccacagggatggaaaggatcaccagcaatatttcaa

agtagcatgataaaaatcttagagccttttagaaaacaaaatccagaaatagttatctay

caatacatggatgatttgtatgtaggatctgacttagagatagggcrgcatagaacaaaa

atagaggaactgagacaacatttgttgagrtggggatttaccacaccagacaagaaacat

cagaaagaacctccattcctttggatggggtatgaactccatcctgacaaatggacagta

cagcctatacagctaccagaaaaagatagctggactgtcaatgatatacaaaagttagtg

ggaaaattaaactgggcaagtcagatwtatcctggaattaargtgaggcaactttgtaaa

ctccttaggggggtcaaagcactaacagacatagtaccactaactgaagaagcag-----

------------------------------------------------

>CRF07BC.15ZS011

cctcaaatcactctttggcaacgaccccttgttaccataaagataggggggcaattaaaa

gaagctctattagatacaggagcagatgatacagtattagaagacatgaatttgccaggg

aaatggaaaccaaaaatgatagggggaattggaggttttatcaaagtaagacagtatgaa

cagatacccatagaaatctgtggacataaagctataggtacagtattagtaggacctaca

cctgtcaacataattggaagaaatctgttgactcagcttggttgtactttaaattttcca

atcagtcctattgaaactgtaccagtaaaactaaagccaggaatggatggcccaaaggtt

aaacaatggccattgacaaaagagaaaatagaagcattaacagcaatttgtgatgaaatg

gaaaaagaaggaaaaattacaaaaattgggcctgaaaacccatacaacactccaatattt

gccataaaaaagaaagacagtactaagtggagaaaattagtagatttcagggaactcaat

aaaagaactcaagatttttgggaagtccaattaggaataccacacccagcaggattaaga

aagaaaaaatcagtgacagtgctagatgtgggggatgcatatttctcagttcctttatat

gaagacttcaggaaatatactgcattcaccatacctagtataaacaatgaaacaccaggg

attaggtatcagtacaatgtacttccacaaggatggaaaggatcaccagcaatatttcaa

agtagcatgacaaaaatcttagagccttttagaaaacaaaatccaaaaatagtcatctat

caatacatggatgatttgtatgtaggatctgacttagagatagggcagcatagaacaaaa

atagaggaactgaggcagcatttgttgaggtggggatttaccacaccagacaagaaacat

cagaaagaacctccatttctttggatggggtatgaactccatcctgacaaatggacagta

cagcctatacagctgccagtacaagatagctggactgtcaatgatatacaaaagttagtg

ggaaaattaaactgggcaagtcaaatttatcctggaattaaagtaaggcaactttgtaaa

ctccttaggggggccaaagcactaacagacatagtaccactaactgaagaa---------

------------------------------------------------

>CRF07BC.15ZS012

cctcaaatcactctttggcaacgaccccttgtcacaataaagataggggggcaattaaag

gaagctctattagatacaggagcagatgatacagtattagaagaaatgaatttgccaggg

aaatggaaaccaaaaatgatagggggaattggaggttttatcaaagtaagacagtatgaa

caggtacccatagaaatctgtggacacaaagctgtaggtacagttttaataggacccaca

cctgtcaacataattggragaaatctgttgactcagcttggttgcactytaaattttcca

atcagtcctattgaaactgtaccagtaaaattaaagccaggaatggatggcccaaaggtt

aaacaatggccattgacagaagagaaaataaaagcattaacagcaatttgtgatgaaatg

gagaaggaaggaaaaattacaaaaattgggcctgaaaatccatataacactccaatattt

gccataaaaaagaaggacagtactaartggagaaaattagtagatttcagggaactcaat

aaaagaactcaagatttttgggaagttcagttaggaataccacacccagcagggttaaaa

aggaaaaaatcagtracagtaytggatgtgggggatgcatatttttcagttcctttatat

gaagacttcagaaaatatactgcattcaccatacctagtataaacaatgaaacaccaggg

attaggtatcagtacaatgtgcttccacagggatggaaaggatcaccagcaatattccaa

agtagcatgacaaaaattttagagccttttagaagaaaaaatccagacatagttatttat

caatacatggatgatttgtatgtaggatctgacttagagatagggcagcatagaacaaaa

atagaggaactgagacaacatttgttgaggtggggatttaccaccccagacaagaaacat

cagaaagaacctccatttctttggatgggatatgaactccatcctgacaaatggacagta

cagcctatacagttgccagaaaargatagctggactgtcaatgatatacaaaagttagtg

ggaaaattaaactgggcaagtcagatttatcctggaattaargtaaggcaactttgtaaa

ctccttagggggaccaaagcactaacagacatagtaccactaactgaagaagcagaa---

------------------------------------------------

>CRF07BC.15ZS3020

cctcaaatcactctttggcarcgacccctcgtcacagtaaagataggggggcaattaaag

gaagctctattagatacaggagcagatgatacagtattagaagaaatgaatttgcccggg

aaatggaaaccaaaaatgatagggggaattggaggttttatcaaagtaagacagtatgaa

cagatacccatagaaatctgyggacacaaagctataggtacagtattaataggacctaca

cctgtcaacataattggaagaaayctgttgactcagcttggttgcactttaaattttcca

atcagtcccattgaaactgtaccagtaaaattaaagccaggaatggatggcccaarggtt

aaacaatggccmttgacagaagaraaaataaaagcattaacagaaatttgtgatgaaatg

gagaaggaaggaaaaattacaaaaattgggcctgaaaatccatataacactcctatattt

gccataaaaaagaaggacagtactaagtggagaaaattagtagatttyagggaactcaat

aaaagaactcaagatttttgggaagttcaattaggaataccacacccagcagggttaaaa

argaaaaaatcagtgacgrtactggatgtgggggatgcatatttttcagttcctttagat

aaagactttaggaaatatactgcattcaccatacctagtacaaacaatgaaacaccaggg

attagatatcaatataatgtacttccacagggatggaaaggatcaccagcaatattccaa

agtagcatgacaaaaatcttagagccttttagaaaacaaaacccagacatagttatytat

caatacatggatgatttgtatgtaggatctgacttagagatagggcagcatagaacaaag

atagaggaactgagacaacatttgttgargtggggatttaccacaccagacaagaaacat

cagaaagaacctccatttctttggatggggtatgaactccatcctgacaaatggacagta

cagcctatacagctgccagaaaaggatagctggactgtcaatgatatacaaaagttagtg

ggaaaattaaactgggcaagtcagatttatcctggaattaagataaggcaactttgtaaa

ctccttargggggccaaagcactaacagacatagtaccactaactg--------------

------------------------------------------------

>CRF07BC.16-12ZJ0669

cctcaaatcactctttggcaacgaccccttgtcacaataaaaataggaggacagctaaaa

gaagctctattagatacaggagcagatgatacagtattagaagatataaatttgccagga

aaatggaaaccaaaaatgatagggggaattggaggttttatcaaagtaagacagtatgaa

cagatayccgtagaaatctgtggacataaagctataggtacagtattagtaggacctaca

cctgtcaacataattggaagaaatctgttgactcagcttggttgtactttaaattttcca

atcagtcctattggaactgtaccagtaaaactaaagccaggaatggatggcccaaaagtt

aaacaatggccattgacaaaagagaaaatagaagcattaacagcaatttgtaatgaaatg

gaaaaggaaggaaaaattacaaaaattgggcctgaaaacccatacaacactccaatattt

gccataaaaaagaaagacagtactaagtggagaaaattagtagattttagggaactcaat

aaaagaactcaagatttttgggaagttcaattaggaataccacacccagcaggattaaaa

agaaaaagatcagtgacagtgctggatgtgggggatgcatatttttcagttcctttatat

gaagacttcaggaaatatactgcattcaccatacctagtataaacaatgaagcaccaggg

attaggtatcagtacaatgtacttccacagggatggaaaggatcaccagcaatatttcaa

agtagcatgacaaaaatcttagagccttttagacagcaaaatccagacatagtaatctat

caatacatggatgatttgtatgtaggatctgacttagagatagggcagcatagaacgaaa

atagaggaactgagacaacatttgttgaagtggggatttaccacaccagacaagaaacat

cagaaagaacctccatttctttggatggggtatgaactccatcctgacaaatggacagta

cagcctatacagctgccagtacaagatagctggactgtcaatgatatacagaaattagtg

ggaaaactaaattgggcaagtcaaatttatccagggattaaggtaaagcaactgtgtaga

ctcctcaggggagctaaagcactaacagacatagtac-----------------------

------------------------------------------------

>CRF07BC.16-HuZ12140

cctcaaatcactctttggcaacgaccccttgttaccataaagataggggggcaattaaag

gaagctctattagatacaggagcagatgatacagtattagaagacatgaatttgccaggg

aaatggaaaccaaaaatgatagggggaattggaggttttatcaaagtaagacagtatgaa

cagatacccatagaaatctgtggacataaagctataggtacagtattagtaggacctaca

cctgtcaacataattggaagaaatctgttgactcagcttggttgtactttaaattttcca

atcagtcctattgaaactgtaccagtaaaactaaagccaggaatggatggcccaaaggtt

aaacaatggccattgacaaaagaaaaaatagaagcattaacagcaatttgtgatgaaatg

gaaaaggaaggaaaaattacaaaaattgggcctgaaaatccatacaacactccaatattt

gccataaaaaagaaagacagtactaagtggagaaaattagtagatttcagggaactcaat

aaaagaactcaagatttttgggaagttcaattaggaataccacacccagcaggattaaaa

aagaaaaaatcagtgacagtgctggatgtgggggatgcatatttttcagttcctttacat

gaagacttcaggaaatatactgcattcaccatacctagtataaacaatgaaacaccaggg

attaggtatcagtacaatgtacttccacagggatggaaaggatcaccagcaatatttcaa

agtagcatgacaaaaatcttagagccttttagaaaacaaaatccagacatagtcatctat

caatacatggatgatttgtatgtaggatctgacttagagatagggcagcatagaacaaaa

atagaggaactgagacaacatttgttgaggtggggatttaccacaccagacaagaaacat

cagaaagaacctccatttctttggatggggtatgaactccatcctgacaaatggacagta

cagccyatacagctgccagtacaagatagctggacggtcaatgatatacaaaagttagtg

ggaaaattaaactgggcaagtcagatttatcctggaattaaagtaaggcaactttgtaaa

ctccttaggggggccaaagcactaacagacatagtaccactaactgaagaagcagaa---

------------------------------------------------

>CRF07BC.16-LC006

cctcaaatcactctttggcaacgacccctcgtcacagtaaagataggggggcaattaaag

gaagctctattagatacaggagcagatgatacagtattagaagacatgaatttgcccggg

aaatggaaaccaaaaatgatagggggaattggaggttttatcaaagtaagacagtatgaa

cagatacccatagaaatctgcggacacaaagctataggtacagtattagtgggacctaca

cctgtcaacataattggaagaaatctgttgactcaaattggttgcactttaaattttcca

atcagtcccattgadactgtaccagtaaaattaaagccaggaatggatggcccaaaggtt

aaacaatggccattgacagaagagaaaataaaagcattaacggaaatttgtaatgaaatg

gagaaggaaggaaaaattacaaaaattgggcctgaaaatccatataacactccaatattt

gccataaaaaagaaggacagtactaagtggagaaagttagtggatttcagagaactcaat

aaaagaactcaggatttttgggaagttcaattaggaataccacacccagcagggttaaaa

aagaaaaaatcagtgacagtgctagatgtgggggatgcatatttttcagttcctttacat

gaagacttcaggaaatatactgcattcaccatacctagtataaacaatgaaacaccaggg

attaggtatcagtacaatgttcttccacagggatggaaaggatcaccagcaatattccaa

agtagcatgacaagaatcttagagccttwtagaaaacaaaatccagacatagttatctat

caatacatggatgatttgtatgtaggatctgacttagagatagggcagcatagaacaaaa

atagaggaactgagacaacatttgttgaagtggggatttaccacaccagacaaaaaacat

caaaargaacctccattcctttggatggggtatgaactccatcctgacaaatggacagta

cagcctatacagctaccagagaaagatagctggactgtcaatgatattcaaaagttagtg

ggaaaattaaactgggcaagtcagatttatcctggaatcaaaataaggcaactttgtaaa

ctccttaggggaactaaagcactaacagaagtagtaccactaactgaagaagcagaatta

g-----------------------------------------------

>CRF07BC.16-SX2012475

cctcaaatcactctttggcaacgacccctcgtcccaataaggataggggggcaattaaag

gaagctctattagatacaggagcagatgatacagtattagaagaaataaatttaccaggg

aaatggaaaccaaaaatgatagggggaattggagggtttatcaaagtaagacagtatgaa

gagatacctgtagaaatctgcggacacaaagctataggtacagtattagtaggacctaca

cctgtcaacataattggcagaaatctgttgactcagcttggttgcactttaaattttcca

atcagtcctattgaaactgtaccagtaaaattaaagtcaggaatggatggcccaaaggtt

aaacaatggccattgacagaagagaaaataaaagcattaacagcaatttgtaaggaaatg

gagaaggaaggaaaaatcacaaaaattgggcctgaaaatccatataacactccaatattt

gccataaaaaagaaggacagtactaaatggagaaaattagtagattttagggaactcaat

aaaagaactcaagatttttgggaagttcaattaggaataccacacccagcagggttaaga

aagaaaaagtcagtgacagtactagatgtgggggatgcatatttttcagttcctttatat

gaagacttcaggaagtatactgcattcaccatacctagtatgaacaacgaaacaccaggg

attaggtatcagtacaacgtactcccacagggatggaaaggatcaccagcaatattccaa

agtagcatgacaagaatcttagagccgtttagaaaacaaaatccagacatagttatctat

caatacatggatgatttgtatgtaggatctgacttagagatagggcagcatagaacaaaa

atagaggaactgagacaacatttgttgaggtggggatttaccacaccagacaagaaacat

cagaaagaacctccatttctttggatggggtatgaactccatcctgacaaatggacagta

cagcctatacagctgccagacaaagatagctggactgtcaatgatatacaaaagttagtg

ggaaaattaaactgggcaagtcagatatatcctggaattaaagtaagacaactttgtaaa

ctccttaggggggctaaagcactaacagacatagtaccactaactgaagaagcagaa---

------------------------------------------------

>CRF07BC.16BDZL0304

cctcaaatcactctttggcaacgaccccttgttaccataaagatagggggacaagtcaag

gaagctctattagatacaggagcagatgatacagtattagaagacatgaatttgccaggg

aaatggaaaccaaaaatgatagggggaattggaggttttatcaaagtaagacagtatgaa

cagatatccatagacatctgtggacataaagtcataggtacagtattagtaggacctaca

cctgtcaacataattggaagaaatctgttgactcagcttggttgtaccttaaattttcca

atcagtcctattgaaactgtaccagtaaaattaaagccaggaatggatgacccaaaggtt

aaacaatggccattgacaaaagagaaaatagaagcattaacagcaatttgtgatgaaatg

gaaaaggaaggaaaaattacaaaaattgggcctgaaaatccatataacactccaatattt

gccataaaaaagaaagacagtactaagtggagaaaattagtagatttcagggaactcaat

aaaagaactcaagatttttgggaagttcaattaggaataccacacccagcaggattaaaa

cagaaaaaatcagtgacagtactagatgtgggggatgcatatttctcagttcctttatat

gaagacttcaggaagtatactgcattcaccatacctagtataaacaatgaaacaccaggg

attaggtatcagtacaatgtactcccacagggatggaaaggatcaccagcaatatttcaa

gctagcatgacaaaaatcttagagccttttagaaaacaaaatccagacatagtcatctat

caatatatggatgatttgtatgtaggatctgacttagagatagggcagcatagaacaaaa

atagaggaactgagacaacatctgttgaagtgggggtttaccacaccagacaagaaacat

cagaaagaacctccatttctttggatggggtatgaactccatcctgacaaatggacagta

cagcccatacagctgccagtacaagatagctggactgtcaatgatatacaaaagttagtg

ggaaaattaaactgggcaagtcagatttatcctggaattaaagtaagacaactttgtaaa

cttcttaggggggccaaagcactaacagacatagtaccactaactgaagaagcagaat--

------------------------------------------------

>CRF07BC.16BDZL0917

cctcaaatcactctttggcaacgaccccttgttaccataaaggtaggggggcaattaaag

gaagctctattagatacaggagcagatgatacagtattagaagacataaatttgccaggg

aaatggaaaccaaaaatgatagggggaattggaggttttatcaaagtaagacagtatgaa

cagatacctatagaaatctgtggacataaagctataggtacagtattagtaggacctaca

cctgtcaacataattggaaggaatctgttgactcagattggttgtactttaaattttcca

atcagtcctattaaaactgtaccagtaaaactaaagccaggaatggatggcccaaaggtt

aaacaatggccattgacaaaagagaaaatagaagcattaacagcaatttgtgaggaaatg

gaaaaggaaggaaaaattacaaaaattgggcctgaaaatccgtacaacactccaatattt

gccataaaaaagaaagacagtactaagtggagaaaattagtagatttcagggaactcaat

aaaagaactcaagatttttgggaagttcaattaggaataccacacccagcaggattaaga

aagaaaaaagcagtgacggtgctggatgtaggggatgcatatttttcagttcctttatat

gaagacttcaggaaatatactgcattcaccatacctagtacaaacaatgagacgccagga

atcagatatcagtacaatgtgctaccacagggatggaaaggatctccggcaatattccag

tgtagcatgacaaaaatcttagagccctttagaagaaaaaatccagagatggttatctat

caatatatggatgacttgtatgtaggatctgatttagaaatagggcaacacagaacaaaa

atagaggagctaagagctcatctattaagctggggatttactacaccagacaaaaagcat

cagaaggaacctccatttctttggatgggatatgaactccatccggacagatggacagtc

caacctatagaactgccagcaaaagacagctggactgtcaatgatatacagaaattagtg

ggaaaactaaattgggcaagtcaaatttatgcaggaattaaggtaaagcaattgtgtaga

ctcctcaggggagctaaagcactaacagacatagtaccactgactgaagaagcagaatta

gaattggc----------------------------------------

>CRF07BC.16BDZL1028

cctcaaatcactctttggcarcgaccccttgttacaataaagataggagggcaattaaag

gaagctytattagatacaggagcagatgatacagtattagaagacatgaatttgccaggg

aaatggaaaccaaaaatgatagggggaattggaggttttatcaaagtaagacagtatgaa

cagrtacccatagaaatctgtggacataaagctataggtacagtattartaggrcctaca

cctgtcaacataattggaagaaatctgttractcagcttggttgtactttaaattttcca

atcagycctattgaaactgtaccagtaaaaytaaagccaggaatggatggcccaaaagtt

aaacaatggccattaacaaaagagaaaatagaagcrttaacagcaatttgtgaagaaatg

gaaaaggaaggaaaaattacaaaaattgggcctgaaaatccatacaacactccaatattt

gccataaaaaagaaagacagtactaagtggagaaaattagtagatttcagggaactcaat

aaaagaactcaagatttttgggaagttcaattaggaataccacacccagcaggattaaaa

aagaaaaaatcagtgacagtgctggatgtgggggatgcatatttttcagttcctttatat

gaagacttcaggaaatatactgcattcaccatacctagtataaacaatgaaacrccaggg

attaggtatcagtacaatgtactkccacagggatggaagggatcaccagcaatatttcaa

agtagcatgacaaaaatcttagagccttttagaaaacaaaatccagacatagtcatctat

caatacatggatgatttgtatgtaggatctgacttagagataggrcarcatagaacaaaa

atagaggaactgagacaacatttgttgaggtggggatttaccacaccagacaagaaacat

cagaaagaacctccatttctttggatggggtatgaactccatcctgacaaatggacagta

cagcctattcagctgccagtacaagatagctggactgtcaatgatatacaaaagttagtg

ggaaaattaaactgggcaagtcagatttatcctggaattaaagtaaggcaactttgtaaa

ctccttaggggggccaaagcactaacagacatagtaccactaactgaagaagcagaatta

gaattggcaga-------------------------------------

>CRF07BC.16BDZL1100

cctcaaatcactctttggcaacgacccattgtcacaataaagrtaggggggcarttaaag

gaagctctattagatacaggagcagatgayacagtattagaagacatraatttgkcaggg

aaatggaaaccaaaaatgatagggggaattggaggttttatcaaagtaagacagtatgaa

saratacctgtagaaatctgtggacacaaagctataggtacagtattagtrggacctaca

cctgtcaacataattggaagaaatctgttgactcarcttggttgcactttaaattttcca

atcagtcccattgawactrtaccagtaaaattaaagccaggaatggatggcccaaaggtt

aaacaatggccattgacagaagagaaaataaaagcattaacagaaatttgtratgaaatg

garaargaaggaaaaattacaaaaattgggcctgaaaatccatataacactccaatattt

gccataaaaaagaaggacagtactaagtggagaaarktagtagatttcagggaactcaat

aaaagaacacaagatttytgggaggttcaattaggaataccacacccagcaggrttaaaa

argaaaaaatcagtgwcagtactagatrtrggggatgcatatttttcagttcctttacat

gaagayttcaggaaatatactgcattcaccatacctagtataaacaatgaaacaccaggg

attaggtatcagtacaatgtacttccacarggatggaaaggatcaccagcaatatttcaa

agtagtatgacaagaatcttrgagccttttagaaarcaaaatccagacatarwtatctrt

caatacatggatgatttgtatgtaggatctgacttagagatagggcagcayagaacaaaa

rtagaggaactgagacaacatttgttgaggtggggattyaccacaccagataagaaacat

cagaaagaaccwccatttctttggatggggtatgaactccatcctgacaaatggacagta

cagcctatacagctgccagaaaaagatagctggactgtcaatgatatacaaaagttagtg

ggaaaattaaattgggcaagtcagatttaccctggaattaaagtaaggcaactttgtaaa

ctccttaggggggccaaagcactaacagamatagtaccactaactgaagaagcagaa---

------------------------------------------------

>CRF07BC.17-LC002

cctcagatcactctttggcaacgaccccttgtcacaataaagataggggggcaattaarg

gaagctctattagatacaggagcagatgatacagtattagaagacataaatttgccaggg

aaatggaaaccaaaaatgatagggggaattgggggttttatcaaagtaagacaatatgaa

cagatacccatagaaatctgtggacatcaagctataggtacagtattagtaggacctaca

cctgtcaacataattggaagaaatctgttgactcagcttggttgtactttaaattttcca

atcagtcctattgaaactgtaccagtaaaactaaagccaggaatggatggcccaaaggtt

aaacaatggccattgacaaaagagaaaatagaagcattaacagcaatttgtgatgaaatg

gaaaaggaaggaaaaattacaaaaattgggcctgaaaatccatacaacactccaatattt

gccataaaaaagaaagacagtactaagtggagaaaattagtagatttcagggaactcaat

aaaagaactcaagatttttgggaagttcaattaggaataccacacccagcaggattaaaa

aagaaaaaatcagtgacagtgctggacgtgggggatgcatatttttcagttcctttatat

gaagactttaggaaatatactgcattcaccatacctagtataaacaatgaaacaccaggg

attaggtatcagtacaatgtacttccacagggatggaaaggatcaccagcaatatttcaa

agtagcatgacaagaatcttagaaccttttagaaaacaaaatccagacataatcatctat

caatacatggatgatttgtatgtaggatctgacttagagatagggcagcatagaacaaaa

atagaagaactgagacaacatttgttgaggtggggatttaccacaccagacaagaaacat

cagaaagaacctccatttctttggatggggtatgaactccatcctgataaatggacagta

cagcctatacagctgccagtacaagatagctggactgtcaatgatatacaaaagttagtg

ggaaaattaaactgggcaagtcagatttatcctgga------------------------

------------------------------------------------------------

------------------------------------------------

>CRF07BC.18-LC003

cctcaaatcactctttggcaacgaccccttgttaccataaagataggggggcaattaaag

gaagctctattagatacaggagcagatgatacagtattagaagacatgaatttgccaggg

aaatggaaaccaaaaatgatagggggaattggaggttttatcaaagtaagacagtatgaa

cagatacccatagaaatctgtggacataaagctacaggtacagtattagtaggacctaca

cctgtcaacataattggaagaaayctgttgactcaacttggttgtactttaaattttcca

atcagtcctattgaaactgtaccagtaaaaytaaagccaggratggatggcccaaaggtt

aaacaatggccattgacaaaagagaaaatagaagcattaacagcaatttgtgaggaaatg

gaaaaggaaggaaaaattacaaaaattgggcctgaaaatccatacaacactccaatattt

gccataaaaaagaaagacagtactaagtggagaaaattagtagatttcagggaactcaat

aaaagaactcaagatttttgggaagttcaattaggaataccacacccagcaggattaaaa

aagaaaagatcagtgacagtgctggatgtaggggatgcatatttttcagttcctttatat

gaagacttcaggaaatatacygcattcaccatacctagtataaacaatgaaacaccaggg

attaggtatcagtacaatgtacttccacagggatggaaaggatcaccagcaatatttcaa

agtagcatgacaaaaatcttagagccttttagaaaacaaaatccagacatagtcatctat

caatatatggatgatttgtatgtaggatctgatttagagatagggcagcatagaacaaaa

atagaggaactgagacaacatttgttgaggtggggatttaccacaccagacaagaagcat

cagaaagaacctccatttctttggatggggtatgaactccatcctgacaaatggacagta

cagcctatacagctgccagtacaagatagctggactgtcaatgatatacaaaagttagtg

ggaaaattaaactgggcaagtcagatttatcctggaattaaagtaaggcaactttgtaaa

ctccttaggggggccaaagcactaacagacatagtaccactaactgaggaagcagaatta

g-----------------------------------------------

>CRF07BC.19-5101186211

cctcaaatcactctttggcaacgaccccttgtcacaataaagataggggggcaattaaag

gaagctctattagatacaggagcagatgatacagtattagaagacataaatttgccaggg

aaatggaaaccaaaaatgatagggggaattggaggttttatcaaagtaagacagtatgaa

cagrtacccatagaaatctgtggacatcaagttataggcacagtattaataggacctaca

cctgtcaacataattggaagaaatctgttgactcagcttggttgtactttaaattttcca

atcagtcctattgaaactgtaccagtaaaactaaagccaggaatggatggcccaaaggtt

aaacaatggccattgacaaaagagaaaatagaagcattaacagcaatttgtgatgaaatg

gaaaaggaaggaaaaattacaaaaattgggcctgaaaatccatacaacactccaatattt

gccataaaaaagaaggacagtactaagtggagaaaattagtagatttcagggaactcaat

aaaagaactcaagatttttgggaagttcaattaggaataccacacccagcaggattaaaa

cagaaaaaatcagtracagtgctggatgtgggrgatgcatatttttcagttcctttacat

gaagactttaggaaatatactgcattcaccatacctagtataaacaatgaaacaccaggg

ataaggtatcagtacaatgtacttccacagggatggaaaggatcaccagcaatatttcaa

agtagcatgacaaraatcttagaaccytttagraaacagaatccagacatagtcatctat

caatacatggatgatttgtatgtaggatctgacttagagatagggcagcatagaacaaaa

atagaggarctgagacaacatttgttgaggtggggatttaccacaccagacaagaaacat

cagaaagaacctccatttctttggatggggtatgaactccatcctgayaaatggacagta

cagcctatacagctgccagtacaagatagctggactgtcaatgatatacaaaagttagtg

ggaaaattaaaytgggcaagtcagatttatcctggaattaaagtaaggcaactttgtaaa

ctccttagggggaccaaagcactaacagacatagtaccactaactgaagaagcagaatta

gaa---------------------------------------------

>CRF07BC.20-121220LW1

cctcaaatcactctttggcaacgaccccttgtgacaataaarataggggggcaattaaag

gaagctctattagatacaggagcagatgatacagtattagaagacatgaatttgccaggg

aaatggaaaccaaaaatgatagggggaattggaggttttatcaaagtaagacagtatgaa

caaatacccatagaaatctgtggacataaagctataggtacagtattagtaggacctaca

cctgtcaacataattggaagaaatctgttgactcagcttggttgtactttaaattttcca

atcagtcctattgagactgtaccagtaaaactaaagccaggaatggatggcccaaaggtt

aaacaatggccgttgacaaaagagaaaatagaagcattaacagcaatttgtgatgaaatg

garaaggaaggaaaaattacaaaaattgggcctgaaaatccatacaacactccaatattt

gccataaaaaagaaagacagtactaartggagaaaattagtagatttcagagaactcaat

aaaagaactcaagatttttgggaagttcaattaggaataccacacccagcaggattaaaa

aagaaaaaatcagtgacagtgctggatgtgggggatgcatatttttcagttcctttatat

gaagacttcaggaaatatactgcattcaccatacctagtataaacaatgaaacaccaggg

attaggtatcagtacaatgtacttccacagggatggaaaggrtcaccagcaatatttcaa

agtagcatgacaaaaatcttagagccttttagaaaacaaaatccagacatagtcatctat

caatacatggatgatttgtatgtaggatctgacttagagataggacagcatagaacaaaa

atagaggaactgagacaacatttgttgaggtggggatttaccacaccagacaagaaacat

cagaaggaacctccatttctttggatggggtatgaactccatcctgacaaatggacagta

cagcctatacagctgccagtacaagatagctggactgtcaatgatatacaaaagttagtg

ggaaaattaaactgggcaagtcagatttatcctggaattaaagtaaggcaactttgtaaa

ctccttaggggggtcaaagcactaacagacatagtaccactaactgaagaagcagaatt-

------------------------------------------------

>CRF07BC.20-20121299

cctcaaatcactctttggcaacgaccacttgtcacaataaagataggggggcarttaaag

gaagctytattagatacaggagcagatgatacagtattagaagacatgaatttgccaggg

aaatggaaaccaaaaatgatagggggaattggaggttttatcaaagtaaaacagtatgaa

cagatacccatagaaatctgtggacataaagctataggtacagtattaataggacctaca

cctgtcaacataattggaagaaatctgttgactcagcttggttgtactttaaattttcca

atcagycctattgaaactgtaccagtaaaactaaagccaggaatggatggcccaaaggtt

aarcaatggccattgacaaaagagaaaatagaagcattaacagcaatttgtgatgaaatg

gaaaaggaaggaaaaattacaaaaattgggcctgaaaatccatacaacactccaatattt

gccataaaaaagaaagacagtactaagtggagaaaattagtagatttcagggaactcaat

aaaagaactcaagatttttgggaagttcaattaggaataccacacccagcaggattaaaa

aggaaaaaatcagtgacagtgctggatgtgggggatgcatatttttcagttcctttacat

gaagacttcaggaaatatactgcattcaccatacctagtacaaacaatgaaacaccaggg

attagatatcagtacaatgtacttccacagggatggaaaggatcaccagcaatatttcaa

agtagcatgacaaaaatcttagagccttttagaaaacaaaatccagacatagtcatctat

caatacatggatgatttgtatgtaggatctgacttagarataaggcagcatagaacaaaa

atagaggaactaagacaacatttgttgaggtggggatttaccacaccagacaagaaacat

cagaaagaacctccatttctttggatggggtatgaactccatcctgacaaatggacagta

cagcctatacagctgccagtacaagatagctggactgtcaatgatatacaaaagttagtg

ggaaaattaaactgggcaagtcagatttatyctggarttaaartaaggcaactttgtaaa

ctccttagggggaccaaagcactaacagacatagtaccactaactgaagaagcagaat--

------------------------------------------------

>CRF07BC.20130291

cctcaaatcactctttggcaacgaccccttgttacaataaagatagggggacaagtaaag

gaagctctattagatacaggagcagatgatacagtattagaagacatgaatttgccaggg

aaatggaaaccaaaaatgatagggggaattggaggttttatcaaagtaagacagtatgaa

cagatacccatagaaatctgtggacataaagctataggtacagtattagtaggacctaca

cctgtcaatataattggaagaaatctgttaactcagcttggttgtactttaaattttcca

atcagtcctattgaaactgtaccagtaaaattaaagccaggaatggatggcccaaaggtt

aaacaatggccattgacaaaagagaaaatagaagcattaacagcaatttgtgatgaaatg

gaaaaggaaggaaaaattacaaaaattgggcctgaaaatccatacaacactccaatattt

gccataaaaaagaaagacagtactaagtggagaaaattagtagatttcagggaactcaat

aaaagaactcaagatttttgggaagttcaattaggaataccacacccagcaggattaaaa

aagaaaaaatcagtgacagtgctggatgtgggggatgcatatttttcagttcctttagat

gaagactttaggaaatatactgcattcaccatacctagtacaaacaatgagacaccaggg

actaggtatcagtacaatgtacttccacagggatggaaaggatcaccagcaatatttcaa

agtagcatgacaaaaatcttagagccttttagaaaacaaaatccagacatagtcatctat

caatacatggatgatttgtatgtaggatctgacttagagatagggcagcatagaacaaaa

atagaggaactgagacaacatttgttgaggtggggatttaccacaccagacaagaaacat

cagaaagaacctccatttctttggatggggtatgaactccatcctgacaaatggacagta

cagcctatacagctgccagtacaagatagctggactgtcaatgatatacaaaagttagtg

ggaaaattaaactgggcaagtcagatttatcctggaattaaagtaaggcaactttgtaaa

ctccttaggggggccaaagcactaacagacatagtaccactaactgaagaagcagaatt-

------------------------------------------------

>CRF07BC.2013HuZ024

cctcaaatcactctttggcaacgaccccttgtcacaataaggatagggggacaattaaaa

gaagctctattagatacaggagcagatgatacagtattagaagacatggatttgccaggg

aaatggaaaccaaaaatgatagggggaattggaggttttatcaaagtaagacagtatgaa

cagatacccatagaaatctgtggacataaagctataggtacagtgttagtaggacctaca

cctgtcaacataattggaagaaatctgttgactcagattggttgcactttaaatttccca

atcagtcctattgaaactgtaccagtaaaattaaagccaggaatggatggcccaaaggtt

aaacaatggccattgacaaaagagaaaatagaagcattaacagcaatttgtgaggaaatg

gagaaagaaggaaaaattacaaaaattgggcccgaaaatccatacaacactccaatattt

gctataaaaaagaaagacagtactaagtggagaaaattagtagatttcagggaactcaat

aaaagaactcaagacttttgggaagttcaattaggaataccacacccagcaggattaaaa

aagaaaaaatcagtgacagtgctggatgtgggggatgcatatttttcagttcctttatat

gaagacttcaggaaatatactgcattcaccatacctagtatgaacaatgaaacaccaggg

attaggtatcagtacaatgtacttccacagggatggaaaggatcaccagcaatatttcaa

agtagcatgacaaaaatcttggagccttttagaaaacaaaatccaaacatagtcatctat

caatacatggatgatttgtatgtaggatctgacttagagatagggcagcatagaacaaaa

atagaggaactgagacaacatttgttgaggtggggatttaccacaccagacaagaaacac

cagaaagaacctccatttctttggatggggtatgaactccatcctgacaaatggacagta

cagcctatacagctgccagtacaagatagctggactgtcaatgatatacaaaagttagtg

ggaaaattaaactgggcaagtcagatttatcctggaattaaagtaaggcaactttgtaaa

ctccttaggggggccaaagcactaacagacatagtaccactaactgaagaagcagaa---

------------------------------------------------

>CRF07BC.21-5101699010

cctcaaatcactctttggcaacgacccctcgtcacaataaaggtaggggggcaattaaag

gaagctctattagatacaggagcagatgatacagtattagaagaaatgaatttgccaggg

aaatggaaaccaaaaatgatagggggaattggaggttttatcaaagtaagacagtatgaa

cagatacccatagaaatctgcggacacaaagctataggtacagtattagtagggcctaca

cctgtcaacataattggaagaaatctgttgactcagcttggctgtactttaaattttcca

atcagtcccattgaaactgtaccagtaaaattaaagccaggaatggatggcccaaaggtt

aaacaatggccattgacagaagagaaaataaaagcattaacagcaatttgtgaggaaatg

gagaaggaaggaaaaattacaaaaattgggcctgaaaatccatataatactccaatattt

gcgataaaaaagaaggacagtacaaagtggagaaaattagtagatttcagggaactcaat

aaaagaactcaagatttttgggaagttcaattagggataccacacccagcagggctaaaa

aagaaaaaatcagtgacagtactggatgtgggggatgcatatttttcagttcctttatat

gaagatttcaggaaatatacggcattcaccatacctagtataaacaatgaaacaccaggg

attaggtatcagtacaatgtacttccacagggatggaaaggatcaccagcgatattccaa

agtagcatgacaaaaatcttagaaccttttagaaaacagaatccagacatagttatctat

caatacatggatgatttgtatgtaggctctgacttagaaatagggcaacatagaacaaaa

atagaggaactgagacaacatttgttgaggtggggatttaccacaccagataagaaacat

cagaaagaacctccatttctttggatggggtatgaactccatcctgacaaatggacagta

cagcctataaagctaccagaaaaagatagctggactgtcaatgatatacagaagttagtg

ggaaaattaaactgggcaagtcagatttatcctggaattaaagtaaggcaactttgtaaa

ctccttaggggggccaaggcactaacagaaatagtaccactaactgaagaagcagaat--

------------------------------------------------

>CRF07BC.22-20121555

cctcaaatcactctttggcaacgaccccttgtcacaataaagataggggggcaattaaag

gaagctctattagatacaggagcagatgatacagtattagaagacatgaatttgccagga

aaatggaaaccaaaaatgatagggggaattggaggttttatyaaagtaagacagtatgaa

cagatacccatagaaatctgtgggcataaagctataggtacagtattagtgggacctaca

cctgtcaacataattggaagaaatctrttgactcagcttggttgtactttaaatttccca

atcagtcctattgaaactgtaccagtaaaactaaagccaggaatggatggcccaaaggtt

aaacaatggccattgacaaaagagaaaatagaagcattaacagcaatttgcgatgaaatg

gaaaaggaaggaaaaattacaaaaattgggcctgaaaatccatacaacactccaatattt

gccataaaaaagaaagacagtactaagtggagaaaattagtagatttcagggaactcaat

aaaagaactcaagatttttgggaagttcaattaggaataccacacccagcaggattaaaa

aagaaaaaatcagtgacagtgctagatgtgggggatgcatatttttcagttcctttatat

gaagacttcaggaaatatactgcattcaccatacctagtataaacaatgaaacaccaggg

attaggtatcagtacaatgtacttccacagggatggaaaggatcaccagcaatatttcaa

agtagcatgacaaaaatcttagagccttttagaaaacaaaatccagacatagtcatctat

caatatatggatgatttgtatgtaggatctgacttagagatagggcagcatagaacaaaa

atagaggaactgagacaacatttgttgaggtggggatttaccacaccagacaagaaacat

cagaaagaacccccatttctatggatggggtatgaactccatcctgacaaatggacggta

cagcctatacagctgccagtacaggatagctggactgtcaatgatatacaaaagttagtg

ggaaaattaaactgggcaagtcagatttatcctggaattaaagtaaggcaactttgtaaa

ctccttaggggggccaaagcactaacagacatagtaccactaactgaagaagca------

------------------------------------------------

>CRF07BC.22-5101699048

cctcaaatcactctttggcaacgaccccttgtcacagtaaaaatagaaggacagctgaaa

gaagctctattagatacaggagcagatgatacagtattagaagatataaaattgccagga

aaatggamaccaaaaatgatagggggaattggaggttttatcaaagtaagacagtatgaa

cagatacccatagaaatctgtggacataaagctataggtacagtattagtaggacctaca

cctgtcaacataattggaagaaatctgttgactcagcttggttgtactttaaattttcca

atcagtcctattgaaactgtaccagtaaaactaaagccaggaatggatggcccaaaggtt

aaacaatggccattgacaaaagagaaaatagaagcattaacagcaatttgtgatgaaatg

gaaaaggaaggaaaaattacaaaaattgggcctgaaaatccatacaacactccaatattt

gccataaaaaagaaagacagtactaagtggagaaaattagtagatttcagggaactcaat

aaaagaactcaagatttttgggaagttcaattaggaataccacacccagcaggattaaaa

aagaaaaaatcagtgacagtgctggatgtgggggatgcatatttttcagttcctttatat

gaagacttcaggaaatatactgcattcaccatacctagtataaacaatgaaacaccaggg

attaggtatcagtacaatgtacttccacaaggatggaaaggatcaccagcaatatttcaa

agtagcatgacaaaaatcttagagccttttagaaaacaaaatccagacatagtcatctat

caatacatggatgatttgtatgtaggatctgacttagagataggacagcatagaacaaaa

atagaggaactgagacaacatttgttgaggtggggrtttaccacaccagacaagaaacat

cagaaagaacctccatttctttggatggggtatgaactccatcctgacaaatggacagta

cagcctatacagctgccagtacaagatagctggactgtcaatgatatacaaaagttagtg

ggaaaattaaaytgggcaagtcagatttatcctggaattaaagtaaggcaactgtgtaaa

ctccttaggggggtcaaagcactaacagacatagtaccactaactgaagaagcag-----

------------------------------------------------

>CRF07BC.23-20121547

cctcaaatcactctttggcaacgaccccttgttaccataaagataggggggcaattaaaa

gaagctctattagacacaggagcagatgatacagtattagaagacatgaatttgccaggg

aaatggaaaccaaaaatgatagggggaattggaggttttatcaaagtaagacagtatgaa

cagatacccgtagaaatctgtggacataaagctataggtacagtattagtaggacctaca

cctgtcaacataattggaagaaatctgttgactcagcttggttgtactttaaattttcca

atcagtcctattgaaactgtaccagtaaaactaaagccaggaatggatggcccaaaggtt

aaacaatggccattgacaaaagagaaaatagaagcattaacagcaatttgtgatgaaatg

gaaaaggaaggaaaaattacaaaaattgggcctgaaaatccatacaacaccccaatattt

gccataaaaaagaaagacagtactaagtggagaaaattagtagatttcagggaactcaat

aaaagaactcaagatttttgggaagttcaattagggataccacacccagcaggattaaaa

aagaaaaaatcagtgacagtgctggatgtgggggatgcatatttttcagttcctttatat

gaagacttcaggaaatatactgcattcaccatacctagtataaacaatgaaacaccaggg

attaggtatcagtacaatgtacttccacagggatggaaaggatcaccagcaatatttcaa

agtagcatgacaaaaatcttagagccttttagaaaacaaaatccagacatagtcatttat

caatacatggatgatttgtatgtaggatctgacttagagatagggcagcatagaacaaaa

atagaggaactgagacaacatttgttgaggtggggatttaccacaccagacaagaaacat

cagaaagaacctccgtttctttggatggggtatgaactccatcctgacaaatggacagta

cagcctatacagctgccagtacaagatagctggactgtcaatgatatacaaaagttagtg

ggaaaattaaactgggcaagtcagatttatcctggaattaaagtaaggcaactttgtaaa

ctccttaggggggccaaagcactaacagacatagtaccactaactgaagaagcaga----

------------------------------------------------

>CRF07BC.23-SX2012090

cctcaaatcactctttggcaacgaccccttgttccaataaagataggggggcaattaaag

gaagctttattagatacaggagcagacgatacagtattagaagacatgaatttgccagga

aaatggaaaccaaaaatgatagggggaattggaggttttatcaaagtaagacagtatgaa

gagatacccatagaaatctgtggacataaagctataggtacagtattagtaggacctaca

cctatcaacataattggaaggaatctgttgactcagcttggttgtactttaaattttcca

atcagtcctattgatactgtaccagtaaaactaaagccaggaatggatggcccaaaggtt

aaacaatggccattgacaaaagagaaaatagaagcattaacagcaatttgtgatgaaatg

gaaaaggaaggaaaaattacaaaaattgggcctgaaaatccatacaacactccaatattt

gccataaaaaagaaagacagtactaagtggagaaaattagtagatttcagggaactcaat

aaaagaactcaagatttttgggaagttcaattaggaataccacacccagcaggattaaaa

aagaaaaaatcagtgacagtgctggatgtgggagatgcatatttttcagttcctttagat

gaagacttcaggaaatatactgcattcaccatacctagtataaacaatgaaacaccaggg

attaggtaycagtacaatgtacttccacagggatggaaaggatcaccagcaatatttcaa

agtagcatgacaaaaatcttagagccttttagaaracaaaatccagacatagtcatctat

caatacatggatgatttgtatgtaggatctgacttagagataaagcagcatagaacaaaa

atagaggaactgagacaacatttgttgaggtggggatttaccacaccagacaagaaacat

cagaaagaacctccatttctttggatggggtatgaactccaccctgacaaatggacagta

cagcctatacagctgccagtacaagatagctggacggtcaatgatatacaaaagttagtg

ggaaaattaaactgggcaagtcagatttatcctggaattaaaacaaggcaactttgtaaa

ctccttagggggaccaaagcactaacagacatagtaccactaactgaagaagcagaatta

g-----------------------------------------------

>CRF07BC.24-7340

cctcaaatcactctttggcaacgaccccttgtcacaataaagataggggggcaattaaag

gaagctctattagatacaggagcagatgatacagtattagaagacatggatttgccaggg

agatggaaaccaaaaatgatagggggaattgggggttttatcaaagtaagacagtatgaa

gagatacccatagaaatctgtggacataaagctataggtacagtattagtaggacctaca

cctgtcaacataattggaagaaatctgttgactcagcttggttgtactttaaattttcca

atcagtcctattgaaactgtaccagtaaaactaaagccaggaatggatggcccaaaggtt

aaacaatggccattgacaaaagagaaaatagaagcattaacagcaatttgtgatgaaatg

gaaaaggaaggaaaaattacaaagattgggcctgaaaatccatacaacactccaatattt

gccataaaaaagaaagacagtactaagtggagaaaattagtagatttcagggaactcaat

aaaagaactcaagatttttgggaagttcaattaggaataccacacccagcaggattaaaa

aagaaaaaatcagtgacagtgctggatgtgggagatgcatatttttcagttcctttatat

gaagacttcaggaaatatactgcattcaccatacctagtataaacaatgaaacaccaggr

attaggtatcagtacaatgtrcttccacagggatggaaaggatcaccagcaatatttcaa

agtagcatgayaaaaatcttagagccytttagaaaacaaaatccagacatagtcatctat

caatacatggatgatttatatgtaggrtctgacttagagatagggcagcatagaacaaaa

atagaggaactgagacaacatttgttgaggtggggatttaccacaccagacaagaaacat

cagaaagaacctccatttctttggatggggtatgaactccatcctgacaaatggacagta

cagcctatacagctgccagtacaagatagctggactgtcaatgatatacaaaagttagtg

ggaaaattaaactgggcaagtcagatytatcctggaattaaagtaaggcaactttgtaaa

ctccttaggggggccaaagcactaacagacatagtaccactaactgaagaagcagaatta

caga--------------------------------------------

>CRF07BC.26-712250

cctcagatcactctttggcaacgaccccttgttacaataaagataggggggcaactaaag

gaagctctattggatacaggagcagatgatacagtattagaagacatgaatttgccaggg

aaatggaaaccaaaaatgatagggggaattggaggttttatcaaagtaagacagtatgaa

cagatacccgtagaaatctgcggacacaaagctataggtacagtattagtaggacctaca

cctgtcaacataattggaagaaatctgttgactcagcttggttgcaccttaaactttcca

atcagtcctattgatactgtaccggtaaaattaaagccaggaatggatggcccaaaggtt

aaacaatggccattgacagaagaaaaaataaaagcattaacagcaatttgtgatgaaatg

gagaaggaaggaaaaatttcaaaaattgggcctgaaaacccatataacactccaatattt

gctataaaaaagaaggacagtactaagtggagaaaattagtagatttcagggaactcaat

aaaagaactcaagatttttgggaggttcaattaggaataccacacccagcagggttaaaa

aagaaaaaatcagtgacagtactggatgtgggggatgcatatttttcagttcctctacat

gaagatttcaggaagtatactgcattcaccatacctagtataaacaatgcaacaccaggg

attaggtatcagtacaatgtacttccacagggatggaaaggatcaccagcaatattccaa

agtagcatgacaaaaatcttagagccttttagaaaacaaaatccagacatagttatctat

caatacatggatgatttgtatgtaggctctgacttagagatagggcagcatagaacaaaa

gtagaggaactgagacaacatttgttgagatggggattcaccacaccagacaagaaacat

cagaaagaacctccatttctttggatggggtatgaactccatcctgacaagtggacagta

cagcctatacagctgccagaaaaagatagttggactgtcaatgatatacaaaagttagtg

ggaaaattaaattgggcaagtcagatttatcctggaatcaaagtaaggcaactttgtaaa

ctccttaggggggccaaagcactaacagaaataatacaattaactgaagaagcagaatta

g-----------------------------------------------

>CRF07BC.27-HUZI2076

cctcaaatcactctttggcaacgaccccttgttaccataaagataggggggcaattaaag

gaagctctattagatacaggagcagatgatacagtattagaagacatgaatttgccaggg

aaatggaaaccaaaaatgatagggggaattggaggttttatcaaagtaagacagtatgaa

cagatacccatagaaatctgtggacataaagctataggtacagtattagtaggacctaca

cctgtcaacataattggaagaaatctgttgactcagcttggttgtactttaaattttcca

atcagtcctattgaaactgtaccagtaaaactaaagccaggaatggatggcccaaaggtt

aaacaatggccattgacaaaagaraaaatagaagcattaacagcaatttgtgatgaaatg

gaaaaggagggaaaaattacaaaaattgggcctgaaaatccatacaacactccaatattt

gccataaaaaagaaagacagtactaagtggagaaaattagtagatttcagggaactcaat

aaaagaactcaagatttttgggaagttcaattaggaataccacacccagcaggattaaaa

aagaaaaaatcagtgacagtgctggatgtgggggatgcatatttttcagttcctctatat

gaagacttcaggaartatactgcattcaccatacctagtataaacaatgaaacaccaggg

attaggtatcagtacaatgtacttccacagggatggaaaggatcaccagcaatatttcaa

agtagcatgacaaaaatcttagagccttttagaaaacaaaatccagacatagtcatctat

caatacatggatgatttgtatgtaggatctgacttagagatagggcagcatagaacaaaa

atagaggaattgagacaacatttgttgaggtggggatttaccacaccagacaaaaaacat

cagaaagaacctccatttctttggatggggtatgaactccatcctgacaaatggacagta

cagcctatacagctgccagtacaagatagctggactgtcaatgatatacaaaagttagtg

ggaaaattaaactgggcaagtcagatttatcctggaattaaagtaaggcaactttgtaaa

ctccttaggggggccaaagcattaacagacatagtaccactaactgaagaagca------

------------------------------------------------

>CRF07BC.29-SX2012319

cctcaaatcactctttggcaacgaccccttgttaccataaagataggggggcaattaaag

gaagctctattagatacaggagcagatgatacagtattagaagacatgaatttgccaggg

aaatggaaaccaaaaatgatagggggaattggaggttttatcaaagtaagacagtatgaa

cagatacccatagaaatctgtggacataaagctataggtacagtattartaggacctaca

cctgtyaacataattggaagaaatctgttgactcagcttggttgtactttaaattttcca

atcagtcctattgaaactgtaccagtaaaactaaagccaggaatggatggcccaaaggtt

aaacaatggccattgacaaaagagaaaatagaagcattaacagcaatttgtactgaaatg

gaaaaggaaggaaaaattacaaaaattgggcctgaaaatccatacaacactccaatattt

gccataaaaaagaaagacagtactaagtggagaaaattagtagatttcagggaactcaay

aaaagaactcaagacttttgggaagttcaattaggaataccacacccagcagggttaaaa

aagaaaaaatcagtgacagtgctggatgtgggggatgcatatttttcagttcctttagat

gaagacttcaggaaatatactgcattcaccatacctagtataaacaatgaaacaccaggg

attaggtatcagtacaatgtacttccacagggatggaaaggatcaccagcgatatttcaa

agtagcatgacaaaaatcttagagccttttagaaarcaaaatccagacatagtcatctat

caatacatggatgatttgtatgtaggatctgacttagagatagggcagcatagaacaaar

atagaggaactgagacaacacttgttgaggtggggatttaccacaccagacaaaaaacat

cagaaagaacctccatttctttggatggggtatgaactccatcctgacaaatggacagta

cagcctatacagctaccagtacaagatagctggactgtcaatgatatacaaaagttagtg

ggaaaattaaactgggcaagtcagatttatcctggaattaaagtaaggcagctttgtaaa

ctccttaggggaaccaaagcactaacagacatagtaccactaactgaagaagcagaat--

------------------------------------------------

>CRF07BC.29-TT121831

cctcaaatcactctttggcaacggcccctagtcaaaataaagataggggggcaattaaag

gaagctctattagatacaggagcagatgatacagtattagaagacatgaatttgccaggg

aaatggaaaccaaaaatgatagggggaattggaggttttatcaaagtaagacagtatgaa

caagtacccatagaaatttgtggacacaaagctacaggaacagtattagtaggacctaca

cctgtcaacataattggaagaaacctattgactcagattggttgcactttaaattttcca

atcagtcccattgaaactgtaccagtaaaattaaagccaggaatggatggcccaaaggtt

aaacaatggccattgacagaagagaaaataaaagcattaacagcaatttgtgaggaaatg

gagaaggaaggaaaaattacaaaaattgggcctgaaaatccatataacactccaatattt

gccataaaaaagaaggacagtactaagtggagaaaattagtagatttcagggaactcaat

aaaagaactcaagatttttgggaagttcaattaggaataccacatccagcagggttaaaa

aagaaaaaatcagtgacagtactggatgtgggggatgcatatttttcagttcctttatat

gaagacttcaggaaatatactgcattcaccatacctagtataaacaatgaaacaccaggg

attagatatcagtataatgtacttccacagggatggaaaggatcaccagcaatattccaa

agtagcatgacaagaatcttagagccttttagaaaacaaaatccagacatggtgatctat

caatacatggatgatttgtatgtaggatctgacttagagatagggcaacatagaacaaaa

atagaagaactgagacaacatttgttcagatggggatttaccacacctgacaaaaaacat

cagaaagaacctccatttctttggatggggtatgaactccatcctgacaaatggacagta

cagcctatacagctaccacaaaaagatagctggactgtcaatgatatacagaagttagtg

ggaaaattaaactgggcaagtcagatttatcccggaatcaaagtaaggcaactttg----

------------------------------------------------------------

------------------------------------------------

>CRF07BC.30-20120348

cctcaaatcactctttggcaacgacccctcgtcacaataaggataggggggcaattaaag

gaagctctattagatacaggagcagatgatacagtattagaagatatgaatttaccaggg

aaatggaaaccaaaaatgatagggggaattggaggttttatcaaagtaagacagtatgaa

gagatacctgtagaaatytgcggacacaaagctataggtacagtattrgtaggacctaca

cctgtcaacataattggcagaaatytgttgactcagmttggttgcactttaaattttcca

atcagtcctattgaaactgtaccagtaaaattaaagccaggaatggatggcccaaaggtt

aaacaatggccattgacagaagagaaaataaaagcattaacagyaatttgtgaagaaatg

gagaaggagggaaaaatcacaaaaattgggcctgaaaatccatataacactccaatattt

gccataaaaaagaaggacagtactaaatggagaaaattagtagattttagggaactcaat

aaaagaactcaagatttttgggaagttcaattaggaataccacacccagcagggttaara

aagaaaaagtcagtgacagtactagatgtgggggatgcatatttttcagttcctttatat

gaagatttcaggaartatactgcattcaccatacctagtatgaacaatgaaacaccaggg

attaggtatcagtacaatgtactcccacagggatggaaaggatcaccagcaatattccaa

agtagcatgacaaaaatcttagagccgtttagaaaacaaaatccagacatagttatctat

caatacatggatgatttgtatgtaggatctgacttagagatagggcagcatagaacaaaa

atagargaactgagacaacatttgttgaggtggggatttaccacaccagacaagaaacat

cagaaggaacctccatttctttggatggggtatgaactccatcctgacaaatggacagta

cagcctatacagctgccagacaaagatagctggactgtcaatgatatacaaaagttagtg

ggaaaattaaactgggcaagtcagatatatcctggaattaaagtaaggcaactttgtaaa

ctccttagggggaccaaagcactaacagacatagtaccactaactgaagaagcagaa---

------------------------------------------------

>CRF07BC.30-SX2012348

cctcagatcactctttggcaacgacccctagtcccaataaagatagggggacaattaaag

gaagctctattagacacaggagcagatgatacagtattagaagacatgaatttgccaggg

aaatggaaaccaaaaatgatagggggaattggaggttttatcaaagtaagacaatatgaa

cagatacccatagaaatctgtggacacacaactgtaggtacagtattaataggacctaca

cctgtcaacataattggaagaaatctgttgactcagcttggttgcactttaaattttcca

atcagtcccattgaaactgtaccagtaaaattaaagccaggaatggatggcccaaaggtt

aaacaatggccattgacagaagagaaaatagaagcattaaaggcaatttgtgatgaaatg

gagaaggaaggaaaaattacaaaaattgggcctgaaaatccatataacaccccaatattt

gccataaaaaagaaagacagtactaaatggagaaaattagtagatttcagggaactcaat

aaaagaactcaggatttttgggaagttcaattaggaataccacacccagcagggttaaga

aagaaaaaatcagtgacagtactagatgtgggggatgcatatttttcagttcctttatat

gaagacttcaggaaatatactgcattcaccatacctagtaaaaacaatgaaacaccaggg

attaggtatcagtacaatgtacttccacagggatggaaaggatcaccagcaatattccaa

agcagcatgacaaaaatcttagagccctttagaaaacaaaatccagacatagttatctat

caatacatggatgatttgtatgtagggtctgacctagagatagggcagcatagaacaaaa

atagaggaactgagacaacatttgttgaggtggggatttaccacaccagacaagaaacat

cagaaagaacctccatttctttggatggggtatgaactccatcctgacaagtggacagta

cagcctatacagctaccagaaaaagatagctggactgtcaatgatatacagaagttagtg

ggaaaattaaactgggcaagtcagatttatcctggaattaaagtaaggcaactttgtaaa

ctccttaggggggccaaagcactaacagacatagtaccactaactgaagaagcaga----

------------------------------------------------

>CRF07BC.30-TT121986

cctcaaatcactctttggcagcgaccccttgttacaataaagataggggggcaattaaag

gaagctctattagatacaggagcagatgatacagtattagaagacatgaatttgccaggg

aaatggaaaccaaaaatgatagggggaattggaggttttatcaaagtaagacagtatgaa

cagatacccatagaaatctgtggacataaagccataggtacagtattaataggacctaca

cctatcaacataattggaagaaatctgttgactcagcttggttgtaccttaaattttcca

atcagtcctattgaaactgtaccagtaaaactaaagccaggaatggatggcccaaaggtt

aaacaatggccattgacaaaagagaaaatagaagcattaacagcaatttgtgatgaaatg

gaaaaggaaggaaaaattacaaaaattgggcctgaaaatccatacaacactccaatattt

gccataaaaaagaaagacagtactaagtggagaaaattagtagatttcagggaactcaat

aaaagaactcaagatttttgggaagttcaattaggaataccacacccagcaggattaaaa

aagaaaaaatcagtgacagtactggatgtgggggatgcatatttttcagttcctttacat

gaagatttcaggaaatatactgcattcaccatacctagtataaacaatgaaacaccaggg

attaggtatcagtacaatgtacttccacagggatggaaaggatcaccagcaatatttcaa

agtagtatgacaaaaatcttagagccttttagaaaacaaaatccagacatagtcatctat

caatacatggatgatttgtatgtaggatctgacttagagatagggcagcatagaacaaaa

atagaggaactgagacaacatctgttaaggtggggatttaccacaccagacaagaaacat

cagaaagaacctccatttctttggatgggttatgaactccatcctgacaaatggacagta

caacctatacagctgccagaacaagagagctggactgtcaatgatatacaaaagttagtg

ggaaaattaaactgggcaagtcagatttatcctggaattaaagtaaggcaactttgtaaa

cttcttaggggggccaaagcactaacagacatagtaccactaactgaagaagcag-----

------------------------------------------------

>CRF07BC.31-20120335

cctcaratcactctttggcaacgacccctcgtcacaataaggataggggggcaattaaag

gaagctctattagatacaggagcagatgatacagtattagaagatatgaatttaccaggg

aaatggaaaccaaaaatgatagggggaattggaggttttatcaaagtaagacagtatgaa

gagatacctrtagaaatctgcggrcacaaagctataggtacagtattggtaggacctaca

cctgtcaacataattggcagaaatctgttgactcagcttggttgcactttaaattttcca

atcagtcctattgaaactgtaccagtaaaattaaagccaggaatggatggcccaaaggtt

aaacaatggccattgacagaagagaaaataaaagcattaacagcaatttgtgaagaaatg

gagaaggarggaaaaatcacaaaaattgggcctgaaaatccatataacactccaatattt

gccataaaaaagaaggacagtactaaatggagaaaattagtagattttagggaactcaat

aaaagaactcaagatttttgggaagttcaattaggaataccacacccagcagggttaaga

aagaaaaagtcagtgacagtactagatgtgggggatgcatatttttcagttcctttatat

gaagacttcaggaaatatactgcattcaccatacctagtatgaacaaygaaacaccaggg

attaggtatcagtacaatgtactcccacagggatggaaaggatcaccagcaatattccaa

agtagcatgacaaaaatcttagagccgtttagaaaacaaaatccagrcatrgttatctat

caatacatggatgatttgtatgtaggatctgacttagagatagggcagcatagaacaaaa

atagaggaactgagagagcatytgttgargtggggattyaccacaccagacaaraaacat

cagaaggaacctccatttctttggatggggtatgaactccatcctgacaaatggacagta

cagcctatacagctgccagacaaagatagctggactgtcaatgatatacaaaagttagtg

ggaaaattaaactgggcaagtcagatatatcctggaattaaagtaaggcaactttgtaaa

ctccttagggggaccaaagcactaacagacatagtaccactaactgaagaagcagaatta

g-----------------------------------------------

>CRF07BC.32-G-HZ130260

cctcaaatcactctttggcaacgacccctagttacaataaagataggggggcaattaaag

gaagctctattagatacaggagcagatgatacagtattagaagacatgaatttgccagga

aaatggaaaccaaaaatgatagggggaattggaggttttatcaaagtaagacagtatgaa

cagatacccatagaaatctgtggacataaagctataggtacagtattagtaggacctaca

cctgtcaacataattggaagaaatctgttgactcagcttggttgtaccttaaattttcca

atcagtcctatcgaaactgtaccagtaaaactaaagccaggaatggatggcccaaaggtt

aaacaatggccattgacaaaagagaaaatagaagcattaacagcaatttgtgatgaaatg

gaaaaggaaggaaaaattacaaaaattgggcctgaaaatccatacaacactccaatattt

gccataaaaaagaaagacagtactaagtggagaaaattggtagatttcagggaactcaat

aaaagaactcaagatttttgggaagttcaattaggaataccacacccagcaggattaaaa

aagaaaaaatcagtgacagtgctggatgtgggggatgcatatttttcagttcctttatat

gaagacttcaggaaatatactgcattcaccatacctagtatgaacaatgaaacaccaggg

attaggtatcagtacaatgtacttccacagggatggaaaggatcaccagcaatatttcaa

agtagcatgacaagaatcttagagccttttagaaaacaaaatccagacatagtcatctat

caatacatggatgatttgtatgtaggatctgacttagagatagggcagcatagaacaaaa

atagaggaactgagacaacatttgttgaggtggggatttaccacaccagacaagaaacat

cagaaagaacctccatttctttggatggggtatgaactccatcctgacaaatggacagta

cagcctatacagctgccagtacaagatagctggactgtcaatgatatacaaaagttagtg

gggaaattaaactgggcaagtcagatttatcctggaattaaagtaaggcaactttgtaaa

ctccttagggggaccaaagcactaacagacatagtaccactaactgaagaagcagaa---

------------------------------------------------

>CRF07BC.34-TT120070

cctcaaatcactctttggcaacgaccccttgtcacaataaagataggggggcaattaaag

gaagctctattagatacaggagcggatgatacagtattagaagacataaatttgccaggg

aaatggaaaccaaaaatgatagggggaattggaggttttatcaaagtaagacagtatgaa

cagatacccatagaaatctgtggacatcaagttataggcacagtattaataggacctaca

cctgtcaacataattggaagaaatctgttgactcagcttggttgtactttaaattttcca

atcagtcctattgaaactgtaccagtaaaactaaagccaggaatggatggcccaaaggtt

aaacaatggccattgacaaaagagaaaatagaagcattaacagcaatttgtgatgaaatg

gaaaaggaaggaaaaattacaaaaattgggcctgaaaatccatacaacactccaatattt

gccataaaaaagaaagacagtactaagtggagaaaattagtagatttcagggaactcaat

aaaagaactcaagatttttgggaagttcaattaggaataccacacccagcaggattaaaa

aagaaaaaatcagtgacagtgctggatgtgggggatgcatatttttcagttcctttacat

gaagactttaggaaatatactgcattcaccatacctagtataaacaatgaaacaccaggg

attaggtatcagtacaatgtacttccacagggatggaaaggatcaccagcaatatttcaa

agtagcatgacaagaatcttagaaccttttagaaaacagaatccagacatagtcatctat

caatacatggatgatttgtatgtaggatctgacttagagatagggcagcatagaacaaaa

atagaggaactgagacaacatttgttgaggtggggatttaccacaccagacaagaaacat

cagaaagaacctccatttctttggatggggtatgaactccatcctgataaatggacagta

cagcctatacagctgccagaacaagatagctggactgtcaatgatatacaaaagttagtg

ggaaaattaaactgggcaagtcagatttatcctggaattaaagtaaggcaactttgtaaa

ctccttagggggaccaaagcactaacagacatagtaccactaactgaagaagcaga----

------------------------------------------------

>CRF07BC.35-SX2012206

cctcaaatcactctttggcagcgaccccttgttacmataaagataggggggcaattaaag

gaagctctattagatacaggagcagatgatacagtattagaagacatgaatttgccaggg

aaatggaaaccaaaaatgatagggggaattggaggttttatcaaagtaagacagtatgaa

cagatacccatagaaatctgtggacataaagctataggtacagtattagtaggacccaca

cctgtcaacataattggaagaaatctgttgactcagmttggttgtaccttaaattttcca

atcagtcctattgaaactgtaccagtaaaactaaagccaggaatggatggcccaaaggtt

aaacaatggccattgacaaaagagaaaatagaagcattaacagcaatttgtgatgaaatg

gaaaaggaaggaaaaattacaaaaattgggcctgaaaatccatacaacactccaatattt

gccataaaaaagaaagacagtactaagtggagaaaattagtagatttcagrgaactcaat

aaaagaactcaagatttttgggaagttcaattaggaataccacayccagcaggattaaaa

aagaaaaaatcagtgacagtgctggatgtgggrgatgcatatttttcagttcctttayat

gaagacttcaggaaatatactgcattcacyatacctagtataaacaatgaaacaccaggg

attaggtatcagtacaatgtacttccacagggatggaaaggatcaccagcaatatttcaa

agtagcatgacaaaaatcttagarccttttagaaaacaaaatccagacatagtcatctat

caatacatggatgatttgtatgtaggatctgacttagagatagggcagcatagaacaaaa

atagaggaactgagacaacatttgttgaagtggggatttaccacaccagacaagaaacat

cagaaagaacctccatttctttggatggggtatgaactccatcctgacaaatggacagta

cagcctatacagctgccagtacaagatagctggactgtcaatgatatacaaaagttagtg

ggaaaattaaactgggcaagtcagatttatcctggaattaaagtaaggcaactttgtaaa

ctccttagggggaccaaagcactaacagacatagtaccactaactgaagaagcaga----

------------------------------------------------

>CRF07BC.36-12ZJ1138

cctcaaatcactctttggcaacgaccacttgttacaataaagataggggggcaattaaag

gaagctctattagatacaggagcagatgatacagtattagaagacatgaatttgccaggg

aaatggaaaccaaaaatgatagggggaattggaggttttatcaaagtaaaacagtatgaa

caggtgcccatagaaatttgtggacataaagttataggtacagtattagtaggacctaca

cctgtcaacataattggaaggaatctgttgactcagcttggttgtactttaaattttcca

atcagtcctattgaaactgtaccagtaaaactaaagccaggaatggatggcccaaaggtt

aaacaatggccattgacaaaagagaaaatagaagcattaacagcaatctgtgatgaaatg

gaaaaggaaggaaaaattacaaaaattgggcctgaaaacccatacaacactccaatattt

gccataaaaaagaaagacagtactaagtggagaaaattagtagatttcagggaactcaat

aaaagaactcaagatttttgggaagttcaattaggaataccacacccagcaggattaaaa

aagaaaaaatcagtgacagtgctggatgtgggggatgcatatttttcagttcctttacat

gaagacttcaggaaatatactgcattcaccatacctagtataaacaatgaaacaccaggg

attaggtatcagtacaatgtacttccacagggatggaaaggatcaccagcaatatttcaa

agtagcatgacaaaaatcttagagccttttagaaaacaaaatccagacatagtcatctat

caatacatggatgatttgtatgtaggatctgacttagagatagggcarcatagaacaaaa

atagaggaactgagacaacatttgttgaggtggggatttaccacaccagacaagaaacat

cagaaagaacctccatttctttggatggggtatgaactccatcctgacaaatggacagta

cagcctatacagttgccagaacaagatagctggactgtcaatgatatacaaaagttagtg

ggaaaattaaactgggcaagtcagatttatcctggaattaaagtaaggcaactttgtaaa

ctccttagggggaccaaagcactaacagacatagtaccactaactgaagaagca------

------------------------------------------------

>CRF07BC.36-SX2012209

cctcaaatcactctttggcaacgaccccttgttacaataaagataggggggcaattaaag

gaagctttattagatacaggagcagatgatacagtattagaagacatgaatttgccagga

aaatggaaaccaaaaatgatagggggaattggaggttttatcaaagtaagacagtatgaa

gagatacccatagaaatctgtggacataaagctataggtacagtattagtaggacctaca

cctatcaacataattggaaggaatctgttgactcagcttggttgtactttaaattttcca

atcagtcctattgatactgtaccagtaaaactaaagccaggaatggatggcccaaaggtt

aaacaatggccattgacaaaagagaaaatagaagcattaacagcaatttgtgatgaaatg

gaaaaggaaggaaaaattacaaagattgggcctgaaaatccatacaacactccaatattt

gccataaaaaagaaagacagtactaagtggagaaaattagtagatttcagggaactcaat

aaaagaactcaagatttttgggaagttcaattaggaataccacacccagcaggattaaaa

aagaaaaaatcagtgacagtgctggatgtgggagatgcatatttttcagttcctttagat

gaagacttcaggaaatatactgcattcaccatacctagtacaaacaatgaaacaccaggg

gttaggtatcagtacaatgtacttccacagggatggaaaggatcaccagcaatatttcaa

agtagcatgacaaaaatcttagagccttttagaaaacaaaatccagacatagtcatctat

caatacatggatgatttgtatgtaggatctgacttagagataaagcagcatagaacaaaa

atagaggaactgagacaacatttgttgaggtggggatttaccacaccagacaagaaacat

cagaaagaacctccatttctttggatggggtatgaactccaccctgacaaatggacagta

cagcccatccagctgccagtacaagatagctggacggtcaatgatatacaaaagttagtg

ggaaaattaaactgggcaagtcagatttaccctggaatcaaaacaaggcaactttgtaaa

ctccttagggggaccaaagcactaacagacatagtaccactaactgaagaagcagaat--

------------------------------------------------

>CRF07BC.36-TT120195

cctcaaatcactctttggcaacgaccccttgttacaataaagataggggggcaattaaag

gaggctctattagatacaggagcagatgatacagtattagaagacatgaatttgccaggg

aaatggaaaccaaaaatgatagggggaattggaggttttatcaaagtaagacagtatgaa

cagatacccatagaaatctgtggacataaagctataggtacagtattagtaggacctaca

cctgtcaacataattggaagaaatckgttgactcagcttggttgtactttaaattttcca

atcagtcctattgaaactgtaccagtaaaactaaagccaggaatggatggcccaaaggtt

aaacaatggccattgacaaaagagaaaatagaagcattaacagcaatttgtgatgaaatg

gaaaaggaaggaaaaattacaaaaattgggcctgaaaatccatacaatactccaatattt

gccataaaaaagaaagacagtactaagtggagaaaattagtggatttcagggaactcaat

aaaagaactcaagatttttgggaagttcaattaggaataccacacccagcaggattaaaa

aagaaaaaatcagtgacagtgctggatgtgggggatgcatatttctcagttcctttacat

gaagacttcaggaaatatactgcattcaccatacctagtacaaacaatgaaacaccaggg

attaggtatcagtacaatgtacttccacagggatggaaaggatcaccagcaatatttcaa

agtagcatgacaaaaatcttagagccttttagaaaacaaaatccagacatagtcatctat

caatacatggatgatttgtatgtaggatctgacttagagatagggcagcatagaacaaaa

atagaggaactgagacaacatctgttgaggtggggatttaccacaccagacaagaaacat

cagaaagaacctccatttctttggatggggtatgaactccatcctgacaaatggacagta

cagcctatacaactgccagtacaagatagctggactgtaaatgatatacaaaagttagtg

ggaaaattaaactgggcaagtcagatttatcctggaattaaagtaaggcaactttgtaaa

ctccttaggggggccaaagcactaacagacatagtaccactaactgaagaagcagaatta

g-----------------------------------------------

>CRF07BC.37-20121399

cctcaaatcactctttggcaacgaccccttgtcacaataaagataggggggcaattaaag

gaagctctattagatacaggagcagatgatacagtattagaagacatgaatttgccaggg

aaatggaaaccaaaaatgatagggggaattggaggttttattaaagtaagacaatatgaa

cagatacccatagaaatctgtggacataaagctataggtacagtattagtaggacctaca

cctgtcaacataattggaagaaatctgttgactcagcttggttgtactttaaattttcca

atcagtcctattgaaactgtaccagtaaaactaaagccaggaatggatggcccaaaggtt

aaacaatggccattgacaaaagagaaaatagaagcattaacagcaatttgtgatgaaatg

gaaaaggaaggaaaaattacaaaaattgggcctgaaaatccatacaacactccaatattt

gccataaaaaagaaagacagtactaagtggagaaaattagtagacttcagggaactcaat

aaaagaactcaagatttttgggaagttcaattaggaataccacacccagcaggattaaaa

aagaaaaaatcagtgacagtgctggatgtaggggatgcatatttttcagttcctttacat

gaagacttcaggaaatatactgcattcaccatacctagtataaacaatgaaacaccagga

attaggtatcagtacaatgtacttccacagggatggaaaggatcaccagcaatatttcaa

agtagcatgacaaaaatcttagagccttttagaaaacaaaatccagacatagtcatctat

caatacatggatgatttgtatgtaggatctgacttagagatagggcagcatagaacaaaa

atagaggaactgagacaacatttgttgaggtggggatttaccacaccagacaaaaaacat

cagaaagaacctccatttctttggatggggtatgaactccatcctgacaaatggacagta

cagcctatacagctgccagtacaagatagctggactgtcaatgatatacaaaagttagtg

ggaaaattaaactgggcaagtcagatttatcctggaattaaagtaaggcaactttgtaaa

ctccttaggggggccaaagcactaacagacatagtaccactaactgaagaagcaga----

------------------------------------------------

>CRF07BC.37-SX2012212

cctcaaatcactctttggcagcgacccctcgtcacaataaagataggggggcaattaaag

gaagctctattagacacaggagcagatgatacagtattagaggacatgaatttgccaggg

aaatggagaccaaaaatgatagggggaattggaggttttatcaaagtaagacagtatgag

cagatacccatagaaatctgcgggcacaaagttataggtacagtgttagtaggatctaca

cctgtcaacataatkggaagaaatctgttgactcagcktggttgcactttaaattttcca

atcagtcctattgaaactgtaccagtaaaattaaagccaggaakggatggcccaaaggtt

aaacaatggccattgacagaagaaaaaataaaagcattaacagaaatttgtaatgaaatg

gagaaggaaggaaaaattacaaaaattgggcctgaaaatccatataacactccaatattt

gccataaaaaagaaggacagtactaagtggagaaaattagtagatktcagggaactcaat

aaaagaactcaagatttttgggaagttcagttaggaataccacacccagcagggttaaaa

aagaaaaaatcagtgacagtactggatgtgggggatgcatatttttcagttcctttatat

gaagacttcaggaaatatactgcattcaccatacctagtataaacaatgaaacaccaggg

attaggtatcagtacaatgtacttccacagggatggaaaggatcaccagcaatattccaa

agtagcatgacaaaaatcttagagccttttagaaagcaacatccagacatagttatctat

cagtacatggatgatttgtatgtaggatctgatttagaaatagggcagcatagaacaaaa

atagaagaactgagacaacatttgttaacgtggggatttaccacaccagacaaaaaacat

cagaaagaacctccatttctttggatggggtatgaactccatcctgacaaatggacagta

cagcctatacagctgccagaaaaagatagctggactgtcaatgatatacaaaagttagtg

ggaaaattaaactgggcaagtcagatttatcctggaattaaagtaaggcaactttgtaaa

ctccttagaggggccaaagggctaacagacatagtaccactaactgaa------------

------------------------------------------------

>CRF07BC.37-TT120332

cctcaaatcactctttggcaacgaccccttgttaccataaagataggggggcaattaaag

gaagctctattagatacaggagcagatgatacagtattagaagacatgaatttgccaggg

aaatggaaaccaaaaatgatagggggaattggaggttttatcaaagtaagacagtatgaa

cagatacccatagaaatctgtggacataaagctataggtacagtattagtaggacctaca

cctgtcaacataattggaagaaatctgttgactcagcttggttgtactttaaattttcca

atcagtcctattgaaactgtaccagtaaaactaaagccaggaatggatggcccaaaggtt

aaacaatggccattgacaaaagagaaaatagaagccttaacagcaatttgtgttgaaatg

gaaaaggaaggaaaaattacaaaaattgggcctgaaaatccatacaacactccaatattt

gccataaaaaagaaagacagtactaagtggagaaaattagtagatttcagggaactcaat

aaaagaactcaagatttttgggaagttcaattaggaataccacacccagcaggattaaaa

aagaaaaaatcagtgacagtgctggatgtgggggatgcatatttttcagttcctttatat

gaagacttcaggaaatatactgcattcaccatacctagtataaacaatgaaacaccaggg

actaggtatcagtacaatgtacttccacagggatggaaaggatcaccagcaatatttcaa

agtagcatgacaaaaatcttagagccttttagaaaacaaaatcctgacatagtcatctat

caatacatggatgatttgtatgtaggatctgacttagagatagggcagcatagaacaaaa

atagaggaactgagacaacacttgttgaggtggggatttaccacaccagacaaaaaacat

cagaaagaacctccatttctttggatggggtatgaactccatcctgacaaatggacagta

cagcctatacagctaccagtacaagatagctggactgtcaatgatatacaaaagttagtg

ggaaaattaaactgggcaagtcagatttatcctggaattaaagtaaggcagctttgtaaa

ctccttagggggaccaaagcactaacagacatagtaccactaactgaagaagcagaatta

gaattggcaga-------------------------------------

>CRF07BC.39-20120863

cctcaaatcactctttggcaacgacccctcgtcccaataaagataggggggcaatcaaag

gaagctctattagatacaggagcagatgatacagtattagaagacatgaatttgccaggg

aaatggacaccaaaaatgatagggggaattggaggttttatcaaagtaagacaatatgaa

cagatacccatagaaatctgtggacacaaaactataggtacagtattagtaggacctaca

cctgtcaacataattggaagaaatctgttgactcagattggttgcactttaaattttcca

atcagtcccattgaaactgtaccagtaaaattaaagccaggaatggatggcccaaaggtt

aaacaatggccattgacagaagagaaaataaaagcattaacagaaatttgtraagaaatg

gaaaaagaaggaaaaattacaaaaattgggcctgaaaatccatataacactccaatattt

gccataaaaaagaaggacagtactaagtggagaaaattagtagatttcagggaacttaat

aagagaactcaagatttttgggaagttcaattaggaataccacacccagcagggttaaag

aagaaaaagtcagtgacagtactggatgtgggggatgcatatttttcagttcctttacat

gaagacttcaggaaatatactgcattcaccatacctagtagaaacaatgaaacaccagga

attaggtatcagtacaatgtacttccgcagggatggaaaggatcaccagcaatattccaa

actagcatgacaaaaatcttagaaccttttagaaaacaaaatccagacctagttatctat

caatacatggatgatttatatgtaggatctgacttagagatagggcagcatcgaacaaaa

atagaggaactgagacaacatttgttgaggtggggatttaccacaccagacaagaaacac

cagaaagaacctccatttctttggatggggtatgaactccatcctgacaaatggacagta

cagcctatacatctaccagaaaaagatagctggactgtcaatgatatacaaaagttagtg

ggaaaattaaactgggcaagtcagatttatcctggaattaaagtaagacaactttgtaaa

ctccttaggggggccaaagcactaacagacatagtaccactaactgaagaagcagaa---

------------------------------------------------

>CRF07BC.39-330103018-2012-00105

cctcaaatcactctttggcagcgaccccttgttaccataaagataggggggcaattaaag

gaagctttattagatacaggagcagatgatacagtattagaagacatggatttgccaggg

aaatggaaaccaaaaatgatagggggaattggaggttttatcaaagtaagacagtatgaa

cagattcccatagaaatctgtggacataaaactataggtacagtattagtaggacctaca

cctgtcaacataattggaagaaatctgttgactcagcttggttgtactttaaattttcca

atcagtcctattgaaactgtaccagtaaaactaaagccaggaatggatggcccaaaggtt

aaacaatggccattgacaaaagagaaaatagaagcattaacagcaatttgtgttgaaatg

gaaaaggaaggaaaaattacaaaaattgggcctgaaaatccatacaacactccaatattt

gccataaaaaagaaagacagtactaagtggagaaaattagtagattttagggaacttaat

aaaagaactcaagatttttgggaagttcaattaggaataccacacccagcaggattaaaa

aagaaaaaatcagtgacagtgctggatgtgggggatgcatatttttcagttcctttatat

gaagacttcaggaaatatactgcattcaccatacctagtataaacaatgaaacaccaggg

attaggtatcagtacaatgtacttccacagggatggaaaggatcaccagcaatatttcaa

agtagcatgacaaaaatcttagagccttttagaaaacaaaatccagacatagtcatctat

caatacatggatgatttgtatgtaggatctgacttagagatagggcaacatagaacaaaa

atagaggaactgagacaacatttgttgaggtggggatttaccacaccagacaaaaaacat

cagaaagaacctccatttctttggatggggtatgaactccatcctgacaaatggacagta

cagcctatacaactgccagtacaagatagctggactgtcaatgatatacaaaaattagtg

ggaaaattaaactgggcaagtcagatttatcctggaattaaagtaaggcaactttgtaaa

ctccttagggggaccaaagcgttaacagacatagtaccactaactgaagaagcag-----

------------------------------------------------

>CRF07BC.4-12ZJ0948

cctcaaatcactctttggcagcgaccccttgtcacaataaagataggggggcaattaaag

gaagctctattagatacaggagcagatgatacagtattagaagacatgaatttgccaggg

aaatggaaaccaaaaatgatagggggaattggaggttttattaaagtaagacagtatgaa

caggtacccatagaaatctgtggacataaagctataggtacagtattagtaggacctaca

cctgtcaacataattggaagaaatttgttgactcagcttggytgtactttaaattttcca

atcagtcctattgaaactataccagtaaaactaaagccaggaatggatggcccaaaggtt

aaacaatggccattgacaaaagagaaaatagaagcattaacagcaatttgtgatgaaatg

gaaaaggaaggaaaaattacaaaaattgggcctgaaaatccatacaacactccaatattt

gccataaaaaagaaagacagtactaagtggagaaaattagtagattttagggagctcaat

aaaagaactcaagatttttgggaagttcaattaggaataccacacccagcaggattaaaa

aagaaaaaatcagtgacagtgctggatgtgggggatgcatatttttcagttcctttacat

gaagacttcaggaaatataccgcattcaccatacctagtataaacaatgaaacaccaggg

attaggtatcagyacaatgtacttccacagggatggaaaggatcaccagcaatatttcaa

agtagcatgacaaaaatcttagagccttttagaaaacaaaatccagacatagtcatctat

caatacatggatgatttgtatgtaggatctgacttagagatagggcagcatagaacaaaa

atagaggaactgagacaacatttgttgaggtggggatttaccacaccagacaagaaacat

cagaaagaacctccatttctttggatggggtatgaactccatcctgacaaatggacagta

cagcctatacagctgccagtacaagatagctggactgtcaatgatatacaaaagttagtg

ggaaaattaaactgggcaagtcagatttatcccggaattaaagtaaggcaactttgtaaa

ctccttaggggggccaaagcactaa-----------------------------------

------------------------------------------------

>CRF07BC.4-CD4-142936

cctcaaatcactctttggcaacgacccctagttacaataaagataggggggcaattaaag

gaagctctattagatacaggagcagatgatacagtattagaagacatgaatttgccagga

aaatggaaaccaaaaatgatagggggaattggaggttttatcaaagtaagacartatgaa

cagatactcatagaaatctgtggacataaagctataggtacagtattagtaggacctaca

cctgtcaacataattggaagaaatctgttgactcagcttggttgtaccttaaattttcca

atcagtcctatcgaaactgtaccagtaaaactaaagccaggaatggatggcccaaaggtt

aaacaatggccattgacaaaagagaaaatagaagcattaacagcaatttgtgatgaaatg

gaaaaggaaggaaaaattacaaaaattgggcctgaaaatccatacaacactccaatattt

gccataaaaaagaaagacagtactaagtggagaaaattagtagatttcagggaactcaat

aaaagaactcaagatttttgggaagttcaattaggaataccacacccagcaggattaaaa

aagaaaaaatcagtgacagtgctggatgtgggggatgcatatttttcagttcctttatat

gaagacttcaggaaatatactgcattcaccatacctagtatgaacaatgaaacaccaggg

attaggtatcagtacaatgtacttccacagggatggaaaggatcaccagcaatatttcaa

agtagcatgacaagaatcttagagccttttagaaaacaaaatccagacatagtcatctat

caatacatggatgatttgtatgtaggatctgacttagagatagggcagcatagaacaaaa

atagaggaactgagacaacatttgttgaggtggggatttaccacaccagataagaaacat

cagaaagaacctccatttctttggatggggtatgaactccatcctgacaaatggacagta

cagcctatacagctgccagtacaagatagctggactgtcaatgatatacaaaagttagtg

gggaaattaaactgggcaagtcagatttatcctggaattaaagtaaggcaactttgtaaa

ctccttagggggaccaaagcactaacagacatagtaccactaactgaagaagcaga----

------------------------------------------------

>CRF07BC.40-12ZJ0512

cctcaaatcactctttggcaacgaccccttgttacaataaagataggggggcaattaaag

gaagctttattagatacaggagcagacgatacagtattagaagacatgaatttgccagga

aaatggaaaccaaaaatgatagggggaattggaggttttatcaaagtaagacagtatgaa

gagatacccatagaaatctgtggacataaagctataggtacagtattagtaggacctaca

cctgtcaacataattggaaggaatctgttgactcagcttggttgtactttaaattttcca

atcagtcctattgatactgtaccagtaaaactaaagccaggaatggatggcccaaaggtt

aaacaatggccattgacaaaagagaaaatagaagcattaacagcaatttgtgatgaaatg

gaaaaggaaggaaaaattacaaaaattgggcctgaaaatccatacaacactccaatattt

gccataaaaaagaaagacagtactaagtggagaaaattagtagatttcagggaactcaat

aaaagaactcaagatttttgggaagttcaattaggaataccacacccagcaggattaaaa

aagaaaaaatcagtgacagtgctggatgtgggagatgcatatttttcagttcctttagat

gaagacttcaggaaatatactgcaytcaccataccaagtayaaacaatgaaacaccaggg

attaggtatcagtacaatgtacttccacagggatggaaaggatcaccagcaatatttcaa

agtagcatgacaaaaatcttagagccttttagaaaacaaaatccagacatagtcatctac

caatacatggatgatttatatgtaggatctgacttagagataaggcagcatagaacaaaa

atagaggaactgagacaacatttgttgaggtggggatttaccacaccagacaagaaacat

cagaaagaacccccattcctttggatggggtatgaactccaccctgacaaatggacagta

cagcctatacagctgccagtacaagatagctggacggtcaatgatatacaaaagttagtg

ggaaaattaaactgggcaagtcagatttatcctggaattaaaacaaggcaactttgtaaa

ctccttaggggggccaaagcactaacagacatagtaccactaactgaagaagcagaatta

gaattggcaga-------------------------------------

>CRF07BC.40-G-HZ130410

cctcaaatcactctttggcaacgaccccttgttaccataaagataggggggcaagtaaag

gaagctctattagatacaggagcagatgatacagtattagaagayatgaatttgccaggr

aaatggaaaccaaaaatgatagggggaattggaggttttatcaaagtaagacagtatgaa

cagatacccatagaaatctgtggacatagagctataggtacagtattagtaggacctaca

cctgtcaacataattggaagaaatctgttgactcagcttggttgtactttaaattttcca

atcagtcctattgaaactgtaccagtaaaactaaagccaggaatggatggcccaaargtt

aaacaatggccattgacaaaagagaaaatagaagcattaacagcaatttgtgawgaaatg

gaaaaggaaggaaaaattacaaaaattgggcctgaaaatccatacaacactccaatattt

gccataaaaaagaaagacagtactaagtggagaaaattagtagatttcagggaactcaat

aaaagaactcaagatttttgggaagttcaattaggaataccacacccagcaggattaaaa

argaaaaartcrgtgacagtgctggatgtgggggatgcatatttttcagttcctttakat

gaagacttcaggaaatatactgcattcaccatacctagtrtaaacaatgaaacaccaggg

actaggtatcagtacaatgtacttccacagggatggaaaggatcaccagcaatatttcaa

agtagtatgacaaaaatcttagaaccttttagaaaacaaaatccagacatagtcatctat

caatacatggatgatttgtatgtaggatctgacttagagatagggcagcatagaacaaaa

atagargaactaagrcaacatttgttgavgtggggatttaccacaccagacaagaaacat

cagaaagaacctccatttctttggatggggtatgaactccatcctgacaaatggacagta

cagcctatacagctgccagtacaagatagctggactgtcaatgatatacaaaagttagtg

ggaaaattaaactgggcaagtcagatttatcctggaattagagtaaggcaactgtgtaaa

ctccttaggggggccaaagcactaacagacatagtaccattaactgaagaagcagaatta

gaa---------------------------------------------

>CRF07BC.41-SX2012088

cctcaaatcactctttggcaacgaccccttgttacaataaagatagggggacaattaaag

gaagctctattagatacaggagcagacgatacagtattagaagacatgaatttgccagga

aaatggaaaccaaaaatgatagggggaattggaggttttatcaaagtaagacagtatgaa

gagatacccatagaaatctgtggacataaagctataggtacagtattagtaggacctaca

cctatcaacataattggaaggaatctattgactcagcttggttgtactttaaattttcca

atcagtcctattgatactgtaccagtaaaactaaagccaggaatggatggcccaaaggtt

aaacaatggccattgacaaaagagaaaatagaagcattaacagcaatttgtgatgaaatg

gaaaaggaaggaaaaattacaaaaattgggcctgaaaatccatacaacactccaatattt

gccataaaaaagaaagacagtactaagtggagaaaattagtagatttcagggaactcaat

aaaagaactcaagatttttgggaagttcaattaggaataccacacccagcaggattaaaa

aagaaaaaatcagtgacagtgctggatgtgggagatgcatatttttcagttcctttagat

gaagacttcaggaaatatactgcattcaccatacctagtataaacaatgaaacaccaggg

attaggtatcagtacaatgtacttccacagggatggaaaggatcaccagcaatatttcaa

agtagcatgacaaaaatcttagagccttttagaaaacaaaatccagacatagtcatctat

caatacatggatgatttgtatgtgggatctgacttagagataaagcagcatagaacaaaa

atagaggaactgagacaacatttgttgaggtggggacttaccacaccagacaagaaacat

cagaaagaacctccatttctttggatggggtatgaactccaccctgacaaatggacagta

cagcctatacagctgccagtacaagacagctggacggtcaatgatatacaaaagttagtg

ggaaaattaaactgggcaagtcagatttatcctggaattaaaacaaggcaactttgtaaa

ctccttaggggggtcaaagcactaacagacatagtaccactaactgaagaagcagaa---

------------------------------------------------

>CRF07BC.41-TT121056

cctcaaatcactctttggcaacgaccccttgttacaataaagataggggggcaattaaag

gaagctctattagatacaggagcagatgatacagtattagaagaaatgaatttgccaggg

aaatggaaaccaaaaatgatagggggaattggaggttttatcaaagtaagacagtatgaa

cagatacccgtagaaatctgtggacataaagctataggtacagtattaataggacctaca

cctgtcaacataattggaagaaatctgttgactcagcttggttgtactttaaattttcca

atcagtcctattgaaactgtaccagtaaaactaaagccaggaatggatggcccaaaggtt

aaacaatggccgttgacaaaagagaaaatagaagcattaacagcaatttgtgatgaaatg

gaaaaggaaggaaaaattacaaaaattgggcctgaaaatccatacaacactccaatattt

gctataaaaaagaaagacagtactaagtggagaaaattagtagatttcagggaactcaat

aaaagaactcaagatttttgggaagttcaattaggaataccacacccagcaggattaaaa

aagaaaaaatcagtgacagtgctggatgtgggggatgcatatttttcagttccgttacat

gaagacttcaggaaatatactgcattcaccatacctagtayaaacaatgaaacaccaggg

attaggtatcagtacaatgtacttccacagggatggaaaggatcaccagcaatatttcaa

agtagcatgacaaaaatcttagagccttttagaaaacaaaatccagacatagtcatttat

caatacatggatgatttgtatgtaggatctgacttagagatagggcagcatagaaccaaa

atagaggaactgagacaacatttgttgagatggggatttaccacaccagacaagaaacat

cagaaagaacctccatttctttggatggggtatgaactccatcctgataaatggacagta

cagcctatacagctgccagtacaagatagctggactgtcaatgatatacaaaagttagtg

ggaaaattaaactgggcaagtcagatttatcctggaattaaagtaaggcaactttgtaaa

ctccttaggggggccaaagcactaacagacatagtaccactaactgaagaagcaga----

------------------------------------------------

>CRF07BC.42-20120690

cctcaaatcactctttggcaacgacccctcgtcgcaataaggataggggggcaattaaag

gaagctctattagatacaggagcagatgatacagtattagaagaaatgaatttaccagga

aaatggaaaccaaaaatgatagggggaattggaggttttatcaaagtaagacagtatgaa

gagatacctgtagaaatctgcggacacaaagctataggtacagtattagtaggacctaca

cctgtcaacataattggcagaaatctgttgactcagcttggttgcactttaaattttcca

atcagtcctattgaaactgtaccagtaaaattaaagccaggaatggatggcccaaaggtt

aaacaatggccattaacagaagagaaaataaaagcattaacagcaatttgtgatgaaatg

gagaaggaaggaaaaatcacaaaaattgggcctgaaaatccatataacactccaatattt

gccataaaaaagaaggacagtactaaatggagaaaattggtagattttagggaactcaat

aaaaggactcaagatttttgggaagttcaattaggaataccacacccagcagggttaaga

aagaaaaagtcagtgacagtactagatgtgggggatgcatatttttcagttcctttatat

gaagacttcaggaagtatactgcattcaccatacctagtataaacaacgaaacaccaggg

attaggtatcagtacaacgtactcccacagggatggaaaggatcaccagcaatattccaa

agtagcatgacaaaaatcttagagccgtttagaaaacaaaatccagatatagttatctat

caatacatggatgacttgtatgtaggatctgacttagagatagggcagcatagaacaaaa

atagaggaactgagacaacatttgttgaggtggggatttaccacaccagacaagaaacat

cagaaagaacctccatttctttggatggggtatgaactccatcctgacaaatggacagta

cagcctatacagctaccaaacaaagatagctggactgtcaatgatatacaaaagttagtg

ggaaaattaaactgggcaagtcagatatatcctggaattaaagtaaggcaactttgtaaa

ctccttagggggaccaaagcactaacagacatagtaccactaactgaagaagcagaatt-

------------------------------------------------

>CRF07BC.42-330104008-2012-00007

cctcaaatcactctttggcaacgaccccttgttgccataaagataggggggcaattaaag

gaagctctattagatacaggagcagatgatacagtattagaagacatgaatttgccaggg

aaatggaaaccaaaaatgatagggggaattggaggttttatcaaagtaagacagtatgaa

cagatacccatagaaatctgtggacataaagctataggtacagtattaataggacctaca

cctgtcaacataattggaagaaatctgttgactcagcttggttgtactttaaattttcca

atcagtcctattgaaactgtaccagtaaaactaaagccaggaatggatggcccaaaggtt

aaacaatggccattgacaaaagaaaaaatagaagcactaacagcaatttgtgatgaaatg

gaaaaggaaggaaaaattacaaaaattgggcctgaaaatccatacaacactccaatattt

gccataaaaaagaaagacagtactaagtggagaaaattagtagatttcagggaactcaat

aaaagaactcaagatttttgggaagttcaattaggaataccacacccagcaggattaaaa

aagaaaaaatcagtgacagtgctggatgtgggggatgcatatttttcagttcctttatat

gaagacttcaggaaatatactgcattcaccatacctagtataaacaatgaaacaccaggg

attaggtatcagtataatgtacttccacagggatggaaaggatcaccagcaatatttcaa

agtagcatgacaaaaatcttagaacctttcagaaaacaaaatccagacatagtcatctat

caatacatggatgatttgtatgtaggatctgacttagagatagggcagcatagaacaaaa

atagaggaactgagacaacatctgttaaggtggggatttaccacaccagacaagaaacat

cagaaagaacctccatttctttggatggggtatgaactccatcctgacaaatggacagta

cagcccatacagctgccagtacaagatagctggactgtcaatgatatacaaaagttagtg

ggaaaattaaactgggcaagtcagatttatcctggaattaaagtaaggcaactttgtaaa

ctccttagaggggccaaagcactaacagacatagtaccactaactgaagaagcagaatta

g-----------------------------------------------

>CRF07BC.42-G-HZ130413

cctcaaatcactctttggcaacgaccccttgttaccataaagatagggggrcaaktaaag

gaagctctattagatacaggagcagatgatacagtattagaagaaatgaatttgccaggg

aaatggaaaccaaaaatgatagggggaattggaggttttattaaagtaagacagtatgaa

cagatacccatagaaatctgtggacataaagctataggtacagtattaataggacctaca

cctgtcaacataattggaagaaatctgttgactcagcttgggtgtactttaaattttcca

atcagtcctattgaaactgtaccagtaaaactaaagccaggaatggatggcccaaaggtt

aaacaatggccattgacaaaagagaaaatagaagcattaacagcaatttgtgatgaaatg

gaaaaagaaggaaaaattacaaaaattgggcctgaaaatccatacaacactccaatattt

gccataaaaaagaaagacagtactaagtggagaaaattagtagatttcagggaactcaat

aaaagaactcaagatttttgggaagttcaattaggaataccacacccagcaggattaaaa

aagaaaaaatcagtgacagtgctggatgtgggggatgcatatttttcagttcctttayat

gaagacttcaggaaatatactgcattcaccatacctagtataaacaatgaaacaccaggg

attaggtatcagtayaatgtacttccacagggatggaaaggatcaccagcaatatttcaa

agtagcatgacaaaaatcttagaaccttttagaraacaaaatccagacatagtcatctat

caatacatggatgatttgtatgtaggatctgacttagagatagggcagcatagaacaaaa

atagaaraactgagacaacatttgttgagatggggatttaccacaccagacaagaaacat

cagaaagaacctccatttctttggatggggtatgaactccatcctgacaaatggacagta

cagcctatacagctgccagtacaagatagctggactgtcaatgatatacaaaagttagtg

ggaaaattaaaytgggcaagtcagatttatcctggaattaaagtaaggcaactttgtaaa

ctccttagggggaccaaagcactaacagacatagtaccactaactgaagaagcagaa---

------------------------------------------------

>CRF07BC.42-SX2012089

cctcaaatcactctttggcaacgaccccttgttacaataaagataggggggcaattaaag

gaagctttattagatacaggagcagacgatacagtattagaagacatgaatttgccagga

aaatggaaaccaaaaatgatagggggaattggaggttttatcaaagtaaracagtatgaa

gagatacccatagaaatctgtggacataaagctataggtacagtattagtaggacctaca

cctatcaacataattggaaggaatctgttgactcagcttggttgtactttaaattttcca

atcagtcctattgatactgtaccagtaaaactaaagccaggaatggatggcccaaaggtt

aaacaatggccattgacaaaagagaaaatagaagcattaacagcaatttgtgatgaaatg

gaaaaggaaggaaaaattacaaaaattgggcctgaaaatccatacaacactccaatattt

gccataaaaaagaaagayagtactaagtggagaaaattagtagatttcagggaactcaat

aaaagaactcaagatttttgggaagttcaattaggaataccacacccagcaggattaaaa

aagaaaaaatcagtgacagtgctggatgtgggagaygcatatttttcagttcctttagat

gaagayttcaggaaatatactgcattcaccatacctagtataaacaatgaaacaccaggg

attmggtatcagtacaatgtacttccacagggatggaaaggatcaccagcaatatttcaa

agtagcatgacaaaaatcttagagccttttagaaaacaaaatccagacatagtcatytat

caatacatggatgatttgtatgtaggatctgacttagarataaagcagcatagaacaaaa

atagaggaactgagacaacatttgttgaggtggggatttaccacaccagacaagaaacat

cagaaagaacctccatttctttggatggggtatgaactccaccctgacaaatggacagta

cagcctatacagctgccagtacaagatagctggacggtcaatgatatacaaaagttagtg

ggaaaattaaactgggcaagtcagatttatcctggaattaaaacaaggcaactttgtaaa

ctccttagggggaccaaagcactaacagacatagtaccactaactgaagaagcagaa---

------------------------------------------------

>CRF07BC.42-TT121132

cctcaaatcactctttggcaacgaccccttgttacaataaagataggggggcaattaaag

gaggctctaytagatacaggagcagatgatacagtattagaagayatgaatttgccaggg

aaatggaaaccaaaaatgatagggggaattggaggttttatcaaagtaagacagtatgaa

cagrtacccatagaaatctgtggacataaagctataggtacartattagtaggacctaca

cctgtcaacataattggaagaaatctgttgactcagcttggttgtactttaaattttcca

atcagtcctattgaaactgtaccagtaaaactaaagccaggaatggatggcccaaaggtt

aaacaatggccattgacaaaagagaaaatagaagcattaacagcaatttgtgatgaaatg

gaaaaggaaggaaaaattacaaaaattgggcctgaaaatccatacaatactccaatattt

gccataaaaaagaaagacagtactaagtggagaaaattagtagatttcagggaactcaat

aaaagaactcaagatttttgggaagttcaattaggaataccacacccagcaggattaaaa

aagaaaaaatcagtgacagtgctggatgtgggggatgcatatttttcagttcctttacat

gaagacttcaggaaatatactgcattcaccatacctagtacaaayaatgaaacaccaggr

attagrtatcagtacaatgtacttccacagggatggaaaggatcaccagcaatatttcaa

agtagcatgacaaaaatcttagagccttttagaaaacaaaatccagacatagtcatctat

caatacatggatgatttgtatgtaggatctgacttagagatagggcagcatagarcaaaa

atagaggaactgagacaacatytgttgaggtggggrtttaccacaccagacaagaaacat

cagaaagaacctccatttctttggatggggtatgarctccatcctgacaaatggacagta

cagcctatacagctgccagtacaagatagctggactgtcaatgatatacaaaagttagtg

ggaaaattaaactgggcaagtcagatttatcctggaattaaagtaaggcaactttgtaaa

ctccttaggggggccaaagcactaacagacatagtaccactaactgaagaagcaga----

------------------------------------------------

>CRF07BC.43-G-HZ130569

cctcaaatcactctttggcaacgaccccttgttgccataaagatagggggacaattaaag

gaagctctattagatacaggagcagatgatacagtattagaagacatgaatttgccaggg

aaatggaaaccaaaaatgatagggggaattggaggttttatcaaagtaagacaatatgaa

cagatacccatagaaatctgtggacataaagctataggtacagtattagtaggacctaca

cctgtcaacataattgggaggaatctgttgactcagcttggttgtactttaaattttcca

atwagtcctattgaaactgtaccagtaaaattaaagccaggaatggatggcccaaaggtt

aaacaatggccattgacaaaagagaaaatagaagcattaacagcaatttgtgatgaaatg

gaaaaggaaggaaaaattacaaarattgggcctgaaaatccatacaacactccaatattt

gccataaaaaagaaagacagtactaagtggagaaaattagtagatttcagggaactcaat

aaaagaactcaagatttttgggaagttcaattaggaataccacatccagcaggattaaaa

aagaaaaaatcagtgacagtgctggatgtgggagatgcatatttttcagttcctttatat

gaagacttcaggaaatatactgcattcaccataccwagtataaataatgaaacaccaggg

attagatatcagtacaatgtacttccacagggatggaaaggatcaccagcaatatttcaa

agtagcatgacaaaaatcttagagccttttagaaaacaaaatccagacatagtcatctat

caatacatggatgatttgtatgtaggatctgacttagagatagggcagcatagaacaaaa

atagargaactgagacaacatttgttgaggtggggatttaccacaccagacaagaaacat

cagaaagaacctccatttctttggatggggtatgaactccatcctgacaaatggacagta

cagcctatacagctgccagtacaagatagctggactgtcaatgatatacaaaagttrgtg

ggaaagttaaactgggcaagtcagatttatcctggaattaaagtaaggcaactttgtaaa

ctccttaggggggccaaagcactaacagacatagtaccactaactgaagaagcagaa---

------------------------------------------------

>CRF07BC.44-TT121251-cotig

cctcaaatcactctttggcaacgaccccttgtcacaataaagataggggggcaattaaag

gaagctctattagatacaggagcagatgatacagtattagaagacatgaatttgccaggg

aaatggaaaccaaaaatgatagggggaattggaggttttatcaaagtaagacagtatgaa

carrtacccatagaaatctgcggacacaaagctatrggtacagtattagtaggacctaca

cctgtcaacataattggaagaaatctgttgactcagcttggttgcactttaaattttcca

atcagtcccattgaaacygtaccagtaaaattaaagccaggaatggatggcccaaaggtt

aaacaatggccattgacagaagagaaaataaaagcmttaacggcaatttgtgatgaaatg

gagaaggaaggaaaaattacaaaaattgggcctgaaaatccatataacactccaatattt

gccataaaaaagaaggacagtactaagtggagaaaattagtagatttcagggaactcaat

aaaagaactcaagatttttgggaagttcaattaggaataccacacccagcagggttaaaa

aagaaaaaatcagtgacaggactggatgtgggagatgcctatttttcagttcctttacat

gaggacttcaggaaatatactgcattcaccatacctagtataaacaatgaaacaccaggg

attaggtatcagtacaatgtacttccacagggatggaaaggatcaccagcaatattccaa

agtagcatgacaaraatcttagagccttttagraaacaaaayccaggcatagttatctat

caatacatggatgatttgtatgtagggtctgacttagagatagggcagcatagaataaaa

rtagaggaactgagacaacatttgttgaggtggggatttaccacaccagacaagaaacat

cagaaagaacctccatttctttggatggggtatgaactccatcctgacaaatggacagtr

cagcctatacagctaccagaaaaagatagctggactgtcaatgatatacaaaagttagtg

ggaaaattaaaytgggcaagtcagatttatcctggaattaargtaaggcaactttgtaaa

ctccttaggggggccaaagcactaacagaaatagtaccactaacagaagaagc-------

------------------------------------------------

>CRF07BC.45-TT121225

cctcaaatcactctttggcaacgaccccttgtcacaataaagatagggggacagttaaag

gaagctctattagatacaggagcagatgatacagtattagaagacatgaatttgccaggg

aaatggaaaccaaaaatgatagggggaattggaggttttatcaaagtaagacagtatgaa

cagatacccatagaaatctgtggacataaagctataggtacagtattagtaggacctaca

cctgtcaacataattggaagaaatctgttgactcagcttggatgtactttaaatttccca

atcagtcctattgaaactgtaccagtaaagttaaagccaggaatggatggcccaaaggtt

aaacaatggccattgaccaaagaaaaaatagaagcattaacagcaatctgtgaggaaatg

gaaaaagaaggaaaaattacaaaaattgggcctgaaaatccatacaacactccaatattt

gccataaaaaagaaagacagtactaagtggaggaaattagtagactttagggaactcaat

aaaagaactcaagacttttgggaagttcaattaggaataccacacccagcaggattaaaa

aagaaaaaatcagtgacagtgctggatgtgggggatgcatatttttcagttccgttatat

gaagacttcaggaaatatactgcattcaccatacctagtataaacaatgaaacaccaggg

attaggtatcagtacaatgtacttccacagggatggaaaggatcaccagcaatatttcaa

agcagcatgacaaaaatcttagagccttttagaaaacaaaatccagacatagtcatctat

caatacatggatgatttgtatgtaggatctgacttagagatagggcagcatagaacaaaa

atagaggaactgagacaacatttgttgaggtggggatttaccacaccagacaagaaacat

cagaaagaacccccatttctttggatggggtatgaactccatcctgataaatggacagta

cagcctatacagctgccagtacaagatagctggactgtcaatgatatacaaaagttagtg

ggaaaattaaactgggcaagtcagatctaccctggaattaaagtaaggcaactttgtaaa

ctccttagggggcccaaggcactaacagacatagtaccactaactgaagaagcag-----

------------------------------------------------

>CRF07BC.46-G-HZ130572

cctcaaatcactctttggcaacgaccccttgtcacaataaagataggggggcaattaaag

gaagctctattagatacaggagcagatgatacagtattagaagacatgaatttgccaggg

aaatggaaaccaagaatgatagggggaattgggggttttatcaaagtaagacagtatgaa

cagatacctatagaaatctgtggacataaagctgtaggtacagtattaataggacctaca

cctgtcaacataattggaaggaatctgttgactcagcttggttgtactttaaattttcca

atcagtcctattgaaactgtaccagtaaaactaaaaccaggaatggatggcccaaaggtt

aaacaatggccattgacaaaagagaaaatagaagcattaacagcaatttgtgatgaaatg

gaaaaggaaggaaaaattacaaaaattgggcctgaaaatccatacaacactccaatattt

gccataaaaaagaaagacagtactaagtggagaaaattagtagatttcagggaactcaat

aaaagaactcaagatttttgggaagttcaattaggaataccacacccagcrggattaaaa

aagaaaaaatcagtaacagtgctggatgtgggagatgcatatttttcagttcctttatat

gaagactttaggaaatatactgcattcaccatacctagtataaacaatgaaacaccaggg

attaggtatcagtacaatgtacttccacagggatggaaaggatcaccagcaatatttcaa

agtagcatgacaaaaatcttagagccttttagaaaacaaaacccagacatagtcatctat

caatacatggatgatttatatgtaggatctgacttagagatagggcagcatagagcgaaa

atagaggaactgagacaacatttgttgaggtggggatttaccacaccagacaagaaacat

cagaagcaacctccatttctttggatggggtatgaactccatcctgacaaatggacagta

cagcctatacagctgccagtacaagatagctggactgtcaatgatatacaaaagttagtg

ggaaaattaaattgggcaagtcagatttatcctggaattaaagtaaggcaactttgtaaa

ctccttagggggaccaaagcactaacagacatagtaccactaactgaagaagcagaat--

------------------------------------------------

>CRF07BC.46-HZ20140705

cctcagatcactctttggcagcgacccctcgtcacaataaagataggggggcaattaaag

gaagctctcttagatacaggagcagatgatacagtattagaagacatgaatttgccaggg

aagtggaaaccaaaaatgatagggggaattggaggttttatcaaagtaagacagtatgaa

cagatacccatagaaatctgtggacacaaagctataggtacagtattagtgggacctact

cctgtcaacataattggaagaaatctgttgactcagcttggttgcactttaaattttcca

atcagtcccattgaaactgtaccagtaaaattaaagccaggaatggatggcccaaaggtt

aaacaatggccattgacagaagagaaaataaaagcattaacagcaatttgtgaggaaatg

gaaaaggaagggaaaattacaaaaattgggcctgaaaatccatataacactccaatattt

gccataaaaaagaaggacagtactaagtggagaaaattagtagatttcagggaactcaat

aaaagaactcaagatttttgggaagttcaattaggaataccacacccagcaggattaaaa

aagaaaaaatcagtaacagtactggatgtgggggatgcatatttttcagttcctttatat

gaagacttcaggaagtatactgcattcaccatacctagtrtaaacaatgaaacaccaggg

attaggtatcagtacaatgtacttccacagggatggaaaggatcaccagcgatattccaa

agtagtatgacaaaaatcttagagccttttagaaaacaaaatccagatatagttatctat

cagtacatggatgatttgtatgtaggatctgacttagagatagggcagcatagaacaaaa

atagaggaactgagacaacatttattgagttggggatttaccacgccagacaagaaacat

cagaaagaacctccatttctttggatggggtatgaactccatcctgacaaatggacagta

cagcctatacagctgccagaaaaagatagctggactgtcaatgatatacaaaagttagtg

ggaaaattaaactgggcaagtcagatttatcctggaattaaagtaaggcaactttgtaaa

ctccttagggggaccaaagcactaacagacatagtagtactaactgaagaagcagaatta

gaa---------------------------------------------

>CRF07BC.47-5100993595

cctcaaatcactctttggcagcgacccctcgttacaataaagataggggggcaattaaag

gaagctctattagatacaggagcagatgatacagtattagaagacatgaatttgccaggg

aaatggaaaccaaaaatgatagggggaattggaggttttatcaaagtaagacagtatgaa

cagatacccatagaaatctgtggacataaagctataggtacagtattagtaggacctaca

cctgtcaacataattggaagaaatctgttgactcagcttggttgtactttaaattttcca

atcagtcctattgaaactgtaccagtaaaactaaagccaggaatggatggcccaaaggtt

aaacaatggccgttgacaaaagagaaaatagaagcattaacagcaatttgtgatgaaatg

gaaaaggaaggaaaaattacaaaaattgggcctgaaaatccatacaacactccaatattt

gccataaaaaagaaagacagtactaagtggagaaaattagtagatttcagggaactcaat

aaaagaactcaagatttttgggaagttcaattaggaataccacacccagcaggattaaaa

aagaaaaaatcagtgacagtgctggatgtgggggatgcatatttttcagttcctttatat

gaagacttcaggaaatatactgcattcaccatacctagtataaacaatgaaacaccaggg

attaggtatcagtacaatgtacttccacagggatggaaaggatcaccagcgatatttcaa

agtagtatgacaaaaatcttagagccttttagaaaacaaaatccagacatagtcatctat

caatacatggatgatttgtatgtaggatctgacttagagatagggcagcatagaacaaaa

atagaggaactgagacaacatttgttgaggtggggatttaccacaccagacaagaaacat

cagaaagaacctccatttctttggatggggtatgaactccatcctgacaaatggacagta

cagcctatacagctgccagtacaagagagctggactgtcaatgatatacaaaagttagtg

ggaaaattaaactgggcaagtcagatttatcctggaattaaagtaaggcaactttgtaaa

ctccttaggggggccaaagcactaacagacatagtaccactaactgaagaagcagaa---

------------------------------------------------

>CRF07BC.47-HZ20140709

cctcaaatcactctttggcaacgaccccttgttaccataaagataggggggcaattaaag

gaagctctactagatacaggagcagatgatacagtattagaagacatgaatttgccaggg

aaatggaaaccaaaaatgatagggggaattggaggttttatcaaagtaagacagtatgaa

caggtacccatagaaatctgtggacataaagctataggtacagtattagtaggacctaca

cctgtcaacataattggaagaaatctgttgactcagcttggttgtactttaaattttcca

atcagtcctattgaaactgtaccagtaaaactaaagccaggaatggatggcccaaaggtt

aaacaatggccattgacaaaagagaaaatagaagcattaacagcaatttgtgatgaaatg

gaaaaggaaggaaaaattacaaaaattgggcctgaaaatccatacaacactccaatattt

gccataaaaaagaaagacagcactaagtggagaaaattagtagatttcagggaactcaat

aaaagaactcaagatttttgggaagttcaattaggaataccacacccagcaggactaaaa

aagaaaaaatcagtgacagtgctggatgtgggggatgcatatttttcagttcctttatat

gaagatttcaggaaatatactgcattcaccatacctagtataaacaatgaaacaccaggg

attaggtatcagtacaatgtacttccacaaggatggaaaggatcaccagcaatatttcaa

agtagcatgataaaaatcttagagcctttcagaaaacaaaatccagacatagtcatctat

caatacatggatgatttgtatgtaggatctgacttagagatagggcagcatagaacaaaa

atagaggaactgagacaacacttgttgargtggggatttaccacaccagacaagaaacat

cagaaagaacctccatttctttggatggggtatgaactccatcctgacaaatggacagta

cagcctatacagctaccagtacaagatagctggactgtcaatgatatacaaaagttagtg

ggaaaattaaactgggcaagtcagatttatcctggaattaaagtaaggcaactttgtaaa

ctccttaggggggccaaagcactaacagacatagtaccactaactgaagaagcagaa---

------------------------------------------------

>CRF07BC.49-20120896

cctcaaatcactctttggcaacgaccccttgtcacaataaaaataggggggcaattaaag

gaagctctattagatacaggagcagatgatacagtattagaagacatggatttgccaggg

aaatggaaaccaaaaatgatagggggaattggaggttttatcaaagtaagacagtatgaa

cagatacccatagaaatctgtggacataaagctataggtacagtattagtaggacctaca

cctgtcaacataattggaagaaatttgttgactcagcttggttgtactttaaattttcca

ataagtcctattgaaactgtaccagtaaaactaaagccaggaatggatggcccaaaggtt

aaacaatggccattgacaaaagagaaaatagaagcattaacagcaatttgtgatgaaatg

gaaaaggaaggaaaaattacaaaaattgggcctgaaaatccatacaacactccaatattt

gccataaaaaagaaagacagtactaagtggagaaaattagtagatttcagggaactcaat

aaaagaactcaagatttttgggaagttcaattaggaataccacacccagcaggattaaaa

aagaaaaaatcagtgacagtgctggatgtgggggatgcatatttttcagttcctttatat

gaagacttcaggaaatatactgcattcaccatacctagtataaacaatgaaacaccaggg

attaggtatcagtacaatgtacttccacagggatggaaaggatcaccagcaatatttcaa

agtagcatgacaaaaatcttagagccttttagaaaacaaaatccagacataatcatctat

caatacatggatgatttgtatgtaggatctgacttagagatagggcagcatagaacaaaa

atagaggaactgcgacaacatttgttgaggtggggatttaccacaccagacaagaaacat

cagaaagaacctccatttctttggatggggtatgaactccatcctgacaaatggacagta

cagcctatacagctgccagtacaagatagctggactgtcaatgatatacaaaagttagtg

ggaaaattaaactgggcaagtcagatttatcctggaattaaagtaaggcaactttgcaaa

ctccttagggggaccaaagcactaacagacatagtaccactaactgaagaagcagaa---

------------------------------------------------

>CRF07BC.49-330111001-2012-00181

cctcaaatcactctttggcaacgaccccttgttaccataaagataggggggcaattaaag

gaagctctattagatacaggagcagatgatacagtattagaagacatgaatttgccaggg

aaatggaaaccaaaaatgatagggggaattggaggttttatcaaagtaagacagtatgaa

caaatacccatagaaatctgtggacataaagctataggtacagtattagtaggacctaca

cctgtcaacataattggaagaaatctgttgactcagcttggttgtactttaaattttcca

atcagtcctattgaaactgtaccagtaaaactaaagccaggaatggatggcccaaaggtt

aaacaatggccattgacaaaagagaaaatagaagcattaacagcaatttgtgatgaaatg

gaaaaggaaggaaaaattacaaaaattgggcctgaaaatccatacaacactccaatattt

gccataaaaaagaaagacagtactaagtggagaaaattagtagatttcagggagctcaat

aaaagaactcaagatttttgggaagttcaattaggaataccacacccagcaggattaaaa

aagaaaaaatcagtgacagtgctggatgtgggggatgcatatttttcagttcctttatat

gaagacttcaggaaatacactgcattcaccatacctagtacaaacaatgagacaccagga

atcaggtatcagtacaatgtgctaccacagggatggaaaggatctccggcaatattccag

tgtagcatgacaaaaatcttagagccctttagaagaaaaaatccagagataattatctat

caatacatggatgacttgtatataggatctgatttagaaatagggcaacacagaacaaaa

atagaggagctaagagctcatctattgagctggggatttaccacaccagacaagaaacat

cagaaagaacctccatttctttggatggggtatgaactccatcctgacaaatggacagta

cagcctatacagctgccagtacaagatagctggactgtcaatgatatacaaaagttggtg

ggaaaattaaactgggcaagtcagatttatcctggaattaaagtaaggcaactttgtaag

ctccttaggggggccaaagcactaacagacatagtaccactaactgaagaagca------

------------------------------------------------

>CRF07BC.49-HZ20140533

cctcaaatcactctttggcaacgaccccttgttaccataaagataggggagcaattaaag

gaggctctattagacacaggagcagatgatacagtattagaagacataaatttgccaggg

aaatggaaaccaagaatgatagggggaattggaggttttatcaaagtaagacagtatgaa

caggtacccatagaaatctgtggacataaggttataggtacagtattagtaggacctaca

cctgtcaacataattggaagaaatctgttgactcagcttggttgtactttaaattttcca

atcagtcctattgaaactgtaccagtaaaaytaaagccaggaatggatggcccaaaggtt

aracaatggccattgacaaaagagaaaatagaagcattaacagcaatttgtgatgaaatg

gaaaaggaaggaaaaattwcaaaaattgggcctgaaaatccatacaacactccaatattt

gctataaaaaagaaagacagtactaagtggagaaaattagtagatttcagggaactcaat

aaaagaactcaagatttttgggaagttcaattaggaataccacacccagcaggattaaaa

aagaagaaatcagtgacagtactggatgtgggggatgcatatttttcagtgcctttacat

gaagacttcaggaagtatactgcattcaccatacctagtacaaacaatgaaacaccaggg

rttaggtatcagtacaatgtacttccacagggatggaarggatcaccagcaatatttcaa

agtagcatgacaaaaatcttagagccttttagaaaacaaaatccagacatagtcatttat

caatacatggatgatttgtatgtaggatctgacttagagataggrcagcayagaacaaaa

atagaggaactaagacaacatttgttgaggtggggatttaccacaccagacaagaaacat

cagaaagaacctccatttctttggatggggtatgaactccatcctgacaagtggacagta

cagcctatacagctgccagtacaagatagctggactgttaatgatatacaaaagttagtg

ggaaaattaaactgggcaagtcagatttatcctggaattaaagtaagacaactttgtaga

ctccttaggggggccaaagcactaacagacatagtacca---------------------

------------------------------------------------

>CRF07BC.50-20120934

cctcaaatcactctttggcaacgaccccttgttccaataaagatagggggacagttaaag

gaagctctattagatacaggagcagatgatacagtattagaagacatgaatttgccaggg

aaatggaaaccaaaaatgatagggggaattggaggttttattaaagtaagacagtatgaa

cagatacccatagaaatctgtggacataaagttataggtacagtattaataggacctaca

cctgtcaacataattggaaggaatctgttgactcagcttggttgtactttaaattttcca

atcagtcctattgaaactgtaccagtaaaactaaagccaggaatggatggcccaaaggtt

aaacaatggccattgacaaaagagaaaatagaagctttaacagcaatttgtgatgaaatg

gaaaaggaaggaaaaattacaaaaattgggcctgaaaatccatacaacactccaatattt

gccataaaaaagaaagacagtactaagtggagaaaattagtagatttcagggaactcaat

aaaagaactcaagacttttgggaagttcaattaggaataccacacccagcaggattaaaa

aagaaaaaatcagtgacagtgctggatgtgggggatgcatatttttcagttcctttatat

gaagacttcaggaaatatactgcattcaccatacctagtataaacaatgaaacaccaggg

attaggtatcagtacaatgtacttccacagggatggaaaggatcaccagcaatatttcaa

agtagcatgacaaaaatcttagagccttttagaaaacaaaatccagacatagtcatctat

caatacatggatgatttgtatgtaggatctgacttagagatagggcagcatagaacaaaa

atagaggaactgagacaacatttgttgaagtggggatttaccacaccagacaagaaacat

cagaaagaacctccatttctttggatggggtatgaactccatcctgacaagtggacagta

cagcctatacagctgccagtacaagatagctggactgtcaatgatatacaaaagttagtg

ggaaaattaaactgggcaagtcagatttatcctggaattaaagtaaggcaactttgtaaa

ctccttaggggggccaaagcactaacagacatagtaccactaactgaagaagcagaa---

------------------------------------------------

>CRF07BC.50-G-HZ130416

cctcaaatcactctttggcaacgaccccttgtcacaataaagatagggggacaattaaag

gaagctctattagatacaggagcagatgatacagtgttagaagacatgaatttgccaggg

aaatggaaaccaaaaatgatagggggaattggaggttttatcaaagtaagacagtatgaa

caggtamtcatagaaatctrtggacataaagttataggtacagtattagtaggacctaca

cctgtcaacataattggaagaaatctgttgactcagcttggwtgtactttaaattttcca

atcagtcctattgaaactgtaccagtaaaattaaagccaggaatggatggcccaaaggtc

aaacaatggccattgacaaaagaaaaaatagaagcattaacagcaatttgtgaggaaatg

gaaaaagaaggaaaaattacaaaaattgggcctgaaaatccatacaacactccaatattt

gccataaaaaagaaagacagtactaagtggagaaaattagtagatttcagggaactcaat

aaaagaactcaagacttttgggaagttcaattaggaataccacacccagcaggattaaaa

aagaaaaaatcagtgacagtgctggatgtgggggatgcatatttttcagttcctttatat

gaagacttcaggaaatatactgcattcaccatacctagtataaacaatgaaacaccaggg

attaggtatcagtacaatgtacttccacaaggatggaaaggatcaccagcaatatttcaa

agtagcatgacaaaaatcttagagccttttagaaaacaaaatccagacatagtcatctat

caatacatggatgatttgtatgtaggatctgacttagagatagggcagcatagaacaaaa

atagaggaactgagacaacatttgttgaggtggggatttaccacaccagacaagaaacat

cagaaagaacctccatttctttggatggggtatgaactccatcctgacaaatggacagta

cagcctatacagctgccagtacaagatagctggactgtcaatgatatacaaaaattagtg

ggaaaattaaactgggcaagtcagatttatcctggaattaaagtaaggcaactttgtaaa

ctccttaggggggccaaagcactaacagacatagtacc-ctaactgaagaa---------

------------------------------------------------

>CRF07BC.5101827698

cctcaaatcactctttggcaacgaccccttgttaccataaagataggggggcaattaaag

gaagctctattagatacaggagcagatgatacagtattagaagaaatgaatttgccagga

aaatggaaaccaaaaatgatagggggaattggaggttttatcaaagtaagacagtatgaa

caggtrcccatagaaatttgtggacataaagctataggtacagtattaataggacctaca

cctgtcaacataattggaagaaatctgttgactcagcttggttgtactttaaattttcca

atcagtcctattaaaactgtaccagtaaaactaaagccaggaatggatggcccaagggtt

aaacaatggccattgacaaaagagaaaatagaagcattaacagcaatttgtgawgaaatg

gaaaaggaaggaaaaattacaaaaattgggcctgaaaatccatacaacactccaatattt

gctataaaaaagaaagacagtactaagtggagaaaattagtagatttcagggaactcaat

aaaagaactcaagatttttgggargttcaattaggaataccacacccagcaggattaaaa

aagaaaaaatcagtgacagtgctggatgtgggggatgcatatttttcagttcctttagat

gaagacttcaggaaatatactgcattcaccatacctagtataaacaatgaaacaccaggg

attaggtatcagtacaatgtactyccacagggatggaaaggatcaccagcaatatttcaa

agyagcatgacaaaaattttagagccttttagaaaacaaaatccagacatagtcatctat

caatacatggatgatttgtatgtaggatctgacttagagatagggcagcatagaataaaa

atagaggaactgagacaacatttgttgaggtggggatttactacaccagacaagaaacat

cagaaagaacctccatttytgtggatggggtatgaactccatcctgacaaatggacagta

cagcctatacagctgccaatacaagatagctggactgtcaatgatatacaaaagttagtg

ggaaaattaaactgggcaagtcaratttatcctggaattaaagtaaggcaactttgtaaa

ctccttagggggrccaaagcactaacagacatagtaccactaactgaagaagcagaatta

gaattggcaga-------------------------------------

>CRF07BC.52-20121017

cctcaaatcactctttggcaacgacccctcgtcccaataaagataggggggcaatcaaag

gaagctctattagatacaggagcagatgatacagtattagaagacatgaatttgccaggg

aaatggacaccaaaaatgatagggggaattggaggttttatcaaagtaagacaatatgaa

cagatacccatagaaatctgtggacacaaaactataggtacagtattagtaggacctaca

cctgtcaacataattggaagaaatctgttgactcagattggttgcactttaaattttcca

atcagtcccattgaaactgtaccagtaaaattaaagccaggaatggatggcccaaaggtt

aaacaatggccattgacagaagagaaaataaaagcattaacagaaatttgtaaagaaatg

gaaaaagaaggaaaaattacaaaaattgggcctgaaaatccatataacactccaatattt

gccataaaaaagaaggacagtactaagtggagaaaattagtagatttcagggaacttaat

aagagaactcaagatttttgggaagttcaattaggaataccacacccagcagggttaaag

aagaaaaagtcagtgacagtactggatgtgggggatgcatatttttcagttcctttacat

gaagacttcaggaaatatactgcattcaccatacctagtagaaacaatgaaacaccagga

attaggtatcagtacaatgtacttccgcagggatggaaaggatcaccagcaatattccaa

actagcatgacaaaaatcttagaaccttttagaaaacaaaatccagacctagttatctat

caatacatggatgatttatatgtaggatctgacttagagatagggcagcatcgaacaaaa

atagargaactgagacaacatttgttgaggtggggatttaccacaccagacaagaaacac

caraaagaacctccatttctttggatggggtatgaactccatcctgacaaatggacagta

cagcctatacatctaccagaaaaagatagctggactgtcaatgatatacaaaagttagtg

ggaaaattaaactgggcaagtcagatttatcctggaattaaagtaagacaactttgtaaa

ctccttaggggggccaaagcactaacagacatagtaccactaactgaagaagcagaa---

------------------------------------------------

>CRF07BC.52-5101037281

cctcaaatcactctttggcaacgaccccttgttaccataaagataggggggcaattaaag

gaagctctactagatacaggagcagatgatacagtattagaagacatgaatttgccaggg

aaatggaaaccaaaaatgatagggggaattggaggttttatcaaagtaagacagtatgaa

cagatacccatagaaatctgtggacataaagctataggtacagtattagtaggacctaca

cctgtcaacataattggaagaaatctgttgactcagcttggttgtactttaaattttcca

atcagtcctattgaaactgtaccagtaaaactaaagccaggaatggatggcccaaaggtt

aaacaatggccattaacaaaagagaaaatagaagcattaacagcaatttgtgatgaaatg

gaaaaggaaggaaaaattacaaaaattgggcctgaaaatccatacaacactccaatattt

gccataaaaaagaaagacagtactaagtggagaaaattagtagatttcagggaactcaat

aaaaraactcaagatttttgggaagttcaattaggaataccacacccagcaggactaaaa

aagaaaaaatcagtgacagtgctggatgtgggggatgcatatttttcagttcctttatat

gaagacttcaggaaatatactgcattcaccatacctagtataaacaatgaaacaccaggg

attaggtatcagtacaatgtacttccacagggatggaaaggatcaccagcaatatttcaa

agtagtatgataaaaatcttagagccttttagaaaacaaaatccagacatagtcatctat

caatacatggatgatttgtatgtaggatctgacttagagatagggcagcatagaacaaaa

atagaggaactgagacaacatttgttgaggtggggatttaccacaccagacaagaaacat

cagaaagaacctccatttctttggatggggtatgaactccatcctgacaaatggacagta

cagcctatacagctaccagtacaagatagctggactgtcaatgatatacaaaagttagtg

ggaaaattaaactgggcaagtcagatttatcctggaattaaagtaaggcaactttgtaaa

ctccttaggggggccaaagcactaacagacatagtaccactaactgaagaagc-------

------------------------------------------------

>CRF07BC.53-5101095431

cctcaaatcactctttggcaacgaccccttgttaccataaagataggggggcaatcaaaa

gaggctctattagatacaggagcagatgatacagtattagaagacatgaatttgccaggg

aaatggaaaccaaaaatgatagggggaattggaggttttatcaaagtaagacartatgaa

cagatacccatagaaatctgyggacataaagctataggtacagtattagtaggacctaca

cctgtcaacataattggaagaaatctgttgactcagcttggttgtactttaaattttcca

atcagtcctatcgaaactgtaccagtaaaactaaagccaggaatggatggcccaaaggtt

aaacaatggccattgacaaaagagaaaatagaagcattaacagcaatttgtgctgaaatg

gaaaaggaaggaaaaattacaaaaattgggcctgaaaatccatacaacactccaatattt

gccataaaaaagaaagacagtactaagtggagaaaattagtagatttcagggaactcaat

aaaaaaactcaagatttttgggaggttcaattaggaataccacacccagcaggattaaaa

aagaaaaaatcagtgacagtgctggatgtgggggatgcatatttttcagttcctctatat

gaagacttcaggaaatatactgcattcaccatacctagtataaacaatgaaacaccaggr

attaggtatcagtacaatgtacttccacagggatggaaaggatcaccagcaatattycaa

agtagcatgacaaaaatcttagagccttttagaaaacaaaatccagacatagtcatctat

caatacatggatgatttgtatgtaggatctgacttagagatagggcagcatagaacaaaa

atagaggaactgagacaacatttgttgaggtggggattcaccacaccagacaagaaacat

cagaaagaacctccatttctttggatggggtatgaactccatcctgacaaatggacagta

cagcctatacagctgccagtacaagatagctggactgtcaatgatatacaaaagttagtg

ggaaaattaaactgggcaagtcagatttatcctggaattaaggtaaggcaactttgtaaa

ctccttaggggagccaaagcactaacagacatagtac-----------------------

------------------------------------------------

>CRF07BC.53-TT121339

cctcaaatcactctttggcaacgacccctcgtcacaataaaagtaggggggcaattaagg

gaggctctattagatacaggagcagatgatacagtattagaagacatgaatttaccaggg

aaatggaaaccaaaaatgatagggggaattggaggttttatcaaagtaagacaatatgaa

cagatacccatagaaatctgcggacacaaagctataggtacagtattagtaggacctaca

cctgtcaacataattggaagaaatctgttgactcaaattggttgcactttaaactttcca

atcagtcccattgaaactgtaccagtaaaattaaagccaggaatggatggcccaaaggtt

aaacaatggccattgacagaagagaaaataaaagcattaacagcaatttgtgatgaaatg

gagaaagaaggaaaaattacaaaaattgggcctgaaaatccatataacactccaatattt

gccataaaaaagaaggatagtactaagtggagaaagttagtagatttcagggaactcaat

aaaagaacccaagatttttgggaagttcaattaggaataccacacccagcagggttaaaa

aagaaaaaatcagtgacagtactggatgtgggggatgcatatttttcagttcctttatat

gaagacttcaggaaatatactgcattcaccatacctagtacaaacaatgaaacaccaggg

attaggtatcagtataatgtacttccacagggatggaaaggatcacctgcaatattccaa

agtagcatgacaaaaatcttagagccttttagaaaacaaaatccagacatagttatctat

caatacatggatgatttgtatgtaggatctgacttagaaatagggcagcatagaacaaaa

atagaggaactgagacagcatttgttaaagtggggatttaccacaccagacaagaaacat

cagaaagaacctccatttctttggatggggtatgaactccatcctgacaaatggacagta

cagcctatacaactgccagaaaaggatagctggactgtcaatgatatacaaaagttagtg

ggaaaattaaactgggcaagtcagatttatcctggaattaaagtaaggcaactatgtaaa

cttcttagggggaccaaagcactaacagacatagtaccactaactgaagaagcagaatta

g-----------------------------------------------

>CRF07BC.56-5100803696

cctcaaatcactctttggcaacgacccctagtcacaataaagataggggggcaattaaag

gaagcyctattagatacaggagcagatgatacagtattagaagaaatgaatttgccaggg

aaatggagaccaaaaatgatagggggaattggaggttttatcaaagtaagacagtatgaa

caaatacccatagaaatttgyggacacaaagctataggtacagtgttagtgggacctaca

cctgtcaacataattggacgaaatctgttgactcagcttggttgcactttaaattttcca

atcagtcccattgaaactgtaccagtacaattaaagccaggaatggatggcccaaaggtt

aaacaatggccattgacagaagagaaaataaaagcattaacagaaatttgtgatgaaatg

gaaaaggaaggaaaaattacaaarattgggcctgagaatccatataacactccaatattt

gccataaaaaagaaagacagtactaagtggagaaaattagtagattttagggaactcaat

aaaagaactcaagatttttgggaagttcaattaggaataccacacccagcagggttaaaa

aagaaaaaatcagtgacagtaytggatgtgggggatgcatatttttcagttcctttatat

gaagaattcaggaaatatactgcattcaccatacctagtacaaacaatgagacaccaggg

attaggtatcagtacaatgtacttccacaaggatggaaaggatcaccagcaatattccaa

agtagcatgacaaaaatcytagagccctttagaaaacaaaatccagacatagttatctat

caatacatggatgatttgtatgtaggatctgacttagagataggacagcatagaacaaaa

atagaagaactgagacaacatttgttgaggtggggatttaccacaccagacaagaaacat

cagaaagaacctccattcctttggatggggtatgagctccatcctgacaaatggacggta

cagcctatccagctgccagaaaaggatagctggactgtcaatgatatacaaaagttagtg

ggaaaattaaactgggcaagtcagatttatcctggaattaaagtaaggcaactttgtaaa

ctccttaggggggtcaaagcactaacagacatagtaccactaactgaagaagc-------

------------------------------------------------

>CRF07BC.56-G-HZ130980

cctcagatcactctttggcaacgacccctcgtcacaataaagataggggggcaagtaaag

gaagctctattagatacaggagcagatgatacagtattagaagacatgaatttgccaggg

aaatggaaaccaaaaatgatagggggaattggaggttttatcaaagtaaracagtatgaa

cagatacccatagaaatatgtggacacaaagctataggtacagtattagtaggacctaca

cctgtcaacataattggaagaaatctgttgactcagcttggttgcackttaaattttcca

atcagtccyattraaactatcccagtaaaattaaagccaggaatggatggcccaaaggtt

aaacaatggccattgacagaagaraaaataaaagcgttaacagaaatttgtaatgatatg

gagaaggaaggaaaaattacaaaaattgggcctgaaaatccatataacactccaatattt

gccataaaaaagaaggacagtactaagtggagaaaattagtagatttcagrgaactcaat

aaaagaactcaggatttttgggaagttcaattaggaataccacacccagcagggttgaaa

aagaaaaaatcagtgacagtactggatgtgggggatgcatatttttcagttcctttasat

gaagacttcaggaaatatactgcattcaccatacctagtataaacaatgaaacaccaggg

attaggtatcagtacaatgtacttccacagggatggaaaggatcaccagcaatattccaa

tgtagcatgacaaaratcttagagccttttagaagaaaaaatccagayatagttatttat

caatacatggatgatttrtatgtaggatctgacttagagatagggcagcatagaacaaaa

gtagargaactgagacaacatttgttgarttggggatttaccaccccagacaagaaacat

cagaaagaacccccatttctttggatggggtatgaactccatcctgacaaatggacagta

cagcctatacagctgccagaaaaggatagctggactgtcaatgatatacaaaagttagtg

ggaaaattaaactgggcaagtcagatttatcctggaattaaagtaaagcaactttgtaaa

ctccttagggggaccaaagcactaacagacatagtaccactaactgaagaagcagaat--

------------------------------------------------

>CRF07BC.56-HZ20140691

cctcaaatcactctttggcaacgacccatcgtcacaataatgataggggggcagttaaag

gaagctctattagatacaggagcagatgatacagtattagaagacatgaatttgccaggg

aaatggaaaccaaaaatgatagggggaattggaggttttatcaaagtaagacagtatgaa

caggtacccatagaaatctgcggacacaaagttataggtacagtgttagtgggacctaca

cctgtcaacataattggaagaaatctgttgactcagcttggttgcactttaaattttcca

atcagtcccattgaaactgtaccagtaaaattaaagccaggaatggatggcccaaaggtt

aaacaatggccattgacagaagaraaaataaaagcattaatggaaatttgtaatgaaatg

garaaggaaggaaaaattacaaaaattgggcctgaaaatccatataacactccaatattt

gccataaaaaagaaggacagtactaagtggagaaagttagtggatttcagggaactcaat

aaaagaactcaagatttttgggaagttcaattaggaatcccacacccagcagggttaaaa

aagaaaaaatcagtgacagtactggatgtgggggatgcatatttttcagttcctttacat

gaagacttcaggaaatatactgcatttaccatacctagtataaacaatgaaacaccaggg

attaggtatcagtacaatgtacttccacagggatggaaaggatcaccagcaatattccaa

agtagcatgacaaaaatcttagagccttttagaaaacaaaatccagacatagttatctat

caatacatggatgatttgtatgtaggatctgacttagagatagggcagcatagaacaaaa

atagaggaactgagacaacatttgttgaagtggggatttaccacaccagacaaaaaacat

caaaaagaacctccatttctttggatggggtatgaactccatcctgacaaatggacagta

cagcctatacagctaccagaaaaagatagctggactgtcaatgatatacaaaagttagtg

ggaaaattaaactgggcaagtcagatttatcctggaattaaagtaagrcaactttgtaaa

ctccttaggggarccaaagcactaacagaagtagtaccactaactgaagaagcagaat--

------------------------------------------------

>CRF07BC.57-G-HZ130981

cctcagatcactctttggcarcgacccctcgtcacaataaagataggggggcaagtaaag

gaagctctattagatacaggagcagatgatacagtattagaagacatgaatttgccaggg

aaatggaaaccaaaaatgatagggggaattggaggttttatcaaagtaaaacagtatgaa

cagatacccatagaaatatgtggacacaaagctataggtacagtattaataggacctaca

cctgtcaacataattggaagaaatctgttgactcagcttggttgcactttaaattttcca

atcagtcccattaaaactatcccagtaaaattaaagccaggaatggatggcccaaaggtt

aaacaatggccattgacagaagagaaaataaaagcgctaacagaaatctgtaatgakatg

gagaaggaaggaaaaattacaaaaattgggcctgaaaatccatataacactccaatattt

gccataaaaaagaaggacagtactaagtggagaaaattagtagattttagggaactcaat

aaaagaactcaggatttttgggaagttcaattaggaataccacacccagcagggttgaaa

aagaaaaaatcagtgacagtactggatgtgggggatgcatatttttcagttcctttatat

gaagacttcaggaaatatactgcattcaccatacctagtataaacaatgaaacaccaggg

attaggtatcagtacaatgtacttccacagggatggaaaggatcaccagcaatattccaa

tgtagcatgacaaaaatcttagagccttttagaagaaaaaatccagacatagttatttat

caatacatggatgatttgtatgtaggatctgacttagagatagggcagcatagaacaaaa

gtagaggaactgagacaacatttgttgagttggggatttaccaccccagacaagaaacat

cagaaagaacccccatttctttggatggggtatgaactccatcctgacaaatggacagta

cagcctatacagctgccagaaaaggatagctggactgtcaatgatatacaaaagttagtg

ggaaaattaaactgggcaagtcagatttatcctggaattaaagtaaagcaactttgtaaa

ctccttargggggccaaagcactaacagacatagtaccactaactgaagaagcagaat--

------------------------------------------------

>CRF07BC.57-HZ20141042

cctcaaatcactctttggcaacgaccccttgtcacaataaagataggggggcaattaaag

gaagctctattagatacaggagcagatgatacagtattagaagacataaatttgccaggg

aaatggaaaccaagaatgatagggggaattggaggctttatcaaagtaagacagtatgaa

cagatacccgtagaaatctgtggacatcaagctataggcacagtattagtaggacctaca

cctgtcaacataattggcagaaatctgttgactcagcttggttgtactttaaattttcca

atcagtcctattgaaactgtaccagtaaaactaaagccaggaatggatggcccaaaggtt

aaacaatggccattgacaaaagagaaaatagaggcattaacagcaatttgtgatgaaatg

gaaaaggaaggaaaaattacaaaaattgggcctgaaaatccatacaacactccaatattt

gccataaaaaagaaagacagtactaagtggagaaaattagtagatttcagggaactcaat

aaaagaactcaagatttttgggaagttcaattaggaataccacacccagcaggattaaaa

aagaaaaaatcagtgacagtgctggatgtgggagatgcatatttttcagttcctttagat

gaagactttaggaaatatactgcattcaccatacctagtataaacaatgaaacaccaggg

attaggtatcagtacaatgtacttccacagggatggaaaggatcaccagcaatatttcaa

agtagcatgacaaaaatcttagaaccttttagaaaacaaaatccagacatagtcatctat

caatacatggatgatttgtatgtaggatctgatttagagatagggcagcatagaacaaaa

atagaggaactgagagaacatttgttgaggtggggatttaccacaccagacaagaaacat

cagaaagaacctccatttctttggatggggtatgaactccatcctgataaatggacagta

cagcctatacagttgccagtacaagatagctggacagtcaatgatatacaaaagttagtg

ggaaaattaaactgggcgagtcagatttatcctggaattaaagtaaggcaactttgtaaa

ctccttagggggaccaaagcactaacagacatagtaccactaactgaagaagcag-----

------------------------------------------------

>CRF07BC.58-G-HZ130982

cctcagatcactctttggcaacgacccctcgtcacaataaagataggggggcaattaaag

gaagctctattagatacaggagcagatgatacagtattagaagagatgaatttgccaggg

aaatggaaaccaaaaatgatagggggaattggaggctttatcaaagtaagacagtatgaa

cagatacccatagaaatctgcggacacaaagctataggtacagtattaataggacctaca

cctgtcaacataattggaagaaatctgttgactcagcttggttgcactttaaattttcca

atcagtcctattgaaactataccagtaaaattaaagccaggaatggatggcccaagggtt

aaacagtggccattgacagaagagaaaataaaagcattaacagcaatttgtgaggaaatg

gaaaaggaaggaaaaattacaaaaattgggcctgaaaatccatataacactccaatattt

gccataaaaaagaaggacagtactaagtggagaaaattagtagatttcagggagctcaat

aaaagractcaagatttttgggargttcaattaggaataccacacccagcagggttaaaa

aagaaaaaatcagtgacagtactggatgtgggggatgcatatttttcagttcccttacat

gaagatttyaggaagtatactgcattcaccatacccagtagaaacaatgaaacaccaggg

attaggtatcagtacaatgtacttccacagggatggaaaggatcaccagcaatattccaa

agtagcatgacaaaaatcttagagccttttagaaaacaaaatccagagatagttatttat

caatatatggatgatttgtatgtaggatctgacttagagatagggcagcatagaacaaaa

gtagaggaactgagacaacatttgttgaggtggggatttaccacaccagacaaaaaacat

caaaargaacctccatttctttggatggggtatgaactccatcctgacaaatggacagta

cagcctatacagctaccagaaaaagatagctggactgtcaatgatatacaaaagttagtg

ggaaaattaaactgggcaagtcagatttatcctggaattaaagtaaggcaactttgtaaa

ctccttagggggrccaaagcactaacagacatagtaccactaactgcagaagcagaa---

------------------------------------------------

>CRF07BC.58-TT121665

cctcaaatcactctttggcaacgaccccttgtcacagtaaaaatagaaggacagctgaaa

gaagctctattagatacaggagcagatgatacagtattagaagatataaaattgccagga

aaatggaaaccaaaaatgatagggggaattggaggttttatcaaggtaaggcaatatgat

cagatacttatagaaatttgtggaaaaagggctataggtacagtattagtaggacctaca

cctgtcaacataattggacgaaatatgttgactcagattggttgtactttaaatttccca

attagtcctattgaaactgtaccagtaaaactaaagccaggaatggatggcccaaaggtt

aaacaatggccattgacaaaagagaaaatagaagcattaacagcaatttgtgatgaaatg

gaaaaggaaggaaaaattacaaaaattgggcctgaaaatccatacaacactccaatattt

gccataaaaaagaaagacagtactaagtggagaaaattagtagatttcagggaactcaat

aaaagaactcaagatttttgggaagttcaattaggaataccacacccagcaggattaaaa

aagaaaaaatcagtgacagtgctggatgtgggggatgcatatttttcagttcctttatat

gaagacttcaggaaatatactgcattcaccatacctagtataaacaatgaaacaccaggg

attaggtatcagtacaatgtacttccacagggatggaaaggatcaccagcaatatttcaa

agtagcatgacaaaaatcttagagccttttagaaaacaaaatccagacatagtcatctat

caatacatggatgatttgtatgtaggatctgacttagagataggacagcatagaacaaaa

atagaggaactgagacaacatttgttgaggtggggatttaccacaccagacaagaaacat

cagaaagaacctccatttctttggatggggtatgaactccatcctgacaaatggacagta

cagcctatacagctgccagtacaagatagctggactgtcaatgatatacaaaagttagtg

ggaaaattaaactgggcaagtcagatttatcctggaattaaagtaaggcaactttgtaaa

ctccttagggggaccaaagcactaacagacatagtaccactaactgaagaagcagaatta

gaa---------------------------------------------

>CRF07BC.59-712042

cctcaaatcactctttggcaacgacccctcgtcacaataaaggtaggggggcaattaaag

gaagctctattagatacaggagcagatgatacagtattagaagacatgaatttgccaggg

aaatggaaaccaaaaatgatagggggaattggaggttttatcaaagtaagacagtatgaa

cagatacccatagaaatttgcggacacaaagctataggtacagtattagtaggacctaca

cctgtcaacataattggaagaaatctgttgactcagcttggttgtactttaaattttcca

atcagtcccattgaaactgtaccagtaaaattaaagccaggaatggatggcccaaaggtt

aaacaatggcccttgacagaagagaaaataaaagcattaacagcaatttgtgaggaaatg

gagaaggaaggaaaaattacaaaaattgggcctgaaaacccatataatactccaatattt

gcgataaaaaagaaggacagtacaaagtggagaaaattagtagatttcagggaactcaat

aaaagaactcaagatttttgggaagttcaattaggaataccacacccagcagggctaaaa

aagaaaaaatcagtgacagtactggatgtgggagatgcatatttttcagttcctttacat

gaagacttcaggaaatatactgcattcaccatacctagtacaaacaatgaaacaccaggg

actaggtatcagtacaatgtacttccacagggatggaaaggatcaccagcaatatttcaa

agtagcatgacaaaaattttagagccttttagaaaacaaaatccagacatagtcatctat

caatacatggatgatttgtatgtaggatctgacttagagatagggcagcatagaacaaaa

atagaggaactgagacaacatttgttgaggtggggatttaccacaccagacaagaaacat

cagaaagaacctccatttctttggatggggtatgaactccatcctgacaaatggacagta

cagcctatacagctgccagtacaagatagctggactgtcaatgatatacaaaagttagtg

ggaaaattaaactgggcaagtcagatttatcctggaattaaagtaaggcaactttgtaaa

ctccttaggggrgccaaagcactaacagacatagtaccactaactgaagaagcagaatta

g-----------------------------------------------

>CRF07BC.59-TT121956

cctcaaatcactctttggcaacgacccattgtcacaataaagataggggggcaattaaag

gaagctctattagatacaggagcagatgatacagtattagaagacatgaatttgccaggg

aaatggaaaccaaaaatgatagggggaattggaggttttatcaaagtaagacagtatgaa

cagatacccatagaaatctgtggacataaagttataggtacagtattagtaggacctaca

cctgtcaacataattggragaaatctgttgactcagcttaattgtactttaaattttcca

atcagtcctattgaaactgtaccagtaaaactaaagccaggaatggatggcccaaaggtt

aaacaatggccattgacaaaagagaaaatagaagcattaacagcaatttgtgatgagatg

gaaaaggaaggaaaaattacaagaattgggcctgaaaatccatacaacactccaatattt

gccataaaaaagaaagacagtactaagtggagaaaattagtagatttcagggaactcaat

aaaagaactcaagatttttgggaagttcaattaggaataccacacccagcaggattaaaa

aagaaaaaatcagtgacagtgctggatgtgggggatgcatatttttcagttcctttatat

gaagacttcaggaaatatactgcattcaccatacctagtataaacaatgaaacaccaggg

attaggtatcagtacaatgtacttccacagggatggaaaggatcaccagcaatatttcaa

agtagcatgacaaaaatcttagagccttttagaaaacaaaatccagacatagtcatctat

caatacatggatgatttgtatataggatctgacttagagatagggcagcatagaacaaaa

atagaggaactaagacaacatttgttgaagtggggatttaccacaccagacaagaaacat

cagaaagaacctccatttctttggatggggtatgaactccatcctgacaaatggacagta

cagcctatacagctgccagtacaagatagttggactgtcaatgatatacaaaagttagtg

ggaaaattaaactgggcaagtcagatttatcctggaattaaagtaaggcaactttgtaaa

ctccttaggggggccaaagcactaacagacatagtaccactaacggaagaagcagaatta

gaaattggcag-------------------------------------

>CRF07BC.6-G-HZ130873

cctcaaatcactctttggcaacgaccccttgtcacaataaagataggggggcaattaaag

gaagctctgttagatacaggagcagatgatacagtattagaagacatgaatttgccaggg

aaatggaaaccaaaaatgatagggggaattggaggttttatcaaagtaagacagtatgaa

cagatacccctagaaatctgcggacacaaagctataggtacagtgttagtaggacctaca

cctgtcaacataattggaagaaatctgttgactcagattggttgcactttaaattttcca

atcagtcctattgaaaccgtaccagtaaaattaaagccaggaatggatggcccaaaggta

aaacaatggccattgacagaagagaaaataaaagcattaacagcaatttgtgatgaaatg

gaaaaggaaggaaaaattacaaaaattgggcctgaaaatccatataacactccaatattt

gccataaaaaagaaggacagtactaagtggaggaaattagtagatttcagggaactcaat

aaaagaactcaagatttttgggaagttcaattaggaataccacacccagcagggttaaaa

aggaaaaaatcagtgacagtactggatgtgggggatgcctatttttcagttcctttatat

gaagacttcaggaaatatactgcattcaccatacctagcataaacaatgaaacaccaggg

attaggtatcagtacaatgtacttccacagggatggaaagggtcaccagcaatatttcaa

agtagcatgataaaaatcttagagccttttagaaaacaaaatccagacatagttatctat

caatacatggatgatttgtatataggatctgacttagagatagggcagcatagaacaaaa

atagaggagctgagacaacatttgttgagatggggatttaccacaccagataagaaacat

cagaaagaacctccatttctttggatggggtatgaactccatcctgacaaatggacagta

cagcctatacagctaccagaaaaagatagctggactgtcaatgatatacaaaagttagtg

ggaaaactaaactgggcaagtcagatttatcctggaattaaagtgaggcaactttgtaaa

ctccttaggggggccaaagcactaacagacatagtaccactaactgaagaagcagaatta

gaaattggcaga------------------------------------

>CRF07BC.60-20120093

cctcaaatcactctttggcaacgacccctagtcacaataaagataggrgggcaattaaar

gaagctctattagatacaggagcagatgatacagtattagaagatatgaatttrccaggg

aaatggaaaccaaaaatgatagggggaattggaggttttatcaaagtaagacagtatgaa

cagatacccatagaaatytgcggacacccagctataggtacagtattagtrggacctaca

cctgtcaacataataggaagaaatctgttgactcagcttggttgcactttaaattttcca

atcagtcccattgaaactgtaccagtaaaattaaagccaggaatggatggcccaaaggtt

aaacaatggccattgacagaagagaaaatacaagcattaatagcaatttgtaatgaaatg

gagaaggaaggaaaaattacaaaaattgggcctgaaaatccatataacactccaatattt

gccataaaaaagaaggacagtacgaagtggagaaaattagtagatttcagggaactcaat

aaaagaactcaagatttctgggaagttcaactaggaataccacacccagcagggttaaaa

aagaaaaaatcagtgacagtactggatgtgggggatgcatatttttcagttcctttatat

gaagatttcaggaaatatactgcattcactatacctagtagaaacaatgaaacaccaggg

ataaggtatcagtacaatgtacttccacagggatggaaaggatcaccagcaatatttcaa

agtagcatgacaaaaatcttagagccttttagaaaacaaaatccagacatagttatctat

caatayatggatgatttgtatgtaggatctgacttagagatagggcagcatagagcaaaa

atagaggaactgagacaacatttgttgaggtggggatttaccacaccagacaagaaacat

cagaaagaacctccatttctttggatggggtatgaactccatcctgacaaatggacagta

cagcctatacagctaccagaaaaagatagctggactgtcaatgatatacaaaagttagtg

ggaaaattaaactgggcaagtcagatttatcctggaattaaagtaaggcaactttgtaaa

ctccttaggggggccaaagcactaacagacatagtaccactaactgaagaagcagaa---

------------------------------------------------

>CRF07BC.62-5100289329

cctcaaatcactctttggcaacgaccccttgttaccataaagataggggggcaattaaag

gaagctctattagatacaggagcagatgatacagtattagaagacatgaatttgccaggg

aaatggaaaccaaaaatgatagggggaattggaggttttattaaagtaagacagtatgaa

cagatacccatagaaatctgtggacataaagctataggtacagtattaataggacctaca

cctgtcaacataattggaagaaatctgttgactcagcttgggtgtactttaaattttcca

atcagtcctattgaaactgtaccagtaaaactaaagccaggaatggatggcccaaaggtt

aaacaatggccattgacaaaagagaaaatagaagcattaacagcaatttgtgatgaaatg

gaaaaggaaggaaaaattacaaaaattgggcctgaaaatccatacaacactccaatattt

gccataaaaaagaaagacagtactaagtggagaaaattagtagatttcagggaactcaat

aaaagaactcaagatttttgggaagttcaattaggaataccacacccagcaggattaaaa

aagaaaaaatcagtgacagtgctggatgtgggggatgcatatttttcagttcctttatat

gaagacttcaggaaatatactgcattcaccatacctagtataaacaatgaaacaccaggg

attaggtatcagtacaatgtacttccacarggatggaaaggatcaccagcaatatttcaa

agtagcatgacaaaratcttagaaccttttagaaaacaaaatccagacatagtcrtctat

caatacatggatgatttgtatgtaggatctgacttagagatagggcagcatagaacaaaa

atagaaaaactgagacaacatttgttgagatggggatttaccacaccagacaagaaacat

cagaaagaaccyccatttctttggatggggtatgaactccatcctgacaaatggacagta

cagcctatacagctgccagtacaagatagctggactgtcaatgatatacaaaagttagtg

ggaaaattaaactgggcaagtcagatttatcctggrattaaagtaaggcaactttgtaaa

ctccttaggggggccaaagcactaacagacatagtaccactaactgaagaagcagaatta

g-----------------------------------------------

>CRF07BC.64-20120274

cctcaaatcactctttggcaacgacccctcgtcacaataaagataggggggcaattaaag

gaagctctattagatacaggagcagatgatacagtattagaagacatgaatttgccaggg

aaatggaaaccaaaaatgatagggggaattggaggttttatcaaagtaagacagtatgaa

caratacccatagaaatctgcggacacaaagctataggtacagtattagtaggacctaca

cctgtcaacataattggaagaaatctgttgactcagcttggttgtactttaaattttcca

atcagtcccattgaaactgtaccagtaaaattaaagccaggaatggatggcccaaaggtt

aaacaatggccattgacagaagagaaaataaaagcattaacagcaatttgtgatgaaatg

gagaaggaaggaaaaattacaaagattgggcctgaaaatccatataacactccaatattt

gccataaaaaagaaggacagtactaagtggagraaattagtagacttcagggaactcaat

aaragaactcaagatttttgggaagttcaattaggaataccacacccagcagggttaaaa

aagaaaaaatcagtgacagtactggatgtgggggatgcctatttttcagtwcctttatat

gaagatttcaggaaatatactgcattcaccatacctagtataaacaatgaaacaccaggg

attaggtatcagtacaatgtacttccacagggatggaaaggrtcaccagcaatcttccaa

agtagcatgacaaaaatcttagagccttttagaaaacaaaatccagacatagttatctat

caatacatggatgatttgtatgtaggatctgacttagagataggrcaacatagaacaaaa

atagaggagytaagacagcatttgttaaggtggggatttaccacaccagacaagaaacat

cagaaagaacctccatttctttggatggggtatgaactccatcctgacaagtggacagta

cagcctatacagctaccagaaaaagatagctggactgtcaatgatatacaaaagttagtg

ggaaaattaaaytgggcaagtcaaatttatcctggaatcaaagtaaggcaactttgtaaa

ctccttaggggggccaaagcactaacagacatagtaccactaaccgaagaagcagaatta

g-----------------------------------------------

>CRF07BC.64-G-HZ131314

cctcaaatcactctttggcaacgaccccttgtcacaataaagatagggggacaattaaag

gaagctctattagatacaggagcagatgatacagtattagaagacatgaatttgccaggg

aaatggaaaccaaaaatgatagggggaattggaggttttatcaaagtaagacagtatgaa

cagatacccatagaaatytgtggacataaagctataggtacagtattagtaggacctaca

cctgtcaacataattggaagaaatctgttgactcagcttggttgcactttaaatttccca

atyagtcctattgaaactgtaccagtaaaattaaagccaggaatggatggcccaaaggtt

aaacaatggccattgacaaaagagaaaatagaagcattaacagcaatttgtgaggaaatg

gaaaaagaaggaaaaattacaaaaattgggcccgaaaatccatacaacacyccaatattt

gccataaaaaagaaagatagtactaagtggagaaaattagtagatttcagggaactcaat

aaaagaactcaagacttttgggaagttcaattaggaataccacacccagcaggattaaaa

argaaaaaatcagtgacagtgctggatataggggatgcatatttttcagttcctttatat

gaagacttcaggaaatatactgcattcaccataccwagtacaaacaatgaaacaccaggg

attaggtatcagtacaatgtacttccacagggatggaaaggatcaccagcaatatttcaa

agtagcatgayaaaaatcttagagccttttagaaaacaaaatccaaacatagacatctat

caatacatggatgatttgtatgtaggatctgacttagagatagggcagcatagagcaaaa

atagaggaactgagacaacatttgttgaggtggggatttaccacaccagacaagaaacat

cagaaagaacctccatttctttggatggggtatgaactccatcctgacaaatggacagta

cagcctatacagctgccagtacaagatagctggactgtcaatgatatacaaaagttagtg

ggaaaattaaactgggcaagtcagatttatccyggaattaaagtaaggcaactttgtaaa

ctccttaggggggccaaggcactaacagacatagtaccactaactgaagaagcagaatta

------------------------------------------------

>CRF07BC.68-G-HZ131341

cctcaaatcactctttggcaacgacccctcgtcacaataaagataggggggcaattaaag

gaagccctattagatacaggagcagatgatacagtattagaagacataaatttgccagga

aaatggaaaccaaaaatgatagggggaattggaggttttatcaaagtaagacagtatgaa

cagatacccatagaaatctgcggacacaaagctataggtacagtattagtaggacctaca

cctgtcaacataattggaagaaatctgttgactcagcttgggtgtactttaaattttcca

atcagtcctattgaaactgtaccagtaaaattaaagccaggaatggatggcccaaaggtt

aaacagtggccattgacagaagagaagataaaagcattaacggcaatttgtgatgaaatg

gaaaaggaaggaaaaattacaaaaattgggcctgaaaatccatataacactccaatattt

gccataaaaaagaaggacagtactaartggagaaaactagtagatttcagggaactcaat

aaaagaactcaagatttttgggaagttcaactaggaataccacacccagcagggttaaaa

aagaaaaaatcagtgacagtactggatgtgggggatgcatatttttcagttcctttatat

gaagacttcaggaaatatactgcattcaccatacctagtataaacaatgaaacaccaggg

attaggtatcagtacaatgtacttccacagggatggaaaggatcaccagcaatattccaa

agtagcatgacaaagatcttagagccttttagaaaacaaaatccagacatagttatctat

cagtacatggatgatttgtatgtaggrtcagacttagagatagggcagcatagaacaaaa

atagargaactgagacatcatttgttgaagtggggatttaccacaccagacaagaaacat

cagaaagaacctccatttctttggatggggtatgaactccatcctgacaagtggacagta

cagcctataaagcttccagaaaaagatagctggactgtcaatgatatacaaaaattagtg

ggaaaattaaactgggcaagycagatctatcctggaattaaagtaaggcaactttgtaaa

ctccttaggggggtcaaagcactaacagacatagtaccactaactgaagaagcagaatta

g-----------------------------------------------

>CRF07BC.69-12ZJ0653

cctcaaatcactctttggcaacgaccccttgtcacaataaaaataggaggacagctaaaa

gaagctctattagatacaggagcagatgatacagtattagaagatataaatttgccagga

aaatggaaaccaaaaatgatagggggaattggaggttttatcaaagtaagacagtatgaa

cagatacccgtagaaatctgtggacataaagctataggtacagtattagtaggacctaca

cctgtcaacataattggaagaaatctgttgactcagcttggttgtactttaaattttcca

atcagtcctattgaaactgtaccagtaaaactaaagccaggaatggatggcccaaaagtt

aaacaatggccattgacaaaagagaaaatagaagcattaacagcaatttgtgatgaaatg

gaaaaggaaggaaaaattacaaaaattgggcctgaaaacccatacaacactccaatattt

gccataaaaaagaaagacagtactaagtggagaaaattagtagattttagggaactcaat

aaaagaactcaagatttttgggaagttcaattaggaataccacacccagcaggattaaaa

agaaaaagatcagtgacagtgctggatgtgggggatgcatatttttcagttcctttagat

gaagacttcaggaaatatactgcattcaccatacctagtataaacaatgaaacaccaggg

attaggtatcagtacaatgtacttccacagggatggaaaggatcaccagcaatatttcaa

agtagcatgacaaaaatcttagagccttttagacagcaaaatccagacatagtaatctat

caatacatggatgatttgtatgtaggatctgacttagagatagggcagcatagaacgaaa

atagaggaactgagacaacatttgttgaagtggggatttaccacaccagacaagaaacat

cagaaagaacctccatttctttggatggggtatgaactccatcctgacaaatggacagta

cagcctatacagctgccagtacaagatagctggactgtcaatgatatacagaaattagtg

ggaaaactaaattgggcaagtcaaatttatccagggattaaggtaaagcaactgtgtaga

ctcctcaggggagctaaagcactaacagacatagtaccactgactgaagaagcagaatta

------------------------------------------------

>CRF07BC.7-TT120607

cctcaratcactctttggcaacgacccctcgtcacaataaagatagggggrcaagtaaag

gaagctctattagatacaggagcagatgatacagtattagaagacatgaatttgccagga

aaatggaaaccaaaaatgatagggggaattggaggttttatcaaagtaagacagtatgaa

gagatacccatagaaatttgcggacacaaarctataggtacagtattaataggacctaca

ccaatcaacataattggcagaaatctgttgactcagcttggttgcactttaaattttccc

atcagtcctattgaaactgtaccagtaaaattaaagccaggaatggatggcccaaaggtt

aaacaatggccattgacagaagaaaaaataaaagcattaacagaaatttgtaatgaaatg

gagaaggaaggaaaaattacaaaaattgggcctgagaatccatataacactccaatattt

gccataaaaaagaaggacagtactaagtggagaaaattagtagatttcagggaactcaat

aaaagaactcaagatttttgggaagttcaattaggaataccacacccagcagggttaaaa

aagaaaaaatcagtgacagtactagatgtgggggatgcatatttttcagttcctttatat

gaagacttcaggaaatacactgcattcaccatacctagtataaataatgaagcaccaggg

attaggtatcagtataatgtgcttccccagggatggaaaggatcaccagcaatatttcaa

agtagcatgacaaagatcttagatccttttagaaaacaaaatccagacatagttatctat

caatacatggatgatttgtatgtaggatctgacttagagatagggcagcatagaacaaaa

atagaggaactgagacaacatttgttgaggtggggatttaccacaccagacaagaaacac

cagaaagaacctccatttctgtggatggggtatgaactccatcctgataaatggacagta

cagcctatacagctgccagataaagacagctggactgtcaatgacatacaaaagttagtg

ggaaaattaaactgggcaagtcagatttatcctggaattaaagtaaggcaactttgtaaa

ctccttagggggaccaaagcactaacagacatagtaccactaactgaagaagcagaa---

------------------------------------------------

>CRF07BC.70-G-HZ131348

cctcaaatcactctttggcaacgaccccttgttaccataaaggtaggggggcaattaaag

gaagctctattagatacaggagcagatgatacagtattagaagacatgaatttgccaggg

aaatggaaaccaaaaatgatagggggaattggaggttttatcaaagtaagacagtatgat

cagatacccatagaaatctgtggacataaagctataggtacagtattagtaggacctaca

cctgtcaacataattggaagaaatctgttgactcagcttggttgtactttaaattttcca

atcagtcctattgaaactgtaccagtaaaactaaagccaggaatggatggcccaaaggtt

aaacaatggccattgacaaaagaaaaaatagaagcattaacagcaatttgtgatgaaatg

gaaaaggaaggaaaaattacaaaaattgggcctgaaaatccatacaacactccaatattt

gctataaaaaagaaagacagtactaagtggagaaaattagtagatttcagggaactcaat

aaaagaactcaagatttttgggaagtccaattaggaataccacacccagcaggattaaaa

aagaaaaaatcagtgacagtgctggatgtgggggatgcatatttttcagttcctctatat

gaagacttcaggaaatatactgcattcaccataccgagtataaacaatgaaacaccaggg

attaggtatcagtacaatgtacttccacagggatggaaaggatcaccagcaatatttcaa

aatagcatgacaaaaattttagagccttttagaaaacaaaatccagacatagtcatctat

caatacatggatgatttgtatgtaggatctgacttagagatagggcagcatagaataaaa

atagaggaactgagacaacatttgttgaggtggggatttactacaccagacaagaaacat

cagaaagaacctccatttctttggatggggtatgaactccatcctgacaaatggacagta

cagcctatacagctaccaatacaagatagctggactgtcaatgatatacaaaagttagtg

ggaaaattaaactgggcaagtcagatttatcc----------------------------

------------------------------------------------------------

------------------------------------------------

>CRF07BC.713204

cctcaaatcactctttggcaacgaccccttgtcacaataaagatagggggacaattaaag

gaagctctattagatacaggagcagatgatacagtattagaagacatgaatttgccaggg

aaatggaaaccaaaaatgatagggggaattggaggttttatcaaagtaagacagtatgaa

cagatacccatagaaatctgtggacataaagctataggtacagtattaataggacctaca

cctgtcaacataattggaagaaatttgttgactcagcttggttgtactttaaattttcca

atcagtcctattgaaactgtaccagtaaaattaaagccaggaatggatggcccaaaggtt

aaacaatggccattgacaaaagagaaaatagaagcattaacagcaatttgtgaggaaatg

gaaaaagaaggaaaaattacaaaaattgggcctgaaaatccatacaacactccaatattt

gccataaaaaagaaagacagtactaagtggagaaaattagtagatttcagggaactcaat

aaaagaactcaagacttttgggaagttcaattaggaataccacacccagcaggattaaaa

aagaaaaaatcagtgacagtgctggatgtgggggatgcatatttttcagttcctttatat

raagacttcaggaaatatactgcattcaccatacctagtataaacaatgaaacaccaggg

attaggtatcagtacaatgtacttccacagggatggaaaggatcaccagcaatatttcaa

agtagcatgacaaaaatcttagagccttttagaaaacaaaatccagacatagtcatctat

caatacatggatgatttgtatgtaggatctgacttagagatagggcagcatagarcaaaa

atagaggaactgagacaacatttgttgaggtggggatttaccacaccagataagaaacat

cagaaagaacctccatttctttggatggggtatgaactccatcctgacaaatggacagta

cagcctatacagctrccagtacaagatagctggactgtcaatgatatacaaaagttagtg

ggaaaattaaactgggcaagtcagatttatcctggaattaaaataargcaactttgtaaa

ctccttaggggg------------------------------------------------

------------------------------------------------

>CRF07BC.713318

cctcaaatcactctttggcaacgaccccttgtcacagtaaagataggggggcaattaaag

gaagctctattagatacaggagcagatgatacagtattagaagacatgaatttgccaggg

aaatggaaaccaaaaatgatagggggaattggaggttttatcaaagtaagacagtatgaa

cagatacccatagaaatctgtggacataaagctataggtactgtattagtaggacctaca

cctgtcaacataattggaagaaatttgttgacccagattggttgcactttaaattttcca

atcagtcctattgaaactataccagtaaaattaaagccaggaatggatggcccaaaggta

aaacaatggccattgacaaaagagaaaatagaagcattaacagcaatttgtgatgaaatg

gaaaaggaaggaaaaattacaaaaattgggcctgaaaatccatacaacactccaatattt

gccataaaaaagaaggacagtactaagtggagaaaattagtagatttcagggaactcaat

aaaagaactcaagatttctgggaagttcagttaggaataccacacccagcagggttaaaa

aagaaaaaatcagtgacagtactggatgtgggggatgcatatttttcagttcctttacat

gaagacttcaggaaatatactgcattcaccatacctagtacaaacaatgaaacaccaggg

attaggtatcagtacaatgtacttccacagggatggaaaggatcaccagcaatatttcaa

agtagcatgacaagaatcttagagccttttagaaaacaaaatccagacatagtcatctat

caatacatggatgatttgtatgtaggatctgacttagagatagggcagcatagaacaaaa

atagaggaactgagacaacatttgttgaggtggggatttaccacaccagacaagaaacac

cagaaagaacctccatttctttggatggggtatgaactccatcctgacaaatggacagta

cagcctatacagctaccagtacaagatagctggactgtcaatgatatacaaaagttagtg

ggaaaattaaactgggcaagtcagatttatcctggaattaaagtaaggcaactttgtaaa

ctccttaggggggccaaagcactaacagacatagtaccactaactgaagaagcagaatta

ga----------------------------------------------

>CRF07BC.713352

cctcagatcactctttggcaacgacccctcgtcacaataaagataggggggcaatcaaag

gaagctctattagatacaggagcagatgatacagtattagaagatatgaatttgccaggg

aaatggaaaccaaaaatgatagggggaattggaggttttatcaaagtaaracaatatgaa

cagatacccatagaaatttgtggacacaaagctataggtacagtattagtaggacctaca

cctgtcaacataattggaagaaatctgttgactcagcttggttgcactttaaattttcca

atcagtcctattgacactgtaccagtaaaattaaagccaggaatggatggcccaaaggtt

aaacaatggccattgacagaagagaaaataaaagcattaacggcaatttgtgaagaaatg

gagaaagaagggaaaattacaaaaattgggcctgaaaatccatataacactccaatattt

gctataaaaaagaaggacagtactaagtggagaaaattagtagatttcagggaactcaat

aaaagaactcaagatttttgggaagtccaattaggaataccacacccagcagggttaaaa

aagaaaaagtcagtgacagtactggatgtgggggatgcatatttttcagttcctttatat

gaagacttcaggaagtatactgcattcaccatacctagtacaaacaatgaaacaccagga

attaggtatcagtacaatgtacttccgcagggatggaaaggatcaccagcaatattccaa

aatagcatgacaaaaatcttagagccttttagaaaacagaatccagacatrgttatctat

caatacatggatgatttatatgtaggatctgacttagagataaggcagcatagaataaaa

atagaggaactgagacaacatttgttgaggtggggatttaccacaccagacaagaaacat

cagaaagaacctccatttctttggatggggtatgarctccaccctgacaaatggacagta

cagcctatacagctaccagaaaaagatagctggactgtcaatgatatacaaaagttagtg

ggaaaattaaactgggcaagtcagatttatcctggaattaaagtaagacaactttgtaaa

ctccttagggggaccaaagcactaacagacatagtgccactaactgaagaagcagaat--

------------------------------------------------

>CRF07BC.850

cctcaratcactctttggcaacgaccccttgttacaataaagataggggggcrattaaag

gaagcyctattagatacaggagcagatgatacagtattagaagacatgaatttrccaggr

aaatggaaaccaaaaatgatagggggaattggaggttttatcaaagtaagacagtatgaa

caggtayccatagaaatttgcgggcacaaagctataggtacagtattagtgggacctaca

cctatcaacataattggaagaaatytgttgactcagctwggttgcactttaaattttcca

atcagtcccattgaaactgtaccagtaaaattaaagccaggaatggatggyccaaaggtt

aaacaatggccattgacagaagaraaaataaaagcattaacagaaatttgtgatgaaatg

gagaaggaaggaaaaattacaaaaattgggcctgaaaacccatataacactccaatattt

gccataaaaaagaaggacrgtactaagtggagaaaattagtagatttcagggaactcaat

aaaagaactcaagatttttgggaagttcaattaggaataccacacccagcagggttaaaa

cagaacaaatcartgacagtactggatgtgggggatgcatatttttcagttcctttagat

gaagayttcagraaatatactgcattcaccatacctagtataaacaatgaaacaccaggg

attaggtatcagtacaatgtacttccacagggatggaaaggatcaccagcaatattccaa

agtagcatgacaaaaatcttggarccttttagaaaacaaaatccagacatagttatctat

caatacatggatgatttgtatgtaggatctgayttagagatagggcrgcatagaacaaaa

atagaggaactragacaacatttgttgaagtggggatttaccacaccagacaagaaacat

cagaaagaacctccattcctttggatggggtatgaactccatcctgacaaatggacagta

cagcctatacagctgccagaaaaagatagctggactgtcaatgatatacaaaagttagtg

ggaaaattaaactgggcaagtcagatttatcctggaattaaagtaaggcaactttgt---

------------------------------------------------------------

------------------------------------------------

>CRF07BC.9-TT120678

cctcaaatcactctttggcagcgaccccttgtcacaataaagataggggggcaattaaag

gaagctctattagatacaggagcagatgatacagtattagaagacatgaatttgccaggg

aaatggaaaccaaaaatgatagggggaattggaggttttatcaaagtaagacagtatgaa

gagatacccatagaaatttgcggacacaaagctataggtacagtattaataggacctaca

cctgtcaacataattggaagaaatctgttgactcagcttggttgcactttaaattttcca

atcagtcccattgaaactgtaccagtaaaattaaagccaggaatggatggcccaagggtt

aaacaatggccattgacagaagagaaaataaaagcattgacagaaatctgtaatgaaatg

gaaaaggaaggaaaaatttcaaaaattgggcctgaaaatccatataacactccaatattt

gccataaaaaagaaggacagtactaagtggagaaaactagtagatttcagggaactcaat

aaaagaactcaagatttttgggaagttcaattaggaataccacacccagcagggttgaaa

aagaaaaaatcagtgacagtactggatgtgggggatgcatatttttcagttcctttatat

gaagacttcaggaaatatactgcgttcaccatacctagtataaacaatgaaacaccaggg

attaggtatcagtacaatgtacttccacagggatggaaaggatcaccagcaatattccaa

agtagtatgacaaaaatcttagagccttttagaaaacaaaatccagacatagttatctat

caatacatggatgatttgtatgtaggatctgacttagagatagggcagcatagagcaaaa

atagaggaactgaggcaacatttgttgaagtggggatttaccacaccagacaagaaacat

cagaaagaacctccatttctttggatggggtatgaactccatcctgacaaatggacagta

cagcctatacggctgccagaaaaagatagctggactgtcaatgatatacaaaagttagtg

ggaaaattaaactgggcaagtcagatttatcctggaattaaagtaaggcaactttgtaaa

ctccttaggggggccaaagcactaacagacatagtgacactaactgaagaagcaga----

------------------------------------------------

>CRF07BC.CD4-141523

cctcaaatcactctttggcaacgaccccttgtcacaataaagataggggggcaattaaag

gaagctctattagatacaggagcagatgatacagtattagaagacatgaatttgccaggg

aaatggaaaccaaaaatgatagggggaattggaggttttatcaaagtaagacagtatgaa

cagatacccatagaaatctgtggacataaagctataggtacagtattrataggacctaca

cctgtcaacataattggaaggaatctgttgactcagcttggttgtactttaaattttcca

atcagtcctattgaaactgtaccagtaaaactaaagccaggaatggatggcccaaaggtt

aaacaatggccattaacaaaagaraaaatagaagcattaacrgcaatttgtgatgaaatg

gaaaaggaaggaaaaattacaaaaattgggcctgaaaatccctacaacactccaatattt

gccataaaaaagaaagacagtactaagtggagaaaattagtagatttcagggaactcaat

aaaagaactcaagatttttgggaagttcarttaggaataccacacccagcaggattaaaa

aagaaaagatcagtgacagtgctggatgtgggggatgcatatttttcagttcctttatat

gaagacttcaggaaatatactgcattcaccatacctagtataaayaatgaaacaccaggr

attaggtatcagtacaatgtacttccacagggatggaaaggatcaccagcaatatttcaa

agtagcatgacaaaaatcttagagccttttagaaaacaaaatccagacatagtcatctat

caatacatggatgatttgtatgtaggatctgacttagagatagggcaacatagagcaaaa

atagaggaactgagacaacatttgttgaggtggggatttaccacaccagacaagaaacat

cagaaagaacctccatttctttggatggggtatgaactccatcctgacaaatggacagta

caacctatacagctgccagtacaagatagctggactgtcaatgatatacaaaagttagtg

ggaaaattaaactgggcaagtcagatttatcctggaattaaagtaaggcaactttgtaaa

ctccttaggggggccaaagcactaacagacatagtaccactaactgaagaagcagaa---

------------------------------------------------

>CRF07BC.CD4-141581

cctcagatcactctttggcaacgaccccttgtcacaataaagataggggggcaattaaag

gaagctctattagatacaggagcagatgatacagtattagaagacatgaatttgccaggg

aaatggaaaccaaraatgatagggggaattgggggttttatcaaagtaagacagtatgaa

cagatacccatagaaatctgtggacataaagctgtaggtacagtattaataggacctaca

cctgtcaacataattggaaggaatctgttgactcagcttggttgtactttaaattttcca

atcagtcctattgaaactgtaccagtaaaactaaaaccaggaatggatggcccaaaggtt

aaacaatggccattgacaaaagagaaaatagaagcattaacagcaatttgtgatgaaatg

gaaaaggaaggaaaaattacaaaaattgggcctgaaaatccatacaacactccaatattt

gccataaaaaagaaagacagtactaagtggagaaaattagtagatttcagggaactcaat

aaaagaactcaagatttttgggaagttcaattaggaataccacacccagcaggattaaaa

aagaaaaaatcagtgacagtgctggatgtgggagatgcatatttttcagttcctttagat

aaagacttcaggaaatatactgcattcaccatacctagtataaacaatgaaacaccaggg

attaggtatcagtacaatgtacttccacagggatggaaaggatcaccagcaatatttcaa

agtagcatgacaaaaatcttagagccttttagaaaacaaaacccagacatagtcatctat

caatacatggatgatttatatgtaggatctgacttagagatagggcagcatagaacaaaa

atagaggaactgagacaacatttgttgaggtggggatttacaacaccagacaagaaacat

cagaaggaacctccatttctttggatggggtatgaactccatcctgacaaatggacagta

cagcctatacagctgccagtacaagatagctggactgtcaatgatatacaaaagttagtg

ggaaaattaaattgggcaagtcagatttatcctggaattaaagtaaggcaactttgtaaa

ctccttagggggaccaaagcactaacagacatagtaccactaactgaagaagcagaatta

gaa---------------------------------------------

>CRF07BC.CD4-142092

cctcaaatcactctttggcaacgaccccttgtyacaataaagataggagggcaaytaaag

gaagctctattagatacaggagcagatgatacagtattagaagacatgaatttgccaggg

aaatggaaaccaaaaatgataggaggaattggaggttttatcaaagtaagacagtatgaa

cagatacccatagaaatttgtggacataaagctataggtacagtattagtaggacctaca

cctgtcaacataattggaagaaatctgttgactcagcttggttgtactttaaattttcca

attagtcctattgaaactgtaccagtaaaactaaagccaggaatggatggcccaaaggtt

aaacaatggccattgacaaaagagaaaatagaagcattaacagcaatttgtgatgaaatg

gaaaaggaaggaaaaattacaaaaattgggcctgaaaatccatacaatactccaatattt

gccataaaaaagaaagacagyactaagtggagaaaattagtagatttcagggaactcaat

aaaagaactcaagatttttgggaagttcaattaggaataccacacccagcaggattaaaa

aagaaaaaatcagtgacagtgctggatgtgggggatgcatatttttcagttccgttatat

gaagacttcaggaaatatactgcattcaccatacctagtataaacaatgaaacaccaggg

attaggtatcagtacaatgtacttccacagggatggaaaggatcaccagcaatatttcaa

agtagcatgacaaaaatcttagagccttttagaaaacaaaatccagacatagtcatctat

caatacatggatgatttgtatgtaggatctgacttagagatagggcagcatagaacaaaa

atagaggaactgagacaacacttgttraggtggggattcaccacaccagacaagaaacat

cagaaagaaccgccatttctttggatggggtatgaactccatcctgacaaatggacagta

cagcctatacagctgccagtacaagatagctggactgtcaatgatatacaaaagttagtg

ggaaaattaaactgggcaagtcagatttatcctggaattaaagtaaggcaactttgtaaa

ctccttaggggggccaaagcactaacagacatagtaccactaactgaagaagcagaatta

g-----------------------------------------------

>CRF07BC.CD4-142137

cctcagatcactctttggcaacgaccccttgttaccataragataggggggcaattaaag

gaagctctattagatacaggagcagatgatacagtattagaagacatgaatttgccaggg

aaatggaaaccaaaaatgatagggggaattggaggttttatcaaagtaagacagtatgaa

cagatacccatagaaatctgtggacataaagctataggtacagtattagtaggacctaca

cctgtcaacataattggaagaaatctgttgactcagcttggttgtactttaaattttcca

atcagtcctattgaaactrtaccagtaaaactaaagccaggaatggatggcccaaaggtt

aarcaatggccattgacaaaagagaaaatagaagcattaacagcaatttgtgatgaaatg

gaaaaggaaggaaaaattacaaaaattgggcctgaaaatccatacaacactccaatattt

gccataaaaaagaaagacagtactaagtggagaaaattagtagatttcagggaactcaat

aaaagaactcaagatttttgggaagttcaattaggaataccacacccagcaggattaaaa

aagaaaaaatcagtgacagtgctggatgtgggggatgcatatttttcagttcctttatat

gaagacttcaggaaatatactgcattcaccatacctagtataaacaatgaaacaccaggg

attaggtatcagtacaatgtacttccacagggatggaaaggatcaccagcaatatttcaa

agtagcatgacaaaaatcttagagccttttagaaaacaaaatccagacatagtcatctat

caatacatggatgatttgtatgtaggatctgacttagagatagggcagcatagaacaaaa

atagaggaactgagacaacatttgttgaggtggggatttaccacaccagacaagaaacat

cagaaagaacctccatttctttggatggggtatgaactccatcctgacaaatggacagta

cagcctatacagctgccagtacaagatagctggactgtcaatgatatacaaaaattagtg

ggaaaattaaactgggcaagtcagatctatcctggaattaaagtaaggcaactttgtaaa

ctccttaggggggccaaagcactaacagacatagtgccactaactgaagaagcagaatta

aaa---------------------------------------------

>CRF07BC.CD4-142194

cctcaaatcactctttggcaacgaccccttgttaccataaagataggggggcaattaaag

gaagctctattagatacaggagcagatgatacagtattggaagacatgaatttgccaggg

aaatggaaaccaaaaatgataggaggaattggaggttttatcaaagtaaaacagtatgaa

cagatacccatagaaatctgtggacataaagctataggtacagtattagtaggacctaca

cctgtcaacataattggaagaaatttgttgactcagcttggttgtactttaaatttccca

atcagtcctattgaaactgtaccagtaaaactaaagccaggaatggatggcccaaaggtt

aaacaatggccattgacaaaagagaaaatagaagcattaacagcaatttgtgatgaaatg

gaaaaggaaggaaaaattacaaaaattgggcctgaaaatccatacaacactccaatattt

gccataaaaaagaaagacagtactaagtggagaaaattggtagattttagggaactcaat

aaaagaactcaagatttttgggaagttcaattaggaataccacacccagcaggattaaaa

aagaaaaaatcagtgacagtgctggatgtgggggatgcatatttttcagttcctttatat

gaagacttcaggaaatatactgcattcaccatacctagtataaacaatgaaacaccaggg

attaggtatcagtacaatgtacttccacagggatggaaaggatcaccagcaatatttcaa

agtagcatgacaaaaatcttagagccttttagaaaacaaaatccagacatagtcatctat

caatacatggatgatttgtatgtaggatctgacttagagatagggcagcatagaacaaaa

atagaggaactgagacaacatttgttgaggtggggatttaccacaccagacaagaaacat

cagaaagaacctccatttctttggatggggtatgaactccatcctgacaaatggacagta

cagcctatacacctgccagtacaagatagctggactgtcaatgatatacaaaagttagtg

ggaaaattaaactgggcaagtcagatttatcctggaattaaagtaagacaactttgtaaa

ctccttaggggggccaaagcgct-------------------------------------

------------------------------------------------

>CRF07BC.CD4-142520

cctcaaatcactctttggcaacgaccccttgttaccataaagataggggggcaattaaag

gaagctctattagatacaggrgcagatgatacagtattagaagamatgaatttgccaggg

aaatggaaaccaaaaatgatagggggaattggaggttttatcaaagtaagacagtatgar

sagatacccatagaaatctgtggacataaagctataggtacagtattaataggacctaca

cctgtcaacataattggaagaaatctgttgactcagcttggttgtactttaaattttcca

atcagtcctattgaaactgtaccagtaaaactaaagccaggaatggatggcccaaaggtt

aaacaatggccattaacaaaagagaaaatagaagcattaacagcaatttgtgatgaaatg

gaaaaggaaggaaarattacaaaaattgggcctgaaaatccatacaacactccaatattt

gccataaaaaagaaagacagtactaagtggagaaaattagtagatttcagggaactcaat

aaaagaactcaagatttttgggaagttcaattaggaataccacacccagcaggattaaaa

aagaaaaaatcagtgacagtgctggatgtgggggatgcatatttttcagttcctttagat

gaggacttcaggaaatatactgcattcaccatacctagtataaacaatgaaacaccaggg

atcaggtatcagtacaatgtacttccacagggatggaaaggatcaccagcaatattycaa

agtagcatgacaaaaatcttagagccttttagaaaacaaaatccagacatagtcatctat

caatacatggatgatttgtatgtaggatctgacttagagatagggcagcatagaacaaaa

atagaggaactgagacaacatttattgaggtggggatttaccacaccagacaagaaacat

cagaaagaacctccatttctttggatggggtatgagctccatcctgacaaatggacagta

cagcctatacagctgccagwacaagatagctggactgtcaatgatatacaaaagttagtg

ggaaaattaaactgggcaagtcagatttatcctggaattaaagtaaggcaactttgtaaa

ctccttaggggggccaargcactaacagacatagtaccactaactgaagaagcag-----

------------------------------------------------

>CRF07BC.CD4-142526

cctcaaatcactctttggcaacgaccccttgttccaataaagatagggggacagttaaaa

gaagctctattagatacaggagcagatgatacagtattagaagamatgaatttgccaggg

aaatggaaaccaaaaatgatagggggaattggaggttttatcaaagtaagacagtatgaa

cagatrcccatagaaatctrtggacataaagytataggtacagtattagtaggacctaca

cctgtcaacataattggaaggaatctgttgactcagcttggttgtactytaaattttcca

atcagtcctattgaaactgtaccagtaaaactaaagccaggaatggatggcccaaaggtt

aaacaatggccattgacaaaagagaaaatagaagcattaacagcaatttgtgatgaaatg

gaaaaggaaggaaaaattacaaaaattgggcctgaaaatccatacaacactccaatattt

gccataaaaaagaaagacagtactaagtggagaaaattagtagatttcagggaactcaat

aaaagaactcaagatttttgggaagttcaattaggaataccacacccagcaggattaaaa

aagaaaaaatcagtgacagtgctggatgtgggrgatgcatatttttcagttcctttakat

gaagacttcaggaaatatactgcattcaccatacctagtataaacaatgaaacaccaggg

attaggtatcagtacaatgtacttccacagggatggaaaggatcaccagcaatatttcaa

agtagcatgacaaaaatcttagagccttttagaaaacaaaatccagacatagtcatctat

caatacatggatgatttgtatgtaggatctgacttagagatagggcagcatagaacaaaa

atagaggaactgagacaacatttgtkgamgtggggatttaccacaccagacaagaaacat

cagaaagaacccccatttctttggatggggtatgaactccatcctgacaaatggacagta

cagcctatacagctgccagtrcaagatagctggactgtcaatgatatacaaaagttagtg

ggaaaattaaactgggcaagtcagatttatcctggaattaaagtaaggcaactttgtaaa

ctccttaggggggccaaagcactaacagacatagtaccactaactgaagaagcag-----

------------------------------------------------

>CRF07BC.CD4-142528

cctcaaatcactctttggcaacgaccccttgtcacaataaggataggggggcaattaaag

gaagctctattagatacaggagcagatgatacagtattagaagacatggatttgccaggg

aaatggaaaccaaaaatgatagggggaattggaggttttatcaaagtaagacagtatgaa

cagatacccatagaaatttgcggacacaaagccataggtacagtattagtaggacctaca

cctgtcaacataattggaagaaatctgytgactcagcttggttgcactttaaattttcca

atcagtcctattgaaactgtaccagtaaaattgaagccaggaatggatggcccaagggtt

aaacaatggccattgacagaagagaaaataaaagcattaacagcaatttgtgatgaaatg

gaaaaggaaggaaaaatttcaaaaattgggcctgaaaacccatataacactccaatattt

gccataaaaaagaaggatagtactaagtggagaaaattagtagatttcagggaactcaat

aaaagaactcaagatttttgggaagttcaattaggaataccacacccagcagggttaaaa

aagaaaaaatcagtgacagtattggatgtgggggatgcatatttttcagtacctctatat

gaagacttcaggaaatatactgcatttactatacctagtgtaaacaatggaacaccaggg

attaggtatcagtacaatgtacttccacagggatggaaaggatccccagcaatattccaa

agtagcatgacaaaaatcttggagccttttaggaaacaaaatccagacatagatatctat

caatacatggatgatctgtatgtaggatctgacttagagatagggcagcatagaacaaaa

atagaggaactgagacaacatttgttgaggtggggattgactacaccagacaagaaacat

cagaaagaacctccatttctttggatggggtatgaactccatcctgacaaatggaccgta

cagcctatacagctgccagaaaaagatagctggactgtcaatgacatacaaaagttagtg

ggaaaattaaactgggcaagtcagattt--------------------------------

------------------------------------------------------------

------------------------------------------------

>CRF07BC.CD4-142529

cctcaaatcactctttggcaacgaccccttgtcccaataaagataggggggcaattaaag

gaagctctattagatacaggagcagatgatacagtattagaagacatgaatttgccaggg

aaatggaagccaaaaatgatagggggaattggaggttttatcaaagtaagacagtatgaa

cagatacccatagaaatctgtggacataaagctataggtacagtattagtaggacctaca

cctgtcaacataattggaagaaatctgttaactcagcttggttgtactttaaattttcca

atcagtcctattgaaactgtaccagtaaaactaaagccaggaatggatggcccaaaggtt

aaacaatggccattaacaaaagagaaaatagaagcattaacagcaatttgtgtagaaatg

gaaaaggaaggaaaaattacaaaaattgggcctgaaaatccmtacaacactccaatattt

gccataaaaaagaaagacagtactaagtggagaaaattagtagatttcagggaactcaat

aaaagaactcaagatttttgggaagttcaattaggaataccacacccagcaggattaaaa

aagaaaaaatcagtaacagtgctggatgtgggggatgcatatttttcagtccctytatat

gaagacttcaggaaatatactgcattcaccatacctagtataaacaatgagacaccaggg

attaggtatcagtacaatgtacttccacagggatggaagggatcaccagcaatatttcaa

agtagcatgacaaaaatcttagagccttttagaaaacaaaatccagacatagtcatctat

caatacatggatgatttgtatgtaggatctgacttagagataggacagcatagaacaaaa

atagaggaactgagacaacatttgttgaggtggggatttaccacaccagacaagaaacat

cagaaagaacctccatttctttggatggggtatgaactccatcctgacaaatggacagta

cagcctatacagctgccagtacaagatagctggactgtcaatgatatacaaaagttagtg

ggaaaattaaactgggcaagtcagatttatcctggaattaaagtaaggcaactttgtaaa

ctccttaggggggccaaagcactaacagacatagtaccactaactg--------------

------------------------------------------------

>CRF07BC.CD4-142533

cctcaaatcactctttggcaacgaccccttgttaccataaagataggggggcagttaaag

gaagctctattagatacaggagcagatgatacagtattggaagacatgaatttgccaggg

aaatggaaaccaaaaatgataggaggaattggaggttttatcaaagtaaaacagtatgaa

cagatacccatagaaatctgtggacataaagctataggtacagtattagtaggacctaca

cctgtcaacataattggaagaaatctgttgactcagattggttgtactttaaattttcca

atcagtcctattgaaactgtaccagtaaaactaaagccaggaatggatggcccaaaggtt

aaacaatggccattgacaaaagagaaaatagaagcattaacagcaatttgtgaggaaatg

gaaaaggaaggaaaaattacaaaaattgggcctgaaaatccatacaacactccaatattt

gccataaaaaagaaagacagtacwaagtggagaaaattggtagattttagggaactcaat

aaaagaactcaagatttttgggaagttcaattaggaataccacacccagcaggattaaaa

aagaaaaaatcagtgacagtrctggatgtgggggatgcatatttttcagttcctttatat

gaagacttcaggaaatatactgcattcaccatacctagtataaacaatgaaacaccaggg

attaggtatcagtacaatgtacttccacagggatggaaaggatcaccagcaatatttcaa

agtagcatgacaaaaatcttagagccttttagaaaacaaaatccagacatagtcatctat

caatacatggatgatttgtatgtaggatctgacttagagatagggcagcatagaacaaaa

atagaggaactgagacaacatttgttgaggtggggatttaccacaccagacaagaaacat

cagaaagaacctccatttctttggatggggtatgaactccatcctgacaaatggacagta

cagcctatacacctgccagtacaagatagctggactgtcaatgatatacaaaagttagtg

ggaaaattaaactgggcaagtcagatttatcctggaattaaagtaaggcaactttgtaaa

ctccttaggggggccaaagcgctaacagacatagtaccactaactgaagaagcagaat--

------------------------------------------------

>CRF07BC.CD4-142536

cctcaaatcactctttggcagcgacccctagttaccataargataggggggcaawtaaag

gaagctctattagatacaggagcagatgatacagtattagaagacatggatttgccaggg

aaatggaaaccaaaaatgatagggggaattggaggttttatcaaagtaagacartatgaa

caaattcccatagaaatctgtgggcataaagctataggtacagtattagtaggacctaca

cctgtcaacataattggaagaaatctgttgactcagcttggttgtactttaaattttcca

atcagtcctattgaaactgtaccagtaaaactaaagccaggaatggatggcccaaaggtt

aaacaatggccattgacaaaagagaaaatagaagcattaacagcaatttgtgctgaaatg

gaaaaggaaggaaaaattacaaaaattgggcctgaaaatccatacaacactccaatattt

gccataaaaaagaaagacagtactaagtggagaaaattagtagatttcagggaactcaat

aaaagaactcaagatttttgggaagttcaattaggaataccacacccagcaggattaaaa

aagaaaaaatcagtgacagtgctggatgtgggggatgcatatttttcagttcctttatat

gaagacttcaggaaatatactgcattcaccatacctagtayraacartgaaacaccaggg

attaggtatcagtacaatgtacttccacagggatggaaaggatcaccagcaatatttcaa

agtagcatgacaaaaatcttagagcctttcagaaaacaaaatccagacatagtcatctat

caatacatggatgatttgtatgtaggatctgacttagagatagggcaacatagaacaaaa

atagaggaactgagacaacatttgttgaggtggggatttaccacacctgacaaaaaacat

cagaaagaacctccatttctttggatggggtatgaactccatcctgacaaatggacagta

cagcctatacaactgccagtacaagatagctggactgtcaatgatatacaaaaattagtg

ggaaaattaaactgggcaagtcagatttatcctggaattaaagtaaggcaactttgtaaa

ctccttagggggaccaaagcgttaacagacatagtaccacta------------------

------------------------------------------------

>CRF07BC.CD4-142537

cctcaaatcactctttggcaacgaccccttgttaccataaagataggggggcaattaaaa

gaagctctattagatacaggagcagatgatacagtattagaagacatgaatttgccaggg

aaatggaaaccaaaaatgatagggggaattggaggttttatcaaagtaagacagtatgaa

cagatacccatagaaatctgtggacataaagctataggtacagtattaataggacctaca

cctgttaacataattggaagaaatctgttaactcagcttggttgtactttaaattttcca

atcagtcctattgaaactgtaccagtaaaattaaagccaggaatggatggcccaaaggtt

aaacaatggccattgacaaaagagaaaatagaagcattaacagcaatctgtgatgaaatg

gaaaaggaaggaaaaattacaaaaattgggcctgaaaatccatacaacactccaatattt

gycataaagaagaaagatagtactaagtggagaaaactagtagatttcagggaactcaat

aaaagaactcaagatttttgggaagttcaattaggaataccacacccagcaggattaaaa

aggaaaaaatcagtgacagtgctggatgtgggggatgcatatttttctgttcctttatat

gaagacttcaggaaatatactgcattcaccatacctagtataaacaatgaaacaccaggg

attaggtatcagtacaatgtactcccacagggatggaaaggatcaccagcaatatttcaa

agtagcatgacaagaatcttagagccttttagaaaacaaaatccagacatagtcatctat

caatacatggatgatttgtatgtaggatctgacttagagatagggcagcatagaacaaaa

atagaggaactgagacaacatttgttgaagtggggatttaccacgccagacaagaaacat

cagaaagaacctccatttctttggatggggtatgaactccatcctgacaaatggacagta

cagcctatacagttgccagtacaagatagctggactgtcaatgatatacaaaagttagtg

ggaaaattaaactgggcaagtcaaatttatcctggaattaaagtaaggcaactttgcaaa

ctccttagggggaccaaagcactaacagacatagtaccactaactgaagaagcag-----

------------------------------------------------

>CRF07BC.CD4-142716

cctcaaatcactctttggcagcgaccccttgtcacaataaagataggggggcaattaaag

gaagctctattagatacaggagcagatgatacagtattagaagaaatgaatttaccaggg

aaatggaaaccaaaaatgatagggggaattggaggttttatcaaagtaagacagtatgaa

cagatacccatagaaatttgtggacacaaagctataggtacagtattaataggacctaca

cctgtcaacataattggaagaaatctgttgactcagcttgggtgcactttaaattttcca

atcagtcccattgaaactgtaccagtaaaattaaagccagaartggatggcccaagggtt

aaacaatggccattgacagaagagaaaataaaagcattaacagcaatttgtgatgaaatg

gagaaggaaggaaaaattacaaaaattgggcctgaaaatccatataacactccaatattt

gccataaaaaagaaagacagtactaagtggagaaaattagtagatttcagggagctcaat

aaaagaactcaagacttttgggaagttcaattaggaatacctcacccagcagggttaaaa

aagaaaaaatcagtaacagtactggatgtgggggatgcatatttttcagttcctttatat

gaagacttcaggaaatatactgcatttaccatacctagtataaacaatgaaacaccaggg

attaggtatcagtataatgtacttccacarggatggaaaggatcaccagcaatattccaa

agtagcatgacaaaaatcttagagccttttagaaaacaaaatccagacatagttatctat

caatacatggatgatttgtatgtaggatctgacttagagatagggcagcatagaacaaaa

atagaggaactgagagaatacctgttgaggtggggatttacyacaccagacaagaaacat

cagaaagaacctccatttctatggatggggtatgaactccatcctgacaaatggacagta

cagcccatacagctaccagaaaaagatagttggactgtcaatgatatacaaaagttagtg

ggaaaattaaactgggcaagtcagatttaccctggaattaaagtaaggcaactgtgtaaa

ctccttaggggagccaaagcactaacagaaatagtaccactaacggaagaagcagaatta

------------------------------------------------

>CRF07BC.CD4-142930

cctcaaatcactctttggcagcgacccctcgtcacaataaagataggggggcaattaaag

gaagctctattagatacaggagcagatgatacagtattagaagaaatgaatttgccaggg

agatggaaaccaaaaatgatagggggaattggaggttttatcaaagtaagacagtttgaa

cagatacccgtagaaatctgtggacacaaagctataggtacagtattagtaggacctaca

cctgtcaacataattggaagaaatctgttgactcagcttggttgtactttaaattttcca

atcagtcccattgaaactgtaccagtaaaattaaagccaggaatggatggcccaaaggtt

aaacaatggccattgacagaagagaaaataaaggcattaacagaaatttgtactgaaatg

gagaaggaaggaaagatcacaaaaattgggcctgaaaatccatataatactccaatattt

gccataaaaaagaaggacagtactaagtggagaaagttagtggatttcagggaacttaat

aaaagaactcaagacttttgggaagttcaattaggaataccacacccagcagggttaaaa

aagaaaaaatcagtgacagtactggatgtgggggatgcatatttttcagttcctttacat

gaagacttcaggaaatatactgcattcaccatacctagtataaacaatgaaacaccaggg

attaggtatcagtacaatgtgcttccacagggatggaaaggatcaccagcaatattccaa

agtagcatgacaaaaatcttagagccttttagaaaacaaaatccagacatagttatctat

caatacatggatgatttgtatgtaggatctgacttagagatagggcagcatagaataaaa

atagaggaactgaggcaacatttgttgaartggggattcaccacaccagacaaaaaacat

caaaaggagccgccattcctttggatggggtatgaactccatcctgacaaatggacagta

cagcctatacagctaccagaaaaagatagctggactgtcaatgatatacaaaagttagtg

ggaaaattaaactgggcaagtcagatttatcctggaattaaggtaaggcaactttgtaaa

cttcttaggggaaccaaagcactaacagaagtagtaccactaactgaagaagcagaatt-

------------------------------------------------

>CRF07BC.CD4-142935

cctcaaatcactctttggcaacgaccccttgttaccataaagatagggggacaattaaag

gaagctctattagatacaggagcagatgatacagtattagaagacatgaatttgccaggg

aaatggaaaccaaaaatgatagggggaattggaggttttatcaaagtaagacagtatgaa

cagatacccgtagaaatctgtggacataaagctataggtacagtattaataggrcctaca

cctgtcaacataattggaagaaatctgttgactcagcttggttgtactttaaattttcca

atcagtcctattgaaactgtaccagtaaaactaaagccaggaatggatggcccaaaggtt

aaacaatggccattgacaaaagagaaaatagaagcattaacagcaatttgtgatgaaatg

gaaaaggaaggaaaaattacaaaaattggacctgaaaatccatacaatacyccaatattt

gccataaaaaagaaagacagtactaagtggagaaaattagtagatttcagggaactcaat

aaaagaactcaagatttttgggaagttcaattaggaataccacacccagcaggattaaaa

aagaaaaaatcagtgacagtactggatgtgggggatgcatatttttcagttcctttatat

gaagacttcaggaaatatactgcattcaccatacctagtataaacaatgaaacaccagga

attaggtatcagtacaatgtacttccacagggatggaaaggatcaccagcaatatttcaa

agtagcatgacaaaaatcttagagccttttagaaaacaaaatccagaaatagtcatctat

caatacatggatgatttgtatgtaggatctgacttagagatagggcagcatagagcaaaa

atagaagaactgagacaacatttgttgagatggggacttaccacaccagacaagaaacat

cagaaagaacctccatttctttggatggggtatgagctccatcctgacaaatggacagta

cagcctatacagctgccagtacaagatagctggactgtcaatgatatacaaaagttagtg

ggaaaattaaactgggcaagtcagatttatcctggaattaaagtaaggcaactttgtaaa

ctccttaggggggccaaagcactaacagacatagtaccactaactgaagaagcaga----

------------------------------------------------

>CRF07BC.changliang

cctcaaatcactctttggcaacgaccccttgtcrcaataaaaataggaggacagctaaaa

gaagctctcttagatacaggagcagatgatacagtattagaagatataaatttgccagga

aaatggaaaccaaaaatgatagggggaattggaggttttatcaaagtaaggcaatatgat

cagatagctatagaaatttgtggaaaaagggctgtaggtacagtgttagtaggacctaca

cctgtcaacataattggacgaaatatgttgactcagattggttgyactttaaattttcca

atyagtcctattgamactataccagtaaaaytaaagccaggaatggatggcccaaaggtt

aaacaatggccattgacaaaagagaaaatagaagcattaacagcaatttgtgatgaaatg

gaaaaggaaggaaaaattacaaaaattgggcctgaaaatccatacaacactccaatattt

gccataaaaaagaaggacagtactaagtggagraaattagtagatttcagggaacttaat

aaaaggactcaagatttttgggaagttcaactaggaataccacacccagcagggttaaaa

aagaaaaaatcagtgacagtgctggatgtgggggatgcatatttttcagttcctttatat

gaagacttcaggaagtatactgcattcaccatacctagtataaacaatgaaacaccaggg

attaggtatcagtacaatgtacttccacaaggatggaaaggatcaccagcaatatttcaa

agtagcatgacaaaaatcttagagccttttagaaaacaaaatccagacatagtcatctat

caatacatggatgatttgtatgtaggatctgacttagagatagggcagcatagaacaaaa

gtagaggaactgagacaacatttgttggggtggggatttaccacaccagacaagaaacac

cagaaagaacctccatttctttggatggggtatgaactccatcctgacaaatggacagta

cagcctatacagctgccaatrcaagatagctggactgtcaatgatatacaaaaattagtg

ggaaaactaaattgggcaagtcaaatttatgcagggattaaggtaaggcaactgtgtaaa

ctcctcaggggagctaaagcattaacagacgtagtaccactgactgaa------------

------------------------------------------------

>CRF07BC.cn2013CD4-066

cctcaaatcactctttggcaacgaccccttgtcacaataaagatagggggacaattaaag

gaagctctattagatacaggagcagatgatacagtattagaagacatgaatttgccaggg

aaatggaaaccaaaaatgatagggggaattggaggttttatcaaagtaagacagtatgaa

cagatacccatagaaatctgtggacataaagctataggtacagtattagtaggacctaca

cctgtcaacataattggaagaaatctgttgactcagcttggttgtactttaaattttcca

atcagtcctattgaaactgtaccagyaaaactaaagccaggaatggatggcccaaaggtt

aaacaatggccattgacaaaagagaaaatagaagcattaacagcaatttgtgaggaaatg

gaaaaggaaggaaaaattacaaaaattgggcctgaaaatccatacaacactccaatattt

gccataaaaaagaaagacartactaagtggagaaaattagtagatttcagggaactcaat

aaaagaactcaagacttttgggaagttcaattaggaataccacayccagcaggattaaaa

aagaaaaaatcagtgacagtgctggatgtgggggatgcatatttttcagttcctttatat

gaagacttcaggaaatatactgcattcaccatacctagtataaacaatgaaacaccaggg

attaggtatcagtacaatgtacttccacaaggatggaaaggatcaccagcaatatttcaa

agtagcatgacaaaaatcttagagccttttagaaaacaaaatccagacatagtcatctat

caatacatggatgatttgtatgtaggatctgacttagagatagggcagcatagaacaaaa

atagaggaactgagagaacatttgttgaggtggggatttaccacaccagacaaaaaacat

cagaaagaacctccatttctttggatggggtatgaactccatcctgacaaatggacagta

cagcctatacagctgccagtacaagatagctggactgtcaatgatatacaaaaattggtg

ggaaaattaaactgggcaagtcagatttatcctggcattagagtaaggcaactttgtaaa

ctccttaggggggccaaagcactaacagacatagtacccctaactgaagaagcagaatta

r-----------------------------------------------

>CRF07BC.D2014-001

cctcaaatcactctttggcaacgaccccttgtcacagtaaagataggggggcaattaaag

gaagctctattagatacaggagcagatgatacagtattagaagacatgaatttgccagga

aaatggaaacc-aaaatgatagggggaattggaggttttatcaaagtaagacagtatgaa

cagatacccgtagaaatctgtggacataaagctataggtacagtgttagtaggacctaca

cctgtcaacataattggaaggaatctgttgactcagattggttgtactttaaactttcca

atcagtcctattgaaactgtaccagtaaaactaaagccaggaatggatggcccaaaggtt

aaacaatggccattgacaaaagaaaaaatagaagcattaacagcaatttgtgatgaaatg

gaaaaggaaggaaaaattacaaaaattgggcctgaaaatccatacaacactccaatattt

gccataaaaaagaaagacagtactaagtggagaaaattagtagatttcagggaactcaat

aaaagaactcaagatttttgggaagttcagttaggaataccacacccagcaggattaaaa

aagaagaaatcagtgacagtgctggatgtgggggatgcatatttttcagttcctttatat

gaagacttcaggaagtatactgcattcaccatacctagtacaaacaatgaaacaccaggg

attaggtatcagtacaatgtacttccacagggatggaaaggatcaccagcaatatttcaa

agtagcatgacaaaaatcttagagccttttagaaaacaaaatccagacatagtcatctat

caatacatggatgayttgtatgtaggatctgacttagagatagggcagcatagaacaaaa

atagaggaactgagacaacatttgttgaggtggggatttaccacaccagacaagaaacat

cagaaagaacctccatttctttggatggggtatgaactccatcctgacaaatggacagta

cagcctatacagctgccagtacaagatagctggactgtcaatgatatacaaaagttagtg

ggaaaactaaactgggcaagtcagatttatcctggaattaaagtaaggcaactttgtaaa

ctccttagggggaccaaagcactaacagacatagtaccactaactgaagaagcagaatta

g-----------------------------------------------

>CRF07BC.D2014-021

cctcaaatcactctttggcaacgaccccttgtcacaataaagatagggggacaattaaag

gaagctctattagatacaggagcagatgatacagtattagaagacatgaatttgccagga

aaatggaaaccaaaaatgatagggggaattggaggttttatcaaagtaagacagtatgaa

cagatacccgtagaaatctgtggacataaagctataggtacagtattagtaggacctaca

cctgtcaacataattggaagaaatctgttgactcagcttggttgcactttaaatttccca

atcagtcctattgaaactgtaccagtaaaattaaagccaggaatggatggcccaaaggtt

aaacaatggccattgacaaaagagaaaatagaagcattaacagcaatttgtgaggaaatg

gaaaaagaaggaaaaattacaaaaattgggcccgaaaatccatacaacactccaatattt

gccataaaaaagaaagacagtactaagtggagaaaattagtagatttcagggaactcaat

aaaagaactcaagacttttgggaagttcaattaggaataccacacccagcaggattaaaa

aagaaaaaatcagtgacagtgctggatgtgggggatgcatatttttcagttcctttatat

gaagacttcaggaaatatactgcattcaccatacctagtatraacaatgaaacaccaggg

attaggtatcagtacaatgtacttccacagggatggaaaggatcaccagcaatatttcaa

agtagcatgacaaaaatcttagagccttttagaaaacaaaatccaaacatagtcatctat

caatacatggatgatttgtatgtaggatctgacttagagatagggcagcatagaacaaaa

atagaggaactgagacaacatttgttgaggtggggatttaccacaccagacaagaaacat

cagaaagaacctccatttctttggatggggtatgaactccatcctgacaaatggacagta

cagcctatacagctgccagtacaagatagctggactgtcaatgatatacaaaagttagtg

ggaaaattaaactgggcaagtcagatttatcctggaattaaarcaaggcaactttgtaaa

ctccttaggggggccaaagcactaacagacatagtaccactaactgaagaagcagaatta

g-----------------------------------------------

>CRF07BC.D2014-025

cctcaaatcactctttggcaacgaccccttgtcccaataaaaataggggggcaattaaag

gaagctctattagatacaggagcagatgatacagtattagaagacatgaatttgccaggg

aaatggaaaccaaaaatgatagggggaattggaggttttatcaaagtaagacagtatgaa

cagatacccatagaaatctgtggacataaagctataggtacagtattagtrggacctaca

cctgtcaacataattggaagaaatctgttgactcagcttggttgcactttaaattttcca

atcagtcctattgaaactgtaccagtaaaattaaaaccaggaatggatggcccaaaggta

aaacaatggccattgacaaaagagaaaatagaagcattaacagcaatttgtgatgaaatg

gaaaaggaaggaaaaattacaaaaattgggcctgaaaatccatacaacactccaatattt

gccataaaaaagaaggacagtactaagtggagaaaattagtagatttcagggaactcaat

aaaagaactcaagatttctgggaagttcagttaggaataccacacccagcagggttaaaa

aagaaaaaatcagtgacagtactagatgtgggggatgcatatttttcagttcctttacat

gaagacttcaggaaatacactgcattcaccatacctagtgtaaacaatgaaacaccaggg

gttaggtatcagtacaatgtacttccacagggatggaaaggatcaccagcaatatttcaa

agtagcatgacaaaaatcttagagccttttagaaaacaaaatccaaacatagtcatctat

caatacatggatgatttgtatgtaggatctgacttagagatagggcagcatagaacaaaa

atagaggaactgagaaaacatttgttgaggtggggatttaccacaccagacaagaaacac

cagaaagaacccccatttctgtggatggggtatgaactccatcctgacaaatggacagta

cagcctatacagctgccagtacaagatagctggactgtcaatgatatacaaaagttagtg

ggaaaattaaactgggcaagtcagatttatcctggaattaaagtaaggcaactttgtaaa

ctcctta-----------------------------------------------------

------------------------------------------------

>CRF07BC.D2014-030

cctcaaatcactctttggcaacgaccccttgtcacagtaaagataggggggcaaataaag

gaagctctattagatacaggagcagatgatacagtattagaagacatgaatttgccaggg

aaatggaaaccaaaaatgatagggggaattggaggttttatcaaagtaagacagtatgaa

cagatacccatagaaatctgtggacataaagctataggtacagtattagtaggacctaca

cctgtcaacataattggaagaaatctgttgactcagcttggttgtaccttaaattttcca

atcagtcctattgaaactgtaccagtaaaactaaagccaggaatggatggcccaaaggtt

aaacaatggccattgacaaaagagaaaatagaagcattaacagcaatttgtgatgaaatg

gaaaaagaaggaaaaattacaaaaattgggcctgaaaatccatacaacactccaatattt

gccataaaaaagaaagacagtactaagtggagaaaattagtagatttcagggaactcaat

aaaagaactcaagatttttgggaagttcaattaggaataccacacccagcaggattaaaa

aggaaaagatcagtgacagtactggatgtgggagatgcatatttttcagttcccttatat

gaagacttcagaaaatatactgcattcaccatacctagtataaacaatgaaacaccaggg

attaggtatcagtacaatgtacttccacagggatggaaaggatcaccagcaatatttcaa

agtagcatgacaaagatcttagagccttttagaaaacaaaatccagacatagtcatctat

caatacatggatgatttgtatgtaggatctgacttagagatagggcagcatagaacaaaa

atagaggaactgagacaacatttgttgaggtggggatttaccacaccagacaagaaacat

cagaaagaacctccatttctttggatggggtatgaactccatcctgacaaatggacagta

cagcctatacagctgccagtacaagatagctggacggtcaatgatatacagaaattagtg

gggaaactaaattgggcaagtcaaatttacgcaggaattaagataaagcaactgtgtaaa

ctcctcaggggaactaaagcactaacagacatagtaccattgactgaagaagcagaatta

gaattggcaga-------------------------------------

>CRF07BC.D2014-036

cctcaaatcactctttggcaacgaccccttgtcacaataaagataggagggcaattaaag

gaagctttattagatacaggagcagatgatacagtattagaagacatgaatttgccagga

aaatggaaaccgaaaatgatagggggaattggaggttttatcaaagtaagacagtatgaa

cagatacccatagaaatctgtggacataaagctataggtacagtattagtaggacctaca

cctgtcaacataattggaagaaatctgttgactcagattggttgtactttaaattttccg

atcagtcctattgaaactgtaccagtaaaactaaagccaggaatggatggcccaaaggtt

aaacaatggccattgacaagagaaaaaatagaagcattaacagcaatttgtgatgaaatg

gaaaaggaaggaaaaattacaaaaattgggcctgaaaatccatacaacactccaatattt

gctataaaaaagaaagacagtactaagtggagaaaattagtagatttcagggaactcaat

aaaagaactcaagatttttgggaagttcaattaggaataccacacccagcaggattaaaa

aagaaaaaatcagtgacagtgctggatgtgggggatgcatatttttcagttcctttacat

gaagacttcaggaaatatactgcattcaccatacctagtataaacaatgaaacaccaggg

attaggtatcagtacaatgtacttccacagggatggaaaggatcaccagcaatatttcaa

agtagcatgacaaaaatcttagagccttttagaaaacaaaatccagacatagtcatctat

cagtacatggatgatttgtatgtaggatctgacttagagatagggcagcatagaacaaaa

atagaggaactgagacaacatttgttgagatggggatttaccacaccagacaagaaacat

cagaaagaacctccatttctttggatggggtatgagctccatcctgacaaatggaccgta

cagcctatacagctgccagtacaagatagctggactgtcaatgatatacaaaagttagtg

ggaaaattaaactgggcgagtcagatctatcctggaattaaagtaaggcaactttgtaaa

ctccttaggggggccaaaacactaacagacatagtaccactaactgaagaagcagaatta

gaaattggcag-------------------------------------

>CRF07BC.D2014-037

cctcaaatcactctttggcaacgaccccttgtcacaataaagataggggggcaattaaag

gaagctctattagatacaggagcagatgatacagtattagaagacatgaatttgccaggg

aaatggaaaccaaaaatgatagggggaattggaggttttatcaaagtaagacagtatgaa

cagatacccatagaaatctgtggacataaagctataggtacagtattagtaggacctaca

cctgtcaacataattggaagaaatctgttgactcagattggttgtactttaaattttcca

atcagtcctattgaaactgtaccagtaaaactaaagccaggaatggatggcccaaaggtt

aaacaatggccattgacaaaagagaaaatagaagcattaacagcaatttgtgatgaaatg

garaaggaaggaaaaattacaaaaattgggcctgaaaatccatacaacactccaatattt

gccataaaaaagaaagacagtactaagtggagaaaattagtagatttcagggarcttaat

aaaagaactcaagatttttgggaagtacaattaggaataccacacccagcaggattaaaa

aagaaaaaatcagtaacagtgctggatgtrggggatgcatatttctcagttcctttagat

aaagacttcaggaaatatactgcattcaccatacctagtataaacaatgaaacaccaggg

ataaggtatcagtacaatgtacttccacagggatggaaaggatcaccagcaatatttcaa

agtagtatgacaaaaatcttagagccttttagaaaacaaaatccagatatagtcatytat

caatacatggatgatttgtatgtaggatctgacttagagatagggcagcatagaacaaaa

atagaggaactgagagaacatttgttgaggtggggatttaccacaccagacaagaaacat

cagaaagaacctccatttctttggatggggtatgaactccatcctgacaaatggacagta

cagcctatacagttgccagtacaagatagctggactgtcaatgatatacaaaagttagtg

ggaaaattaaactgggcaagtcagatttaycctggaattaaaataaggcaactktgtaaa

ctccttaggggggccaaagcactaacagacatagtaccactaactgaagaagcagaa---

------------------------------------------------

>CRF07BC.D2014-038

cctcaaatcactctttggcaacgaccccttgttaccataaagataggggggcaattaaag

gaagctctactagatacaggagcagatgatacagtattagaagacatgaatttgccaggg

aaatggaaaccaaaaatgatagggggaattggaggttttatcaaagtaagacagtatgaa

cagatacccatagaaatytgtggacataaagctataggtacagtattagtaggacctaca

cctgtcaacataattggaagaaatctgttgactcagattggttgtactttaaattttcca

atcagtcctattgaaactgtaccagtaaarctaaagccaggaatggatggcccaaaggtt

aaacaatggccattaacaaaagagaaaatagaagcattaacagcaatttgtgatgaaatg

gaaaaggaaggaaaaattacaaaaattgggcctgaaaatccatacaacactccaatattt

gccataaaaaagaaagacagtactaagtggagaaaattagtagatttcagggaactcaat

aaaagaactcaagatttttgggaagttcaattaggaataccacacccagcaggactaaaa

aagaaaaaatcagtgacagtactggatgtgggggatgcatatttttcagttcctttatat

gaagacttcaggaaatatactgcattcaccatacctagtacaaacaatgaaacaccaggg

attaggtatcagtacaatgtacttccacagggatggaaaggatcaccagcaatatttcaa

agtagtatgataaaaatcttagagccttttaraaaacaaaatccagacatagycatctat

caatacatggatgatttgtatgtaggatctgacttagagatagggcagcatagaacaaaa

atagaggaactgagacaacatttgttgaggtggggatttacyacaccagacaagaaacat

cagaaagaacctccatttctttggatggggtatgaactccatcctgacaaatggacagta

cagcctatacagctaccagtacaagatagctggacygtcaatgatatacaaaagttagtg

ggaaarttaaactgggcaagtcagatttatcctggaattaaagtaaggcaactttgtaaa

ctccttaggggggccaaagcactaacagacatagtaccactaactgaagaagcagaatta

gaaattggcag-------------------------------------

>CRF07BC.D2014-039

cctcaaatcactctttggcaacgaccccttgttaccataaagataggggggcaatcaaaa

gaggctctattagatacaggagcagatgatacagtattagaagacatgaatttgccaggg

aaatggaaaccaaaaatgatagggggaattggaggttttatcaaagtaagacagtatgaa

cargtacccatagaaatctgtggacataaagctataggtacagtrttagtaggacctaca

cctgtcaacataattggaagaaatctgttgactcagcttggttgtactttaaattttcca

atcagtcctatcgacactgtaccagtaaaactaaagccaggaatggatggcccaaaggtt

aaacaatggccattgacaaaagagaaaatagaagcattaacagcaatttgtgttgaaatg

gaaaaggaaggaaaaattacaaaaattgggcctgaaaatccatacaacactccaatattt

gccataaaaaagaaagacagcactaagtggagaaaattagtagatttcagggaactcaat

aaaagaactcaagatttttgggaggttcaattaggaataccacacccagcaggattaaaa

aagaaaaaatcagtgacagtgctggatgtgggggatgcatatttttcagttcctctatat

gaagacttcaggaaatatactgcattcaccatacctagtataaacaatgaaacaccaggg

atcaggtatcagtacaatgtacttccacagggatggaaaggatcaccagcartatttcaa

agtagcatgayaaaaatcttagagccttttagaaaacaaaatccagacatagtcatctat

caatacatggatgatttgtatgtaggatctgacttagagatagggcagcatagaacaaaa

atagaggaactgagacaacatttgttgaggtggggattcaccacaccagacaagaaacat

cagaaagaacctccatttctttggatggggtatgaactccatcctgacaaatggacagta

cagcctatacagctgccagtacaagatagctggactgtcaatgatatacaaaagttagtg

ggaaaattaaactgggcaagtcagatttatcctggaattaaggtaaggcagctttgtaaa

ctccttaggggaaccaaagcactaacagacatagtaccactaactgaagaagcaga----

------------------------------------------------

>CRF07BC.D2014-076

cctcaaatcactctttggcaacgaccccttgttaccataaaaataggggggcaattaaag

gaagctctattagatacaggagcagatgatacagtattagaagacatggatttgccaggg

aaatggaaaccaaaaatgatagggggaattggaggttttatcaaagtaaracagtatgaa

cagatacccatagaaatctgtggacataaagttataggtacagtattagtaggacctaca

cctgtcaacataatyggaagaaatctgttgactcagcttggttgtactttaaattttcca

atcagtcctattgaaactgtaccagtaaaactaaagccaggaatggatggcccaaaggtt

aaacaatggccattgacaaaagagaaaatagaagcattaacagaaatttgtactgaaatg

gaaaaggaaggaaaaattacaaaaattgggcctgaaaatccatacaatactccaatattt

gccataaaaaagaaagacagtactaagtggagaaaattagtagatttcagggaactcaat

aaaagaactcaagatttttgggaagtgcaattaggaataccacacccagcaggattaaaa

aggaaaaaatcagtgacagtgctggatgtgggggatgcatatttttcagttcctttagat

gaaracttcaggaaatatactgcattcaccatacctagtataaacaatgaaacaccaggg

attaggtatcagtacaatgtacttccacagggatggaaaggatcaccagcgatatttcaa

agtagcatgacaaaaatcttagagccttttagaaaacaaaatccagacatagtcatctat

caatacatggatgatttgtatgtaggatctgacttagagatagggcagcatagaacaaaa

atagaggaactgagacaacacttgttgaggtggggatttaccacaccagacaaaaaacat

cagaaagaacctccatttctttggatggggtatgaactccatcctgacaaatggacagtg

cagcctatacagctaccagtacaagatagctggactgtcaatgatatacaaaagttagtg

ggaaaattaaactgggcaagtcagatttatcctggaattaaagtaagacagctttgtaaa

ctccttagggggaccaaagcactaacagatatagtaccactaactgaagaagcaga----

------------------------------------------------

>CRF07BC.D2014-079

cctcaaatcactctttggcaacgaccccttgttaccataaagataggggagcaattaaag

gaagctctattagatacaggagcagatgatacagtattagaagacatgaatttgccaggg

aaatggaaaccaaaaatgatagggggaattggaggttttatcaaagtaagacagtatgaa

cagatayccatagaaatctgtggacataaagttataggtacagtattagtaggacctaca

cctgtcaacataattggaagaaatctgttgactcagcttggttgtactttaaattttcca

atcagtcctattraaactgtaccagtaaaactaaagccaggaatggatggcccaaaggtt

aaacaatggccattgacaaaagagaaaatagaagcattaacagcaatttgtgatgaaatg

gaaaaggaaggaaaaattacaaaaattgggcctgaaaatccatacaacactccaatattt

gccataaaaaagaaagacagtactaagtggagaaaactagtagatttcagggaactcaat

aaaagaactcaagatttttgggaagttcaattaggaataccacacccagcaggattaaaa

aagaaaaaatcagtgacagtgctggatgtgggggatgcatatttttcagttcctttacat

gaagacttyaggaaatatacwgcattcaccatacctagtataaacaatgaaacaccaggg

attaggtatcagtacaatgtacttccacagggatggaaaggatcaccagcaatatttcaa

agtagcatgacaaraatcttagarccttttagaaaacaaaatccagacatagtcatctat

caatacatggatgatttgtatgtaggatctgacttagagatagggcagcatagaacaaaa

atagaggaactgagacaacatttgttgaggtggggatttaccacaccagacaaraaacat

caaaaagaacctccatttctttggatgggrtatgaactccatcctgacaaatggacagta

cagcctatacagctgccagtacaagatagctggactgtcaatgatatacaaaagttagtg

ggaaaattaaattgggcaagtcagatttatcctggaattaaagtaaggcaactttgtaaa

ctccttagggggaccaaagcaytaacagacatagtaccactaactgaagaagcagaa---

------------------------------------------------

>CRF07BC.D2014-081

cctcaaatcactctttggcaacgaccccttgtctcaataaagataggggggcaattaaag

gaagctctattagatacaggagctgatgatacagtattagaagacatgaatttgccaggg

aaatggaaaccaaaaatgatagggggaattggaggttttatcaaagtaagacagtatgaa

cagatacccatagaaatctgtggacacaaagctgttggtacagtattaataggacctaca

cctgtcaacataattggaagaaatctgttgactcagcttggatgtactttaaattttcca

atcagtcctattgaaactgtaccagtaaaattaaagccaggaatggatggcccaaaggtt

aaacaatggccattgacagaagagaaaataaaagcattaacagcaatttgtgatgaaatg

gagaaggaaggaaaaattacaaaaattgggcctgaaaatccatataacactccaatattt

gccataaaaaagaaggacagtactaagtggagaaaattagtagatttcagggaactcaat

aaaagaactcaagatttttgggaagttcaattaggaataccacacccagcagggttaaaa

aagaaaaaatcagtgacagtactagatgtgggggacgcatatttttcagttcctttacat

gaagacttcaggaaatatactgcattcaccatacctagtaaaaacaatgaaacaccagga

attaggtatcagtacaatgtacttccacagggatggaaaggatcaccagcaatattccaa

agtagcatgacaaaaatcttagagccgtttagaaaacaaaatccagacatagatatctat

caatacatggatgatttgtatgtaggatctgacttggaaatagggcagcatagaacaaaa

atagaggaactgagacagcatttgttgaggtggggatttaccacaccagacaagaaacat

cagaaagaacctccatttctttggatggggtatgagctccatcctgacaaatggacagta

cagcctatacagctgccagaaaaagatagctggactgtcaatgatatacaaaagttagtg

ggaaaattaaactgggcaagtcagatctatcctggaattaaagtcaggcaactttgtaaa

ctccttaggggggccaaagcactaacagacatagtaccactaactgaagaagcagaatta

g-----------------------------------------------

>CRF07BC.D2014-082

cctcaaatcactctttggcaacgaccccttgttaccataaagataggggggcaattaaag

gaagctctattagatacaggagcagatgatacagtattagaagacatgaatttgccaggg

aaatggaaaccaaaaatgatagggggaattggaggttttatcaaagtaagacagtatgaa

cagatacccatagaaatctgtggacataaagctataggtacagtattagtaggacctaca

cctgtcaacataattggaaggaatctgttgactcagcttggttgtactttaaattttcca

atcagtcctattgaaactgtaccagtaaaactaaagccaggaatggatggcccaaaggtt

aaacaatggccattgacaaaagagaaaatagaagcattaacagcaatttgtaatgaaatg

gaaaaggaaggaaaaattacaaaaattgggcctgaaaatccatacaacactccaatattt

gccataaaaaagaaagacagtactaagtggagaaaattagtagatttcagggaactcaat

aaaagaactcaagatttttgggaagttcaattagggataccacacccagcaggattaaaa

aagaagaaatcagtgacagtgctggatgtgggggatgcatatttttcagttcctttatat

gaagacttcaggaaatatactgcattcaccatacctagtataaacaatgaracaccaggg

attaggtatcagtacaatgtacttccacagggatggaaaggatcaccagcaatatttcaa

agtagcatgacaaaaattttagagccttttagaaaacaaaatccagacatagtaatctat

caatacatggatgatttgtatgtaggatctgacttagagatagggcagcatagaacaaaa

atagaggaactgagacaacatttgttgaggtggggatttaccacaccagacaagaaacat

caaaaggaacctccatttctttggatggggtatgaactccatcctgacaaatggacagta

cagcctatacaactgccagtacaagatagctggactgtcaatgatatacaaaagctagtg

ggaaaattaaactgggcaagtcagatttatcctggaattaaagtaaggcaactttgtaaa

ctccttaggggggccaaagcactaacagacatagtaccactaactgaagaagcagaatta

gaaattggcaga------------------------------------

>CRF07BC.D2014-086

cctcaaatcactctttggcaacgaccccttgttacaataaagatagggggrcaattaaag

gaagcyctattagayacaggagcagatgayacagtattagaagayatgaatttgccaggg

aaatggaaaccaaaaatgataggrggaattggaggttttatcaaagtaagacartatgaa

cagrtacccatagaaatytgtggacataaagctataggtacagtattagtaggacctaca

cctrtyaacataattggaagaaatytgttractcagcttggttgtacwttaaattttcca

atcagtcctattgaaactgtaccagtaaaaytaaagccaggaatggatggcccaaaggtt

aaacaatggccattgacaaaagagaaaatagaagcattaacagcaatttgtgaggaaatg

gaaaaagaaggaaaaattacaaaaattgggcctgaaaatccatacaacactccaatattt

gccataaaaaagaaagacagtactaagtggagaaaattagtagatttcagggaactcaat

aaaagaactcaagayttttgggaagttcaattaggaataccacacccagcaggattaaaa

aagaaaaaatcagtgacagtgctggatgtgggggatgcatatttttcagttcctttatat

gaagacttcaggaaatatactgcattcaccatacctagtataaacaatgaaacaccaggg

attaggtatcagtacaatgtacttccacagggatggaaaggatcaccagcaatatttcaa

agtagcatgacaaaaattttagagccttttagaaagcaaaatccagayatagtcatctat

caatayatggatgatttgtatgtaggatctgacttagagataggrcagcatagaacaaaa

atagaggaactgagacaacatttgttgrrgtggggatttaccacaccagacaagaaacay

cagaaagaacctccatttctttggatggggtatgaactccatcctgacaaatggacagta

cagcctatacagctgccagtaaaagatagctggactgtcaatgatatacaaaagttagtg

ggaaaattaaactgggcaagtcagatttatcctggaattaaagtaaggcaactttgtaaa

ctccttagggggaccaaagcactaacagacatagtaacactaactgaagaagcagaatta

gaaattggcaga------------------------------------

>CRF07BC.D2014-090

cctcaaatcactctttggcaacgaccccttgtcccaataaaaataggggggcaattaaag

gaagctctattagatacaggagcagatgatacagtattagaagacatgaatttgccaggg

aaatggaaaccaaaaatgatagggggaattggaggttttatcaaagtaagacartatgaa

cagatacccatagaaatctgtggacataaagctataggtacagtattagtaggacctaca

cctgtcaacataattggaagaaatctgttgacycagcttggttgcactttaaattttcca

atcagtcctattgaaactgtaccagtaaaattaaaaccaggaatggatggcccaaaggta

aaacaatggccattgacaaaagagaaaatagaagcattaacagcaatttgtgatgaaatg

gaaaaggaaggaaarattacaaaaattgggcctgaaaatccatacaacactccaatattt

gccataaaaaagaaggacagtactaagtggagaaaattagtagatttyagggaactcaat

aaaagaactcaagatttctgggaagttcagttaggaataccacacccagcagggttaaaa

aagaaaaaatcagtgacagtactggatgtgggggatgcatatttttcagttcctttacat

gaagacttcaggaaatayactgcattcaccatacctagtgtaaacaatgaaacaccrggg

attaggtatcagtataatgtacttccacagggatggaaaggatcaccagcaatatttcaa

agtagcatgacaaaaatcttagagccttttagaaaacaaaatccagacatagtcatctat

caatacatggatgatttgtatgtaggatctgacttagagataggrcagcatagaacaaaa

atagargaactgagacaacatttgttgaagtggggatttaccacaccagacaaraaacac

cagaaagaacctccatttctgtggatggggtatgaactccatcctgacaaatggacagta

cagcctatacagctgccagtacaagatagctggactgtcaaygatatacaaaagttagtg

ggaaaattaaactgggcaagtcagatttatcctggaattaaagtaaggcaactttgtaaa

ctccttagggggaccaaagcactaacagacatagtaccactaactgaagaagcag-----

------------------------------------------------

>CRF07BC.D2014-091

cctcaaatcactctttggcaacgaccccttgttaccataaagataggggggcaattaaag

gaagctctattagatacaggagcagatgatacagtattagaagacatgaatttgccaggg

aaatggaaaccaaaaatgatagggggaattggaggttttatcaaagtaagacaatatgaa

cagatacccatagaaatctgtggacataaagctataggtacagtattagtaggacctaca

cctgtcaacataattggcagaaatctgttgactcagctcggttgtactttaaattttcca

atcagtcctattgaaactgtaccagtaaaactaaagccaggaatggatggcccaaaggtt

aaacaatggccattgacaaaagagaaaatagaagcattaacagcaatttgtgatgaaatg

gaaaaggaaggaaaaattacaaaaattgggcctgaaaatccatacaacactccaatattt

gccataaaaaagaaagacagtactaagtggagaaaattagtagatttcagggaactcaat

aaaagaactcaagatttttgggaagttcaattaggaataccacacccagcaggattaaaa

aagaaaaaatcagtgacagtgctggatgtgggggatgcatatttttcagttcctttacat

gaagacttcaggaaatatactgcattcaccatacctagtacaaacaatgaaacaccaggg

attaggtatcagtacaatgtacttccacagggatggaaaggatcaccagcaatatttcaa

agtagcatgacaaaaatcttagagccttttagaaagcaaaatccagacatagtcatctat

caatacatggatgatttgtatgtaggatctgacttagagatagggcaacatagaacaaaa

atagaggaactgagacaacatttgttgcggtggggatttaccacaccagacaaaaaacat

cagaaagaacctccatttctttggatggggtatgaactccatcctgacaaatggacagta

cagcctatacagctgccagtacaagatagctggactgtcaatgatatacaaaagttagtg

ggaaaattaaactgggcragtcagatttatcctggaattaaagtaaggcaactttgtaaa

ctccttaggggggccaaagcattaacagacatagtaccactaactgaagaagcagaatta

gaaattggcag-------------------------------------

>CRF07BC.D2014-092

cctcaaatcactctttggcaacgaccccttgtcacaataaagataggggggcaattaaag

gaagctctactagatacaggagcagatgayacagtattagaagacatgaatttgccaggg

aaatggaaaccaagaatgatagggggaattgggggttttatcaaagtaagacagtatgag

cagatacccatagaaatctgtggacataaagctgtaggtacagtattaataggaccaaca

cctgttaacataattggaaggaatctgttgactcagcttggttgtactttaaattttcca

atcagtcctattgaaactgtaccagtaaaactaaaaccaggaatggatggcccaaaggtt

aaacaatggccattgacaaaagagaaaatagaagcattaacagcaatttgtgatgaaatg

gaaaaggaaggaaaaattacaaaaattgggcctgaaaatccatacaacactccaatattt

gccataaaaaagaaagacagtactaagtggagaaaattagtagatttcagggaactcaat

aaaagaactcaagatttttgggaagttcaattaggaataccacacccagcaggattaaaa

aagaaaaaatcagtgacagtgctggatgtgggagatgcatatttttcagttcctttatat

gaagacttcagraaatatactgcattcaccatacctagtataaacaatgaaacaccaggg

attaggtatcagtacaatgtacttccacagggatggaaaggatcaccagcaatatttcaa

agtagcatgacaaaaatcttagagccttttagaaaacaaaacccagacatagtcatctat

caatacatggatgatttatatgtaggatctgacttagagatagggcagcatagaacaaaa

atagaggaactgagacagcatttgttaaggtggggatttaccacaccagacaagaaacat

cagaaggaacctccatttctttggatggggtatgaactccatcctgacaaatggacagta

cagcctatacagctaccagtacaagatagctggactgtcaatgatatacaaaagttagtg

ggaaaattaaattgggcaagtcagatttatcctggaattaaagtaaggcaactttgtaaa

ctccttagggggaccaaagcactaacagacatagtaccactaactgaagaagcagaa---

------------------------------------------------

>CRF07BC.D2014-146

cctcaaatcactctttggcaacgaccccttgttacaataaagataggggggcaattaagg

gaagctctattagatacaggagcagatgatacagtattagaagacatggatttgccaggg

aaatggaaaccaaraatgatagggggaattggaggttttatcaaagtaagacagtatgaa

cagatacccatagaaatctgtggacataaagctataggtacagtattagtaggacctaca

cctgtcaacataattggaagaaatctgttgactcagcttggttgtaccttaaattttcca

atcagtcctattgaaactgtaccagtaaaactaaagccaggaatggatggcccaaaggtt

aaacaatggccattgacaaragaaaaaatagaagcattaacagmaatttgtratgaaatg

gaaaaggaaggaaaaattacaaaaattgggcctgaaaatccatacaacactccaatattt

gccataaaaaagaaagacagtactaagtggagaaaattagtagatttcagggaactcaat

aaaagaactcaagatttttgggaagttcaattaggaataccacacccagcaggattaaaa

aagaaaaaatcagtaacagtgctggatgtgggggatgcatatttttcagttcctttagat

raagacttcaggaaatatactgcattcaccatacctagtrtaaacaatgaarcaccaggr

attagatatcaatacaatgtacttccacagggatggaaaggatcaccagcaatatttcaa

agtagcatgacaaaaatcttagagccttttagaaagcaaaatccagacatagtcatctat

caatacatggatgatttgtatgtaggatctgacttagagatagggcagcatagaacaaaa

atagaggaactgagacaacatttgttgargtggggatttaccacaccagacaagaaacat

cagaaagaaccaccatttctttggatggggtatgaacttcatcctgacaaatggacagta

cagcctatacagctgccagtacaagatagctggactgtcaatgatatacaaaagttagtg

ggaaagttaaactgggcaagtcagatttatcctggaattaaaryaagacaactttgtaaa

ctccttagggggrccaaagcactaacagacatagtaccactaactgaagaagcagaat--

------------------------------------------------

>CRF07BC.D2014-147

cctcaaatcactctttggcagcgaccccttgttacaataaagataggggggcaattaaag

gaagctctattagatacaggagcagatgatacagtattagaagacatgaatttgccaggg

aaatggaaaccaaaaatgatagggggaattggaggttttatcaaagtaaggcagtatgaa

cagatacccatagaaatctgtggacataaagctataggtacagtattagtaggacctaca

cctgtcaacataattggaagaaatctgttgactcagcttggttgtactttaaattttcca

atcagtcctattgaaactgtaccagtaaaactaaagccaggaatggatggcccaaaggtt

aaacaatggccattgacaaaagagaaaatagaagcattaacagcaatttgtgatgaaatg

gaaaaggaaggaaagattacaaaaattgggcctgaaaatccatacaacactccaatattt

gccataaaaaagaaagacagtactaagtggagaaaattagtagatttcagggaactcaat

aaaagaactcaagatttttgggaagttcaattaggaataccacacccagcaggattaaaa

aagaaaaaatcagtaacagtgctggatgtgggggatgcatatttttcagttcctttatat

gaagacttcaggaaatatactgcattcaccatacctagtataaacaatgaaacaccaggg

attaggtatcagtacaatgtacttccacagggatggaaaggatcaccagcaatatttcaa

agtagcatgacaagaatcttagagccttttagaaaacaaaatccagatatagtcatctat

caatacatggatgatttgtatgtaggatctgacttagaaatagggcagcatagaacaaaa

atagaggaactgagacaacatttgttgaagtggggatttaccacaccagacaagaaacat

cagaaagaacctccatttctttggatggggtatgaactccatcctgacaaatggacagta

cagcctatacagctgccagtacaagatagctggactgtcaatgatatacaaaagttagtg

ggaaaattaaattgggcaagtcagatttatcctggaattaaagtaaggcaactttgtaaa

ctccttagggggaccaaagcactaacagacatagtaccactaactgaagaagcaga----

------------------------------------------------

>CRF07BC.D2014-150

cctcaaatcactctttggcaacgaccccttgtcccaataaagataggggggcaattaaag

gaagctctattagatacaggagcagatgatacagtattagaagacatgaatttgccaggg

aaatggaagccaaaaatgatagggggaattggaggttttatcaaagtaagacagtatgaa

cagatacccatagaaatctgtggacataaagctataggtacagtattagtaggacctaca

cctgtcaacataattggaagaaatctgttaactcagcttggttgtactttaaattttcca

atcagtcctattgaaactgtaccagtaaaactaaagccaggaatggatggcccaaaggtt

aaacaatggccattaacaaaagagaaaatagaagcattaacagcaatttgtgtagaaatg

gaaaaggaaggaaaaattacaaaaattgggcctgaaaatccatacaacactccaatattt

gccataaaaaagaaagacagtactaagtggagaaaattagtagatttcagggaactcaat

aaaagaactcaagatttttgggaagttcaattaggaataccacacccagcaggattaaaa

aagaaaaaatcagtaacagtgctggatgtgggggatgcatatttttcagtccctttatat

gaagacttcaggaaatatactgcattcaccatacctagtataaacaatgagacaccaggg

attaggtatcagtacaatgtacttccacagggatggaagggatcaccagcaatatttcaa

agtagcatgacaaaaatcttagagccttttagaaaacaaaatccagacatagtcatctat

caatacatggatgatttgtatgtaggatctgacttagagataggacagcatagaacaaaa

atagaggaactgagacaacatttgttgaggtggggatttaccacaccagacaagaaacat

cagaaagaacctccatttctttggatggggtatgaactccatcctgacaaatggacagta

cagcctatacagctgccagtacaagatagctggactgtcaatgatatacaaaagttagtg

ggaaaattaaactgggcaagtcagatttatcctggaattaaagtaaggcaactttgtaaa

ctccttaggggggccaaagcactaacagacatagtaccactaactgaagaagcagaat--

------------------------------------------------

>CRF07BC.D2014-152

cctcaaatcactctttggcaacgaccccttgtyacmataaagataggggggcaattaagg

gaagctctattagatacaggagcagatgatacagtattagaagacatggatttgccaggg

aaatggaaaccaaaaatgatagggggaattggaggttttatcaaagtaagacagtatgaa

cagatacccatagaaatctgtggacataaagctataggtacagtattagtaggacctaca

cctgtcaacataattggaagaaatctgttgactcagcttggttgtaccttaaattttcca

atcagtcctattgaaactgtaccagtaaaactaaagccaggaatggatggcccaaaggtt

aaacaatggccattgacaaaagaaaaaatagaagcattaacagcaatttgtgatgaaatg

gaaaaggaaggaaaaattacaaaaattgggcctgaaaatccatacaacactccaatattt

gccataaaaaagaaagacagtactaagtggagaaaactagtagatttcagggaactcaat

aaaagaactcaagatttttgggaagttcaattaggaataccacacccagcaggattaaaa

aagaaaaaatcagtaacagtgctggatgtgggggatgcatatttttcagttcctttagat

aaagacttcaggaaatatactgcattcaccatacctagtgtaaacaatgaagcaccaggg

attaggtatcaatacaatgtacttccacagggatggaaaggatcaccagcaatatttcaa

agtagcatgacaaaaatcttagagccttttagaaagcaaaatccagacatagtcatctat

caatacatggatgatttgtatgtaggatctgacttagagatagggcagcatagaacaaaa

atagaggaactgagacaacatttgttgaggtggggatttaccacaccagacaagaaacat

cagaaagaaccaccatttctttggatggggtatgaacttcatcctgacaaatggacagta

cagcctatacagctgccagtacaagatagctggactgtcaatgatatacaaaagttagtg

ggaaagttaaactgggcaagtcagatttatcctggaattaaagtaagacaactttgtaaa

ctccttaggggggccaaagcactaacagacatagtaccactaactgaagaagcagaatta

gaattggca---------------------------------------

>CRF07BC.D2014-153

cctcaaatcactctttggcaacgaccccttgttaccataaagataggggggcaattaaag

gaagctctattagatacaggagcagatgatacagtattagaagacatgaatttgccaggg

aaatggaaaccaaaaatgatagggggaattggaggttttatcaaagtaagacaatatgaa

gagatacccatagaaatctgtggacataaagytataggtacagtattagtaggacctaca

cctgtcaacataattgggaggaatctgttgactcagcttggttgtactttaaattttcca

attagtcctattgaaactgtaccagtaaaattaaagccaggaatggatggcccaaaggtt

aracaatggccattgacaaaagagaaaatagaagcattaacagcaatttgtgatgaaatg

gaaaaggaaggaaaaattacaaaaattgggcctgaaaatccatacaacactccaatattt

gccataaaaaagaaagacagtactaagtggagaaaattagtagatttcagggaactcaat

aaaagaactcaagatttttgggaagttcaattaggaataccacatccagcaggattaaaa

aagaaaaaatcagtgacagtgctggatgtgggagatgcatatttttcagttcctttatat

gaagacttcaggaaatatactgcattcaccatacctagtataaataatgaaacaccaggg

rttagatatcartacaatgtacttccacagggatggaaaggatcaccagcaatatttcaa

agtagcatgacaaaaatcttagagccttttagaaaacaaaatccagacatagtcatctat

caatacatggatgatttgtatgtaggatctgacttagagatagggcarcatagaacaaaa

atagaggaactgagacaacatttgttgaggtggggatttaccacaccagacaagaaacat

cagaaagaacctccatttctttggatggggtatgaactccatcctgacaaatggacagta

cagcctatacagttgccagtacaagatagctggactgtcaatgatatacaaaagttagtg

ggaaarttaaactgggcaagtcagatttatcctggaattaaagtaaggcaactttgtaaa

ctccttaggggggccaaagcactaacagacatagtaccactaactgaagaagcagaatta

gaattgg-----------------------------------------

>CRF07BC.D2014-156

cctcaaatcactctttggcaacgaccccttgttaccataaagataggggggcaattaaag

gaagccctattagatacaggagcagatgatacagtattagaagaaatgaatttgccaggg

aaatggaaaccaaaaatgatagggggaattggaggttttatcaaagtaagacagtatgaa

cagatacccatagaaatctgtggacataaagctataggtacagtattaataggacctaca

cctgtcaacataattggaaggaatctgttgactcaacttggttgtactttaaattttcca

atcagtcctattgaaactgtgccagtaaaattaaaaccaggaatggatggcccaagggtt

aaacaatggccattgacaaaagagaaaatagaagcattaacagcaatttgtgatgaaatg

gaaaaggaaggaaaaattacaaaaattgggcctgaaaatccatacaacactccaatattt

gccataaaaaagaaagacagtactaagtggagaaaattagtagatttcagggaactcaat

aaaaggactcaagatttttgggaagttcaattaggaataccacatccagcaggattaaaa

aagagaaaatcagtgacagtgctggatgtgggagatgcatatttttcagttcctttatat

gaagacttcaggaaatatactgcattcaccatacctagtataaacaatgaaacaccaggg

attagatatcagtacaatgtacttccacagggatggaaaggatcaccagcaatatttcaa

agtagcatgacaaaaatcttagagccttttagaaaacaaaacccagacataatcatttat

caatacatggatgatttgtatgtaggatctgacttagagatagggcagcatagaacaaaa

atagaggaactgagacaacatttgttgaggtgggggtttaccacaccagacaagaaacat

cagaaagaacctccatttctttggatggggtatgaactccatcctgacaaatggacagta

cagcctatacagctgccagaacaagatagctggactgtcaatgatatacaaaagttagtg

ggaaaattaaactgggcaagtcagatttatcctggaattaaggtaaggcaactttgtaaa

ctccttagggggaccaaagcactaacagacatagtaccactaactgaagaagcagaatta

g-----------------------------------------------

>CRF07BC.D2014-157

cctcaaatcactctttggcaacgaccacttgttacmataaagataggggggcaattaaag

gaagctctattagatacaggagcagatgatacagtattagaagacatggacttgccaggg

aaatggaaaccaaaaatgatagggggaattggaggttttatcaaagtaagacaatatgaa

gagatacccatagaaatctgtggacataaagctataggtacagtattagtaggacctaca

cctgtcaacataattggaaggaatctgttgactcagcttggttgtactttaaattttcca

atcagtcctattgaaactgtaccagtaaaactaaagccaggaatggatggcccaaaggtt

aaacaatggccattgacaaaggagaaaatagaagcattaacagcaatttgtgatgaaatg

gaaaaggaaggaaaaattacaaaaattgggcctgaaaatccatataacactccaatattt

gccataaaaaagaaagacagtactaagtggagaaaattagtagatttcagggaactcaat

aaaagaactcaagatttttgggaagttcaattaggaataccacacccagcaggattaaaa

aagaaaaaatcagtgacagtgctggatgtgggagatgcatatttttcagttcctttagat

raaracttcaggaaatatactgcattcaccatacctagtataaacaatgaaacaccaggg

attaggtatcagtacaatgtacttccacagggatggaaaggatcaccagcaatatttcaa

agtagcatgacaaaaatcttagagccttttagaaaacaaaatccagacatagtcatctat

caatacatggatgatttgtatgtaggatctgacttagagatargacagcatagaacaaaa

atagaggaactgagacaacatttgttgaagtggggatttaccacaccagacaagaaacat

cagaaagaacctccatttctttggatggggtatgaacttcatcctgacaaatggacagta

cagcctatacagttgccagtacaagatagctggactgtcaatgatatacaaaagttagtg

ggaaaattaaactgggcaagtcagatttatcctggaattaaagtaaggcaactttgtaaa

ctccttaggggggccaaagcactaacagacatagtaccactaactgaagaagcagaatta

gaattgg-----------------------------------------

>CRF07BC.D2014-158

cctcaaatcactctttggcaacgaccccttgtcacaataaagataggggggcaattaaag

gaagctctattagatacaggagcagatgatacagtattagaagacatgaatttgccaggr

aaatggaaaccaaaaatgatagggggaattggaggttttatcaaagtaagacagtatgaw

cagatacccrtagaaatctgtggrcayaargctrtaggtacagtattartaggrccyaca

cctgtcaacataattggaagaaatytgttgactcagmttggytgtactttaaatttyccm

atyagtcctattgaaactgtaccagtaaaaytaaagccaggaatggatggcccaaaggtt

aaacaatggccattgacaaaagaaaaaatagaagcattaacagcaatttgtgatgaaatg

gaaaaggaaggaaaaattacaaaaattgggcctgaaaatccatacaacactccaatattt

gccataaaaaagaaagacagtactaagtggagaaarttagtagatttcagggaactcaat

aaaagaactcaagatttttgggaagttcaattaggaataccacacccagcaggattaaaa

aagaaaaaatcagtgacagtgctggatgtgggggatgcatatttttcagttcctttatat

gaagacttcaggaaatatactgcattcaccatacctagtataaacaatgaaacaccaggg

attaggtatcagtacaatgtacttccacagggatggaaaggatcaccagcaatatttcaa

agtagcatgacaagaatyttagagccttttagaaaacaaaatccagacatagtcatctat

caatacatggatgatttgtatgtaggatctgacttagagatagggcagcatagaacaaaa

atagaggaactgagacaacatttgttgaggtggggatttaccacaccagacaagaaacat

cagaaagaacctccatttctttggatggggtatgarctccatcctgacaaatggacagta

cagcctatacagctgccagtacaagatagctggactgtcaatgatatacaaaagttagtg

ggaaaattaaactgggcaagtcagatttatcctggaattaaagtaaggcaactttgtaaa

ctccttaggggggccaaagcactaacagacatagtaccactaactgaagaagcagaat--

------------------------------------------------

>CRF07BC.D2014-189

cctcaaatcactctttggcaacgaccccttgttaccataaagataggggggcaattaaag

gaagctctattagatacaggagcagatgatacagtattagaagacatgaatttgccaggg

aaatggaaaccaaaaatgatagggggaattggaggttttatcaaggtaaggcaatatgat

cagatacctatagaaatttgtggaaaaagggctayaggtacagtgttagtaggacctaca

cctgtcaacataattggacgaaacmtgttgactcagcttggttgtactttaaattttcca

atcagtcctattgaaactgtaccagtaaaactaaagccaggaatggatggcccaarggtt

aaacaatggccattgacaaaagaaaaaatagaagcattaacagmaatttgtaaggaaatg

gaaaaagaaggaaaaattacaaaaattgggcctgaaaatccatacaacactccaatattt

gccataaaaaagaaagacagtactaagtggagaaaattagtagatttcagggaactcaat

aaaagaactcaagacttttgggaagttcaattaggaataccacacccagcaggattaaaa

aagaaaaaatcagtgacagtgctggatgtgggggatgcatatttttcagttcctttagat

gaagacttcaggaaatatactgcattcaccatacctagtataaacaatgaaacaccaggg

attaggtatcagtacaatgttcttccacagggatggaaaggatcaccagcgatatttcaa

agtagcatgacaaaaatcttagagccttttagaaaacaaaatccagacatagtcatctat

caatacatggatgatttgtatgtaggatctgacttagagatagggcagcatagaacaaaa

atagaggaactgagacaacacttgttgargtggggatttaccacaccagacaaaaaacat

cagaaagaacctccatttctttggatggggtatgaactccatcctgacaaatggacagta

cagcctatacagctaccagtacaagatagctggactgtcaatgatatacaaaagttagtg

ggaaaattaaactgggcaagtcagatttatcctggaattaaagtaaggcagctttgtaaa

ctccttaggggaaccaaagcactaacagacatagtaccactaactgaagaagcagaa---

------------------------------------------------

>CRF07BC.D2014-195

cctcaaatcactctttggcaacgaccccttgttaccataaagataggggggcaattaaag

gaagctctattagatacaggagcagatgatacagtattagaagaaatgaatttrccagga

aaatggaarccaaaaatgatagggggaattggaggttttatcaaagtaagacagtatgaa

caggtacccatagaaatttgtggacataaagctataggtacagtattaataggacctaca

cctgtcaacataattggaagaaatctgttgactcagcttggttgtactttaaattttcca

atcagtcctattraaactgtaccagtaaaactaaagccaggaatggatggcccaagggtt

aaacartggccattgacaaaagagaaaatagaagcattaacagcaatttgtgatgaaatg

gaaaaggaaggraaaattacaaaaattgggcctgaaaatccatacaacactccaatattt

gctataaaaaagaaagacagtactaagtggagaaaattagtagatttcagggaactcaat

aaaagaactcaagatttttgggaagttcaattaggaataccacacccagcaggattaaaa

aagaaaaaatcagtgacagtgctggatgtgggggatgcatatttttcagttcctttagat

gaagacttcaggaaatatactgcattcaccatacctagtataaacaatgaaacaccaggg

attaggtatcagtacaatgtactyccacagggatggaaaggatcaccagcaatatttcaa

agyagcatgacaaaaattttagagccttttagaaaacaaaatccagacatagtcatctat

caatacatggatgatttgtaygtaggatctgayttagagatagggcagcatagaataaaa

atagaggaactgagacaacatttgttgaggtggggatttactacaccagacaagaaacat

cagaaagaacctccatttttgtggatggggtatgaactccatcctgacaaatggacagta

cagcctatacagctgccaatacaagatagctggactgtcaatgatatacaaaagttagtg

ggaaaattaaactgggcaagtcagatttatcctggaattaaagtaaggcaactttgtaaa

ctccttaggggggccaaagcactaacagacatagtaccactaactgaagaagcagaa---

------------------------------------------------

>CRF07BC.G-HZ130057

cctcaaatcactctttggcagcgaccccttgtcacaataaagataggggggcaattaaag

gaagctctattagatacaggagcagatgatacagtattagmagacatgaatttgccaggg

aaatggaaaccaaaaatgatagggggaattggaggttttatcaaagtaagacagtatgaa

caggtacccatagaaatctgtggacataaagctataggtacagtattagtaggacctaca

cctgtcaacataattggaagaaatctgttgactcagcttggttgtactttaaattttcca

atcagtcctattgaaactgtaccagtaaaactaaagccaggaatggatggcccaaaggtt

aaacaatggccattgacaaaagagaaaatagaagcattaacagcaatttgtgatgaaatg

gaaaaggaaggaaaaattacaaaaattgggcctgaaaatccatacaacactccaatattt

gccataaaaaagaaagacagtactaagtggagaaaattagtagatttcagggaactcaat

aaaagaactcaagatttttgggaagttcaattaggaataccacacccagcaggattaaaa

aagaaaaaatcagtgacagtgctggatgtgggggatgcatatttttcagttcctttatat

gaagacttcaggaaatayactgcattcaccatacctagtataaacaatgaaacaccaggg

attaggtatcagyacaatgtacttccacagggatggaaaggatcaccagcaatatttcaa

agtagcatgacaaaaatcttagagccttttagaaaacaaaatccagacatagtcatctat

caatacatggatgatttgtatgtaggatctgacttagagataggacagcatagaacaaaa

atagaggaactgagacaacatttgttgaggtggggatttaccacaccagacaagaaacat

cagaaagaacctccatttctttggatggggtatgaactccatcctgacaaatggacagta

cagcctatacagctgccagtacaagatagctggactgtcaatgatatacaaaagttagtg

ggaaaattaaactgggcaagtcagatttatcctggaattaaagtaaggcaactttgtaaa

ctccttagggggaccaaagcactaacagacatagtaccactaactgaagaagcagaat--

------------------------------------------------

>CRF07BC.G-HZ130060

cctcaaatcactctttggcaacgacccctcgtcacaataaagataggggggcaattaaag

gaagctctattagatacaggagcagatgatacagtattagaagacatgaatttgccagga

aaatggaaaccaaaaatgatagggggaattggaggttttatcaaagtaagacagtatgaa

cagatacccatagaaatttgcggacacaaagctataggtacagtattaataggacctaca

cctgtcaacataattggcagaaatctgttgactcagcttggttgcactttaaactttccc

atcagtccyattgaaactgtaccagtaaaattaaagccaggaatggatggcccaaaggtt

aaacaatggccattgacagaagaaaaaataaaagcattaacagcaatatgtgatgaaatg

gaaaaggaagggaaaattacaaaaattgggcctgagaatccatataayactccaatattt

gccataaaaaagaaggacagtactaaatggagaaaattagtrgatttcagagaactcaat

aaaagaactcaagatttttgggargttcaattaggaataccacayccagcagggttaaaa

aagaaaaaatcagtgacagtactggatgtgggggatgcatatttttcagttcctctatat

gaagacttcaggaaatatactgcattcaccatacctagtataaacaatgaaacaccaggg

attaggtatcagtacaatgtacttccccagggatggaaaggatcaccagcaatattccaa

agtagcatgacaaagatcttagatccttttagaaaacaaaatccagacatagttatctat

caatacatggatgatttgtatgtaggatctgacttagagatagggcagcatagaacaaaa

atagaggaactgagacaacatttgttgaggtggggatttaccacaccagacaagaaacat

cagaaagaacctccatttctttggatggggtatgaactccatcctgacaaatggacagta

cagcctatacagctgccagataaagatagctggactgtcaatgacatacaaaagttagtg

ggaaaattaaactgggcgagtcaaatttatcctggaattaaagtaaggcaactttgtaaa

ctccttagggggaccaaagcactaacagacatagtaccactaactgaagaagcagaatta

gaaattggcaga------------------------------------

>CRF07BC.G-HZ130062

cctcaaatcactctttggcaacgaccccttgtcacaataaagataggagggcaattaaag

gaagctctattagatacaggagcagatgacacagtattagaagacatgaatttgccaggg

aaatggaaaccaaaaatgatagggggaattggaggttttatcaaagtaagacagtatgaa

gagatacccatagaaatctgtggacataaagttataggtacagtattagtaggacctaca

cctgtcaacataattggaagaaatctgttgactcagcttggttgtactttaaattttcca

atcagtcctattgaaactgtaccagtaaaactaaagccaggaatggatggcccaaaggtt

aaacaatggccattgacaaaagaaaaaatagaagcattaacagcaatttgtgatgaaatg

gaaaaggaaggaaaaattacaaagattgggcctgaaaacccatacaacactccaatattt

gccataaaaaagaaagacagtactaagtggagaaaattagtagatttcagggaactcaat

aaaagaactcaagatttttgggaagttcaattaggaataccacacccagcaggattaaaa

aagaaaaaatcagtgacagtgctggatgtgggggatgcatatttttcagttcctttatat

gaagactccaggaaatatactgcattcaccatacctagtataaacaatgaaacaccaggg

attaggtatcagtacaatgtacttccacaaggatggaaaggatcaccagcaatatttcaa

agtagcatgacaaaaatcttagagccttttagaaaacaaaatccagacatagtcatctat

caatacatggatgatttgtatgtagggtctgacctagagataggacagcatagaacaaaa

atagaggaactgagacaacatttgttgaggtggggatttaccacaccagacaagaagcat

caaaaagaacctccatttctgtggatggggtatgaactccatcctgacaaatggacagta

cagcctatacagctgccagtacaagatagctggactgtcaatgatatacaaaagttagtg

ggaaaattaaactgggcaagtcagatttatcctggaattaaagtaaggcaactttgtaaa

ctccttagggggaccaaagcactaacagacatagtaccactaactgaagaagcagaatta

g-----------------------------------------------

>CRF07BC.G-HZ130110

cctcagatcactctttggcaacgacccctcgtcccaataaggataggggggcaattaagg

gaagctctattagatacaggagcagatgatacagtattagaagacatgaatctgccaggg

aaatggaaaccaaaaatgatagggggaattggaggttttatcaaagtaaaacagtatgaa

cagatacccatagaaatctgtggacacaaagctataggtacagtattaataggaccaaca

cctgtcaacataattggaagaaatctgttgactcagcttggttgcactctaaattttcca

atcagtcccattgaaactgtaccagtaaaattgaagccaggaatggatggcccaaaggtt

aaacaatggccattgacagaagaaaaaataaaagcattaacagaaatttgtgatgaaatg

gaaaaggaaggaaagattacaaaaattgggcctgaaaatccatataacactccaatattt

gccataaaaaagaaggacagtactaagtggagaaaattagtagatttcagggaactcaat

aaaagaactcaagatttttgggaagttcaattaggaataccacacccagcagggctaaaa

aagaaaaagtcagtgacagtactggatgtgggggatgcatatttttcagttcctttacat

gaagacttcaggaaatatacagcattcaccatacctagtacaaacaatgaaacaccaggg

attaggtatcagtacaatgtacttccacagggatggaaaggatcaccagcaatattccaa

agtagtatgacaaaaatcttagaaccttttagaaaacaaaatccagacatagttatctac

caatacatggatgatttgtatgtaggatctgacttagagataaagcaacatagaacaaaa

atagaggaactgagacaacatttgttgaggtggggatttaccacaccagacaagaaacat

cagaaagaaccgccatttctttggatggggtatgaactccatcctgacaaatggacagta

cagcctatacagctgccagaaaaagatagctggactgtcaatgatatacaaaagttagtg

ggaaaattaaactgggcaagtcagatttatcctggtattaaagtaaggcaactttgtaaa

ctccttagggggactaaagcactaacagacatagtaccactaactgaagaagcagaat--

------------------------------------------------

>CRF07BC.G-HZ130190

cctcaaatcactctttggcaacgaccccttgttaccataaagataggggggcaattaaag

gaagctctattagatacaggagcagatgatacagtattagaagacatgaatttgccaggg

aaatggaaaccaaaaatgatagggggaattggaggttttatcaaagtaagacagtatgaa

cagatacccatagaaatctgtggacataaagctataggtacagtattaataggacctaca

cctgtcaacataattggaagaaatctgttgactcagcttggttgtactttaaattttcca

atcagtcctattgaaactgtaccagtaaaactaaagccaggaatggatggcccaaaggtt

aaacaatggccattgacaaaagagaaaatagaagcattaacagcaatttgtgatgaaatg

gaaaaggaaggaaaaattacaaaaattgggcctgaaaatccatacaacactccaatattt

gccataaaaaagaaagacagtactaagtggagaaaattagtagatttcagggaactcaat

aaaagaacccaagatttttgggaagttcaattaggaataccacacccagcaggattaaaa

aagaaaaaatcagtgacagtgctggatgtgggggatgcatatttttcagttcctttatat

gaagactttaggaagtatactgcattcaccatacctagtataaacaatgaaacaccaggg

attaggtatcagtacaatgtacttccacagggatggaaaggatcaccagcaatatttcaa

agtagcatgacaaaaatcttagagccttttagaaaacaaaatccagacatagtcatctat

caatacatggatgatttgtatgtaggatctgacttagaaatagggcaacatagaacgaaa

atagaggaactgagacaacatttgttgaggtggggatttaccacaccagacaagaaacat

cagaaagaacctccatttctttggatggggtatgaactccatcctgacaaatggacagta

cagcctatacagctaccagtacaagatagctggactgtcaatgatatacaaaagttagtg

ggaaaattaaactgggcaagtcagatttatcctggaattaaagtaaggcaactttgtaaa

gtccttaggggggccaaagcactaacagacatagtaccactaactgaagaagcaga----

------------------------------------------------

>CRF07BC.G-HZ130191

cctcaaatcactctttggcaacgaccccttgtcacaataaagataggggggcaattaaag

gaagctctactagatacaggagcagatgatacagtattagaagacatgaatttgccaggg

aaatggaaaccaaaaatgatagggggaattggaggttttatcaaagtacgacagtatgaa

cagatacccatagaaatctgtggacataaagctataggtacagtattagtaggacccaca

cctgtcaacataattggaagaaatctgttgactcagcttggttgtactttaaattttcca

atcagtccyattgaaactgtaccagtaaaactaaagccaggaatggatggcccaaaggtt

aaacaatggccattgacaaaagagaaaatagaagcattaacagcaatttgtgatgaaatg

gaaaaggaaggaaaaattacaaaaattgggcctgaaaatccatacaacactccaatattt

gccataaaaaagaaagacagtactaagtggagaaaattagtagatttcagggaactcaat

aaaagaactcaagatttttgggaagttcaattaggaataccacacccagcaggattaaga

aagcaaaaatcagtgacagtgctggatgtgggggatgcatatttttcagttcctttayat

gaagacttcaggaaatatactgcattcaccatacctagtataaacaatgagacaccaggg

attaggtatcagtacaatgtacttccacagggatggaaaggatcaccagcaatatttcaa

agtagcatgacaaaaatcttagagccttttagaaaacaaaatccagacatagtcatctat

caatacatggatgatttgtatgtaggatctgacttagagatagggcagcatagagcaaaa

atagaagaactgaggcaacatttgttgaggtggggatktaccacaccagacaaaaaacat

cagaaagaacctccatttctttggatggggtatgaactccatcctgacaaatggacagta

cagcctatacagctgccagtacaagatagctggactgtcaatgatatacaaaagttagtg

ggaaaattaaactgggcaagtcagatttatcctggaattaaagtaaggcaactttgtaaa

ctccttaggggagctaaagcattaacagacgtagtgccactaactgaagaagcagaa---

------------------------------------------------

>CRF07BC.G-HZ130192

cctcaaatcactctttggcagcgaccccttgttaccataaagataggggggcaattaaag

gaagctctattagatacaggagcagatgatacagtattagaagacatgaatttgccaggg

aaatggaaaccaaaaatgatagggggaattggaggttttatcaaagtaagacagtatgaa

cagrtacccatagaaatctgtggacataaagctataggtacagtattartaggacctaca

cctgtcaacataattggaagaaatctgttgactcagmttggttgtactttaaattttcca

atcagtcctattgaaactgtaccagtaaaactaaagccaggaatggatggcccaaaggtt

aaacaatggccattgacaaaagagaaaatagaagcattaacagcaatttgtgatgaaatg

gaaaaggaaggaaaaattacaaaaattgggcctgaaaatccatacaacactccaatattt

gccataaaaaagaaagacagtactaagtggagaaaattagtagatttcagggaactcaat

aaaagaactcaagatttttgggaagttcaattaggaataccacacccagcaggattaaaa

aagaaaaaatcagtgacagtgctggatgtgggggatgcatatttttcagttcctttatat

gaagacttcaggaaatatactgcattcaccatacctagtataaacaatgaaacaccaggg

attaggtatcagtacaatgtacttccacagggatggaaaggatcaccagcaatatttcaa

agtagcatgacaaaaatcttagagccttttagaaaacaaaatccagacatagtcatctat

caatacatggatgatttgtatgtaggatctgacttagagataggrcagcatagaacaaaa

atagaggaactgagacaacatttgttgargtggggatttaccacaccagacaagaaacat

cagaaagaacctccatttctttggatggggtatgaactccatcctgacaaatggacagta

cagcctatacagctgccagwacaagatagctggactgtcaatgatatacaaaagttagtg

ggaaaattaaactgggcaagtcagatttatcctggaattaaagtaaggcaactttgtaaa

ctccttagggggaccaaagcactaacagacatagtaccactaactgaagaagcagaatta

gaattgg-----------------------------------------

>CRF07BC.G-HZ130195

cctcaaatcactctttggcaacgaccccttgttacaataaagataggggggcaattaaag

gaagctctattagatacaggagcagatgatacagtattagaagatatgaatttgccagga

aaatggaaaccaaaaatgatagggggaattggaggttttatcaaagtaagacagtatgaa

cagatacccatagaaatctgtggacataaagctataggtacagtattagtaggacctaca

cctgtcaacataattggaagaaatctgttgactcagattggttgtactttaaattttcca

atcagtcctattgaaactgtaccagtaaaactaaagccaggaatggatggcccaaaggtt

aaacaatggccattgacaaaagaaaaaataraagcattaacagcaatttgtgatgaaatg

gaaaaggaaggaaaaattacaaaaattgggcctgaaaatccatacaacactccaatattt

gccataaaaaagaaagacagtactaagtggagaaaattagtagatttcagggaactcaat

aaaagaactcaagatttttgggaagttcaattaggaataccacacccagcaggattaaaa

aagaaaaaatcagtgacagtgctggatgtgggggatgcatatttttcagttcctttatat

gaagacttcaggaaatatactgcattcaccatacctagtataaacaatgaaacaccaggg

attaggtatcagtacaatgtacttccacagggatggaaaggatcaccagcaatatttcaa

agtagcatgacaaaaatcttagagccttttagaaaacaaaatccagacatagtcatctat

caatatatggatgatttgtatgtaggatctgacttagagatagggcagcatagaacaaaa

atagaggaactgagacaacatttgttgaggtggggatttaccacaccagacaagaaacat

cagaaagaacctccatttctttggatggggtatgagctccatcctgacaaatggacagta

cagcctatacagctgccagtacaagatagctggactgtcaatgatatacaaaagttagtg

ggaaaattaaactgggcaagtcagatttatcctggaattaaagtaaggcaactttgtaaa

ctccttaggggggccaaagcactaacagacatagtaccactaactgaagaagcagaat--

------------------------------------------------

>CRF07BC.G-HZ130198

cctcaaatcactctttggcaacgacccattgttacagtaaagataggggggcaattaaag

gaagctctattagatacaggagcagatgatacagtattagaagacatgaatttgccaggg

aaatggaaaccaagaatgatagggggaattggaggttttatcaaagtaagacagtatgaa

cagatacccgtagaaatctgtggacataaagctataggtacagtattagtaggacctaca

cctgtcaacataattggaagaaatctgttgactcagcttggttgtaccttaaattttcca

atcagtcctattgaaactgtaccagtaaaactgaagccaggaatggatggcccaaaggtt

aaacaatggccattgacaaaagagaaaatagaagcattaacagcaatttgtgatgaaatg

gaaaaggaaggaaaaatctcaaaaattgggcctgaaaatccatacaacactccaatattt

gcmataaaaaagaaagacagtactaagtggagaaaattagtagatttcagggaactcaat

aaaagaactcaagatttttgggaagttcaattaggaataccacacccagcaggattaaaa

aagaaaaaatcagtgacagtactagatgtgggagatgcatatttttcagtccctttagat

gaaagctttagaaagtatactgcattcaccatacctagtataaacaatgaaacaccaggg

atyaggtatcagtacaatgtacttccacagggatggaaaggatcaccagcaatatttcaa

agtagcatgacaaaaatcttagagccctttagaaaacaaaatccagagatggttatctat

caatacatggatgacttgtatgtaggatctgatttagaaatagggcaacatagaacaaaa

atagatgagctaagagctcacctattgagctggggatttactacaccagacaaaaagcat

cagaaggaacctccatttctttggatggggtatgaactccatcctgacaaatggacagtc

cagcctatagaactgccagaaaaagacagctggactgtcaatgatatacagaagttagtg

ggaaaactaaattgggcaagtcaaatttatccagggattaaggtaaagcaactgtgtaaa

ctcctcaggggaactaaagcat--------------------------------------

------------------------------------------------

>CRF07BC.G-HZ130199

cctcaaatcactctttggcaacgaccccttgtcacagtaaagataggggggcaattaaag

gaagctctattagatacaggagcagatgatacagtattagaagacatgaatttgccaggg

aaatggaaaccaaaaatgatagggggaattggcggttttatcaaagtaagacagtatgaa

cagatacccatagaaatctgtggacatacagttataggtacagtattagtaggacctacr

cctgtcaacataattggaagaaatctgttgactcagcttggttgtactttaaattttcca

atcagtcctattgamactgtaccagtaaaactaaagccaggaatggatggcccmaaggtt

aaacaatggccattgacaaaagagaaaatagaagcattaacagcaatttgtgatgaaatg

gaaaaggaaggaaaaattacaaaaattgggcctgaaaatccatacaacactccaatattt

gccataaaaaagaaagacagtactaagtggagaaaattagtagatttcagggaactcaat

aaaagaactcaagatttttgggaagttcaattaggaataccacacccagcaggattaaaa

aagaaaaaatcagtgacagtgctggatgtgggagatgcatatttttcagttcctttatat

gaagacttyaggaaatatactgcattcacyatacctagtataaacaatgaaacaccaggg

attaggtatcagtacaatgtacttccacagggatggaaaggatcaccagcaatatttcaa

agtagcatgacaaaaatcttagagccttttagaaaacaaaacccagacatagtcatctat

caatacatggatgatttatatgtaggatctgacttagagatagggcagcatagaacaaaa

atagaggaactgagacaacatttgttgaagtggggatttaccacaccagacaagaaacat

cagaaagaacctccatttctttggatggggtatgaactccatcccgacaaatggacagta

cagcctatacagctgccagtacaagatagctggactgtcaatgatatacaaaagttagtg

ggaaaattaaactgggcaagtcagatttatcctggaattaaagtaaggcaactttgtaaa

ctccttagggggaccaaagcactaacagacatagtaccactaactgaagaagcagaat--

------------------------------------------------

>CRF07BC.G-HZ130201

cctcaaatcactctttggcaacgaccccttgttaccataaagataggggggcaattaaag

gaagctctattagatacaggagcagatgatacagtattagaagacatgaatttgccaggg

aaatggaaaccaaaaatgatagggggaattggaggttttattaaagtaagacagtatgaa

cagatacccatagaaatctgtggacataaagctataggtacagtattaataggacctaca

cctgtcaacataattggaagaaatctgttgactcagcttgggtgtactttaaattttcca

atcagtcctattgaaactgtaccagtaaaactaaagccaggaatggatggcccaaaggtt

aaacaatggccattgacaaaagagaaaatagaagcattaacagcaatttgtgatgaaatg

gaaaaggaaggaaaaattacaaaaattgggcctgaaaatccatacaacactccaatattt

gccataaaaaagaaagacagtactaagtggagaaaattagtagatttcagggaactcaat

aaaagaactcaagatttttgggaagttcaattaggaataccacacccagcaggattaaaa

aagaaaaaatcagtgacagtgctggatgtgggggatgcatatttttcagttcctttatat

gaagacttcaggaaatatactgcattcaccatacctagtataaacaatgaaacaccaggg

attaggtatcagtacaatgtacttccacagggatggaaaggatcaccagcaatatttcaa

agtagcatgataaaaatcttagaaccttttagaaaacaaaatccagacatagtcatctat

caatacatggatgatttgtatgtaggatctgacttagaratagggcagcatagarcaaaa

atagaaaaactgagacaacatttgttgagatggggatttaccacaccagacaagaaacat

cagaaagaacctccatttctttggatggggtatgaactccatcctgacaaatggacagta

cagcctatacagctgccagtacaagatagctggactgtcaatgatatacaaaagttagtg

ggaaaattaaactgggcaagtcagatttatcctggaattaaagtaaggcaactttgtaaa

ctccttaggggggccaaagcactaacagacatagtaccactaactgaagaagcagaatta

g-----------------------------------------------

>CRF07BC.G-HZ130206

cctcaaatcactctttggcaacgaccccttgttaccataaagataggggggcaagtaaag

gaagctctattagatacaggagcagatgatacagtattagaagacatgaatttgccaggg

aaatggaaaccaaaaatgatagggggaattggaggttttatcaaagtaagacagtatgaa

cagatacccatagaaatctgtggacataaagctataggtacagtattagtaggacctaca

cctgtcaacataattggaagaaatctgytgactcagcttggttgtactttaaattttcca

atcagtcctattgaaactgtaccagtaaaactaaagccaggaatggatggcccaaaggtt

aaacaatggccattgacaaaagagaaaatagaagcrttaacagcaatttgtgatgaaatg

gaaaaggaaggaaaaattacaaaaattgggcctgaaaatccatacaacactccaatattt

gccataaaaaagaaagacagtactaagtggagaaaattagtagatttcagggaactcaat

aaaagaactcaagatttttgggaarttcaattaggaataccacacccagcaggattaaaa

aagaaaaaatcagtgacagtgctggatgtgggggatgcatatttttcagttcctttatat

gargacttcaggaaatatactgcattcaccatacctagtataaacaatgaaacaccaggg

attaggtatcagtacaatgtacttccacagggatggaaaggatcaccagcaatatttcaa

agtagcatgacaaaaatcttagagccttttagaaaacaaaatccagacatagtcatctat

caatacatggatgatttgtatgtaggatctgacttagagatagggcagcatagaacaaaa

atagaggaactgagacaacatttgttgaggtggggatttaccacaccagacaagaaacat

cagaaagaacctccatttctttggatggggtatgaactccatcctgacaaatggacagta

cagcctatacagctaccagtaaaagatagctggactgtcaatgatatacaaaagttagtg

ggaaaattaaactgggcaagtcagatttatcctggaatcaaagtaaggcaactttgtaaa

ctccttaggggggccaaagcactaacagacatagtaccactaactgaagaagcag-----

------------------------------------------------

>CRF07BC.G-HZ130248

cctcaaatcactctttggcaacgaccccttgttaccataaagataggggggcaattaaag

gaagctctattagatacaggagcagatgatacagtattagaagacatgaatttgccaggg

aaatggaaaccaaaaatgatagggggaattggaggttttatcaaagtaagacagtacgaa

cagatacccatagaaatctgtggacataaagctataggtacagtattagtaggacctaca

cctgtcaacataattggaagaaatctgttgactcagcttggttgtactttaaattttcca

atcagtcctattgaaactgtaccagtaaaactaaagccaggaatggatggcccaarggtt

aaacaatggccrttgacaaaagagaaaatagaagcattaacagcaatytgtratgaaatg

gaaaaggaaggaaaaattacaaaaattgggcctgaaaatccatacaacactccartattt

gccataaaaaagaaagacagtactaagtggagaaaattagtagatttcagggaactcaat

aaaagaactcaagatttttgggaagttcaattaggaataccacacccagcaggattaaaa

argaaaaaatcagtgacagtgctggatgtgggrgatgcatatttttcagttcctttakat

gaagacttcaggaaatatactgcattcaccatacctagtayaaacaatgaaacaccaggg

attaggtatcagtacaatgtacttccrcagggatggaaaggatcaccagcaatatttcaa

artagcatgacaaaaatcttagagccttttagaaaacaaaatccagacatagtcatctat

caatacatggatgatttgtatgtaggatctgacttagaratagggcagcatagaacaaaa

atagaggaactgagacaacatttgttraggtggggatttaccacaccagacaagaagcat

cagaaagaacctccatttctttggatggggtatgagctccatcctgacaaatggacagta

cagcctgtacagctgccagtacaagatagctggactgtcaatgatatacaaaagttagtg

ggaaaattaaactgggcaagtcagatttatcctggaattaaagtaaggcaactttgtaaa

ctccttaggggggccaaagcactaacagacatagtaccactaactgaagaagcagaatta

g-----------------------------------------------

>CRF07BC.G-HZ130255

cctcaaatcactctttggcaacgaccccttgttaccataaagataggggggcaattaaag

gaagctctattagatacaggagcagatgatacagtattagaagacatgaatttgccaggg

aaatggaaaccaaaaatgatagggggaattggaggttttatcaaagtaagacagtatgaa

cagatacccatagaaatctgtggacataaagctataggtacagtattagtaggacctaca

cctgtcaacataattggaagaaatctgttgactcagcttggttgtactttaaattttcca

atcagtcctattgaaactgtaccagtaaaactaaagccaggaatggatggtccaaaagtt

aaacaatggccattgacaaaagaaaaaatagaagcattaacagcaatttgtgatgaaatg

gaaaaggaaggaaaaattacaaaaattgggcctgaaaatccgtacaacactccaatattt

gccataaaaaagaaagacagtactaagtggagaaaattagtagatttcagggaactcaat

aaaagaactcaagatttttgggaagttcaattaggaataccacacccagcaggattaaaa

aagaaaaaatcagtgacagtgctggatgtgggggatgcatatttttcagttcctttatat

gaagacttcaggaaatatactgcattcaccatacctagtataaacaatgaaacaccaggg

attaggtatcagtacaatgtacttccacagggatggaaaggatcaccagcaatatttcaa

agtagcatgacaaaaatcttagagccttttagaaaacaaaatccagacatagtcatctat

caatacatggatgatttgtatgtaggatctgacttagagatagggcagcatagaacaaaa

atagaggaactgagacaacatttgttgaggtggggatttaccacaccagacaagaaacat

caaaaagaacctccatttctttggatggggtatgaactccatcctgacaaatggacagta

cagcctatacagctgccagtacaagatagctggactgtcaatgatatacaaaagttagtg

ggaaaattaaactgggcaagtcagatttatcctggaattaaagtaaggcaactttgtaaa

ctccttaggggggccaaagcactaacagacatagtaccactaactgaagaagcaga----

------------------------------------------------

>CRF07BC.G-HZ130257

cctcaaatcactctttggcaacgaccccttgttaccataaaaataggggggcaattaaag

gaagctctattagatacaggagcagatgatacagtattagaagacatgaatttgccaggg

aaatggaaaccaaaaatgatagggggaattggaggttttatcaaagtaagacagtatgaa

cagatacccatagaaatctgtggaaaaaaggctataggtacagtattagtaggacctaca

cctgtcaacataattggaagaaatctgttgactcagcttggttgtactttaaattttcca

atcagtcctattgaaactgtaccagtaaaactaaagccaggaatggatggcccaaaggtt

aaacaatggccattgacaaaagagaagatagaagcattaacagcaatttgtgatgaaatg

gaaaaggaaggaaaaattacaaaaattgggcctgaaaatccatacaacactccaatattt

gccataaaaaagaaagacagtactaagtggagaaaattagtagatttcagggaactcaat

aaaagaactcaagatttttgggaagttcaattaggaataccacacccagcaggattaaaa

aagaaaaaatcagtgacagtgctggatgtgggggatgcatatttttcagttcctttatat

gaagacttcaggaaatatactgcattcaccatacctagtataaacaatgaaacaccaggg

attaggtatcagtacaatgtacttccacagggatggaaaggatcaccagcaatatttcaa

agtagcatgacaaaaatcttagagccttttagaaagaagaatccagatatagtcatctat

caatacatggatgatttgtatgtaggatctgacttagagataggacagcatagaacaaaa

atagaggaactgagacaacatttgttgaggtggggatttaccacaccagacaagaaacat

cagaaagaacctccatttctttggatggggtatgaactccatcctgacaaatggacagta

cagcctatacagctgccagtacaagatagctggactgtcaatgatatacaaaagttagtg

ggaaaattaaactgggcaagtcagatttatcctggaattaaagtaagacaactttgtaaa

ctccttaggggggccaaagcactaacagacatagtaccactaactgaagaagcagaa---

------------------------------------------------

>CRF07BC.G-HZ130422

cctcaaatcactctttggcaacgaccccttgttaccataaagataggggggcaattaaag

gaagctctattagatacaggagcagatgatacagtgttagaagatatgaatttgcaaggg

aaatggaagccaaaaatgatagggggaattggaggttttatcaaagtaagacagtatgaa

gagatactcatagaaatctgtggacataaagttataggtacagtattggtaggacctaca

cctgtcaacataattggaaggaatctgttgactcagcttggttgtactttaaattttcca

atcagtcctattaaaactgtaccagtaaaactgaagccaggaatggatggcccaaaggtt

aaacaatggccattgacaaaagagaaaatagaagcattagcagcaatttgtgatgaaatg

gaaaaggaaggaaaaattacaaaaattgggcctgaaaatccatacaacactccaatattt

gccataaaaaagaaagacagtactaagtggagaaaattagtagatttcagggaactcaat

aaaagaactcaagatttttgggaagttcaattaggaataccacacccagcaggattaaaa

aagaaaaaatcagtgacagtgctggatgtgggggatgcatatttttcagttcctttatat

gaagacttcaggaaatatactgcattcaccatacctagtataaacaatgaaacaccaggg

attaggtatcagtacaatgtacttccacagggatggaaaggatcaccagcaatatttcaa

agtagcatgacaaaaatcttagagccttttagaaaacaaaatccagacatagtcatctat

caatacatggatgatttgtatgtaggatctgacttagagatagggcaacatagaacaaaa

atagaggaactgagacaacatttgttgaggtggggatttaccacaccagacaagaaacat

cagaaagaacctccatttctttggatggggtatgaactccatcctgacaaatggacagta

cagcctatacagctgccagtgcaagatagctggactgtcaatgatatacaaaagttagtg

ggaaaattaaactgggcaagtcagatttatcctggaattaaagtaaggcaactttgtaaa

ctccttaggggggccaaagcactaacagacatagtaccactaactgaagaagcagaatt-

------------------------------------------------

>CRF07BC.G-HZ130424

cctcaaatcactctttggcaacgaccccttgtcacaataaagataggggggcaattaarg

gaagctctattagatacaggagcagatgatacagtattagaagacatgaatttgccagga

aaatggaaaccaaaaatgatagggggaattggaggttttatcaaagtaagacartatgaa

cagatacccrtagaaatctgtggacataaagcaataggtacagtattagtaggacctaca

cctgtcaacataattggaagaaatctgttgactcarattggttgtactttaaattttcca

atcagtcctattgaaactgtaccagtaaaactaaagccaggaatggatggcccaaaggtt

aaacaatggccattgacaaaagaaaaratagaagcattaacagcaatttgtgatgaratg

gaaaaggaaggaaaaattacaaaaattgggcctgaaaatccatacaacactccaatattt

gccataaaaaagaaagayagtactaagtggagaaaattagtagatttcagggaactcaat

aaaagaactcaagatttttgggaagttcaattaggaataccacacccagcaggattaaaa

aagaaaaaatcagtgacagtgctrgatgtgggggatgcatatttttcagttcctttatat

gaagacttcaggaartatactgcattcaccatacctagtataaacaatgaaacaccaggg

attaggtatcagtacaatgtacttccacagggatggaaaggatcaccagcaatatttcaa

agtagcatgacaagaatcttagagccttttagaaaacaaaatccagacatagtcatctat

caatacatggatgatttgtatgtaggatctgacttagagataggrcagcatagaacaaaa

atagaggaactgagacarcatttgttgagrtggggatttaccacaccagacaagaaacat

cagaaagarcctccatttctttggatggggtatgarctccatcctgacaaatggacagta

cagcctatacarctrccagtacaagatagctggactgtcaatgatatacaaaagttagtg

ggaaaattaaactgggcaagtcagatttatcctggaattaaagtaaggcaactttgtaaa

ctccttaggggggccaaagcactaacagacatagtaccactaactgaagaagcagaatta

gaa---------------------------------------------

>CRF07BC.G-HZ130438

cctcaaatcactctttggcaacgaccccttgtcacaataaagataggggggcaattaaag

gaagctctattagatacaggagcagatgatacagtattagaagacatgaatttgccaggg

aaatggaaaccaaaaatgatagggggaattggaggttttatcaaagtaagacagtatgaa

cagatacccatagaaatctgtggacataaagctataggtacagtattartaggacctaca

cctgtcaacataattggaaggaatctgttgactcagcttggttgtactttaaattttcca

atcagtcctattgaaactgtaccagtaaaaytaaagccaggaatggatggcccaaaggtt

aaacaatggccattgacaaaagagaaaatagaagcattaatagcaatttgtgatgaaatg

gaaaaggaaggaaaaattacaaaaattgggcctgaaaatccatacaacactccaatattt

gccataaaaaagaaagacagtactaagtggagaaaattagtagatttyagggaactcaat

aaaagaactcaagatttttgggaagttcaattaggaataccacacccagcaggattaaaa

aagaaaaaatcagtaacagtgctggatgtgggggatgcatatttttcagttcctttatat

gaagacttcaggaaatatactgcattcaccatacctagtatgaacaatgaaacaccaggg

attagrtaycagtataatgtacttccacagggatggaaaggatcaccagcaatatttcaa

agtagcatgacaaaaatcttagagccttttagaaaacaaaatccagacatagtcatctat

caatacatggatgatttgtatgtrggatctgacttagagatagggcagcatagaacaaaa

atagaggaactgagacaacatttgttgmrgtggggatttaccacaccagacaagaaacat

cagaaagaacctccatttctttggatggggtatgaactccatcctgacaaatggacagta

cagcctatacagytgccagtacaagatagctggactgtcaatgatatacaaaagttagtg

ggaaaattaaactgggcaagtcaratttatcctggaattaaagtaaggcaactttgtaaa

ctccttaggggrgccaaagcactaacagacatagtaccactaactgaagaagcag-----

------------------------------------------------

>CRF07BC.G-HZ130439

cctcaaatcactctttggcaacgaccccttgttaccataaagataggggggcaattaaag

gaagctctattagatacaggagcagatgatacagtattagaggacatgaatttgccaggg

aaatggaaaccaaaaatgatagggggaattggaggttttatcaaagtaagacagtatgaa

cagatacccatagaaatttgtggacataaagctataggtacagtattaataggacctaca

cctgtcaacataattggaagaaatctgttgackcagcttggttgtactttaaattttcca

atcagtcctattgaaactataccagtaaaactaaagccaggaatggatggcccaaaggtt

aaacaatggccattgacaaaagagaaaatagaagcattaacagcaatttgtgatgaaatg

gaaaaggaaggaaaaattacaaaaattgggcctgaaaatccatacaacactccaatattt

gccataaaaaagaaagacagcactaagtggagaaaattagtagayttcagggaactcaat

aaaaggactcaagatttttgggaagttcaattaggaataccacacccagcaggattaaaa

aagaaaaaatcagtgacagtactggatgtgggggatgcatatttttcagttccyttatat

gaagatttcaggaaatatactgcattcaccatacctagtataaacaatgaaacaccaggg

attaggtatcagtacaatgtacttccacagggatggaaaggatcaccagcaatatttcaa

agtagcatgacaaaaatcttagagccttttagaaaacaaaatccagacatagtcatctat

caatacatggatgatttgtatgtaggatctgacttagagatagggcagcacagaacaaaa

atagaggaactgagacaacatttgttgaagtggggatttaccacaccagacaagaaacat

cagaaagaacctccatttctttggatggggtatgaactccatcctgacaaatggacagta

cagcctatacagctgccggtacaagatagctggactgtcaatgatatacaaaagttagtg

ggaaaattaaactgggcaagtcagatttatcctggaattaaagtaaggcaactttgtaaa

ctccttaggggggccaaagcactaacagacatagtaccactaactgaagaagcagaa---

------------------------------------------------

>CRF07BC.G-HZ130491

cctcagatcactctttggcaacgacccctygtcacaataaagataggggggcaattaaag

gaagctctattagatacaggagcagatgatacagtrttagaagacatgaatttgccaggr

aaatggagaccaaaaatgatagggggaattgggggttttatcaargtaaracagtatgaa

carrtacccatagaaatctgtggacacaaagctataggtacagtattagtaggacctaca

cctgtcaacataattggaagaaatctgttgactcagmttggttgcactttaaattttcca

atcagtccyattgaaactgtaccagtaaaattaaagccaggaatggatggccctaaggtt

aaacaatggccattgacagaggagaaaataaaagcattaacagcaatttgtgatgaaatg

gagaaggagggaaaaattacaaaaattgggcctgaaaatccatataacactccaatattt

gccatmaaaaagaaggacagtactaagtggagaaaattagtrgatttcagggaactcaat

aaaagaactcaagatttttgggaagttcaattaggaataccacacccggcagggttaaaa

aagaaaaaatcagtgacagtrctggatgtgggggatgcatatttytcagttcctttatat

gaagacttcaggaagtatactgcattcaccatacctagtacaaacaatgaaacaccaggg

attaggtatcagtacaatgtactcccacagggatggaaaggatcaccagcaatattccaa

agtagcatgacaaaaatcttagagccttttagaaaacaaaatccagacatagctatctat

caatacatggatgatttgtatgtaggatctgacttagagatagggcagcatagaacaaaa

atagaggaactgagacaacatttgttggggtggggattaaccacrccagayaagaaacat

cagaaagaaccyccatttctktggatggggtatgaactccatcctgacaaatggacagta

cagcctatacagctaccagaaaargatagctggactgtcaatgatatacaaaagttagtg

ggaaaattaaactgggcaagtcaratttatcctggaattaaagtaaggcaactttgtaaa

ctccttaggggggccaaagcactaacagacatag--------------------------

------------------------------------------------

>CRF07BC.G-HZ130496

cctcaaatcactctttggcaacgaccccttgttacaataaagataggggggcaagtaaag

gaagctctattagatacaggagcagatgatacagtattagaagaaatgaatttgccaggg

aaatggaaaccaaaaatgatagggggaattggaggttttatcaaagtaagacagtatgaa

caggtccccttagaaatctgtggacataaagctataggtacagtattartaggacctaca

cctgtcaacataattggaagaaatctgttgactcagcttggttgtaccttaaattttcca

atcagtcctattgaaactgtaccagtaavactaaagccaggaatggatggcccaaaggtt

aaacaatggccattgacaaaagaraaaatagaagcattaacagcaatctgtgatgaaatg

gaaaaggaaggaaaaattacaaaaattgggcctgaaaatccatacaacactccaatattt

gccataaaaaagaaagacagtactaagtggagaaaattagtagatttcagggaactcaat

aaaagaactcaagatttttgggargttcaattaggaataccacacccagcaggattaaaa

aagaaaaaatcagtgacagtrctrgatgtgggggatgcatatttttcagttcctttacat

gaagacttcaggaaatatactgcattcaccatacctagtataaacaatgaaacaccaggg

atwaggtatcagtacaatgtacttccrcagggatggaaaggatcaccagcaatatttcaa

agtagcatgacaagaatcttagagccttttagaaaacaaaatccagacatagtcatctat

caatacatggatgatttgtatgtaggatctgacttagaratagggcagcatagaacaaaa

atagaggaactgagacaacatttgttgaggtggggatttaccacaccagacaagaaacat

cagaaagaacctccatttctttggatggggtatgaactccatcctgacaaatggacagta

cagcctatacaactgccagtacaagatagctggactgtcaatgatatacaaaagttagtg

ggaaaattaaactgggcaagtcagatttatcctggaattaaagtaagrcaactttgtaaa

ctccttaggggggccaaagcactaacagacatagtaccactaactgaagaagcagaa---

------------------------------------------------

>CRF07BC.G-HZ130525

cctcaaatcactctttggcaacgaccccttgttaccataaagatagggggacaatyaaag

gaagctctattagatacaggagcagatgatacagtattagaagacatgaatttgccaggg

aaatggaaaccaaaaatgatagggggaattggaggttttatcaaagtaagacaatatgaa

cagatacccatagaaatctgtggacataaagctataggtacagtattagtaggacctaca

cctgtcaacataattgggaggaatctgttgactcagcttggttgtactttaaattttcca

attagtcctattgaaactgtaccagtaaaattaaagccaggaatggatggcccaaaggtt

aaacaatggccattaacaaaagaaaaaatagaagcattaacagcaatttgtgatgaaatg

gaaaaggaaggaaaaattacaaaaattgggcctgaaaatccatacaacactccaatattt

gccataaaaaagaaagacagtactaagtggagaaaattagtagatttcagggaactcaat

aaaagaactcaagatttttgggaagttcaattaggaataccacatccagcaggattaaaa

aagaaaaaatcagtgacagtgctggatgtgggagatgcatatttttcagttcctttatat

gaagacttcaggaaatatactgcattcaccatacctagtataaataatgaaacaccaggg

attagatatcagtacaatgtacttccacaaggatggaaaggatcaccagcaatatttcaa

agtagcatgacaaaaatcttagagccttttagaaaacaaaatccagacatagtcatctat

caatacatggatgatttgtatgtaggatctgacttagagatagggcagcatagaacaaaa

atagaggaactgagacaacatttgttgaggtggggatttaccacaccagacaagaaacat

cagaaagaacctccatttctttggatggggtatgaactccatcctgacaaatggacagta

cagcctatacagttgccagtacaagatagctggactgtcaatgatatacaaaagttagtg

ggaaaattaaactgggcaagtcagatttatcctggaattaaagtaaggcaactttgtaaa

ctccttaggggggccaaagcactaacagacatagtaccactaactgaagaagcagaatta

a-----------------------------------------------

>CRF07BC.G-HZ130528

cctcaaatcactctttggcaacgaccccttgttaccataaagataggggggcaattaaag

gaagctctattagatacaggagcagatgatacagtattagaagacatggatttgccaggg

aaatggaaaccaaaaatgatagggggaattggaggttttatcaaagtaagacagtatgaa

cagatacccatagaaatytgtggacataaagctataggtacagtattagtaggacctaca

cctgtcaacataattggaagaaatctgttgactcagcttggttgtaccttaaattttcca

atcagtcctattgaaactrtaccagtaaaaytaaagccaggaatggatggcccaaaggtt

aaacaatggccattgacaaaagagaaaatagaagcattaacagcaatttgtgatgaaatg

gaaaaggaaggaaaaattacaaaaattgggcctgaaaatccatacaacactccaatattt

gccataaaaaagaaagacagtactaagtggagaaaattagtagatttcagggaactcaat

aaaagaactcaagatttttgggaagttcaattaggaataccacacccagcaggattaaaa

aagaaaaaatcagtgacagtgctggatgtgggggatgcatatttttcagttcctttatat

gaagacttcaggaaatatactgcattcaccatacctagtataaacaatgaaacaccaggg

attaggtatcagtacaatgtacttccacagggatggaaaggatcaccagcaatatttcaa

agtagcatgacaaaaatcttagagccttttagaaaacaaaatccagacatagtcatctat

caatacatggatgatttgtatgtaggatctgacttagagatagggcaacatagaacaaaa

atagaggaactgagacaacatttgttgagrtggggatttaccacaccagacaagaaacat

cagaaagaacctccatttctttggatggggtatgaactccatcctgacaaatggacagta

cagcctatacagctgccagtacaagatagctggactgtcaatgatatacaaaagttagtg

ggaaaattaaactgggcaagtcagatttatcctggaattaaagtaaggcaactttgtaaa

ctccttaggggggccaaagcactaacagacatagtaccactaactgaagaagcagaa---

------------------------------------------------

>CRF07BC.G-HZ130577

cctcaaatcactctttggcaacgaccccttgtcacaataaagataggggggcaattaaag

gaagctctactagatacaggagcagatgatacagtattagaagacatgaatttgccaggg

aaatggaaaccaagaatgatagggggaattgggggttttatcaaagtaagacagtatgaa

cagatacccatagaaatctgtggacataaagctgtaggtacagtattaataggacctaca

cctgtcaacataattggaaggaatctgttgactcagcttggttgtactttaaattttcca

atcagtcctattgaaactgtaccagtaaaactaaaaccaggaatggatggcccaaaggtt

aaacaatggccattgacaaaagagaaaatagaagcattaacagcaatttgtgatgaaatg

gaaaaggaaggaaagattacaaaaattggacctgaaaatccatacaacactccaatattt

gccataaaaaagaaagacagtactaagtggagaaaattagtagatttcagggaactcaat

aaaagaactcaagatttttgggaagttcaattaggaataccacacccagcaggattaaaa

aagaaaaaatcagtgacagtgctggatgtgggagatgcatatttttcagttcctttacat

gaagacttcaggaaatatactgcattcaccatacctagtataaacaatgaaacaccaggg

attaggtatcagtacaatgtacttccacagggatggaaaggatcaccagcaatatttcaa

agtagcatgacaaagatcttagagccttttagaaaacaaaacccagacatagtcatctat

caatacatggatgatttatatgtaggatctgacttagagatagggcagcatagagcaaaa

atagaggaactgagacaacatttgttgaggtggggatttaccacaccagacaagaaacat

cagaaggaacctccatttctttggatggggtatgaactccatcctgacaaatggacagta

cagcctatacagctaccagtacaagatagctggactgtcaatgatatacaaaagttagtg

ggaaaattaaattgggcaagtcagatttatcctggaattaaagtaaggcaactttgtaaa

ctccttagggggaccaaagcactaacagatatagtaccactaactgaagaagcagaat--

------------------------------------------------

>CRF07BC.G-HZ130578

cctcaaatcactctttggcaacgaccccttgttaccataaagataggggggcaagtaaag

gaagctctattagatacaggagcagatgatacagtattagaagacatgaatttgccaggg

aaatggaaaccaaaaatgatagggggaattggaggttttatcaaagtaagacagtatgaa

cagatacccatagaaatctgtggacatagagctataggtacagtattagtaggacctaca

cctgtcaacataattggaagaaatctgttgactcagcttggttgtactttaaattttcca

atcagtcctattgaaactgtaccagtaaarctaaagccaggaatggatggcccaaaggtt

aaacaatggccattgacaaaagagaaaatagaagcattaacagcaatttgtgatgaaatg

gaaaaggaaggaaaaattacaaaaattgggcctgaaaatccatacaacactccaatattt

gccataaaaaagaaagacagtactaagtggagaaaattagtagatttcagggaactcaat

aaaagaactcaagatttttgggaagttcaattaggaataccacacccagcaggattaaaa

aggaaaaaatcagtgacagtgctggatgtgggggatgcatatttttcagttcctttatat

gaagacttcaggaaatatactgcattcaccatacctagtgtaaacaatgaaacaccaggg

actaggtatcagtacaatgtacttccacagggatggaaaggatcaccagcaatatttcaa

agtagtatgacaaaaatcttagaaccttttagaaaacaaaatccagacatagtcatctat

caatacatggatgatttgtatgtaggatctgacttagagatagggcagcatagarcaaaa

atagargaactaagacaacatttgttgaggtggggatttaccacaccagacaagaaacat

cagaaagaacctccatttctttggatggggtatgaactccatcctgacaaatggacagta

cagcctatacagctgccagtacaagatagctggactgtcaatgatatacaaaagttagtg

ggaaaattaaactgggcaagtcagatttatcctggaattagagtaaggcaactgtgtaaa

ctccttaggggggccaaagcactaacagacatagtaccattaactgaagaagcagaa---

------------------------------------------------

>CRF07BC.G-HZ130585

cctcaaatcactctttggcaacgaccccttgttaccataaagataggggggcaattgaag

gaagctctattagatacaggagcagatgataccgtattagaagaaatgaatttgccaggg

aaatggaaaccaaaaatgatagggggaattggaggttttatcaaagtaagacagtatgaa

cagatacccatagaaatttgtggacataaggctataggtacagtattagtaggacctaca

cctgtcaacataattggaagaaatctgttgactcagcttggttgtactttaaattttcca

atcagtcctattgacactgtaccagtaaaactaaagccaggaatggatggcccaaaggtt

aaacaatggccattgacaaaagagaaaatagaagcattaacagcaatttgtgatgaaatg

gaaaaggaaggaaaaattacaaaaattgggcctgaaaatccatacaatactccaatattt

gccataaaaaagaaagacagtactaagtggagaaaattagtagatttcagggaactcaat

aaaaggactcaagatttttgggaagttcaattaggaataccacacccagcaggattaaaa

aagaaaaaatcagtgacagtgctagatgtgggggatgcatatttttcagttcctttatat

gaagacttcaggaaatataccgcgttcaccatacctagtacaaacaatgaaacaccagga

attaggtatcagtacaatgtacttccacagggatggaaaggatcaccagcaatatttcaa

agtagcatgacaagaatcttagagccttttagaaaacaaaatccagacatagtcatctat

caatacatggatgatttgtatgtaggatctgacttagagataggacagcatagaacaaaa

atagaggaactgagacaacatttgttgaggtggggatttaccacaccagacaagaaacat

cagaaagaacctccatttctttggatggggtatgaactccatcctgacaaatggacagta

cagcctatacagctgccagtacaagatagctggactgtcaatgatatacaaaagttagtg

ggaaaattaaactgggcaagtcagatttatcctggaattaaagtaagacaactttgtaaa

ctccttaggggggccaaagcactaacagacatagtaccactaactgaagaagcagaatta

gaaa--------------------------------------------

>CRF07BC.G-HZ130609

cctcaaatcactctttggcaacgaccccttgtcacaataaagatagggggacaattaaar

gaagctctattagatacaggagcagatgatacagtattagaagacatgaatttgccaggg

aaatggaaaccaaaaatgatagggggaattggaggttttatcaaagtaagacagtatgaa

cagatacccatagaaatctgtggacataaagctataggtacagtmttagtaggacctaca

cctgtcaatataattggaagaaatttgttgactcagcttggttgtactttaaattttcca

atcagtcctattgaaactgtaccagtaaaactaaagccaggaatggatggcccaaaggtt

aaacaatggccattgacaagagagaaaatagaagcattaacagcaatttgtgaggaaatg

gaaaaagaaggaaaaattacaaaaattgggcctgaaaatccatacaacactccaatattt

gccataaaaaagaaagacagtactaagtggagaaaattagtagatttcagggagctcaat

aaaagaactcaagacttttgggaagttcaattaggaataccacacccagcaggattaaaa

aagaaaaaatcagtgacagtgctggatgtgggggatgcatacttttcagttcctttatat

gaagacttcaggaaatatactgcattcaccatacctagtacaaacaatgaaacaccaggg

attaggtatcagtacaatgtacttccacagggatggaaaggatcaccagcaatatttcaa

agtagcatgacaaaaatcttagagccttttagaaaacaaaatccagacatagtcatctat

caatacatggatgatttgtatgtaggatctgacttagagatagggcagcatagaacaaaa

atagaggaactgagacaacatttgttgaggtggggatttaccacaccagacaagaaacat

cagaaagaacctccatttctttggatggggtatgaactccatcctgacaaatggacagta

cagcctrtacagttgccartacaagatagctggactgtcaatgatatacaaaagttagtg

ggaaaattaaactgggcaagtcagatttatcctggaattaaagtaaggcaactttgtaaa

ctccttaggggggccaaagcactaacagacatagtaccactaactgaagaagcagaatta

g-----------------------------------------------

>CRF07BC.G-HZ130630

cctcaaatcactctttggcagcgaccccttgttaccataaagataggggggcaattaaag

gaagctctattagatacaggagcagatgatacagtattagaagacatgaatttgccaggg

aaatggaaaccaaaaatgatagggggaattggaggttttatcaaagtaagacagtatgaa

cagatacccatagaaatctgtggacataaagctataggtacagtattagtaggacctaca

cctgtcaacataattggaagaaatctgttgactcagcttggttgtactttaaatttccca

attagtcctattgaaactgtaccagtaaaactaaagccaggaatggatggcccaaaggtt

aaacaatggccattgacaaaagagaaaatagaagcattaacagcaatttgtgatgaaatg

gaaaaggaaggaaaaattacaaaaattgggcctgaaaatccatacaacactccaatattt

gccataaaaaagaaagacagtactaagtggagaaaattagtagatttcagggaactcaat

aaaagaactcaagatttttgggaagttcaattaggaataccacacccagcaggattaaaa

argaaaaaatcagtgacagtgctggatgtgggggatgcatatttttcagttcctttatat

gaagacttcaggaaatatactgcattcaccatacctagtataaacaatgaaacaccaggg

attaggtatcagtacaatgtacttccacagggatggaaaggatcaccagcaatatttcaa

agtagcatgacaaaaatcttagagccttttagaaaacaaaatccagacatagtcatctat

caatacatggatgatttgtatgtaggatctgacttagagataggacagcatagaacaaaa

atagaggaactgagacaacatttgttgaggtggggatttaccacaccagacaagaaacat

cagaaagaacctccatttctttggatggggtatgaactccatcctgacaaatggacagta

cagcctatacagctgccagtacaagatagctggactgtcaatgatatacaaaagttagtg

ggaaaattaaactgggcaagtcagatttatcctggaattaaagtaaggcaactttgtaag

ctccttaggggggccaaagcactaacagacatagtaccactaaccgaagaagcagaatta

gaattggcaga-------------------------------------

>CRF07BC.G-HZ130713

cctcaaatcactctttggcaacgaccccttgttaccataaagataggggggcaattaaag

gaagctctattagatacaggagcagatgatacagtattagaagacatgaatttgccaggg

aaatggaaaccaaaaatgatagggggaattggaggttttatcaaagtaagacaatatgaa

caggtacccatagaaatttgtggacataaagctataggtacagtattaataggacctaca

cctgtcaacataattgggaggaatctgttgactcagcttggttgtactttaaattttcca

attagtcctattgaaactgtaccagtaaaattaaagccaggaatggatggcccaaaggtt

agacaatggccattgacaaaagagaaaatagaagcattaacagcaatttgtaatgaaatg

gaaaaggaaggaaaaattacaaaaattgggcctgaaaatccatacaacactccaatattt

gccataaaaaagaaagacagtactaagtggagaaaattagtagatttcagggaactcaat

aaaagaactcaagacttttgggaagttcaattaggaataccacatccagcaggattaaaa

aagaaaaaatcagtgacagtgctggatgtgggagatgcatatttttcagttcctttatat

gaagacttcaggaaatatactgcattcaccatacctagtataaataatgaaacaccaggg

attagatatcagtacaatgtacttccacagggatggaaaggatcaccagcaatatttcaa

agtagcatgacaaaaatcttagagccttttagaaaacaaaatccagacatagtcatctat

caatacatggatgatttgtatgtaggatctgacttagagatagggcaacatagaacaaaa

atagaggaactgagacaacatttgttgaggtggggatttaccacaccagacaagaaacat

cagaaagaacctccatttctttggatggggtatgaactccatcctgacaaatggacagta

cagcctatacagctgccagtacaagatagctggactgtcaatgatatacaaaagttagtg

ggaaaattaaattgggcaagtcagatttatcctggaattaaagtaaggcaactttgtaaa

ctccttaggggggccaaagcactaacagacatagtaccactaactgaagaagcaga----

------------------------------------------------

>CRF07BC.G-HZ130716

cctcaaatcactctttggcaacgaccccttgttaccataaagataggggggcaattaaag

gaagctctattagatacaggagcagatgatacagtattagaggacatgaatttgccaggg

aaatggaaaccaaaaatgatagggggaattggaggttttatcaaagtaagacagtatgaa

caggtacccatagaaatttgtggacataaagctataggtacagtattaataggacctaca

cctgtcaacataattggaagaaatctgttgactcagcttggttgtactttaaattttcca

atcagtcctattgaaactataccagtaaaactaaagccaggaatggatggcccaaaggtt

aaacaatggccattgacaaaagagaaaatagaagcattaacagcaatttgtgatgaaatg

gaaaaggaaggaaaaattacaaaaattgggcctgaaaatccatacaacactccaatattt

gccataaaaaagaaagacagcactaagtggagaaaattagtagatttcagggaactcaat

aaaaggactcaagatttttgggaagttcaattaggaataccacacccagcaggattaaaa

aagaaaaaatcagtgacagtactggatgtgggggatgcatatttttcagttcctttatat

gaagatttcaggaaatatactgcattcaccatacctagtataaacaatgaaacaccaggg

attaggtatcagtacaatgtrcttccacagggatggaaaggatcaccagcaatatttcaa

agtagcatgacaaaaatcttagagccttttagaaaacaaaatccagacatagtcatctat

caatacatggatgatttgtatgtaggatctgacttagagatagggcagcacagaacaaaa

atagaggaactgagacaacatttgttgaggtggggatttaccacaccagacaagaaacat

cagaaagaacctccatttctttggatggggtatgaactccatcctgacaaatggacagta

cagcctatacagctgccagaacaagatagctggactgtcaatgatatacaaaagttagtg

ggaaaattaaactgggcaagtcagatttatcctggaattaaagtaaggcaactttgtaaa

ctcctwaggggggccaaagcactaacagacatagtaccactaactgaagaagcagaa---

------------------------------------------------

>CRF07BC.G-HZ130719

cctcaaatcactctttggcaacgaccccttgtcacaataaagrtaggggggcaattaaag

gaagctctattagatacaggagcagatgatacagtattagaagacatgaatttgccaggg

aaatggaaaccaaaaatgatagggggaattggaggttttatyaaagtaagacagtatgaa

cagatacccatagaaatctgyggacataaagctataggtacagtattagtaggacctaca

cctatcaacataattggaagaaatatgttgactcagcttggttgtactttaaattttcca

atcagtcctattgaaactgtaccagtaaaactaaagccaggaatggatggcccaaaggtt

aaacaatggccattgacaaaagagaaaatagaagcattaacagcaatttgtgaggaaatg

gaaaaggaaggaaaaattacaaaaattgggcctgaaaatccatacaacactccaatattt

gccataaaaaagaaagacagtactaagtggagaaaattagtagatttcagggaactcaat

aaaagaactcaagatttttgggaagttcaattaggaataccacacccagcaggattaaaa

aagaaaaaatcagtaacagtgctggatgtgggggatgcatatttttcagttcctttatat

gaagacttcaggaaatatactgcattcaccatacctagtacaaacaatgaaacaccaggg

attaggtatcagtacaatgtacttccacagggatggaaaggatcaccagcaatatttcaa

agtagcatgacaaaaatcttagagccttttagaaaacaaaatccagacatagtcatctat

caatacatggatgatttgtatgtaggatctgacttagaaatagggcagcatagaacaaaa

atagaggaactgagacaacatttgttgaggtggggatttaccacaccagacaagaaacat

cagaaagaacctccatttctgtggatggggtatgaactccatcctgacaaatggacagta

cagcctatacagctgccagwacaagatagctggactgtcaatgatatacaaaagttagtg

ggaaaattaaactgggcaagtcagatttatcctggaattaaagtaaggcaactttgtaaa

ctccttaggggagccaaagcaytaacagacatagtaccactaactgaagaagcag-----

------------------------------------------------

>CRF07BC.G-HZ130723

cctcaaatcactctttggcaacgacccctggtcacaataaagataggggggcaattaaag

gaagctctattagatacaggagcagatgatacagtattagaagacatgaatttgccaggg

aaatggaaaccaaaaatgatagggggaattggaggttttatcaaagtaagacagtatgaa

caaatacccatagaaatctgtggacataaagctataggtacagtattagtaggacctaca

cctgtcaacataattggaagaaatctgttgactcagcttggttgtactttaaattttcca

atcagtcctattgaaactataccagtaaaactaaagccaggaatggatggcccaaaggtt

aaacaatggccattgacaaaagagaaaatagaagcattaacagcaatttgtgatgaaatg

gaaaaggaaggaaaaattacaaaaattgggcctgaaaatccatacaacactccaatattt

gccataaaaaagaaagacagtactaagtggagaaaattagtagatttcagggaactaaat

aaaagaactcaagatttttgggaagttcaattaggaataccacacccagcaggrttaaaa

aagaaaaaatcagtgacagtgctggatgtgggggatgcatatttttcagttcctttatat

gaagacttcaggaaatatactgcattcaccatacctagtataaacaatgaaacaccaggg

attaggtatcagtacaatgtacttccacagggatggaaaggatcaccagcaatatttcaa

agtagcatgacaaaaatcttagagccttttagaaaacaaaatccagacataatcatctat

caatacatggatgatttgtatgtaggatctgacttagagatagggcagcatagaacaaaa

atagaggaactgagacaacatttgttgaggtggggatttaccacaccagacaagaaacat

cagaaagaacccccgtttctttggatggggtatgaactccatcctgacaaatggacagta

cagcctatacagctgccagtacaagatagctggactgtcaatgatatacaaaagttagtg

ggaaaattaaattgggcaagtcagatttatcctggaattaaagtaaggcaactttgtaaa

ctccttagggggaccaaagcactaacagacatagtaccactaactgaagaagcag-----

------------------------------------------------

>CRF07BC.G-HZ130726

cctcagatcactctttggcaacgaccccttgttaccataaagataggggggcaattaaag

gaagctctattagatacaggagcagatgatacagtattagaagacatgaatttaccaggg

aaatggaaaccaaaaatgatagggggaattggaggttttatcaaggtaagacagtatgac

caaatacccatagaaatctgtggacataaagctataggtacagtattaataggacctaca

cctgtcaacataattggaagaaatctgttgactcagcttggttgtactttaaattttcca

atcagtcctattgaaactgtaccagtaaaactaaagccaggaatggatggcccaaaggtt

aaacaatggccattgacaaaagagaaaatagaagcattaacagcaatttgtgatgaaatg

gaaaaggaaggaaaaattacaaaaattgggcctgaaaatccatacaacactccaatattt

gccataaaaaagaaagacagtactaagtggagaaaattagtagacttcagggaactcaat

aaaagaactcaagatttttgggaagttcaattaggaataccacacccagcaggattaaaa

aagaaaaggtcagtgacagtgctggatgtgggggatgcatatttttcagttcctttacat

gaagacttcaggaaatatactgcattcaccatacctagtataaacaatgaaacaccaggg

attaggtatcagtacaatgtacttccacagggatggaaaggatcaccagcaatatttcaa

agtagcatgacaaaaatcttagaaccctttagaaaacaaaatccagacatagtcatctat

caatatatggatgatttgtatgtaggatctgacttagagatagggcagcatagaacaaaa

atagaggaactgagacaacatttgttgaagtggggatttaccacaccagacaagaaacat

cagaaagaacctccatttctttggatggggtatgaactccaccctgacaaatggacagta

cagcctatacaactgccagtacaagatagctggactgtcaatgatatacaaaagttagtg

ggaaaattaaactgggcgagtcaaatttatcctggaattaaagtaaggcaactttgtaaa

ctccttaggggggccaaagcactaacagacataataccactaactgaggaagcagaat--

------------------------------------------------

>CRF07BC.G-HZ130727

cctcaratcactctttggcaacgaccccttgttaccataaagataggggggcaattaaag

gaagctctattagatacaggagcagatgatacagtattagaagacatgaatttgccaggg

aaatggaaaccaaaaatgatagggggaattggaggttttatcaargtaagacagtatgac

cagatacccatagaaatctgtggacataaagctataggtacagtattartaggacctaca

cctgtcaacataattggaagaaatctgttgactcagcttggttgtactttaaattttcca

atcagtcctattgaaactgtaccagtaaaactaaagccaggaatggatggcccaaaggtt

aaacaatggccattgacaaaagagaaaatagaagcattaacagcaatttgtgatgaaatg

gaaaaggaaggaaaaattacaaaaattgggcctgaaaatccatacaacactccaatattt

gccataaaaaagaaagacagtactaagtggagaaaattagtagacttcagggaactcaat

aaaagaactcaagatttttgggaagttcaattaggaataccacacccagcaggattaaaa

aagaaaagatcagtgacagtgctggatgtgggggatgcatatttttcagttcctttayat

gaagacttcaggaaatatactgcattcaccatacctagtataaacaatgaaacaccaggg

attaggtatcagtacaatgtacttccacagggatggaaaggatcaccagcaatatttcaa

agtagcatgacaaaaatcttagagccttttagaaaacaaaatccagacatagtcatctat

caatatatggatgatttgtatgtaggatctgacttagagatagggcagcatagaacaaaa

atagaggaactgagacamcatttgttgaagtggggatttaccacaccagacaagaaacat

cagaaagaacctccatttctttggatggggtatgaactccaccctgacaaatggacagta

cagcctatacagctgccagtacaagatagctggactgtcaatgatatacaaaagttagtg

ggaaaattaaactgggcgagtcagatttatcctggaattaaagtaaggcaactttgtaaa

ctccttaggggggccaaagcactaacagacatagtaccactaactgaggaagcagaatta

gaaattggcag-------------------------------------

>CRF07BC.G-HZ130729

cctcaaatcactctttggcaacgaccccttgtcacaataaagataggggggcaattaaag

gaagctctattagatacaggagcagatgatacagtattagaagaaatgaatttaccaggg

aaatggaaaccaaaaatgatagggggaattggaggttttatcaaagtaagacagtatgaa

cagatacccatagaaatctgtggacataaagctataggtacagtattagtaggacctaca

cctgtcaacataattggaagaaatctgttgactcagcttggttgtactttaaattttcca

atcagtcctattgaaactgtaccagtaaaattaaagccaggaatggatgggccaaaggtt

aaacaatggccattgacaaaagagaaaatagaagcattaacagcaatttgtgatgaaatg

gaaaaggaaggaaaaattacaaaaattgggcctgaaaatccatacaacactccaatattt

gccataaaaaagaaagacagtactaagtggagaaaattagtagatttcagggaactcaat

aaaagaactcaagatttttgggaagttcaattaggaataccacacccagcaggattaaaa

aagaaaaaatcagtgacagtgctggatgtgggggatgcatatttttcagttcctttatat

gaagacttcaggaaatatactgcattcaccatacctagtataaacaatgaaacaccaggg

attaggtatcagtacaatgtacttccacagggatggaaaggatcaccagcaatatttcaa

agtagcatgacaaaaatcttagagccttttagaaaacaaaatccagacatagtcatctat

caatacatggatgatttgtatgtaggatctgacttagagataggacagcatagaacaaaa

atagaggaactgagacaacatttgttgaggtggggatttaccacaccagataagaaacat

cagaaagaacctccatttctttggatggggtatgaactccatcctgacaaatggacagta

cagcctatacagctaccagtacaagatagctggactgtcaatgatatacaaaagttagtg

ggaaaattaaactgggcgagtcagatttatcctggaattaaagtaaggcaactttgtaaa

ctccttaggggggccaaagcactaacagacatagtgccactaactgaagaagcagaatta

gaattggcaga-------------------------------------

>CRF07BC.G-HZ130733

cctcaaatcactctttggcaacgaccccttgttaccataaagataggggggcaattaaag

gaagctctattagatacaggagcagatgatacagtattagaagacatgaatttgccaggg

aaatggaaaccaaaaatgatagggggaattggaggttttatcaaagtaagacagtatgaa

cagatacccatagaaatttgtggacataaagctataggtacagtattaataggacctaca

cctgtcaacataattggaagaaatctgttgactcagcttggttgtactttaaattttcca

atcagtcctattgaaactataccagtaaaactaaagccaggaatggatggcccaaaggtt

aaacaatggccgttgacaaaagagaaaatagaagcattaacagcaatttgtgatgaaatg

gaaaaggaaggaaaaattacaaaaattgggcctgaaaacccatacaacactccaatattt

gccataaaaaagaaagacagcactaagtggagaaaattagtagatttcagggaactcaat

aaaaggactcaagatttttgggaagttcaattaggaataccacacccagcaggattaaaa

aagaaaaaatcagtgacagtgctggatgtgggggatgcatatttttcagttcctttatat

gaggatttcaggaaatatactgcattcactatacctagtataaacaatgaaacaccaggg

attaggtaccagtacaatgtacttccacagggatggaaaggatcaccagcaatatttcaa

agtagcatgacaaaaatcttagagccttttagaaaacaaaatccagacatagtcatctat

caatacatggatgatttgtatgtaggatctgacttagagatagggcagcacagaacaaaa

atagaggaactgagacagcatttgttgaagtgggggtttaccacaccagacaagaaacat

cagaaagaacctccatttctttggatggggtatgaactccatcctgacaaatggacagta

cagcctatacagctgccagtacaagatagctggactgtcaatgatatacaaaagttagtg

ggaaaattaaactgggcaagtcagatttatcctggaattaaagtaaggcaactttgtaaa

ctccttaggggggccaaagcactaacagacatagtaccactaactgaagaagcagaatta

ga----------------------------------------------

>CRF07BC.G-HZ130738

cctcaaatcactctttggcaacgaccccttgttaccataaagataggggggcaatcaaaa

gaggctctattagatacaggagcagatgatacagtattagaagacatgaatttaccaggg

aaatggaaaccaaaaatgatagggggaattggaggttttatcaaagtaagacagtatgaa

cagatacccatagaaatctgtggacataaagctgtaggtacagtattagtaggacctaca

cctgtcaacataattggaagaaatctgttgactcagcttggttgtactttaaattttcca

atcagtcctatcgaaactgtaccagtaaaactaaagccaggaatggatggcccaaaggtt

aaacaatggccattgacaaaagagaaaatagaagcattaacagcaatttgtgctgaaatg

gaaaaggaaggaaaaattacaaaaattgggcctgaaaatccatacaacactccaatattt

gccataaaaaagaaagacagtactaagtggagaaaattagtagatttcagggaactcaat

araaaaactcaagatttttgggaggttcaattaggaataccacacccagcaggattaaaa

aagaaaaaatcagtgacagtgctggatgtgggggatgcatatttttcagttcctctatat

gaagacttcaggaaatatactgcattcaccatacctagtataaacaatgaaacaccaggg

attaggtatcagtacaatgtacttccacagggatggaaaggatcaccagcaatatttcaa

agtagcatgacaaaaatcttagagccttttagaaaacaaaatccagacatagtcatctat

caatacatggatgatttgtatgtaggatctgacttagagatagggcagcatagaacaaaa

atagaggaactgagacaacatttgttgaggtggggattcaccacaccagacaagaaacat

cagaaagaacctccatttctttggatggggtatgaactccatcctgacaaatggacagta

cagcctatacagctgccagtacaagatagctggactgtcaatgatatacaaaagttagtg

ggaaaattaaactgggcaagtcagatttatcctggaattaaggtaaggcaactttgtaaa

ctcmttaggggagtcaaagcactaacagacatagtaccactaactgaagaagcagaa---

------------------------------------------------

>CRF07BC.G-HZ130742

cctcaaatcactctttggcaacgaccccttgttacaataaagataggggggcaactaaag

gaagctctattagatacaggagcagatgacacagtattagaagacatgaatttgccaggg

aaatggaaaccaaaaatgatagggggaattggaggttttatcaaagtaagacagtatgaa

cagatacccatagaaatctgtggacataaagctataggtacagtattagtaggacctaca

cctgtcaacataattggaagaaatctgttgactcagcttggttgtactttgaattttcca

atcagtcctattgaaactgtaccagtaaaactaaagccaggaatggatggcccaaaggtt

aaacaatggccattgacaaaagaaaaaatagaagcattaacagcaatttgtgatgagatg

gaaaaggaaggaaaaattacaaaaattgggcctgaaaatccatacaacactccaatattt

gccataaaaaagaaagacagtactaagtggagaaaattagtagatttcagggaactcaat

aaaagaactcaagatttttgggaagttcaattaggaataccacacccagcaggattaaaa

aagaaaaaatcagtgacagtgctggatgtgggggatgcatatttttcagttcctttatat

gaagacttcagaaaatatactgcatttaccatacctagtacaaacaatgaaacaccaggg

attaggtatcagtacaatgtacttccacagggatggaaaggatcaccagcaatatttcaa

agtagcatgacaaaaatcttagagccttttagaaaacaaaatccagacatagtcatctat

caatacatggatgatttgtatgtaggatctgacttagagatagggcagcatagaacaaaa

atagaggaactgagacaacatttgttgaggtggggatttaccacaccagacaagaaacat

cagaaagaacctccatttctttggatggggtatgaactccatcctgacaaatggacagta

cagcctatacagctgccaatacaagatagctggactgtcaatgatatacaaaagttagtg

ggaaaattaaactgggcaagtcagatttatcctggaattaaagtaaggcaactttgtaaa

cttcttaggggggccaaagcactaacagacatagtaccactaactgaagaagcagaat--

------------------------------------------------

>CRF07BC.G-HZ130788

cctcaaatcactctttggcaacgaccccttgtcacaataaagrtaggagggcaattaawg

gaagctctattagatacaggagcagatgatacagtattagaagamattaatttgccaggg

aaatggaaaccaaaaatgataggaggaattggaggttttatcaaagtaagacagtatgaa

cagrtacccatagaaatctgtggacataaagctataggtacagtattaataggacctaca

cctgtcaacataattggaagaaatctgttgactcagcttggttgtactttaaattttcca

atcagtcctattgaaactgtaccagtaaaactaaagccaggaatggatggcccaaaggtt

aaacaatggccattgacaaaagagaaaatagaagcattaacagcaatttgtgatgaaatg

gaaaaggaaggaaaaattacaaaaattgggcctgaaaatccatacaacactccaatattt

gccataaaaaagaaagacagtactaagtggagaaaattagtagatttcagrgaactyaat

aaaagaactcaagatttttgggaagttcaattaggaataccacacccagcaggattaaaa

aagaaaaaatcagtgacagtgctggatgtgggggatgcatatttttcagttcctttacat

gaagacttcaggaaatatactgcattcaccatacctagtataaacaatgaaacaccaggg

attaggtatcagtacaatgtacttccacagggatggaaaggatcaccagcaatatttcaa

artagcatgacaaaaatcttagagccttttagaaaacaaaatccagacatagtcatctat

caatacatggatgatttgtatgtaggatctgacttagagatagggaagcatagaacaaaa

atagaggaactgagacaacatttgttgaggtggggatttaccacaccagataagaaacat

cagaaagaacctccatttctttggatggggtatgaactccatcctgacaaatggacagta

cagcctatacagctgccagtacaagatagctggactgtyaatgatatacaaaagttagtg

ggaaaattaaactgggcaagtcagatttatcctggaattaaagtaaggcaactttgtaaa

ctccttaggggggccaaagcactaacagacatagtaccactaactgaagaagcagaatta

gaaattggcaga------------------------------------

>CRF07BC.G-HZ130790

cctcaaatcactctttggcaacgaccccttgttaccataaagataggggggcaattaaag

gaagctctattagatacaggagcagatgatacagtattagaagacatgaatttgccaggg

aaatggaaaccaaaaatgatagggggaattggaggttttatcaaagtaagacagtatgaa

cagatacccatagaaatctgtggacataaagctataggtacagtattagtaggacctaca

cctgtcaacataattggaagaaatctgttgactcagcttggttgtactttaaattttcca

atcagtcctattgaaactgtaccagtaaaactaaagccaggaatggatggcccaaaggtt

aaacaatggccattgacaaaagagaaaatagaagcattaacagcaatttgtgatgaaatg

gaaaaggaaggaaaaattacaaaaattgggcctgaaaatccatacaacactccaatattt

gccataaaaaagaaagacagtactaagtggagaaaattagtagatttcagggaactcaat

aaaagaactcaagatttttgggaagttcaattaggaataccacacccagcaggattaaaa

aagaaaaaatcagtgacagtgctggatgtgggggatgcatatttttcagttcctttacat

gaagacttcaggaaatatactgcattcaccatacctagtataaacaatgaaacaccaggg

attaggtatcagtacaatgtacttccacagggatggaaaggatcaccagcaatatttcaa

agtagcatgacaaaaatcttagagccttttagaaaacaaaatccagacatagtcatctat

caatacatggatgatttgtatgtaggatctgacttagagatagggcagcatagagcaaaa

atagaggaactgagacaacatttgttgaggtggggatttaccacaccagacaagaaacat

cagaaagaacctccatttctttggatggggtatgaactccatcctgataaatggacagta

cagcctatacagctgccagtacaagatagctggactgttaatgatatacaaaagttagtg

ggaaaattaaactgggcaagtcagatttatcctggaattaaagtaaggcaactttgtaaa

ctccttaggggggccaaagcactaacagacatagtaccactaactgaagaagcagaatta

gaaattggcaga------------------------------------

>CRF07BC.G-HZ130792

cctcaaatcactctttggcaacgaccccttgttaccataaagataggggagcaattaaag

gaagctctattagatacaggagcagatgatacagtattagaagacatgaatttgccaggg

aaatggaaaccaaaaatgatagggggaattggaggttttatcaaagtaagacagtatgaa

cagatacccatagaaatctgtggacataaagttataggtacagtattagtaggacctaca

cctgtcaacataattggaagaaatctgttgactcagcttggttgtactttaaattttcca

atcagtcctattgaaactgtaccagtaaaactaaagccaggaatggatggcccaaaggtt

aaacaatggccattgacaaaagagaaaatagaagcattaacagcaatttgtgatgaaatg

gaaaaggaaggaaaaattacaaaaattgggcctgaaaatccatacaacactccaatattt

gccataaaaaagaaagacagtactaagtggagaaaattagtagatttcagggaactcaat

aaaagaactcaagatttttgggaagttcaattaggaataccacacccagcaggattaaaa

aagaaaaaatcagtgacagtgctggatgtgggggatgcatatttttcagttcctttacat

gaagacttcaggaaatatactgcattcaccatacctagtataaacaatgaaacaccaggg

attaggtatcagtacaatgtacttccacagggatggaaaggatcaccagcaatatttcaa

agtagcatgacaaaaatcttagagccttttagaaaacaaaatccagacatagtcatctat

caatacatggatgatttgtatgtaggatctgacttagagacagggcagcatagaacaaaa

atagaggaactgagacaacatttgttgaggtggggatttaccacaccagacaagaaacat

caaaaagaacctccatttctttggatgggatatgaactccatcctgataaatggacagta

cagcctatacagctgccagtacaagatagctggactgtcaatgatatacaaaagttagtg

ggaaaattaaactgggcaagtcagatttatcctggaattaaagtaaggcaactttgtaaa

ctccttaggggggccaaagcactaacagacatagtaccactaactgaagaagcagaatta

gaaattggcag-------------------------------------

>CRF07BC.G-HZ130793

cctcaaatcactctttggcaacgaccccttgtcacaataaagatagggggacaattaaag

gaagctctattagatacaggagcagatgatacagtattagaagacatgaatttgccaggg

aaatggaaaccaaaaatgatagggggaattggaggttttatcaaagtaagacagtatgaa

gacatacccatagaaatctgtggacataaagctataggtacagtattagtaggacctaca

cctgtcaacataattggaagaaatctgttgactcagcttggttgtactttaaattttcca

atcagccctattgaaactgtaccagtaaaactaaagccaggaatggatggcccaaaggtt

aaacaatggccattgacaaaagagaaaatagaagcattaacagcaatttgtgaggaaatg

gaaaaagaaggaaaaattacaaaaattgggcctgaaaatccatacaacactccaatattt

gccataaaaaagaaagacagtactaagtggagaaaattagtagatttcagggaactcaat

aaaagaactcaagacttttgggaagttcaattaggaataccacacccagcaggattaaaa

aagaaaaaatcagtgacagtgctagatgtgggggatgcatatttttcagttcctttatat

gaagacttcaggaaatatactgcattcaccatacctagtataaacaatgaaacaccaggg

attagatatcagtacaatgtacttccacaaggatggaaaggatcaccagcaatatttcaa

agcagcatgacaaaaatcttagagccttttagaaaacaaaatccagacatagtcatctat

caatacatggatgatttgtatgtaggatctgacttagagatagggcagcatagaacaaaa

atagaggaactgagacaacatttgttgaggtggggatttaccacaccagacaagaaacat

cagaaaraacctccatttctttggatggggtatgaactccatcctgacaaatggacagta

cagcctatacaactgccagtacaagatagctggactgtcaatgatatacaaaaattagtg

ggaaagttaaactgggcaagtcagatttatcctggaattaaagtaagacaactttgtaga

ctccttaggggggccaaagcactgacagacatagtaccactaaccgaagaagcagaatta

gaattggcaga-------------------------------------

>CRF07BC.G-HZ130794

cctcaaatcactctttggcaacgaccccttgttaccataaagataggggggcaattaaar

gaagctctattagatacaggagcagatgatacagtattagaagacatgaatttgccaggg

aaatggaaaccaaaaatgatagggggaattggaggttttatcaaagtaagacagtatgaa

cagatacccatagaaatctgtggacataaagctataggtacagtattartaggacctaca

cctgtcaacataattggaagaaatctgttgactcagattggttgtactttaaattttcca

atcagtcctattgaaactgtaccagtaaaactaaagccaggaatggatggcccaaaggtt

aaacaatggccattgacaaaagagaaaatagaagcattaacagcaatttgtgakgaaatg

gaaaaggaaggaaaaattacaaaaattgggcctgaaaatccatacaacactccaatattt

gccataaaaaagaaagacagtactaagtggagaaaattagtagatttcagggaactcaat

aaaagaactcaagatttttgggaagttcaattaggaataccacacccagcaggattaaaa

aagaaaaaatcagtgacagtgctggatgtgggggatgcatatttttcagttcctttatat

gaagacttcaggaaatatactgcattcaccatacctagtataaacaatgaaacaccaggg

attaggtatcagtacaatgtactkccacagggatggaaaggatcaccagcaatatttcaa

agtagyatgacaaaaatcttagagccgttyagaaaacaaaatccagacatagtcatctat

caatacatggatgatttgtatgtaggatctgacttagaaatagggcagcatagaacaaaa

atagaggaactgagacaacatttgttgaggtggggatttaccacaccagacaagaaacat

cagaaagagcctccatttctttggatggggtatgaactccatcctgacaaatggacagta

cagcctatacagctrccagtacaagatagctggactgtcaatgatatacaaaagttagtg

ggaaaattaaactgggcaagtcagatttatcctggaattaaagtaaggcaactttgtaaa

ctccttaggggggccaaagcactaacagacatagtaccactaacagaagaagcagaatta

gaaattggcaga------------------------------------

>CRF07BC.G-HZ130812

cctcaaatcactctttggcaacgaccccttgtcacaataaagataggggggcaattaaag

gaagctctattagatacaggagcagatgatacagtattagaagacataaatttgccaggr

aaatggaaaccaaaaatgatagggggaattggaggttttatcaaagtaagacagtatgaa

caggtacccatagaaatctgtggacatcaagttataggcacagtattagtaggacctaca

cctgtcaacataattggaagaaatctgttgactcagcttggttgtactttaaattttcca

atcagtcctattgaaactgtaccagtaaaactaaagccaggaatggatggcccaaaggtt

aaacaatggccattgacaaaagaraaaatagaagcattaacagcaatttgtgatgaaatg

gaaaaggaaggaaaaattacaaaaattgggcctgaaaatccatacaacactccaatattt

gccataaaaaagaaggacagtactaagtggagaaaattagtagatttcagggaactcaat

aaaagaactcaagatttttgggaagttcaattrggaataccacacccagcaggattaaaa

cagaaaaaatcagtaacagtgctggatgtgggrgatgcatatttttcagttcctttacat

gaagactttaggaaatatactgcattcaccatacctagtataaacaatgaaacaccaggg

ataaggtatcagtacaatgtacttccacagggatggaaaggrtcaccagcaatatttcaa

agtagcatgacaagaatcttagaaccttttagaaaacagaatccagacatagtcatctat

caatacatggatgatttgtatgtaggatctgacttagagatagggcagcatagaacaaaa

atagaggaactgagacarcatttgttgaggtggggatttaccacaccagacaagaaacat

cagaaagaacctccatttctttggatggggtatgaactccatcctgataaatggacagta

cagcctatacagctgccagtacaagatagctggactgtcaatgatatacaaaagttagtg

ggaaaattaaaytgggcaagtcagatttatcctggaattaaagtaaggcaactttgtaaa

ctccttagggggaccaaagcactaacagacatagtaccactaactgaagaagcagaa---

------------------------------------------------

>CRF07BC.G-HZ130925

cctcaaatcactctttggcaacgaccccttgttgccataaagataggggggcaattaaag

gaagctctattagatacaggagcagatgatacagtattagaagatatggatttgccaggg

aaatggaaaccaaaaatgataggaggaattggaggttttatcaaagtaagacagtatgaa

cagatacccatagaaatctgtggacataaagctataggtacagtattagtaggacctacc

cctgtcaacataattggaagaaatctgttgactcagcttggttgtactttaaattttcca

atcagtcctattgaaactgtaccagtaaaactaaagccaggaatggatggcccaaaggtt

aaacaatggccattgacaaaagagaaaatagaagcattaacagcaatttgtgatgaaatg

gaaaaggaaggaaaaattacaaaaattgggcctgaaaatccatacaacactccaatattt

gccataaaaaagaaagacagtactaagtggagaaaattagtagatttcagggaacttaat

aaaagaactcaagatttttgggaagttcaattgggaataccacacccagcaggattaaaa

aagaaaaaatcagtgacagtgctggatgtgggagatgcatatttttcagttcctttatat

gaagatttcaggaaatatactgcatttaccatacctagtataaacaatgaaacaccaggg

rttaggtatcagtacaatgtacttccacagggatggaaaggatcaccagcaatatttcaa

agtagcatgacaaaaatcttagagccttttagaaaacaaaatccagacatagtcatttat

caatacatggatgatttgtatgtaggatctgacttagagatagggcagcatagagyaaaa

atagaggaactgagacaacatttgttgaggtggggatttaccacaccagacaaaaaacat

cagaaagaacctccatttctttggatggggtatgaactccatcctgacaaatggacagta

cagcctatacagctgccagtacaagatagctggactgtcaatgatatacaaaagttagtg

ggaaaattaaactgggcaagtcagatttatcctggaattaaagtaagacaactttgtaaa

ctccttagggggaccaaagcactaacagacatagtaccactaactgaagaagcagaatta

g-----------------------------------------------

>CRF07BC.G-HZ130947

cctcaaatcactctttggcaacgaccccttgtcacaataaaaataggaggacagctaaga

gaagctctattagatacaggagcagatgatacagtattagaagatataaatttgccagga

aaatggaaaccaaaaatgatagggggaattggaggttttataaaagtaaggcaatatgat

cagataactatagaaatttgtggaaaaaaggctataggtacagtgttagtaggacctaca

cctgtcaacataattggacgaaatatgttgactcagcttggttgtactttaaatttccca

attagtcctattgacactrtaccagtaacattaaagccaggaatggatggaccaaaggtt

aaacaatggccattgacaaaagagaaaatagaagcattaacagcaatttgtgatgaaatg

gaaaaagaaggaaaaattacaaaaattgggcccgaaaatccatacaacactccaatattt

gctataaaaaagaaagacagtactaagtggagaaaattagtagatttcagggaactcaat

aaaagaactcaagatttttgggaagttcaattaggaataccacacccagcaggattaaaa

aagaaaaaatcagtgacagtgctggatgtgggggatgcatatttttcagttcctttatat

gaagacttcaggaaatatactgcattcaccatacctagtataaacaatgaagcaccaggg

attaggtatcagtacaatgtacttccacagggatggaaaggatcaccagcaatatttcaa

agtagcatgacaaaaattttagagccttttagaaaacaaaatccagacatagtcatctat

caatacatggatgatttgtatgtaggatctgacttagagatagggcagcatagaataaaa

atagaggaactgagacaacatttgttgaggtggggatttactacaccagacaagaaacat

cagaaagaacctccatttctttggatggggtatgaactccatcctgacaaatggacagta

cagcctatacagctgccagaaaaagatagctggactgtcaatgatatacaaaagttagtg

ggaaaattaaactgggcaagtcagatttatcctggaattaaagtaaggcaactttgtaaa

ctccttagggggrccaaagcactaacagacatagtaccactaactgaagaagcagaatta

gaa---------------------------------------------

>CRF07BC.G-HZ130961

cctcaaatcactctttggcaacgaccccttgtcacaataaagataggggggcaattaaag

gaagctctattagatacaggagcagatgatacagtattagaagacatgaatttgccaggg

aaatggaaaccaaaaatgatagggggaattggaggttttatcaaagtaagacagtatgaa

cagatacccatagaaatctgtggacataaagctataggtacagtattagtaggacctaca

cctgtcaacataattggaaggaatctgttgactcagcttggttgtactttaaattttcca

atcagtcctattgaaactgtaccagtaaaactaaagccaggaatggatggcccaaaggtt

aaacaatggccattgacaaaagagaaaatagaagcattaacagcaatttgtgatgaaatg

gaaaargaaggaaaaattacaaaaattgggcctgaaaatccatacaacactccaatattt

gccataaaaaagaaagacagtactaagtggagaaaattagtagatttcagggaactcaat

aaaagaactcaagatttttgggaagttcaattaggaataccacacccagcaggattaaaa

aagaaaaaatcagtgacagtgctggatgtgggggatgcatatttttcagttcctttatat

gaagacttcaggaaatatacwgcattcaccatacctagtataaacaatgaaacgccaggg

atyaggtatcagtacaatgtacttccacagggatggaaaggatcaccagcaatatttcaa

agtagcatgacaaraatcttagaaccttttagaaaacaaaatccagacatagtcatctat

caatacatggatgatttgtatgtaggatctgacttagagatagggcagcatagaacaaaa

atagaggaactgagacaacatttgttgaggtggggatttaccacaccagacaagaaacat

cagaaagaacctccatttctttggatggggtatgaactccatcctgacaaatggacagta

cagcctatacagytrccagtacaagatagctggactgtcaatgatatacaaaagttagtg

ggaaaattaaactgggcaagtcagatttatcctggaattaaagtaaggcaactttgtaaa

ctccttaggggggccaaarcactaacagacatagtaccactaactgaagaagcag-----

------------------------------------------------

>CRF07BC.G-HZ131096

cctcaaatcactctttggcaacgaccccttgttaccataaagataggggggcaattaaag

gaagctctattagatacaggagcagatgatacagtattagaagacatgaatttgccaggg

aaatggaaaccaaaaatgatagggggaattggaggttttatcaaagtaagagagtatgaa

cagatacccatagaaatctgtggacataaggctataggtacagtattagtaggacctaca

cctgtcaacataattggaaggaatctgttgactcagcttggttgtaccttaaattttcca

atcagtcctattgaaactgtaccagtaaaattaaagccaggaatggatggcccaaaggtt

aaacaatggccattgacaaaagagaaaatagaagcattaacagcaatttgtgatgaaatg

gaaaaggaaggaaaaattacaaaaattgggcctgaaaatccatacaacactccaatattt

gccataaaaaagaaagayagtactaagtggagaaaattagtagatttcagggaactcaac

aaaagaactcaagatttttgggaagttcaattaggaataccacatccagcaggattaaaa

aagaaaaaatcagtgacagtgctggatgtgggagatgcatatttttcagttcctttakat

gaagmcttcaggaaatatactgcattcaccatacctagtataaacaatgaaacaccaggg

attagatatcagtacaatgtacttccacagggatggaaaggatcaccagcaatatttcaa

agtagcatgacaaaaatcttagagccttttagaaaacaaaatccagacatagtcatctat

caatacatggatgatttgtatgtaggatctgacttagagatagggcagcatagaacaaaa

atagaggaactgagacaacatttgtycaggtggggatttaccacaccagacaagaaacat

cagaaagaacctccatttctttggatggggtatgaactccatcctgacaaatggacagta

cagcctatacagctaccagtacaagatagctggactgtcaatgatatacaaaagttagtg

ggaaaattaaactgggcaagtcagatttatcctggaattaaagtaaggcaactttgtaaa

ctccttaggggggccaaagcactaacagacatagtaccactaactgaagaagcagaatta

ga----------------------------------------------

>CRF07BC.G-HZ131101

cctcaaatcactctttggcaacgacccctcgtcccaataaggataggtgggcaattaaag

gaagctctattagatacaggagcagatgatacagtrttagaagacatgaatttgccaggg

aaatggaaaccaaaaatgatagggggaattggaggttttatcaaagtaagagagtatgaa

cagatacccatagaaatttgcggacacaaagctataggtacagtattagtaggagmtaca

cctgtcaacataattggaagaaatctgttgactcagcttggttgcactttaaattttcca

atcagtcccattgaaactgtaccagtaaaattaaagccaggaatggatggcccaaaggtt

aaacaatggccattgacagaagaraaaataaaagcattaacagcaatttgtgatgaaatg

gagaaggaaggaaaaattacaaaaattgggcctgaaaatccatataacactccaatattt

gccataaaaaagaaggacagtactaagtggagaaaattagtagacttcagggaactcaat

aaaagaactcaagatttttgggaagttcaattaggaataccacacccagcagggttaaaa

aagaaaaaatcagtgacagtactrgatgtgggggatgcatatttttcagttcctttatat

gaagacttcaggaartatactgcattcaccatacctagtataaacaatgaaacaccaggg

attaggtatcagtacaatgtacttccacagggatggaaaggatcaccagcaatattccaa

agtagcatgacaaaaatcttagagccttttagaaaacaaaatccagacatagtyatctat

cagtacatggatgatttgtatgtaggatctgacttagagataggacagcatagaacaaaa

atagaggaaytgagacaacatttgttgaggtggggatttaccacaccagacaagaaacat

cagaaagaacctccatttctttggatggggtatgaactccatcctgacaaatggacagta

cagcctatacagctgccagaaaaagatagctggactgtcaatgatatacaaaagttagtg

ggaaaattaaactgggcaagtcagatttatcctggaattaaagtaaggcaactttgyaaa

ctccttaggggrgccaaagcactaacagaaatagtaccactaactgaagaagcaga----

------------------------------------------------

>CRF07BC.G-HZ131105

cctcaaatcactctttggcaacgaccccttgttccaataaagatagggggacagttaaag

gaagctctattagatacaggagcagatgatacagtattagaagacatgaatttgccaggg

aaatggaaaccaaaaatgatagggggaattggaggttttattaaagtaagacagtatgaa

cagatacccatagaaatctgtggacataaagctataggtacagtattagtaggacctaca

cctgtcaacataattggaaggaatttgttgactcagcttggttgtactttaaattttcca

atcagtcctattgaaactgtaccagtaaaactaaagccaggaatggagggcccaaaggtt

aaacaatggccattgacaaaagagaaaatagaagcattaacagcaatttgtgatgaaatg

gaaaaggaaggaaaaattacaaaaattgggcctgagaatccatacaacactccaatattt

gctataaaaaagaaagacagtactaagtggagaaaattagtagatttcagggaactcaat

aaaagaactcaagatttttgggaagttcaattaggaataccacacccagcaggattaaaa

aagaaaaaatcagtgacagtgctggatgtgggggatgcatatttttcagtccctttatat

gaagacttcaggaaatatactgcattcaccatacctagtataaacaatgaaacaccaggg

attaggtatcagtataatgtacttccacagggatggaaaggatcaccagcaatatttcaa

agtagcatgacaaaaatcttagagccttttagaaaacaaaatccagacatagtcatctat

caatacatggatgatttgtatgtaggatctgacttagagatagggcagcatagaacaaaa

atagaggaactgagacaacatttgttgaagtggggatttaccacaccagacaagaaacat

cagaaagaacctccatttctttggatggggtatgaactccatcctgacaagtggacagta

cagcctatacagctgccagtacaagatagctggactgtcaatgatatacaaaagttagtg

ggaaaattaaactgggcaagtcagatttatcctggaattaaagtaaggcaactttgtaaa

ctccttaggggggccaaagcactaacagacatagtaccactaactgaagaagcaga----

------------------------------------------------

>CRF07BC.G-HZ131197

cctcaaatcactctttgggaacgacccattgttacmataaagataggggggcaattaaag

gaagctctattagatacaggagcagatgatacagtattagaagacatgaatttgccaggg

aaatggaaaccaaaaatgatagggggaattggaggttttatcaaagtgagacagtatgaa

caaatacccatagaaatytgcggacacaaagctataggtacagtattaataggacctaca

cctgtcaacataattggaagaaatctgttgactcaactkggttgcactttaaattttcca

atcagtcccattgaaaccataccagtaaaattaaagccaggaatggatggcccaaaggtc

aaacartggccattgacagaagaaaaaataaaagcattaacagaaatttgtaatgaaatg

gagaaggaaggraaaattacaaaaattgggcctgaaaatccatataacactccaatattt

gccataaaaaagaaggacagtactaagtggagaaaattagtagatttcagggaactcaat

aaaagaactcaagatttttgggaagttcaattaggaataccacacccagcagggttaaaa

aagaaaaaatcagtgacagtgctggatgtgggagatgcatatttttcagttcctttatat

gaagacttcaggaaatatactgcatttaccatacctagtataaacaatgaaacaccaggg

attaggtatcaatacaacgtacttccacagggatggaaaggctcaccagcaatattccaa

agtagcatgacaaaaatcttagagccttttagaaaacaaaatccagamwtrgttatctat

caatacatggatgatttgtatgtaggmtctgayttagagatagggaagcacagaacaaaa

atagaggaactaagacaacatttgttgggrtgggggtttactacaccagataagaaacat

cagaaagaacctccatttctttggatggggtatgaactccatcctgacaaatggacagta

cagcctatacagctgccagacaaagatagctggactgtcaatgatatacagaagttagtg

ggaaaattaaactgggcaagtcagatttatcctggaattaaagtaaggcaactttgtaaa

ctccttagggggaccaaagccctaacagacatagtaccactaactgaagaagcagaatta

gaattggcag--------------------------------------

>CRF07BC.G-HZ131301

cctcaaatcactctttggcaacgaccccttgttaccataaagataggggggcaattaaag

gaagctctattagatacaggagcagatgatacagtattagaagacatgaatttgccaggg

aaatggaaaccaaaaatgatagggggaattggaggttttatcaaagtaagacagtatgaa

cagatacccatagaaatctgtggacatcaagytataggtacagtattartaggacctaca

cctgtcaacataattggaagaaatctgttgactcagcttggttgtactttaaattttcca

atcagtcctattgaaactgtaccagtaaaactaaagccaggaatggatggcccaaaggtt

aaacaatggccattgacaaaagagaaaatagaagcattaacagcaatttgtgatgaaatg

gaaaaggaaggaaaaattacaaaaattgggcctgaaaatccatacaacactccaatattt

gccataaaaaagaaagacagtactaagtggagaaaattagtagatttcagagaactcaat

aagagaactcaagatttttgggaagttcaattaggaataccacacccagcaggattaaaa

aggaaaaaatcagtgacagtgctggatgtgggggatgcatatttttcagttcctttatat

gaagacttcaggaaatatactgcattcaccatacctagtataaacaatgaaacaccaggg

attaggtatcagtacaatgtacttccacagggatggaaaggatcaccagcaatatttcaa

agtagcatgacaaaaatcttagagccttttagaaaacaaaatccagacatagtcatctat

caatacatggatgatttgtatgtaggatctgacttagagatagggcagcatagaacaaaa

atagargaactgagacaacatttgttgaggtgggggtttaccacaccagacaagaaacat

cagaaagaacctccatttctttggatggggtatgaactccatcctgacaaatggacagta

cagcctatacagytgccagtacaagatagctggactgtcaatgatatacaaaagttagtt

ggaaaattaaactgggcaagtcagatttatcctggaattaaagtaaggcaactttgtaaa

ctccttagggggaccaaagcactaacagacatagtaccactaactgaagaagcagaa---

------------------------------------------------

>CRF07BC.G-HZ131411

cctcaaatcactctttggcaacgacccattgtcacaataaagatagggggacaagtaaag

gaagctctattagatacaggagcagatgatacagtattagaagacatgaatttgccagga

aaatggaaaccaaaaatgatagggggaattggaggktttatcaaagtaagacagtatgaa

cagatacccatagaaatctgtggacataaagctataggtacagtattaataggacctaca

cctgtcaacataattggaagaaatctgttgactcagcttggttgcactttaaatttccca

atcagtcctattgaaactgtaccagtaaaattaaagccaggaatggatggcccaaaggtt

aaacaatggccattgacaaaagagaaaatagaagcattaacagcaatttgtgaggaaatg

gaaaaagaaggaaarattacaaaaattgggcccgaaaatccatacaacactccaatattt

gccataaaaaagaaagacagtactaagtggagaaaattagtagatttcagggaactcaat

aaaagaactcaagacttttgggaagttcaattaggaataccacacccagcaggattaaaa

aggaaaaaatcagtgacagtgytggatgtgggggatgcatatttttcagttcctttatat

gaagacttcaggaaatatactgcattcaccatacctagyataaacaatgaaacaccaggg

attaggtatcagtacaatgtacttccacagggatggaaaggatcaccagcaatatttcaa

agtagcatgacaaaaatcttagagccttttagaaaacaaaatccagacatggtcatctat

caatacatggatgatttgtatgtaggatctgacttagagataggtcagcatagaacaaaa

atagaggaactgagacaacatttgttgaggtggggatttaccacaccagacaagaaacat

cagaaagaacctccatttctttggatggggtatgaactccatcctgacaaatggacagta

cagcctatacagctgccagtacaagatagctggactgtcaatgatatacaaaagttagtg

ggaaaattaaactgggcaagtcagatttatcctggaattaaagtaaggcaactttgtaaa

ctccttaggggggccaaagcactaacagacatagtaccactaactgaa------------

------------------------------------------------

>CRF07BC.G-HZ20131153

cctcaaatcactctttggcaacgaccccttgttaccataaagataggggggcaattaaag

gaagctctattagatacaggagcagatgatacagtgttagargatatgaatttgcaaggg

aaatggaagccaaaaatgatagggggaattggaggttttatcaaagtaagacagtatgaa

gagatactcatagaaatctgtggacataaagttataggtacagtattggtaggacctaca

cctgtcaacataattggaaggaatctgttgactcagcttggttgtactttaaattttcca

atcagtcctattaaaactgtaccagtaaaactgaagccaggaatggatggcccaaaggtt

aaacaatggccattgacaaaagagaaaatagaagcattaacagcaatttgtgatgaaatg

gaaaaggaaggaaaaattacaaaaattgggcctgaaaatccatacaacactccaatattt

gccataaaaaagaaagacagtactaagtggagaaaattagtagatttcagggaactcaat

aaaagaactcaagatttttgggaagttcaattaggaataccacacccagcaggattaaaa

aagaaaaaatcagtgacagtgctggatgtgggggatgcatatttttcagttcctttatat

gaagacttcaggaaatatactgcattcaccatacctagtataaacaatgaaacaccaggg

attaggtatcagtacaatgtacttccacagggatggaaaggatcaccagcaatatttcaa

agtagcatgacaaaaatcttagagccttttagaaaacaaaatccagacatagtcatctat

caatacatggatgatttgtatgtaggatctgacttagagatagggcaacatagaacaaaa

atagaggaactgagacaacatttgttgaggtggggatttaccacaccagacaagaaacat

cagaaagaacctccatttctttggatggggtatgaactccatcctgacaaatggacagta

cagcctatacagctgccagtgcaagatagctggactgtcaatgatatacaaaagttagtg

ggaaaattaaactgggcaagtcagatttatcctggaattaaagtaaggcaactttgtaaa

ctccttaggggggccaaagcactaacagacatagtaccactaactgaagaagc-------

------------------------------------------------

>CRF07BC.Hu2014328

cctcaaatcactctttggcagcgaccccttgtcacaataaagataggggggcaattaaag

gaagctctcttagatacaggagcagatgatacagtattagaagacatgaatttgccaggg

aaatggaaaccaaaaatgatagggggaattggaggttttatcaaagtaaaacagtatgaa

gagatacccatagaaatctgyggacacaaagctrtagggacagtattagtrggrccaacg

cctgtcaacataattggaagaaatttgttgactcagattggttgcactttaaattttcca

atcagtcccattgaaactgtaccagtaaaattaaagccaggaatggatggcccaaaggtt

aaacaatggccattgacagaagagaaaataaaagcattaacactaatttgtgaggaaatg

gagaaggaaggaaaaattacaaaaattgggcctgaaaatccatataacactccaatattt

gccataaaaaagaaagacagtactaagtggagaaagttagtagatttcagggaactcaat

aaaagaactcaagatttttgggaggttcaattaggaataccacatccagcagggttaaaa

aagaacaaatcagtaacagtactggatgtgggggatgcatatttctcagttcctttatat

gaagacttcaggaaatatactgcattcaccatacctagtataaacaatgaaacaccaggg

attaggtatcagtacaatgtacttccacagggatggaaaggatcaccagcaatattccaa

agcagcatgacaaaaatcttagagccttttagaaaacaaaatccagatatagagatctat

caatacatggatgatttgtatgtaggatctgacttagagatagggcagcatagaacaaaa

atagaggaactgagagaacatttgytgaggtggggatttaccacaccagacaagaaacat

cagaaagaacctccatttctttggatggggtatgaactccatcctgacaaatggacagta

cagcctatacagttaccagaaaaagatagctggactgtcaatgatatacaaaagttagtg

ggaaaattaaactgggcgagtcagatttatcctggaattaaagtaagacaactttgtaaa

ctccttagggggaccaaagcactaacagaaatagtaccactaactgaagaagcagaatta

gaattggcaga-------------------------------------

>CRF07BC.HUZ160832

cctcaaatcactctttggcaacgaccccttgttaccataaagataggggggcaattaaag

gaagctctattagacacaggagcagatgatacagtattagaagaaatgaatttgccaggg

aaatggaarccaaaaatgatagggggaattggaggttttatcaaagtaaggcaatatgat

cagatacytatagaaatttgtggaaaaaaggctataggtacagtattagtaggacctaca

cctrtcaacataattggaagaaatmtgttgactcagcttggttgtactttaaattttcca

atcagtcctattgaaactgtaccagtaaaactaaagccaggaatggatggcccaaaggtt

aaacaatggccattgacaaaagaraaaatagaagcattaacagcaatttgtgatgaaatg

gaaaaggaaggaaaaatatcaaaaattgggcctgaaaatccatacaacactccaatattt

gccataaaaaagaaagacagtactaagtggagaaaattagtagatttcagggaactcaat

aaaagaacacaagatttttgggaarttcaattaggaataccacacccagcaggattaaaa

argaayaaatcagtgacagtactagatgtgggagatgcatatttttcagtccctttagat

gaaagctttagaaagtatactgcattcaccatacctagtayaaacaatgagamaccggga

atcagatatcagtacaatgtgctaccacagggatggaaaggatcaccagcaatatttcaa

agtagcatgacmaaaatcttagagccttttagaaaacaaaatccagacatagtcatctrt

caatacgtggatgatttgtatgtaggatctgacttagagatagggcagcatagaacaaaa

atagaggagctgagacaacatttgttgaggtggggatttacyacaccagacaaaaagcat

cagaaggaacckccatttctttggatgggatatgaactccatccggatagatggacagtc

cagcccataraactgccagaaaaagacagctggactgtcaatgatatacaraaattagtg

ggaaaactaaattgggcaagtcaaatttatccagggattaaggtaaagcaattgtgtaaa

ctcctcaggggagctaaagcattaacagacgtagtaccactgactaaagaagcagaat--

------------------------------------------------

>CRF07BC.HUZ161221

cctcaaatcactctttggcaacgaccccttgttaccataaagataggggggcaattaaag

gaagctctattagatacaggagcagatgatacagtattagaagacatgaatttgccaggg

aaatggaaaccaaaaatgatagggggaattggaggttttatcaaagtaagacagtatgaa

caggtacccatagaaatctgtggacataaagccataggtacagtattagtaggacctaca

cctgtcaacataattggaagaaatctgttgactcaacttggttgtactttaaattttcca

atcagtcctattgaaactgtaccagtaaaactaaagccaggaatggatggcccaaaggtt

aaacaakggccattgacaaaagagaaaatagaagcattaacagcaatttgtgctgaaatg

gaaaaggaaggaaaaattacaaaaattgggcctgaaaatccatacaacactccaatattt

gccataaaaaagaaagacagtactaagtggagaaaattagtagatttcagggaactcaat

aaaagaactcaagatttttgggaagttcaattaggaataccacacccagcagggttaaaa

aagaaaaaatcagtgacagtgctggatgtgggggatgcatatttttcagttcctttatat

gaagacttcaggaaatatactgcattcaccatacctagtataaacaatgaaacaccaggg

attaggtatcagtacaatgtacttccacagggatggaaaggatcaccagcaatatttcaa

agtagcatgacaaaaatcttagagccttttagaaaacaaaatccagacatagtcatctat

caatacatggatgatttgtatgtaggatctgacttagagatagggcagcatagaacaaaa

atagaggaactgagacaacacttgttgaggtggggatttaccacaccagacaaaaaacat

cagaaagaacctccatttctttggatggggtatgaactccatcctgacaaatggacagta

cagcctatacagctaccagtacaagatagctggactgtcaatgatatacaaaagttagtg

ggaaaattaaactgggcaagtcagatttatcctggaattaaagtaaggcagctttgtaaa

ctccttaggggaaccaaggcgctaacagacatagtaccactaactgaagaagcagaa---

------------------------------------------------

>CRF07BC.HUZ161440

cctcaaatcactctttggcaacgaccccttgttaccataaaaataggggggcaattaaag

gaagctctattagatacaggagcagatgatacagtattagaagacataaatttgccaggg

aaatggaaaccaaaaatgatagggggaattggaggttttatcaaagtaagacagtatgaa

caggtacccatagaaatctgtgggcataaagttataggtacagtattagtaggacctaca

cctgtcaacataattggaaggaatctgttgactcagcttggttgtactttaaattttcca

atcagtcctattgaaactgtaccagtaaaactaaagccaggaatggatggcccaaaggtt

aaacaatggccattgacaaaagagaaaatagaagcattaacagcaatttgtgatgaaatg

gaaaaagaaggaaaaattacaaaaattgggcctgaaaatccatacaatactccaatattt

gccataaaaaagaaagacagtactaaatggagaaaattagtagacttcagggaactcaat

aaaagaactcaagatttttgggaagttcaattaggaataccacacccagcaggattaaaa

aagagaaaatcagtgacagtgctggatgtgggggatgcatatttttcagttcctttatat

gaagacttcaggaaatatactgcattcaccatacctagtayaaacaatgaaacaccaggg

attaggtatcagtacaatgtacttccacaaggatggaaaggatcaccagcaatatttcaa

agtagcatgacaaaaatcttagagcctttcagaaaacagaacccagacatagacatctgt

caatacgtggatgatttgtatgtaggatctgacttagagatagggcagcatagaacaaaa

atagaggaactgagacaacatctgttgaggtggggatttaccacaccagacaagaaacat

cagaaagaacctccatttctttggatgggttatgaactccatcctgacaaatggacagta

cagccaatacagctgccagtacaagatagctggactgtcaatgatatacaaaagttagtg

ggaaaattaaactgggcaagtcagatttatcctggaattaaagtgaggcaactttgtaaa

ctccttaggggggccaaagcactaacagacatagtaccactaactgaagaagcagaat--

------------------------------------------------

>CRF07BC.HUZ16213

cctcaaatcactctttggcaacgacccctcgtcacagtaaagatagggggacaattaaag

gaagctctattagatacaggagcagatgatacagtattagaagacatgaatttgccaggg

aaatggaaaccaaaaatgatagggggaattggaggttttatcaaagtaagacagtatgaa

cagatacccatagaaatctgtggacataaagctataggtacagtattaataggacctaca

cctgtcaacataattggaagaaatctgttgactcagattggttgcactttaaatttccca

atcagtcctattgaaactgtaccagtaaaattaaagccaggaatggatggcccaaaggty

aaacaatggccattgacaaaagagaaaatagaagcattaacagcaatttgtgaggaaatg

gaaaaagaaggaaaaattacaaaaattgggccagaaaatccatacaacactccaatattt

gccataaaaaagaaagacagtactaagtggagaaaattagtagatttcagggaactcaat

aaaagaacccaagacttttgggaagttcaattaggaataccacacccagcaggattaaaa

aagaaaaaatcagtgacagtgctggatgtgggggatgcatatttttcagttcctttacat

gaagacttcaggaaatatactgcattcaccatacctagtacaaacaatgaaacaccaggg

attaggtatcagtacaatgtacttccacagggatggaaaggatcaccagcaatatttcaa

tgtagtatgacaaaaatcttagagccttttagaaagcagaatccaaacatagtcatctat

caatacatggatgatttgtatgtaggatctgacttagagatagggcagcatagaacaaaa

gtagaggaactgagacaacatttgttgaagtggggatttacaacaccagacaagaaacat

cagaaagaacctccatttctttggatggggtatgaactccatcctgacaaatggacagta

cagcctatacagctgccagtacaagatagctggactgtcaatgatatacaaaagttagtg

ggaaaattaaactgggcaagtcagatttatcctggaattaaagtaaggcaactttgtaaa

ctccttaggggggccaaagcactaacagacatagtaccactaactgaagaagcagaat--

------------------------------------------------

>CRF07BC.HUZ16396

cctcaaatcactctttggcaacgacccattgtcacaataaagatagggggacaagtaaag

gaagctctattagatacaggagcagatgatacagtattagaagacatgaatttgccaggg

aaatggaaaccaaaaatgatagggggaattggaggttttatcaaagtaagacagtatgac

cagatacccatagaaatctgtggacataaagctataggtacagtattagtaggacctact

cctatcaatataattggaaggaatctgttgactcagcttggttgtactttaaatttccca

atcagtcctattgacactgtaccagtaaaactgaagccaggaatggatggcccaaaggtt

aaacagtggccattgacaaaagagaaaatagaagcattaacagcaatttgtgatgaaatg

gaaaaggaaggaaaaattacaaaaattgggcctgaaaatccatacaacactccaatattt

gccataaaaaagaaagacagtactaagtggagaaaattagtagatttcagggaactcaat

aaaagaactcaagatttttgggaagttcaattaggaataccacacccagcaggattaaaa

aagaaaaaatcagtgacagtgctagatgtaggggatgcatatttttcagttcctttagat

gaaaacttcaggaaatatactgcattcaccatacctagtataaacaatgaaacaccaggg

attaggtatcagtacaatgtacttccacagggatggaaaggatcaccagcaatatttcaa

agtagcatgacaaaaatcttagagccttttagaaaacaaaatccagacatagtcatctat

caatacatggatgatttgtatgtaggatctgacctagaaatagggcagcatagaacaaaa

atagaggaactgagacaacatttgttgaggtggggatttaccacaccagacaagaaacat

cagaaagaacctccatttctttggatgggatatgaactccatcctgacaaatggacagta

cagcctatacagctgccagtacaagatagctggactgtcaatgatatacaaaagttagtg

ggaaaattaaactgggcaagtcagatttatcctggaattaaagttaggcaactttgtaaa

ctccttaggggagccaaagcactaacagacatagtaccactaactgaagaagcagaatta

gaattggcag--------------------------------------

>CRF07BC.HuZ16490

cctcaaatcactctttggcaacgaccccttgttaccataaagataggggggcaattaaag

gaagctctattagatacaggagcagatgatacagtattagaagatataaatttgccaggg

aaatggaaaccaaaaatgatagggggaattggaggttttatcaaagtaagagagtatgaa

cagatacccatagaaatctgtggacataaagctataggtacagtattaataggacctaca

cctgtcaacataattggaaggaatctgttgactcagcttggttgtactttaaattttcca

atcagtcctattgaaactgtaccagtaaaattaaagccaggaatggatggcccaaaggtt

aaacaatggccattgacaaaagagaaaatagaagcattaacagcaatttgtgaggaaatg

gaaaaggaaggaaaaattacaaaaattgggcctgaaaatccatacaacactccaatattt

gccataaaaaagaaagacagtactaagtggagaaaattagtagatttcagggaactcaat

aaaagaactcaagatttttgggaagttcaattaggaataccacatccagcaggattaaaa

aagaaaaaatcagtgacagtgctggatgtgggagatgcatatttttcagttcctttatat

gaagacttcaggaaatatactgcattcaccatccctagtacaaacagtgaaacaccaggg

attagatatcagtacaatgtacttccacagggatggaaaggatcaccagcaatatttcaa

agtagcatgacaaaaatcttagagccttttagaaaacaaaatccagacatagtcatctat

caatacatggatgatttgtatgtaggatctgacttagagatagggcagcatagaacaaaa

atagaggaactgagagaacatttgttgaggtggggatttaccacaccagacaagaaacat

cagaaagaacctccatttctttggatggggyatgaactccatcctgacaaatggacagta

cagcctatacagctgccagtacaagatagctggactgtcaatgatatacaaaagttagtg

ggaaaattaaactgggcaagtcagatttatcctggaattaaagtaaggcaactttgtaaa

ctccttaggggggccaaagcactaacagacatagtaccactaactgaagaagcagaatta

gaattggcaga-------------------------------------

>CRF07BC.HUZ16609

cctcaaatcactctttggcaacgaccccttgttaccataaagataggggggcaattaaag

gaagctctattagatacaggagcagatgatacagtattagaagacatgaatttgccaggg

aaatggaaaccaaaaatgatagggggaattggaggttttatcaaagtaagacagtatgaa

cagatacccgtagaaatctgtggacataaagctataggtacagtattagtaggacctaca

cctgtcaacataattggaagaaatctgttgactcagcttggttgtactttaaattttcca

atcagtcctattgaaactgtaccagtaaaactaaagccaggaatggatggcccaaaggtt

aaacaatggccattgacaaaagaaaaaatagaagcattaacagaaatttgtaatgaaatg

gaaaaggaaggaaaaattacaaaaattgggcctgaaaatccatacaacactccaatattt

gccataaaaaagaaagacagtactaagtggagaaaattagtagatttcagggaactcaat

aaaagaactcaagatttttgggaagttcaattaggaataccacacccagcaggattaaaa

aagaaaaaatcagtgacagtgctggatgtgggggatgcatatttttcagttcctttatat

gaagacttcaggaaatayactgcattcaccatacctagtataaacaatgaaacaccaggg

attaggtatcartacaatgtacttccacarggatggaaaggatcaccmgcaatatttcaa

agtagcatgacaaraatcttagagccttttagaaarcaaaatccagacatagtcatctat

caatacatggatgatttgtatgtaggatctgacttagagatagggcagcatagaacaaaa

atagaggaactgagacaacatttgttgagttggggatttaccacaccagacaaraaacat

cagaaagaacctccatttctttggatggggtatgaactccatcctgacaaatggacagta

cagcctatacagctgccagtacaagatagctggactgtcaatgatatacaaaagttagtg

ggaaaattaaactgggcaagtcagatttatcctggaattaaagtaagrcaactttgtaaa

ctccttaggggggccaaagcactaacagacatagtaccactaactgaagaagca------

------------------------------------------------

>CRF07BC.HuZ16703

cctcaaatcactctttggcaacgaccccttgttaccataaaaataggggggcaattaaag

gaagctctattagatacaggagcagatgatacagtattagaagaaatgaatttgccaggg

aaatggaaaccaaaaatgatagggggaattggaggttttatcaaagtaagacagtatgaa

caggtacccatagaaatctgtggacataaagctataggtacagtattaataggacctaca

cctgtcaacataattggaagaaatctgttgactcagcttggttgtactttaaattttcca

atcagtcctattgaaactgtaccagtaaaactaaarccaggaatggatggcccaaaggtt

aaacaatggccattaacaaaagagaaaatagaagcattaacagcaatttgtgatgaaatg

gaaaaggaaggaaagattacaaaaattgggcctgaaaatccatacaacactccaatattt

gccataaaaaagaaagacagtacgaagtggagaaaattagtagatttcagggaactcaat

aaaagaactcaagatttttgggaagttcaattaggaataccacacccagcaggattaaaa

aagaaaaaatcagtgacagtgctggatgtgggggatgcatatttttcagttcctttagat

gaggacttcaggaaatatactgcattcaccatacctagtataaacaatgaaacaccaggg

atcaggtatcagyacaatgtacttccacagggatggaaaggatcaccagcaatatttcaa

agtagcatgacaagaatcttagagccttttagaaaacaaaatccagacatagtcatctat

caatacatggatgatttgtatgtaggctctgacttagagatagggcagcatagaacaaaa

atagaggaactgagacaacatttattgaggtggggatttaccacaccagacaagaaacat

cagaaagaacctccattyctttggakggggtatgagctccatcctgacaaatggacagta

cagcctatacagctgccagtacaagatagctggactgtcaatgatatacaaaagttagtg

ggaaaattaaactgggcaagtcagatttatcctggaattaaagtaaggcaactttgtaaa

ctccttaggggggccaaagcactaacagacatagtaccattaactgaagaagcagaatta

gaattggcaga-------------------------------------

>CRF07BC.HuZ2013168

cctcaaatcactctttggcaacgaccccttgtcacaataaagataggggggcaattaaag

gaagctctattagatacaggagcagatgatacagtattagaagacatgaatttgccagga

aaatggaaaccaaaaatgatagggggaattggaggttttatcaaagtaagacagtatgaa

cagatacccatagaaatctgtggacataaagctataggtacagtgttagtaggacctaca

cctgtcaacataattggaagaaatctgttgactcagattggttgtactttaaattttcca

atcagtcctattgaaactgtaccagtaaaactaaagccaggaatggatggcccaaaggtt

aaacaatggccattgacaaaagaaaaaatagaagcattaacagcaatttgtgatgaaatg

gaaaaggagggaaaaattacaaaaattgggcctgaaaatccatacaacactccaatattt

gccataaaaaagaaagacagtactaagtggagaaaattagtagacttcagggaactcaat

aaaagaactcaagatttttgggaagttcaattaggaataccacacccagcaggattaaaa

aagaaaaaatcagtgacagtgctagatgtgggggatgcatatttttcagttcctttatat

gaagacttcaggaagtatactgcattcaccatacctagtataaacaatgaaacaccaggg

attaggtatcagtacaatgtacttccacagggatggaaaggatcaccagcaatatttcaa

agtagcatgacaaaaatcttagagccttttagaaaacaaaatccagacatagtcatctat

caatacatggatgatttgtatgtaggatctgacttagagatagggcagcatagaacaaaa

atagaggaactgagacaacatttgttgaggtggggatttaccacaccagacaagaaacat

cagaaagaacctccatttctttggatggggtatgaactccatcctgacaaatggacagta

cagcctatacagctgccagtacaagatagctggactgtcaatgatatacaaaagttagtg

ggaaaattaaactgggcaagtcaaatttatcctggaattaaagtaaggcaactttgtaaa

ctccttaggggggccaaagcactaacagacatagtaccactaactgaagaagcagaatta

gaattggcag--------------------------------------

>CRF07BC.HuZ2013173

cctcaaatcactctttggcaacgaccccttgtcacaataaagataggggggcaatcaaag

gaagctctattagatacaggagcagatgatacagtattagaagacatgaatttgccaggg

aaatggaaaccaagaatgatagggggaattggaggttttatcaaagtaagacagtatgaa

caggtacccatagaaatctgtggacataaagctataggtacagtattagtaggacctaca

cctgtcaacataattggaagaaatctgttgactcagcttggttgtactttaaattttcca

atcagtcctattgaaactgtaccagtaaaactaaagccaggaatggatggcccaaaggtt

aaacaatggccattgacaaaagaaaaaatagaagcattaacagcaatttgtgatgaaatg

gaaaaggaaggaaaaattacaaaaattgggcctgaaaatccatacaacactccaatattt

gccataaaaaagaaagacagtactaagtggagaaaattagtagatttcagggaactcaat

aaaagaactcaagatttttgggaagttcaattaggaataccacacccagcaggattaaaa

aagaaaaaatcagtracagtgctggatgtgggggatgcatatttttcagttcctttagat

gaaaacttcaggaaatatactgcattcaccatacctagtataaacaatgaaacaccaggg

attaggtatcagtacaatgtacttccacagggatggaaaggatcaccagcaatatttcaa

agtagcatgacaagaatcttagagccttttagaaaacaaaatccagacatagtcatctat

caatacatggatgatttgtatgtaggwtctgacttagagatagggcagcatagaacaaaa

atagaggaactgagrcaacatttgttgaggtggggatttaccacaccagacaagaaacat

cagaaagaacctccatttctttggatggggtatgaactccatcctgacaartggacagta

cagcctatacagctgccagtacaagatagctggactgtcaatgatatacaaaagttagtg

ggraaattaaactgggcaagtcagatttatcctggaattaaagtaaggcaactttgtaaa

ctccttagggggaccaaagcactaacagacatagtaccactaactgaagaagcagaat--

------------------------------------------------

>CRF07BC.HuZ2013238

cctcaaatcactctttggcaacgaccccttgtcacaataaagataggggggcaattaaag

gaagctctattagatacaggagcagatgatacagtattagaagacatgaatttgccaggg

aaatggaaaccaraaatgatagggggaattggaggttttatcaaagtaaaacagtatgaa

cagatacccatagaaatctgtggacataaagctataggtacagtattagtaggacctaca

cctgtcaacataattggaagaaatctgttgactcagcttggttgtactttaaattttccg

atcagtcctattgaaactgtaccagtaaaactaaagccaggaatggatggcccaaaggtt

aaacaatggccattaacaaaagagaaaatagaagcattaacagcaatttgtgatgagatg

gaaaaggaaggaaaaattacaaaaattgggcctgaaaatccatacaacactccaatattt

gccataaaaaagaaagacagtactaagtggagaaaattagtagatttcagagaactcaat

aaaagaactcaagatttttgggaagctcaattaggaataccacacccagcaggattaaaa

aagaaaaaatcagtgacagtgctggatgtgggggatgcatatttttcagttcctttatat

gaagacttcaggaaatatactgcattcaccatacctagtataaacaatgaaacaccaggg

attaggtatcagtacaatgtacttccacagggatggaaaggatcaccagcaatatttcaa

agtagcatgacaaaaatcttagagccttttagaaagcaaaatccagacatagtcatctat

caatacatggatgatttgtatgtaggatctgacttagagatagggcagcatagaataaaa

atagaggaattgagacaacatttgttgaggtggggatttaccacaccagacaagaaacat

cagaaagaacctccatttctttggatggggtatgaactccatcctgacaagtggacagta

cagcctatacagctgccagtacaagatagctggactgtcaatgatatacaaaagttagtg

ggaaaattaaactgggcaagtcagatttatcctggaattaaagtaaggcaactttgtaaa

ctccttaggggggccaaagcactaacagacatagtaccactaactgaagaagcagaa---

------------------------------------------------

>CRF07BC.HuZ2013240

cctcaaatcactctttggcaacgaccccttgtcacagtaaagatagggggacaattaaag

gaagctctattagatacaggagcagatgatacagtattagaagacatgaatttgccaggg

aaatggaaaccaaaaatgatagggggaattggaggttttatcaaagtaagacagtatgaa

caggtacccatagaaatctgtggacataaagctataggtacagtattagtaggacctaca

cctgtcaacataattggaagaaatctgttgactcagcttggttgcactttaaatttccca

atcagtcctattgaaactgtaccagtaaaattaaagccaggaatggatggcccaaaggtt

aaacaatggccattgacaaaagagaaaatagaagcattaacagcaatttgtgaggaaatg

gaaaaagaaggaaaaattacaaaaattgggcccgaaaatccatacaacactccaatattt

gccataaaaaagaaagacagtactaagtggagaaaattagtagatttcagggaactcaat

aaaagaactcaagacttttgggaagttcaattaggaataccacacccagcaggattaaaa

aagaaaaaatcagtgacagtgctggatgtgggggatgcatatttttcagttcctttatat

gaagacttcaggaaatatactgcattcaccatacctagtacaaacaatgaaacaccaggg

attaggtatcagtacaatgtacttccacagggatggaaaggatcaccagcaatatttcaa

agtagcatgacaaaaatcttggaaccttttagaaaacaaaatccaaacatagtcatctat

caatacatggatgatttgtatgtaggatctgacttagagatagggcagcatagaacaaaa

atagaggaactgagacaacatttgttgaagtggggatttaccacaccagacaagaaacat

cagaaagaacctccattkctttggatggggtatgaactccatcctgacaaatggacagta

cagcctatacagctgccagtacaagatagctggactgtcaatgatatacaaaagttagtg

ggaaaattaaactgggcaagtcagatttatcctggaattaaagtaaggcaactttgtaaa

ctccttaggggggccaaagcactaacagacatagtaccactaactgaagaagcagagcta

gaactggcaga-------------------------------------

>CRF07BC.HuZ2013243

cctcaaatcactctttggcaacgaccccttgttaccataaagataggggggcaattaaag

gaagctctattagatacaggagcagatgatacagtattagaagacatgaatttgccaggg

aaatggaaaccaaaaatgatagggggaattggaggttttatcaaagtaagagagtatgaa

cagatacccatagaaatctgtggacataaagctataggtacagtattaataggacctaca

cctgtcaacataattggaaggaatctgttgactcagcttggttgtactttaaattttcca

atcagtcctattgaaactgtaccagtaaaattaaagccaggaatggatggcccaaaggtt

aaacaatggccattgacaaaagagaaaatagaagcattaacagcaatttgtgaggaaatg

gaaaaggaaggaaaaattacaaaaattgggcctgaaaatccatacaacactccaatattt

gccataaaaaagaaagacagtactaagtggagaaaattagtagatttcagggaactcaat

aaaagaactcaagatttttgggaagttcaattaggaataccacatccagcaggattaaaa

aagaaaaaatcagtgacagtgctggatgtgggagatgcatatttttcagttcctttatat

gaagacttcaggaaatatactgcattcaccatacctagtacaaacagtgaaacaccaggg

attagatatcagtacaatgtacttccacagggatggaaaggatcaccagcaatatttcaa

agtagcatgacaaaaatcttagagccttttagaaaacaaaatccagacatagtcatctat

caatacatggatgatttgtatgtaggatctgacttagagatagggcagcatagaacaaaa

atagaggaactgagacaacatttgttgaggtggggatttaccacaccagacaagaaacat

cagaaagaacctccatttctttggatggggtatgaactccatcctgacaaatggacagta

cagcctatacagctgccagtacaagatagctggactgtcaatgatatacaaaagttagtg

ggaaaattaaactgggcaagtcagatttatcctggaattaaagtaaggcaactttgtaaa

ctccttaggggggccaaagcactaacagaaatagtaccactaactgaagaagcagaa---

------------------------------------------------

>CRF07BC.HuZ2014078

cctcaaatcactctttggcaacgacccmtcgtcrcaataargataggggggcaattaaag

gaggctctattagatacaggagcagatgatacagtattagaagacatgaatttgccagga

aaatggaaaccaaaaatgatagggggaattggaggttttatcaaagtaagacagtatgaa

cagatacccatagaaatttgtggacacaaagctataggtacagtattagtaggacctaca

cctgtcaacataattggcagaaatctgttgactcagcttggttgcactttaaactttccc

atcagtcccattgaaactgtaccagtaaaattaaagccaggaatggatggcccaaaggty

aaacaatggccattgacagaagaaaaaataaaagcattaacagcaatctgtgatgaaatg

gaaaaggaaggaaaaattacaaaaattgggcctgaraatccatataacactccaatattt

gccataaaaaagaaggacagtactaagtggagaaaattagtagatttcagggaactcaat

aaaagaactcaagatttttgggaagttcaattaggaataccacacccagcagggttaaaa

aagaaaaaatcagtgacagtactggatgtgggggatgcatatttttcagttcctttryat

gaagacttcaggaaatatactgcattcaccatacctagtataaacaatgaaacaccaggg

attaggtatcagtacaatgtacttccccagggatggaaaggrtcaccagcaatatttcaa

agtagcatgacaaagatcttagatccttttagaaaacaaaatccagacatagttatctat

caatacatggatgatttgtatgtaggatctgacttagaratagggcagcatagaacaaaa

atagaggaactgagacaacatttgttggggtggggatttaccacaccagacaaaaaacat

cagaaagaacctccatttctttggatggggtatgaactccatcctgacaaatggacagta

cagcctatacagctgccagataaagatagctggactgtcaatgacatacaaaagttagtg

ggaaaattaaactgggcaagtcagatttatcctggaattaaagtaaggcaactttgtaag

ctccttaggggggccaargcactaacagacatartaccactaactgaagaagca------

------------------------------------------------

>CRF07BC.HuZ2014086

cctcaaatcactctttggcaacgaccccttgttaccataaagataggggggcaattaaag

gaagctctattagatacaggagcagatgatacagtattagaagacatgaatttgccaggg

aaatggaaaccaaaaatgatagggggaattggaggttttatcaaagtaagacaatatgaa

gaggtacctatagaaatctgtggacataaagctataggtacagtattagtaggacctaca

cctgtcaacataattggaaggaatctgttgactcagcttggttgtactttaaattttcca

attagtcctattgatactgtaccagtaaaattaaagccaggaatggatggcccaaaggtt

agacaatggccattgacaaaagagaaaatagaagcattaacagcaatttgtgatgaaatg

gaaaaggaaggaaaaattacaaaaattgggcctgaaaatccatacaacactccaatattt

gccataaaaaagaaagacagtactaagtggagaaaattagtagatttcagggaactcaat

aaaagaactcaagatttttgggaagttcaattaggaataccacatccagcaggattaaaa

aagaaaaaatcagtgacagtgctggatgtgggagatgcatatttttcagttcctttatat

gaagacttcaggaaatatactgcattcaccatacctagtataaataatgaaacaccaggg

attagatatcagtacaatgtacttccacaaggatggaaaggatcaccagcaatatttcaa

agtagcatgacaaaaatcttagagccttttagaaaacaaaatccagacatagtcatctat

caatacatggatgatttgtatgtaggatctgacttagagattggacagcatagaataaaa

atagaggaactgagacaacatttgttgaggtggggatttaccacaccagacaagaaacat

cagaaagaacctccatttctttggatggggtatgaactccatcctgacaaatggacagta

cagcctatacagctgccagtacaagatagctggactgtcaatgatatacaaaagttagtg

ggaaagttaaactgggcaagtcagatttatcctggaattaaagtaaggcaactttgtaaa

ctccttagggggaccaaagcactaacagacatagtaccactaaatgaagaagcagaatt-

------------------------------------------------

>CRF07BC.HuZ20142001

cctcaaatcactctttggcaacgaccccttgttaccataaagataggggggcaattaaag

gaagctctattagatacaggagcagatgatacagtattagaagacatgaatttgccaggg

aaatggaaaccaaaaatgatagggggaattggaggttttatcaaagtaagacagtatgaa

sagatacccatagaaatctgtggacataaagctrtaggtacagtattartaggacctaca

cctgtcaacataattggaagaaatctgttgactcagcttggttgtactttaaattttcca

atcagtcctattgaaactgtaccagtaaaaytaaagccaggaatggatggcccaaaggtt

aaacaatggccattgacaaaagagaaaatagaagcattaacagcaatttgtgatgaaatg

gaaaaggaaggaaaaattacaaaaattgggcctgaaaatccatacaacactccaatattt

gccataaaaaagaaagacagtactaagtggagaaaattagtagatttcagrgaactcaat

aaaagaactcaagatttttgggaagttcaattaggaataccacacccagcaggattaaaa

aagaaaaaatcagtgacagtgctrgatgtgggggatgcatatttttcagttcctttacat

gaagacttcaggaaatatactgcattcaccatacctagtataaacaatgaaacaccaggg

attaggtatcagtacaatgtacttccacagggatggaaaggatcaccagcaatatttcaa

agtagcatgacaaaaatcttagagccttttagaaaacaaaatccagacatagtcatctat

caatacatggatgatttgtatgtaggatctgayttagagatagggcagcatagaacaaaa

atagaggaactgagacaacatttgttgaggtggggatttaccacaccagacaagaaacat

cagaaagaacctccatttctttggatggggtatgaactccatcctgacaaatggacagta

cagcctatacagctrccagwacaagatagctggactgtcaatgatatacaaaagttagtg

ggaaaattaaactgggcaagtcagatttatcctggaattaaagtaaggcaactttgtaaa

ctccttaggggrgccaaakcactaacagacatagtaccactaactgaagaagcaga----

------------------------------------------------

>CRF07BC.HuZ2014250

cctcaaatcactctttggcaacgaccccttgtcacagtaaagatagggggacaattaaag

gaagctctattagatacaggagcagatgatacagtattagaagacatgaatttgccaggg

aaatggaaaccaaaaatgatagggggaattggaggttttatcaaagtaagacagtatgaa

cagatacccatagaaatctgtggacataaagctataggtacagtattagtaggacctaca

cctgttaacataattggaagaaatctgttgactcagattggttgcactttaaatttccca

atcagtcctattgaaactgtaccagtaaaattaaagccaggaatggatggcccaaaggtt

aaacaatggccattgacaaaagagaaaatagaagcattaacagcaatttgtgaggaaatg

gaaaaagaaggaaaaattacaaaaattgggccagaaaatccatacaacactccaatattt

gccataaaaaagaaagacagtactaagtggagaaaattagtagatttcagggaactcaat

aaaagaactcaagacttttgggaagttcaattaggaataccacacccagcaggattaaaa

aagaaaaaatcagtgacagtgctggatgtgggggatgcatatttttcagttcctttatat

gaagacttcaggaaatatactgcattcaccatacctagtatraacaatgaaacaccaggg

attaggtatcagtacaatgtacttccacagggatggaaaggatcaccagcaatatttcaa

agtagcatgacaaaaatcttagagccttttagaaaacaaaatccaaacatagtcatctat

caatacatggatgatttgtatgtaggatctgacttagagatagggcagcatagaacaaaa

atagaggaactgagacaacatttgttgaagtggggatttaccacaccagacaagaaacat

cagaaagaacctccatttctttggatggggtatgaactccatcctgacaaatggacagta

cagcctatacagctgccagtacaagatagctggactgtcaatgatatacaaaagttagtg

ggaaaattaaactgggcaagtcagatttatcctggaattaaagtaaggcaactttgtaaa

ctccttaggggggccaaagcactaacagacatagtaccactaactgaagaagcagaa---

------------------------------------------------

>CRF07BC.HuZ2014340

cctcaaatcactctttggcagcgaccccttgtcacaataaagataggggggcaakyaaag

gaagctctcttagatacaggagcagatgatacagtattagaagacatgaatttgccaggg

aaatggaaaccaaaaatgatagggggaattggaggttttatcaaagtaagacagtatgaa

cagatacccatagaaatctgcggacacaaagctatagggacagtattaatagggccaacg

cctgtcaacataattggaagaaatttgttgactcagcttgggtgcactttaaattttcca

atcagtcccattgaaactgtaccagtaaaattaaagccaggaatggatggcccaaaggtt

aaacaatggccattgacagaagagaaaataaaagcattaacagaaatctgtgaggaaatg

gagaaggaaggaaaaattacaaaaattgggcctgaaaatccatataacactccaatattt

gccataaaaaagaaggacagtactaaatggagaaagttagtagatttcagggaactcaat

aaaagaactcaagatttttgggaggttcaattaggaataccacatccagcagggttaaaa

aagaaaaaatcagtaacagtactggatgtgggggatgcatatttttcagttcctttatat

gaagacttcaggaaatatactgcattcaccatacctagtataaacaatgaaacaccaggg

attaggtatcagtacaatgtacttccacagggatggaaaggatcaccagcaatattccaa

agtagcatgacaaaaatcttagagccttttagaaaacaaaatccagatatagttatctat

caatacatggatgatttgtatgtaggatctgacttagagatagggcagcatagaacaaaa

atagaagaactgagagaacatctgttgaggtggggatttaccacaccagacaagaaacat

cagaaagaacctccatttctttggatggggtatgaactccatcctgacaaatggacagta

cagcctatacacctgccagaaaaagatagctggactgtcaatgatatacaaaagttagtg

ggaaaattaaattgggcaagtcagatttatcctggaattaaagtaagacaactttgtaaa

ctccttagggggaccaaagcactaacagacatagtaccactaactgaagaagcagaatta

gaattggcag--------------------------------------

>CRF07BC.HZ140034

cctcaaatcactctttggcagcgaccccttgttacaataaagataggggggcaattaaag

gaagccctattagatacaggagcagatgatacagtattggaagaaatggatttgccaggg

aaatggaaaccaaaaatgatagggggaattggaggttttatcaaagtaagacagtatgaa

cagatacccatagaaatctgtggacataaagctataggtacagtattagtaggacctaca

cctgtcaacataattggaaggaatctgttgactcagcttggttgtactttaaattttcca

atcagtcctattgaaactgtaccagtaaaattaaagccaggaatggatggcccaaaggtt

aaacaatggccattgacaaaagaraaaatagaagcattaacagcaatttgtratgaaatg

gaaaaggaaggaaaaattacgaaaattgggcctgagaatccatacaacactccaatattt

gccataaaaaagaaagacagtactaagtggagaaaattagtagatttcagggaactcaat

aaaagaactcaagatttttgggaagttcaattaggaataccacacccagcaggattaaaa

aagaaaaaatcagtgacagtgctggatgtgggggatgcatatttttcagttcctttacat

gaagactttaggaaatatactgcattcaccatacctagtataaacaatgaracaccaggg

attaggtatcagtacaatgtactgccacagggatggaaaggatcaccagcaatatttcaa

agtagcatgacaaaaatcttagagccttttagaaaacgaaatccagacatagtcatctat

caatacatggatgatttgtatgtaggatctgacttagagatagggcagcatagaacaaaa

atagaggaactgagacaacatttgttgaggtggggatttactacaccagacaagaaacat

cagaaagaacctccctttctttggatggggtatgaactccatcctgacaaatggacagta

cagcctatacagctgccagtacaagatagctggactgtcaatgatatacaaaagttagta

ggaaaattaaaytgggcaagtcagatttatcctggaattaaagtaaggcaactttgtaaa

ctccttaggggggccaaagcactaacagacatagtaccactaactgaagaagcaga----

------------------------------------------------

>CRF07BC.HZ140209

cctcaaatcactctttggcaacgacccctagttccaataaagataggggggcaattaaag

gaagctctattagatacaggagcagatgatacagtattagaagacatgaatttgccaggg

aaatggaaaccaaaaatgatagggggaattggaggttttatcaaagtaagacagtatgaa

cagatacccatagaaatctgtggacataaagctataggtacagtattaataggacctaca

cctgtcaacataattggaagaaatctgttgacgcagcttggttgtactttaaattttcca

atcagtcctattgaaactgtaccagtaaaactaaaaccaggaatggatggcccaaaggtt

aaacaatggccattgacaaaagaaaaaatagaagcattaacagcaatttgtgatgaaatg

gaaaaggaaggaaaaattacaaaaattgggcctgaaaatccatacaacactccaatattt

gccataaaaaagaaagacagtactaagtggagaaaattagtagatttcagggaactcaat

aaaagaactcaagatttttgggaggttcaattaggaataccacacccagcaggattaaaa

aagagaaaatcagtgacagtgctggatgtgggggatgcatatttttcagttcctttatat

gaagacttcaggaaatatactgcattcaccatacctagtacaaacaatgaaacaccaggg

attaggtatcagtacaatgtacttccacagggatggaaaggatcaccagcaatatttcaa

agtagcatgacaaaaatcttagagccttttagaaaacaaaatccagacatagtcatctat

caatacatggatgatttgtatgtaggatctgacttagagatagggcagcatagaacaaaa

atagaggaactgagacaacatttgttgaggtgggaatttaccacaccagacaagaaacat

cagaaagaacctccatttctttggatggggtatgaactacatcctaacaaatggacagta

cagcctatacagctgccagtacaagatagctggactgtcaatgatatacaaaagttagtg

ggaaaattaaactgggcaagtcagatttatcctggaattaaaataaggcaactttgtaag

ctccttaggggggccaaagcgctaacagacatagtaccactaactgaagaagcag-----

------------------------------------------------

>CRF07BC.HZ140707

cctcaaatcactctttggcaacgacccattgtcacaataaagataggggggcaattaaag

gaagctctattagatacaggagcagatgatacagtattagaagacatgaatttgccaggg

aaatggaaaccaaaaatgatagggggaattggaggttttatcaaagtaagacagtatgaa

cagatacccatagaaatctgtggacatcaagctataggtacagtattagtaggacctaca

cctgtcaacataattggaagaaatctgttgactcagcttggttgtactttaaattttcca

atcagtcctattgaaactgtaccagtaaaactaaagccaggaatggatggcccaaaggtt

aaacaatggccattgacaaaagagaaaatagaagcattaacagcaatttgtgatgaaatg

gaaaaggaaggaaaaattacaaaaattgggcctgaaaatccatacaacactccaatattt

gccataaaaaagaaggacagtactaagtggagaaaattagtagatttcagggaactcaat

aaaagaactcaagatttttgggaagttcaattaggaataccacacccagcaggattaaaa

aagaaaaaatcagtgacagtgctggatgtgggggatgcatatttttcagttcctttatat

gaagacttcaggaaatatactgcattcaccatacctagtataaacaatgaaacaccaggg

attaggtaccagtacaatgtacttccacagggatggaaaggatcaccagcaatatttcaa

agtagcatgacaaaaatcttagagccttttagaaaacaaaatccagacatagtcatctat

caatacatggatgatttgtatgtaggatctgacttagagatagggcarcatagaacaaaa

atagaggaactgagacaacatttgttggggtggggatttaccacaccagacaagaaacat

cagaaagaacctccatttctttggatggggtatgaactccatcctgacaaatggacagta

cagcctatacagctgccagtacaagatagctggactgtcaatgatatacaaaagttagtg

ggaaaattaaactgggcaagtcagatttatcctggaattaaggtaaggcaactttgtaaa

ctccttagggggaccaaagcactaacagatgtagtaccactaactgaagaagcagaatta

ga----------------------------------------------

>CRF07BC.HZ140734

cctcaaatcactctttggcaacgaccccttgttaccataaagataggggggcaattaaag

gaagctctattagatacaggagcagatgatacagtattagaagacatgaatttgccaggg

aaatggaaaccaaaaatgatagggggaattggaggttttatcaaagtaaaacagtatgaa

gagatacccatagaaatctgtggccataaagttataggtacagtattartaggacctaca

cctgtcaacataattggaagaaatctgttgactcagcttggttgtactttaaattttcca

atcagtcctattgaaactgtaccagtaaaattaaagccaggaatggatggtccaaaagtt

aaacaatggccattgacaaaagagaaaatagaagcattaacagcaatttgtgatgaaatg

gaaaaggaaggaaaaattacaaaaattgggcctgaaaatccgtacaacactccaatattt

gccataaaaaagaaagacagtactaagtggagaaaattagtagatttcagggaactcaat

aaaagaactcaagatttttgggaagttcaattaggaataccgcacccagcaggattaaaa

aagaaaaaatcagtaacagtgctggatgtgggggatgcatatttttcagttcctttatat

gaagacttcaggaaatatactgcattcaccatacctagtataaacaatgaaacaccaggg

attaggtatcagtacaatgtacttccacagggatggaaaggatcaccagcaatatttcaa

agtagcatgacaaaaatcttagagccttttagaaaacaaaatccagacatagtcatctat

caatacatggatgatttgtatgtaggatctgacttagagatagggcagcatagaacaaaa

atagaggaactgagacaacatttgttgaggtggggatttaccacaccagacaagaaacat

caaaaagaacctccatttctttggatggggtatgaactccatcctgacaaatggacagta

cagcctatacagctgccagtacaagatagctggactgtcaatgatatacaaaagttagtg

ggaaaattaaactgggcaagtcagatttatcctggaattaaagtaaggcaactttgtaaa

ctccttaggggggccaaagcactaacagacatagtaccactaactgaagaagcagaat--

------------------------------------------------

>CRF07BC.HZ141112

cctcaaatcactctttggcaacgaccccttgttaccataaagataggggggcagttaaag

gaagctctattagatacaggagcagatgatacagtattagaagacatgaatttgccaggg

aaatggaaaccaaaaatgatagggggaattggaggttttatcaaagtaagacagtatgaa

cagatacccatagaaatctgtggacataaagctataggtacagtattaataggacctaca

cctgtcaacataattggaagaaatctgttgactcagcttggttgtacattaaattttcca

atcagtcctattgaaactgtaccagtaaaactaaagccaggaatggatggcccaaaggtt

aaacaatggccattgacaaaagagaaaatagaagcattaacagcaatttgtgatgaaatg

gaaaaggaaggaaaaattacaaaaattgggcctgaaaatccatacaacactccaatattt

gccataaaaaagaaagacagtactaagtggagaaaattagtagatttcagggaactcaat

aaaagaactcaagatttttgggaagttcaattaggaataccacacccagcaggattaaaa

aagaaaaaatcagtgacagtgctggatgtgggggatgcatatttttcagttcctttayat

gaagayttcaggaaatatactgcattcaccatacctagtataaacaatgaaacaccaggg

attaggtatcagtacaatgtacttccacagggatggaaaggatcaccagcaatatttcaa

agtagcatgacaaaaatcttagagccttttagaaaacaaaatccagacatagtcatctat

caatacatggatgatttgtatgtaggatctgacttagagatagggcagcatagaacaaaa

atagaggaactgagacaacatttgttgaggtggggatttaccacaccagacaagaaacat

cagaaagaacctccatttctttggatggggtatgaactccatcctgacaaatggacagta

cagcctatacagctgccagtacaagatagctggactgtcaatgatatacaaaagttagtg

ggaaaattaaactgggcaagtcagatttatcctggaattaaagtaaggcaactttgtaaa

ctccttaggggggycaaagcactaacagacatagtaccactaactgaagaagcagaatta

gaattggcag--------------------------------------

>CRF07BC.HZ141122

cctcaaatcactctttggcaacgaccccttgttaccataaagataggggggcaakyaaag

gaagctctattagatacaggagcagatgatacagtattagaggacatgaatttgccaggg

aaatggaaaccaaaaatgatagggggaattggaggttttatcaaagtaagacagtatgaa

cagatacccatagaaatttgtggacataaagctataggtacagtattaataggacctaca

cctgtcaacataattggaagaaatctgttgactcagcttggttgtactttaaattttcca

atcagtcctattgaaactataccagtaaaactaaagccaggaatggatggcccaaaggtt

aaacaatggccattgacaaaagagaaaatagaagcattaacagcaatttgtgatgaaatg

gaaaaggaaggaaaaattacaaaaattgggcctgaaaatccatacaacactccaatattt

gccataaaaaagaaagacagcactaagtggagaaaattagtagatttcagggaactcaat

aaaaggactcargatttttgggaagttcaattaggaataccacacccagcaggattaaaa

aagaaaaaatcagtgacagtgctggatgtgggggatgcatatttttcagttcctttatat

gaagatttcaggaaatatactgcattcacyatacctagtataaacaatgaaacaccaggg

attaggtatcagtacaatgtacttccacagggatggaaaggrtcaccagcaatatttcaa

agtagcatgacaaaaatcttagagccttttagaaaacaaaatccagacatagtcatctat

caatacatggatgatttgtatgtaggatctgacttagagatagggcaacacagaacaaaa

atagaggaactgagacaacatttgttgaagtggggatttaccacaccagacaagaaacat

cagaaagaacctccatttctttggatggggtatgaactccatcctgacaaatggacagta

cagcctatacagctgccagtacaagatagctggactgtcaatgatatacaaaagttagtg

ggaaaattaaactgggcaagtcagatttatcctggaattaaagtaaggcaactttgtaaa

ctccttaggggggccaaagcactaacagacatagtaccactaactgaagaagcagaat--

------------------------------------------------

>CRF07BC.HZ141125

cctcaaatcactctttggcaacgaccccttgttaccataaagataggggggcaattaaag

gaagccctattagatacaggagcagatgatacagtattagaagacatgaatttgccaggg

aaatggaaaccaaaaatgatagggggaattggaggttttatcaaagtaagacagtatgaa

gagatacccatagaaatctgtggacataaagttataggtacagtattagtaggacctacg

cctgtcaacataattggaaggaatctgttgactcagcttggttgtactttaaattttcca

atcagtcctattgatactgtaccagtaaaactaaagccaggaatggatggcccaaaggtt

aaacaatggccattgacaaaagagaaaatagaagcattaacagcaatttgtgatgaaatg

gaaaaggaaggaaaaattacaaaaattgggcctgaaaatccatacaacactccaatattt

gccataaaaaagaaagacagtactaagtggagaaaattagtagatttcagggaactcaat

aaaagaactcaagacttttgggaagttcaattaggaataccacacccagcaggattaaaa

aagaaaaaatcagtgacagtgctggatgtgggagatgcatatttctcagttcctttagat

gaagatttcaggaaatatactgcattcaccatacctagtataaacaatgaaacaccagga

attaggtatcagtacaatgtacttccacagggatggaaaggatcaccagcaatatttcaa

agtagcatgacaaaaatcttagagccttttagaaaacaaaatccagacatagtcatctat

caatacatggatgatttgtatgtaggatctgatttagagatagggcagcatagaacaaaa

atagaggaactgagacrayatttgttaaggtggggatttaccacaccagacaagaaacat

cagaaagaacctccatttctttggatggggtatgaactccatcctgacaaatggacagta

cagcctatacagctgccagtacaagatagctggacggtcaatgatatacaaaagttagtg

ggaaaattaaactgggcaagtcagatttatcctggaatyaaaacaaggcaactttgtaaa

ctccttagggggrycaaagcactaacagacatagtaccactaactgaagaagcagaatta

gaattggc----------------------------------------

>CRF07BC.HZ141128

cctcaaatcactctttggcaacgaccccttgttaccataaagataggggggcaattaaag

gaagctctattagatacaggagcagatgatacagtattagaagamatgaatttgccaggg

aaatggaaaccaaaaatgatagggggaattggaggttttatcaaagtaagacagtatgaa

cagataccyatagaaatctgtggacataaagctataggtacagtattaataggacctaca

cctrtcaacataattggaaggaatmtgttgactcagcttggttgtactttaaattttcca

atcagtcctattgaaactgtaccagtaaaattaaagccaggaatggatggsccaaaggtt

aaacaatggccattgacaaaagaaaaaatagaagcattaacagcaatttgtgatgaratg

gaaaaggaaggaaaaattwcaaaaatygggcctgaaaatccatacaacactccaatattt

gccataaaaaagaaagacagtactaagtggagaaaattagtagatttcagggaactcaat

aaaagaactcaagatttttgggaagttcaattaggaataccacatccagcaggattaaaa

aagaaaaaatcagtgacagtgctggatgtgggagatgcatatttttcagttcctttacat

gaagacttcaggaaatatactgcattcaccatacctagtataaacaatgaracaccaggg

attagatatcagtacaatgtacttccacagggatggaaaggatcaccagcaatatttcaa

agtagcatgacaaaaatyttagagccttttagaaaacaaaatccagacatagtcatctat

caatacatggatgatttgtatgtaggatctgacctagagatagggcagcatagaacaaaa

atagaggaactgagacaacatttgttgaggtggggatttaccacaccagacaagaaacat

cagaaagaacctccatttctttggatggggtatgaactccaycctgacaaatggacagta

cagcctatacagctrccagaacaagatagctggactgtcaatgatatacaaaagttagtg

ggaaaattaaactgggcaagtcagatttatcctggaattaaagtaaggcaactttgtaaa

ctccttaggggggccaaagcactaacagacatagtaccact-------------------

------------------------------------------------

>CRF07BC.HZ141161

cctcaaatcactctttggcaacgaccccttgttaccataaagataggggggcaattaaag

gaagctctattagatacaggagcagatgatacagtattagaggacatgaatttgccaggg

aaatggaaaccaaaaatgatagggggaattggaggttttatcaaagtaagacagtatgaa

caggtacccatagaaatttgtggacataaagctataggtacagtattaataggacctaca

cctgtcaacataattggaagaaatctgttgactcagcttggttgtactttaaattttcca

atcagtcctattgaaactataccagtaaaactaaagccaggaatggatggcccaaaggtt

aaacaatggccattgacaaaagagaaratagaagcattaacagcaatttgtgatgaaatg

gaaaaagaaggaaaaattacaaaaattgggcctgaaaatccatacaacactccaatattt

gccataaaaaagaaagacagcactaagtggagaaaattagtagatttcagggaactcaat

aaaaggactcaagatttttgggaagttcaattaggaataccacatccagcaggattaaaa

aagaaaaaatcagtgacagtgctggatgtgggggatgcatatttttcagttcctttatat

gaagatttcaggaaatatactgcattcactatacctagtataaacaatgaaacaccaggg

attaggtaccagtacaatgtacttccacagggatggaaaggatcaccagcaatatttcaa

agtagcatgacaaaaatcttagagccttttagaaaacaaaatccagacatagtcatctat

caatacatggatgatttgtacgtaggatctgacttagagatagggcaacacagaacaaaa

atagaggaactgagacaacatttgttgaagtggggatttaccacaccagacaagaaacat

cagaaagaacctccatttctttggatggggtatgaactccatcctgacaaatggacagta

cagcctatacagctgccagtacaagatagctggactgtcaatgatatacaaaagttagtg

ggaaaattaaactgggcaagtcagatttatcctggaattaaggtaaggcaactttgtaaa

ctccttagggggaccaaagcactaacagacatagtaccactaactgaagaagcagaa---

------------------------------------------------

>CRF07BC.HZ141477

cctcaaatcactctttggcaacgaccccttgttaccataaaaataggggggcaattaaag

gaagctctattagatacaggagcagatgatacagtattagaagacatgaatttgccaggg

aaatggaaaccaaaaatgatagggggaattggaggttttatcaaagtaagacagtatgaa

cagatacccatagaaatctgtggacataaagctataggtacagtattagtaggacctaca

cctgtcaacataattggaagaaatctgttgactcagcttggttgtactttaaattttcca

atcagtcctattgaaactgtaccagtaaaactaaagccaggaatggatggcccaaaggtt

aaacaatggccattgacaaaagagaaaatagaagcattaacagcaatttgtgatgaaatg

gaaaaggaaggaaaaatttcaaaaattgggcctgaaaatccatacaacactccaatattt

gccataaaaaagaaagacagcactaagtggagaaaattagtagatttcagggaactcaat

aaaagaactcaagatttttgggaagttcaattaggaataccacacccagcaggattaaaa

aagaaaaaatcagtgacagtgctggatgtgggggatgcatatttttcagttcctttatat

gaagacttcaggaaatatactgcattcaccatacctagtataaacaatgaaacaccaggg

attaggtatcagtataatgtacttccacagggatggaaaggatcaccagcaatatttcaa

agtagcatgacaaaaatcttagagccttttagaaaacaaaatccagacatagtcatctat

caatacatggatgatttgtatgtaggatctgacttagagatagggcagcatagaacaaaa

atagaggaactaagacaacatttgttgaggtggggatttaccacaccagacaagaaacat

cagaaagaacctccatttctttggatggggtatgaactccatcctgacaaatggacagta

cagcctatacagctgccagtacaagatagctggactgtcaatgatatacaaaagttagtg

ggaaaattaaactgggcaagtcagatttatcctggaattaaagtaaggcaactttgtaaa

ctccttagggggaccaaagcactaacagacatagtaccactaactgaagaagcagaa---

------------------------------------------------

>CRF07BC.HZ141479

cctcaaatcactctttggcaacgaccccttgtcacaataaagataggggggcaattaaag

gaagctctattagatacaggagcagatgatacagtattagaagacatgaatttgccagga

aaatggaaaccaaaaatgatagggggaattggaggttttatcaaagtaagacagtatgaa

cagatacccgtagaaatctgtggacataaagcaataggtacagtattagtgggacctaca

cctgtcaacataattggaagaaatctgttgactcaacttggttgtactttaaattttcca

atcagtcctattgaaactgtaccagtaaaactaaagccaggaatggatggcccaaaggtt

aaacaatggccattgacaaaagaaaaaatagaagcattaacagcaatttgtgatgagatg

gaaaaggaaggaaaaattacaaaaattgggcctgaaaatccatacaacactccaatattt

gccataaaaaagaaagacagtactaagtggagaaaattagtagatttcagggaactcaat

aaaagaactcaagatttttgggaagttcaattaggaataccacacccagcaggattaaaa

aagaaaaaatcagtgacagtgctggatgtgggggatgcatatttttcagttcctttatat

gaagacttcagaaaatatactgcattcaccatacctagtataaacaatgaaacaccaggg

attaggtatcagtacaatgtacttccacagggatggaaaggatcaccagcaatatttcaa

agtagcatgacaagaatcttagagccttttagaaaacaaaatccagacatagtcatctat

caatacatggatgatttgtatgtaggatctgacttagagatagggcagcatagaacaaaa

atagaggaactgagacaacatttgttgaggtggggatttaccacaccagacaaraaacat

cagaaagaacctccatttctttggatggggtatgagctccatcctgacaaatggacagta

cagcctatacagctgccagtacaagatagctggactgtcaatgatatacaaaagttagtg

ggaaaattaaactgggcaagtcagatttatcctggaattaaagtaaggcaactttgtaaa

ctccttaggggggccaaagcactaacagacatagtac-----------------------

------------------------------------------------

>CRF07BC.HZ141492

cctcaaatcactctttggcaacgacccatygttaccataaaggtaggggggcaattaaag

gaagctctattagatacaggagcagatgatacagtattagaagacatgaatttgccaggg

aaatggaaaccaaaaatgatagggggaattggaggttttatcaaagtaagacagtatgaa

cagatacccatagaaatctgtggacataaagctataggtacagtattaatagggcctaca

cctgtcaacataattggaagaaatctgttgactcagcttggttgtactttaaattttcca

atcagtcctattgaaactgtaccagtaaaactaaagccaggaatggatggcccaaaggtt

aaacaatggccattgacaaaagagaaaatagaagcattaacagcaatttgtgatgaaatg

gaaaaggaaggaaaaattacaagaattgggcctgaaaatccatacaacactccaatattt

gccataaaaaagaaagacagtactaagtggagaaaattagtagatttcagggaactcaat

aaaagaactcaagatttttgggaggttcaattaggaataccacacccagcaggattaaaa

aagaaaaaatcagtgacagtgctggatgtgggggatgcatatttttcagttcctttatat

gaagacttcaggaaatatactgcattcaccatacctagtataaacaatgaaacaccwggg

attaggtatcagtacaatgtacttccacagggatggaaaggatcaccagcaatatttcaa

agtagcatgacaaaaatcytagagccttttagaaaacaaaatccagacatagtcatctat

caatacatggatgatttgtatgtaggatcwgacttagagatagggcagcatagaacaaaa

rtagaggaactgagacaacatttgttaaggtggggatttaccacaccagataagaaacat

cagaaagaacctccatttctttggatggggtatgaactccatcctgacaaatggacagta

cagcctatacagctaccagtacaagatagctggactgtcaatgatatacaaaagttagtg

ggaaaattaaactgggcaagtcagatttatcctggaattaaaataaggcaactttgtaag

ctccttaggggggccaaagcactaacagacatagtaccactaactgaagaagcaga----

------------------------------------------------

>CRF07BC.HZ141497

cctcaaatcactctttggcaacgaccccttgttaccataaagataggggggcaattaaag

gaagctctactagatacaggagcagatgatacagtattagaagacatgaatttgccaggg

aaatggaaaccaaaaatgatagggggaattggaggttttatcaaagtaagacagtatgaa

cagatacccatagaaatctgtggacataaagctataggtacagtattagtaggacctaca

cctgtcaacataattggaagaaatctgttgactcagcttggttgtactttaaattttcca

atcagtcctattgaaactgtaccagtaaaactaaagccaggaatggatggcccaaaggtt

aaacaatggccattaacaaaagagaaaatagaagcattaacagcaatttgtgatgaaatg

gaaaaggaaggaaaaattacaaaaattgggcctgaaaatccatacaacactccaatattt

gccataaaaaagaaagacagtactaagtggagaaaattagtagatttcagggaactcaat

aaaagaactcaagatttttgggaagttcaattaggaataccacacccagcaggactaaaa

aagaaaaaatcagtgacagtgctggatgtgggggatgcatatttttcagttcctttatat

gaagacttcaggaaatatactgcattcaccatacctagtataaacaatgaaacaccaggg

attaggtatcagtacaatgtactkccacagggatggaaaggatcaccagcaatatttcaa

agtagtatgataaaaatcttagagccttttagaaaacaaaatccagacatagtcatctat

caatacatggatgatttgtatgtaggatctgacttagagatagggcagcatagaacaaaa

atagaggaactgagacaacatttgttgaggtggggatttaccacaccagacaagaaacat

cagaaagaacctccatttctttggatggggtatgaactccatcctgacaaatggacagta

cagcctatacagctaccagtacaagatagctggactgtcaatgatatacaaaagttagtg

ggaaaattaaactgggcaagtcagatttatcctggaattaaagtaaggcaactttgtaaa

ctccttaggggggccaaagcactaacagacatagtaccactaactgaagaagcagaatta

g-----------------------------------------------

>CRF07BC.HZ141941

cctcaaatcactctttggcaacgaccccttgttmcmataaagatagggggrcarttaaar

gaagctctattagatacaggagcagatgatacagtattagaagacatgaatttgccaggg

aaatggaaaccaaaaatgatagggggaattggaggttttatcaaagtaagacagtatgaa

cagatacccatagaaatctgtggacataaagctataggtacagtattagtaggacctaca

cctgtcaayataattggaagraatctgttgactcagcttggttgtacyttaaattttcca

atcagtcctattgaaactgtaccagtaaaactaaagccaggaatggatggcccaaaggtt

aaacaatggccattgacaaaagagaaaatagargcattaacagcaatttgtgatgaaatg

gaaaaggaaggaaaaattacaaaaattgggcctgaaaatccatacaacactccaatattt

gccataaaaaagaaagacagtactaagtggagaaaattagtagatttcagggaactcaat

aaaagaactcaagatttttgggaagtycaattaggaataccrcacccagcaggattaaaa

argaaaaaatcagtgacagtgctggatgtgggggatgcmtatttttcagttcckttakat

raagacttcaggaaatatactgcattcaccatacctagtataaayaatgaaacaccaggg

attaggtaycagtacaatgtacttccacarggatggaaaggatcaccagcaatatttcaa

agtagcatgacaaaaatcttagagccttttagaaaacaaaatccagacatagtcatctat

cartacatggatgatttgtatgtaggatctgacttagagatagggcagcatagaacaaaa

atagaggaactgagacaacatttgttgargtggggatttaccacaccagacaagaaacat

cagaaagaaccyccatttctttggatggggtatgaactccatcctgacaaatggacagta

cagcctatacagctrccagtacaagatagctggactgtyaatgatatacaaaagttrgtg

ggaaaattaaactgggcaagtcagatttatcctggaattaaagtaaggcaactttgtaaa

ctccttagrggggccaaagcactaacagacatagtaccactaactgaagaagcag-----

------------------------------------------------

>CRF07BC.HZ141942

cctcaaatcactctttggcaacgaccccttgttgccgtaaagataggggggcaattaaag

gaagctctattagatacaggagcagatgatacagtattagaagacatgaatttgccaggg

aaatggaaaccaaaaatgatagggggaattggaggttttatcaaagtaagacaatatgaa

cagatacccatagaaatctgtggacataaagttataggtacagtattagtaggacctaca

cctgtcaacataattgggaggaatctgttgactcagcttggttgtactttaaattttcca

attagtcctattgaaactgtaccagtaaarttaaagccaggaatggatggcccaaaggtt

aaacaatggccattgacaaaagagaaaatagaagcattaacagcaatttgtgatgaaatg

gaaaaggaaggaaaaattacaaaaattgggcctgaaaatccatacaacactccaatattt

gccataaaaaagaaagacagtactaagtggagaaaattagtagatttcagggaactcaat

aaaagaactcaagatttttgggaagttcaattaggaataccacatccagcaggattaaga

aagaaaaaatcagtgacagtgctggatgtgggagatgcatatttttcagtwcctttatat

gaagacttcaggaaatatactgcattcaccatacctagtataaataatgaaacaccaggg

attagatatcagtacaatgtacttccacagggatggaaaggatcaccagcaatatttcaa

agtagcatgacaaaaatcttagagccttttagaaaacaaaatccagacatagtcatctat

caatacatggatgatttgtatgtaggatctgacttagagataggrcagcatagaacaaaa

atagaggaactgagacaacatttgttgaggtggggatttaccacaccagacaagaaacat

cagaaagaacctccatttctttggatggggtatgaactccatcctgacaaatggacagta

cagcctatacagttgccagtacaagatagctggackgtcaatgatatacaaaagttagtg

ggaaaattaaactgggcmagtcagatttatcctggaattaaagtaaggcaactttgtaaa

ctccttaggggggccaaagcactaacagacatag--------------------------

------------------------------------------------

>CRF07BC.HZ141943

cctcaaatcactctttggcaacgaccccttgttaccataaggataggggggcaattaaag

gaggctctattagatacaggagcagatgatacagtattagaagacatgaatttgccaggg

aaatggaaaccaaaaatgatagggggaattggaggttttatcaaagtgagacagtatgaa

cagatacccatagaaatctgtggacataaagctataggtacagtattartaggacctaca

cctgtcaacataattggaaggaatctgttgactcagcttggttgtactttaaatttccca

atyagccctattgaaactgtaccagtaaaactaaagccaggaatggatggcccaaaggtt

aaacaatggccattgacaaaagaaaaaatagaagcattaacagcaatttgtgatgaaatg

gaaaaggaaggaaaaattacaaaaattggacctgaaaacccatacaacactccaatattt

gccataaaaaagaaagacagtactaagtggagaaaattagtagatttcagggaactcaat

aaaagaactcaagatttttgggaagttcaattaggaataccacacccagcaggattaaaa

aagaaaaaatcagtgacagtgttggatgtgggggatgcctatttttcagttcctttatat

gaagacttcaggaaatatactgcattcaccatacctagtataaacaatgaaacaccaggg

attagatatcagtataatgtacttccacagggatggaaaggatcaccagcaatatttcaa

agtagcatgacaaaaatcttagagccttttagaaaacaaaatccagacatagtcatctat

caatacatggatgatttgtatgtaggatctgacttagagatagggcagcatagaacaaaa

atagaggaactgagagaacatttgttgaggtggggatttaccacaccagacaagaaacat

cagaaagaacctccatttctttggatggggtatgaactccatcctgacaaatggacagta

cagcctatacagctgccagtacaagatagctggactgtcaatgatatacaaaagttagtg

ggaaaattaaactgggcaagtcagatttatcctggaattaaagtaaggcaactttgtaaa

ctccttagggggaccaaagcactaacagacatagtgccactaactgaagaagcagaa---

------------------------------------------------

>CRF07BC.HZ150008

cctcaaatcactctttggcaacgaccccttgtcacaataaaaataggggggcaattaaag

gaagctctattagatacaggagcagatgatacagtattagaagacatggatttgccaggg

aaatggaaaccaaaaatgatagggggaattggaggttttatcaaagtaagacagtatgaa

cagatacccatagaaatctgtggacataaagctataggtacagtattagtaggacctaca

cctgtcaacataattggaagaaatttgttgactcagcttggttgtactttaaattttcca

atcagtcctattgaaactgtaccagtaaaactaaarccaggaatggatggcccaaaggtt

aaacaatggccattgacaaaagagaaaatagaagcattaacagcaatttgtgatgaaatg

gaaaaggaaggaaaaattacaaaaattgggcctgaaaatccatacaacactccaatattt

gccataaaaaagaaagacagtactaagtggagaaaattagtagatttcagggaactcaat

aaaagaactcaagatttttgggaagttcaattaggaataccacacccagcaggattaaaa

aagaaaaaatcagtgacagtgctrgatgtgggggatgcatatttttcagttcctttatat

gaagacttcaggaaatatactgcattcaccatacctagtataaacaatgaaacaccaggg

attaggtatcagtacaatgtacttccacagggatggaaaggatcaccagcaatatttcaa

agtagyatgacmaaaatcttagagccttttagaaaacaaaatccagacataatcatctat

caatacatggatgatttgtatgtaggatctgacttagagayagggcagcatagaacaaaa

atagaggaactgagacaacatttgttgaggtggggatttaccacaccagacaagaaacat

cagaaagaacctccatttctttggatggggtatgaactccatcctgacaaatggacagta

cagcctatacagctgccagwacaagatagctggactgtcaatgatatacaaaagttagtg

ggaaaattaaactgggcaagtcagatttatcctggaattaaartaaggcaactttgcaaa

ctccttagggggaccaaagcactaacagacatagtaccactaactgaagaagc-------

------------------------------------------------

>CRF07BC.HZ150577

cctcaaatcactctttggcaacgaccccttgttaccataaaggtagggggacaattaaag

gaagctctattagatacaggagcagatgatacagtattagaagacatgaatttgccaggg

aaatggaaaccaaaaatgatagggggaattggaggttttatcaaagtaaaacaatatgaa

cagatacccrtagaaatctgtggacataaagctataggtacagtattaatagggcctaca

cctgtcaacataattggaagaaatctgttgactcagcttggttgtactttaaattttcca

atcagycctattgaaactgtaccagtaaaactaaagccaggaatggatggcccaaargtt

aaacaatggccactgacaaaagagaaaatagaagcattaacagcaatttgtgatgaaatg

gaaaaggaaggaaaaattacaaaaattgggcctgaaaatccatacaacactccaatattt

gccataaaaaagaaagacagtactaagtggagaaaattagtagatttcagggaacttaat

aaaagaactcaagatttttgggaagttcaattaggaataccacacccagcaggrttaaaa

aagaaaaaatcagtgacagtgctggatgtgggggatgcatatttttcagttcctttatat

gaagacttcaggaaatatactgcattcaccatacctagtataaacaatgaaacaccaggg

attaggtatcagtacaatgtacttccacagggatggaaaggatcaccagcaatatttcaa

agtagcatgacaaaaatcctagagccttttagaaaacaaaatccagacatagtcatytat

caatacatggatgatttgtatgtaggatctgacttagagatagggcagcatagaacaaaa

atagaggaactgagacaacatttgttgaggtggggatttaccacaccagayaagaaacat

cagaaagaacctccatttctttggatggggtatgagctccatcctgacaaatggacagta

cagcctatacagctaccagtacaggatagctggactgtcaatgatatacaaaagttagtg

ggaaaattaaactgggcaagtcagatttatcctggaattaaaataaggcaactttgtaag

ctccttaggggggccaaagcactaacagacatagtaccactaactgaagaagcagaat--

------------------------------------------------

>CRF07BC.HZ1510033

cctcaaatcactctttggcaacgacccattgtcccaataaagataggggggcaattaaag

gaagctctattagatacaggagcagatgatacagtattagaagacatgaatttgccaggg

aaatggaagccaaaaatgatagggggaattggaggttttatcaaagtaagacagtatgaa

cagatatctgtagaaatctgtggacatcaagctataggtacagtattagtaggacctaca

cctgtcaacataattggaagaaatctgttgactcagcttggttgtactttaaattttcca

atcagtcctattgaaactgtaccagtaaaactaaagccaggaatggatggcccaaaggtt

aaacaatggccattgacaaaagaaaaaatagaagcattaacaacaatttgtgatgaaatg

gaaaaggaaggaaaaattacaaaaattgggcctgaaaatccatacaacactccaatattt

gccataaaaaagaaagacagtactaagtggagaaaattagtagatttcagggaactcaat

aaaagaactcaagatttttgggaagttcaattaggaataccacacccagcaggattaaaa

aagaaaaaatcagtgacagtgctggatgtgggggatgcatatttttcagttcctttatat

gaagatttcaggaagtatactgcattcaccatacctagtataaacaatgaaacaccaggg

attaggtatcagtacaatgtacttccacagggatggaaaggatcaccagcaatatttcaa

agtagcatgacaaaaatcttagagcctttcagaaaacaaaatccagacatagtcatctat

caatacatggatgatttgtatgtaggatctgacttagagatagggcagcatagaacaaaa

atagaggaactgagacaacatttgttgaggtggggatttaccacaccagacaagaaacat

cagaaagaacctccatttctttggatggggtatgagctccatcctgacaaatggacagta

cagcctatacagctgccagtacaagatagctggactgtcaatgatatacaaaagttagtg

ggaaaattaaactgggcaagtcagatttatcctggaattaaggtaaggcaactttgtaaa

ctccttagggggaccaaatcactaacagaaatagtaacactaactgaagaa---------

------------------------------------------------

>CRF07BC.HZ1510037

cctcaaatcactctttggcarcgaccccttgtygcaataaagatagggggacaattaaag

gaagctctattagatacaggagcagatgatacagtattagaagacatgaatttgccaggg

aaatggaaaccaagaatgatagggggaattggaggttttatcaaagtaagacagtatgaa

cagatacccatagaaatctgtggacataaagctataggtacagtattagtaggacctaca

cctgtcaacataattggaagaaatctgttgactcagcttggttgtactttaaattttcca

atcagtcctattgaaactgtaccagtaaaactaaagccaggaatggatggcccaaaggtt

aaacaatggccattgacaaaagagaaaatagaagcattaacagcaatttgtgaggaaatg

gaaaaagaaggaaagattacaaaaattgggcctgaaaatccatacaacactccaatattt

gccataaaaaagaaagacagtactaagtggagaaaattagtagatttyagggaactcaat

aaaagaactcaagacttttgggaagttcaattaggaataccacacccagcaggattaaaa

aagaaaaaatcagtgacagtgctggatgtgggagatgcatatttttcagttcctttatat

gaagatttcaggaaatatactgcattcaccatacctagtataaacaatgaaayaccaggr

attagatatcagtacaatgtacttccacagggatggaaaggatcaccagcaatatttcaa

agtagcatgacaaaaatyttagagccttttagaaaacaaaatccagacatagtcatctat

caatacatggatgatttgtatgtaggatctgacttagagatagggcagcatagaacaaaa

atagaggaactgagacaacatttgttgaggtggggatttaccacaccagacaagaaacat

cagaaagaacctccatttctttggatggggtatgaactccatcctgacaaatggacagta

cagcctatacagcttccagtacaagatagctggactgtaaatgatatacaaaagttagta

ggaaaattaaactgggcaagtcagatttatcccggaattaaagtaaggcaactttgtaaa

ctccttaggggggccaaagcactaacagacatagtaccactaactgaagaagcagaatta

gaattggcag--------------------------------------

>CRF07BC.HZ1510040

cctcaaatcactctttggcaacgaccccttgttacaataaagataggggggcaattaaag

gaagctctattagatacaggagcagatgatacagtactagaagacatgaatttgccaggg

aaatggaaaccaaaaatgatagggggaattggaggttttatcaaagtaagacagtatgaa

cagatacccatagaaatttgtggacataaagctataggtacagtattaataggacctaca

cctgtcaacataattggaagaaatctgttgactcagcttggttgtactttaaattttcca

atcagtcctattgaaactgtaccagtaaaactaaagccaggaatggatggcccaaaggtt

aaacaatggccattgacaaaagagaaaatagaagcattaacagcaatttgtgatgaaatg

gaaaaggaaggaaaaattacaaaaattgggcctgaaaatccatacaacactccaatattt

gccataaaaaagaaagacagcactaagtggagaaaattagtagatttcagggaactcaat

aaaagaactcaagatttttgggaagttcaattaggaataccacacccagcaggattaaaa

aagaaaaaatcagtracagtgctggatgtgggggatgcatatttttcagttcctttacat

gaagacttcaggaaatatactgcattcaccatacctagtagaaacaatgaaacaccaggg

attaggtatcagtacaatgtacttccacagggatggaaaggatcaccagcaatatttcaa

agtagcatgacaaaaatcttagagccttttagaaaacaaaatccagaaatagtcatctac

caatacatggatgacttgtatgtaggatctgatttagagatagggcagcatagaacaaaa

atagaggaactgagacarcatttgttgaggtggggatttaccaccccagacaagaaacat

cagaaagaacctccatttctttggatggggtatgaactccatcctgacaaatggacagta

cagcctatacagctgccagtacaagacagctggactgtaaatgatatacaaaagttagtg

ggaaaattaaactgggcaagtcagatttatcctggaattaaagtaaggcaactttgtaaa

ctccttaggggggccaaagcactaa-----------------------------------

------------------------------------------------

>CRF07BC.HZ1510047

cctcaaatcactctttggcaacgaccccttgttaccataaagataggggggcaattaaag

gaagctctattagatacaggagcagatgatacagtattagaagacatgaatttgccaggg

aaatggaaaccaaaaatgatagggggaattggaggttttatcaaagtaagacagtatgaa

caggtacccatagaaatctgtggacataacgctataggtacagtattagtaggacctaca

cctgtcaacataattggaagaaatctgttgactcagattggttgtactttaaattttcca

atcagtcctattgaaactgtaccagtaaaactaaagccaggaatggatggcccaaaggtt

aaacaatggccattgacaaaagagaaaatagaagcattaacagcaatttgtgatgaaatg

gaaaaggaaggaaaaattacaaaaattgggcctgaaaatccatacaacactccaatattt

gccataaaaaagaaagacagtactaagtggagaaaattagtagatttcagggaactcaat

aaaagaactcaagatttttgggaagttcaattaggaataccacacccagcaggattaaaa

aagaaaaaatcagtgacagtgctggatgtgggggatgcatatttttcagttcctttacat

gaagacttcaggaaatatactgcattcaccatacctagtataaacaatgaaacaccaggg

attaggtatcagtacaatgtacttccacagggatggaaaggatcaccagcaatatttcaa

agcagcatgacaaaaatcttagagccttttagaaaacaaaatccagacatagtcatctat

caatacatggatgatttgtatgtaggatctgacttagagatagggcagcatagaacaaaa

atagaggaactgagacaacatttgttgaggtggggatttaccacaccagacaagaaacat

cagaaggarcctccatttctttggatggggtatgaactccatcctgataaatggacagtc

cagcctatacagctgccagtacaagatagttggactgtcaatgatatacaaaagttagtg

ggaaaattaaactgggcaagtcagatttatcctggaattaaagtaaggcaactttgtaaa

ctccttaggggggccaaagcactaacagacatagtaccactaactgaagaa---------

------------------------------------------------

>CRF07BC.HZ1510052

cctcaaatcactctttggcaacgaccccttgttaccataaagataggggggcaattaaag

gaagctctattagatacaggagcagatgatacagtattagaagacatggatttgccaggg

aaatggaaaccaaaaatgatagggggaattggaggttttatcaaagtaagacagtatgaa

gaaatacccatagaaatctgtggacataaagctataggtacagtattagtaggaccaaca

cctgtyaacataattggaaggaatctgttgactcagcttggctgtactttaaattttcca

atcagtcctattgaaactgtaccagtaaaattaaagccaggaatggatggcccaaaggtt

aaacaatggccattgacaaaagagaaaatagaagcattaacagcaatttgtgatgaaatg

gaaaaggaaggaaaaattacaaaaattgggcctgaaaatccatacaacactccaatattt

gccataaaaaagaaagacagtactaagtggagaaaattagtagatttcagggaactcaat

aaaagaactcaagatttttgggaagttcaattaggaataccacatccagcaggattaaaa

aagaaaaaatcagtgacagtgctagatgtgggagatgcatatttttcagttcctctagat

gaaaatttcaggaaatatactgcattcaccatacctagtataaacaatgaaacaccaggg

attagatatcagtacaatgtacttccacagggatggaaaggctcaccagcaatatttcaa

agtagcatgacaaaaatcttagagccttttagaaaacaaaatccagacatagtcatctat

cagtacatggatgatttgtatgtaggatctgacttagagatagggcagcatagaacaaaa

gtagaggaactgagactacatttgttgaagtggggatttaccacaccagacaagaaacat

cagaaagaacctccatttctttggatggggtatgaactccatcctgacaaatggacagta

cagcctatacagctgccagtacaagatagctggactgtcaatgatatacaaaagttagtg

ggaaaattaaactgggcaagtcagatttatcctggaattaaagtaaggcaactttgtaaa

ctccttagaggggccaaagcactaacagacatagtaccac--------------------

------------------------------------------------

>CRF07BC.HZ1510053

cctcaratcactctttggcaacgaccccttgtcacagtaaagataggggggcaattaaag

gaagctctattagatacaggagcagatgatacagtattagaagacatgaatttgccaggg

aaatggaaaccaaaaatgatagggggaattggcggttttatcaaagtaagacagtatgaa

cagatacccatagaaatctgtggacatacagttataggtacagtattagtaggacctaca

cctgtcaacataattggaagaaatctgttgactcagcttggttgtactttaaattttcca

atcagtcctattgamactgtaccagtaaaaytaaagycaggaatggatggcccmaaggtt

aaacaatggccattgacaaaagagaaaatagaagcattaacagcaatttgtgatgaaatg

gaaaaggaaggaaaaattacaaaaattgggcctgaaaayccatacaacactccaatattt

gccataaaaaagaaagacagtactaagtggagaaaattagtagatttcagggaactcaat

aaaagaackcaagatttttgggaagttcaattaggaataccacacccagcaggattaaaa

aagaaaaaatcagtgacagtgctggatgtgggrgatgcatatttttcagttcctttatat

gaagacttcaggaaatatactgcattcaccatacctagtataaayaatgaaacaccaggg

attaggtatcagtacaatgtacttccacagggatggaaaggatcaccagcaatatttcaa

agtagcatgacaaaaatcttagagccttttagaaaacaaaayccagacatagtcatctat

caatacatggatgatttrtatgtaggatctgacttagagatagggcagcatagarcaaaa

atagaggaactgagacaacatttgttgarrtggggatttaccacaccagacaagaaacat

cagaaagaacctccatttctttggatggggtatgaactccatccygacaaatggacagta

cagcctatacagctrccagtacaagatagctggactgtcaatgatatacaaaagttagtg

ggaaaattaaactgggcmagtcagatttatcctggaattaaagtaaggcaactytgtaaa

ctccttaggggggccaaagcactaacagacatagtaccactaactgaagaa---------

------------------------------------------------

>CRF07BC.HZ1510054

cctcaaatcactctttggcarcgaccccttgttacagtaaagataggggggcaattaaag

gaagctctattagatacaggagcagatgatacagtattagaagacatgaatttgccaggg

aaatggaaaccaagaatgatagggggaattggaggttttatcaaagtaagacagtatgaa

caggtacccatagaaatctgtggacataaagctataggtacagtattagtaggacctaca

cctgtcaacataattgggaggaatctgttgactcagattggttgtactttaaattttcca

atcagycctattgaaactgtaccagtaaaactaaagccaggaatggatggcccaaaggtt

aaacaatggccattgacaaaagaaaaaatagaagcattaacagcaatttgtgatgaaatg

gaaaaggaaggaaaaattacaaaaattgggcctgaaaatccatacaacactccaatattt

gccataaaaaagaaagacagtactaagtggagaaaattagtagatttcagggaactcaat

aaaagaactcaagatttttgggaggttcaattaggaataccacacccagcaggattaaaa

aagaaaaaatcagtgacagtgctggatgtgggggatgcatatttttcagttcctttacat

gaagacttcaggaaatatactgcattcaccatacctagtataaacaatgaaacaccaggg

attaggtatcagtacaatgtacttccacagggatggaaaggatcaccagcaatatttcaa

agcagcatgacaagaatcttagagccttttaggaaacaaaatccagacatagtcatctat

caatacatggatgatttgtatgtaggatctgacttagagatagggcagcatagaacaaaa

atagaggractgaggcaacatttgttgaggtggggatttaccacaccagacaagaaacat

cagaaagaacctccatttctttggatggggtatgaactccatcctgacaaatggacagta

cagcctatacagctgccagtacaagatagctggactgtcaatgatatacaaaagttagtg

ggaaaattaaactgggcaagtcagatttatcctggaattaaagtaaggcaactttgtaga

ctccttaggggggccaaagcactaacagacatagtaccact-------------------

------------------------------------------------

>CRF07BC.HZ1510077

cctcaaatcactctttggcaacgaccccttgttaccataaagataggggggcaattaaag

gaagctctattagatacaggagcagatgatacagtactagaagacatgaatttgccaggg

aaatggaaaccaaaaatgatagggggaattggaggttttatcaaagtaagacagtatgaw

cagrtacccrtagaaatctgtggacatmmagctataggtacagtattartaggacctaca

cctgtcaacataattggragaaacctgttgactcagattggttgtactttaaattttcca

atcagtcctattgaaactgtaccagtaaaaytaaagccaggaatggatggcccaaaggtt

aaacaatggccattracaaaagagaaaatagaagcattaacagcaatttgtgatgaaatg

gaaaaggaaggaaaaattacaaaaattgggcctgaaaatccatacaacactccaatattt

gccataaaaaagaaagacagtactaagtggagaaaattagtagatttcagggaactcaat

aaaagaactcaagatttttgggaagttcaattaggaataccacacccagcaggattaaaa

aagaaaaaatcagtgacagtgctggatgtgggggatgcatatttytcagttcctttagat

aaagacttcaggaaatatactgcattcaccatacctagtataaacaatgaaacaccaggg

attaggtatcagtacaatgtacttccacarggatggaaaggatcaccagcaatatttcaa

agtagcatgacaaaaatcttagagccttttagaaaacaaaatccagacatagtcatctat

caatacatggatgatttgtatgtaggatctgayytrgaaatagggcagcatagagcaaaa

atagargaactgagacaacatttgttgargtggggatttaccacaccagacaagaaacat

cagaaagaacctccrtttctttggatggggtatgaactccatcctgacaaatggacagta

cagcctatacagctgccagyacaagakagctggactgtcaatgatatacaaaagttagtg

ggaaaattaaactgggcaagtcagatttatcctggaattaaagtaaggcaactttgtaaa

ctccttaggggggccaaagcrctaacagacatagtaccactaactgaagaagcagaa---

------------------------------------------------

>CRF07BC.HZ1510080

cctcaaatcactctttggcaacgaccccttgtcacaataaaaataggggggcaattaaag

gaagctctattagatacaggagcagatgatacagtmttagaagacatgaatttgccagga

aaatggaaaccaaaaatgatagggggaattggaggttttatcaaagtaaaacagtatgaa

cagatacccgtagaaatctgtggacataaagctataggtacagtattagtaggacctaca

cctgtcaacataattggaagaaatctgttgactcagattggttgtactttaaattttcca

atcagtcctattgaaactgtaccagtaaaactaaagccaggaatggatggcccaaaggtt

aaacaatggccattgacaaaagaaaaaatagaagcattaacagtaatttgtgatgaaatg

gaaaaggaaggaaaaattacaaaaattgggcctgaaaatccatacaacactccaatattt

gccataaaaaagaaagacagtactaagtggagaaaattagtagatttcagggaactcaat

aaaagaactcaagatttttgggaagttcaattaggaataccacacccagcaggattaaaa

aagaaaaaatcagtgacagtgctrgatgtgggggatgcatatttttcagttcctttatat

gaagacttcaggaaatatactgcattcaccatacctagtataaacaatgaaacaccaggg

attaggtatcagtacaatgtacttccacagggatggaaaggatcaccagcaatatttcaa

agtagcatgacaagaatcttagagccttttagaaaacaaaatccagacatagtcatctat

caatacatggatgatttgtatgtaggatcagacttagagatagggcagcatagaacaaaa

atagaggaactgagacaacatttgttgaggtggggatttaccacaccagacaagaaacat

cagaaagaacctccatttctttggatggggtatgagctccatcctgacaaatggacagta

cagcctatacagctgccagtacaagatagctggactgtcaatgatatacaaaagttagtg

ggaaaattaaactgggcaagtcagatctatcctggaattaaagtaaggcaactttgtaaa

ctccttaggggggccaaagcactaacagacatagtaccactaactgaagaa---------

------------------------------------------------

>CRF07BC.HZ1510082

cctcagatcactctttggcaacgaccccttgttaccataaagataggggggcaattaaag

gaagctctattagatacaggagcagatgatacagtattagaagacatgaatttgccaggg

aaatggaaaccaaaaatgatagggggaattggaggttttatcaaagtaaggcagtatgaa

cagatacccatagacatctgtggacataaagctataggtacagtrttagtaggacctaca

cctgtcaacataattggaaggaatctgttgactcagattggttgtactttaaattttcca

atcagtcctattgaaactgtaccagtaaaattaaarccaggaatggatggcccaaaggtt

aaacaatggccattgacaaaagagaaaatagaagcattaacagcaatttgtgatgaaatg

gaaaaggaaggaaaaattacaaaaattgggcctgaaaatccatacaacactccaatattt

gccataaaaaagaaagacagtactaagtggagaaaattagtagatttcagggaactcaat

aaaagaactcaagatttttgggaagttcaattaggaataccacatccagcaggattaaaa

aagaaaaaatcagtgacagtgctggatgtgggagatgcatatttttcagtacctttatat

gaagacttcaggaaatatactgcattcaccatacctagtataaacaatgaaacaccaggg

attagatatcagtacaatgtacttccacagggatggaaaggatcaccagcaatatttcaa

agtagcatgacaaaaatcttagagccytttagaaaacaaaatccagacatagtcatctat

caatatatggatgatttgtatgtaggatctgacttagagatagggcagcatagaacaaaa

atagaggaactgagacaayatttgttgrsgtggggatttaccacaccagacaagaaacat

cagaaagaacctccatttctttggatggggtatgaactccatcctgacaaatggacagta

cagcctatacagctgccagtacaagatagctggactgtcaatgatatacaaaagttagtg

ggaaaattaaactgggcaagtcagatttatcctggaattaaagtaaggcaactttgtaaa

ctccttaggggrgccaaagcactaacagacatagtaccactaactgaa------------

------------------------------------------------

>CRF07BC.HZ1510088

cctcaaatcactctttggcaacgaccccttgtyacmataarrataggaggacarytaaar

gaagctctattagatacaggagcagatgatacagtattrgaagayatrratttgccaggr

aaatggaaaccaaaaatgataggrggaattggaggttttatcaargtaaarcartatgat

cagrtamyyatagaaatytgtgrgaaaaargytataggtacagtattagtaggrcctaca

cctgtcaacataattggamgaaatatgttgactcagattggktgtactttaaatttyccc

atyagtcctattgacactgtaccagtaaaattaaagccaggaatggatggmccaaaggtt

aaacaatggccattgacaraagaraaaataraagcattaacagmaatttgtrawgaratg

gaaraggaaggaaaaattwcaaaaattgggcctgaaaatccatayaacactccaatattt

gcyataaaraaraargacagcacyaartggagraaattrgtagatttyagggarctcaat

aaaagaactcargayttttgggargttcaattaggaataccgcayccagcaggattaaaa

aagaaaaaatcagtgacagtrctrgatgtgggagatgcatatttttcagttcctttakat

gaarrcttyagraartatactgcattcaccatacctagtataaacaatgaracaccaggg

attaggtatcagtacaatgtacttccacagggatggaaaggatcaccagcaatatttcaa

agtagcatgacaaaaatcttagagcctttcagaaaacaaaatccagacatagtcatctat

caatacatggatgatttgtatgtaggatctgacttagagatagggcagcatagaacaaaa

atagaggaactgagasaacatttgttgaggtggggatttaccacaccagacaagaaacat

cagaaagaacctccatttctttggatggggtatgaactccatcctgacaaatggacagta

cagcctatacagttrccagtacaagatagctggactgtcaatgatatacaaaagttagtg

ggaaaattaaactgggcaagtcagatttatcctggaattaaartaaggcaactttgtaaa

ctccttaggggggccaaagccctaacrgacatagtaccactaactgaagaagcaga----

------------------------------------------------

>CRF07BC.HZ1510133

cctcaaatcactctttggcaacgaccccttgtcacaataaaaataggggggcaattaaag

gaagctctattagatacaggagcagatgatacagtattagaagacatggatttgccaggg

aaatggaaaccaaaaatgatagggggaattggaggttttatcaaagtaagacagtatgaa

cagatacccatagawatctgtggacataaagctataggtacagtattagtaggacctaca

cctgtcaacataattggaagaaatttgttgactcagctgggttgtactttaaattttcca

atcagtcctattgaaactgtaccagtaaaactaaagccaggaatggatggcccaaaggtt

aaacaatggccattgacaaaagagaaaatagaagcattaacagcaatttgtgatgaaatg

gaaaaggaaggaaaaattacaaaaattgggcctgaaaatccatacaacactccaatattt

gccataaaaaagaaagacagtactaagtggagaaaattagtagatttcagggaactcaat

aaaagaactcaagatttttgggaagttcaattaggaataccacacccagcaggattaaaa

aagaaaaaatcagtgacagtgctggatgtgggggatgcatatttttcagttcctttatat

gaagacttcaggaaatatactgcattcaccatacctagtataaacaatgaaacaccaggg

attaggtatcagtacaatgtacttccacagggatggaaaggatcaccagcaatatttcaa

agtagcatgaccaaaatcttagagccttttagaaaacaaaatccagacataatcatctat

caatacatggatgatttgtatgtaggatctgacttagagatagggcagcatagaacaaaa

atagaggaactgagacaacatttgttgaggtggggatttaccacaccagacaagaaacat

cagaaagaacctccatttctttggatggggtatgaactccatcctgacaaatggacagta

cagcctatacagctgccagtacaagatagctggactgtcaatgatatacaaaagttagtg

ggaaaattaaactgggcaagtcagatttatcctggaattaaagtaaggcaactttgcaaa

ctccttagggggaccaaagcactaacagacatagtaccactaactgaagaagcaga----

------------------------------------------------

>CRF07BC.HZ1510709

cctcaaatcactctttggcaacgaccccttgtcacaataaaaataggggggcaattaaag

gaagctctattagatacaggagcagatgatacagtattagaagacatgaatttgccagga

aaatggaaaccaaaaatgatagggggaattggaggttttatcaaagtaagacagtatgaa

cagatatccgtagaaatctgtggacataaagctataggtacagtattagtaggacctaca

cctgtcaacataattggaagaaatctgttgacccagattggttgtactttaaattttcca

atcagtcctattgaaactgtaccagtaaaactaaagccaggaatggatggcccaaaggtt

aaacaatggccattgacaaaagaaaaaatagaagcattaacagtaatttgtgatgaaatg

gaaaaggaaggaaaaattacaaaaattgggcctgaaaatccatacaacactccaatattt

gccataaaaaagaaagacagtactaagtggagaaaattagtagatttcagggaactcaat

aaaagaactcaagatttttgggaagttcaattaggaataccacacccagcaggattaaaa

aagaaaaaatcagtgacagtgctagatgtgggggatgcatatttttcagttcctttatat

gaagacttcaggaaatatactgcattcaccatacctagtataaacaatgaaacaccaggg

attaggtatcagtacaatgtacttccacagggatggaaaggatcaccagcaatatttcaa

agtagcatgacaaaaatcttagagccttttagaaaacaaaatccagacatagtcatctat

caatacatggatgatttgtatgtaggatcagacttagagatagggcagcatagaacaaaa

atagaggaactgagacaacatttgttgaggtggggatttaccacaccagacaagaaacat

cagaaagaacctccatttctttggatggggtatgagctccatcctgacaaatggacagta

cagcctatacagctgccagtacaagatagctggactgtcaatgatatacaaaagttagtg

ggaaaattaaactgggcaagtcagatctatcctggaattaaagtaaggcaactttgtaaa

ctccttaggggggccaaagcactaacagacatagtaccactaactgaagaagcagaatta

------------------------------------------------

>CRF07BC.HZ1510721

cctcaaatcactctttggcaacgaccccttgttaccataaagataggggggcaattaaag

gaagctctattagatacaggagcagatgatacagtattagaagacatgaatttgccaggg

aaatggaaaccaaaaatgatagggggaattggaggttttatcaaagtaagacagtatgaa

caaatacccgtagaaatctgtggacataaagctataggtacagtattagtaggacctaca

cctgtcaacataattggaagaaatctgttgactcagcttggttgtactttaaattttcca

atcagtcctattgaaactgtaccagtaaaactaaagccaggaatggatggcccaaaggtt

aaacaatggccattgacaaaagagaaaatagaagcattaacagcaatttgtgatgaaatg

gaaaaggaaggaaaaattacaaaaattgggcctgaaaatccatacaacactccaatattt

gccataaaaaagaaagacagtactaagtggagaaaattagtagatttcagggaactcaat

aaaagaactcaagatttttgggaagttcaattaggaataccacacccagcaggattaaaa

aagaaaaaatcagtgacagtgctggatgtgggggatgcatatttttcagttcctttacat

gaagacttcaggaaatatactgcattcaccatacctagtataaacaatgaaacaccaggg

attaggtatcagtacaatgtactgccacagggatggaaaggctcaccagcaatatttcaa

agtagcatgacaaaaatcttggagccttttagaaaacaaaatcctgacatagtcatctat

caatacatggatgatttgtatgtaggatctgacttagagatagggcagcatagaacaaaa

atagaggaactgagacaacatctgttgaggtggggatttaccacaccagacaagaaacat

cagaaagaacctccatttctttggatggggtatgaactccatcctgacaaatggacagta

cagcctatacagctgccagaacaagatagctggactgtcaatgatatacaaaagttagtg

ggaaaattaaactgggcaagtcagatttatcctggaatcaaagtaaggcaactttgtaaa

ctccttaggggggccaaagcactaacagacatagtaccactaactgaagaagcaga----

------------------------------------------------

>CRF07BC.HZ1510808

cctcaaatcactctttggcaacgacccgttgttacaataaagatagggggacaatcaaag

gaagctctattagatacaggagcagatgatacagtattagaagacatgaatttgccagga

aaatggaaaccaaaaatgatagggggaattggaggttttatcaaagtacgacagtatgaa

caaatacccatagaaatctgtggacataaagctataggtacagtattagtaggacctaca

cctgtcaacataattggaaggaatctgttgactcagcttggttgtactttaaattttcca

atcagtcctattgaaactgtaccagtaaaactaaagccaggaatggatggcccaaaggtt

aaacaatggccattgacaaaagagaaaatagaagcattaacagcaatttgtgatgaaatg

gaaaaggaaggaaaaattacaaaaattgggcctgaaaatccatacaacactccaatattt

gccataaaaaaraaagacagtactaagtggagaaaattagtagatttcaragagctcaat

aaaagaactcaagatttttgggaagttcaattaggaataccacacccagcaggattaaaa

aaraaaaaatcagtgacagtgctggatgtgggggatgcatatttttcagttcctttatat

gaagacttcaggaaatatactgcattcaccatacctagtataaacaatgaaacaccaggg

attaggtatcagtacaatgtacttccacagggatggaaaggatcaccagcaatatttcaa

agtagcatgacaaaaattttagagccttttagaaaacaaaatccagacatagtcatctat

caatacatggatgatttgtatgtaggatctgacttagagatagggcaacatagaacaaaa

atagaggaactgagacaacatttgttgaggtggggatttaccacaccagacaagaaacat

cagaaagaacctccatttctttggatggggtatgaactccatcctgacaaatggacagta

cagcctatacagctgccagtacaagatagctggactgtcaatgatatacaaaagttagtg

ggaaaattaaactgggcaagtcagatttatcctggaattaaagtaaggcaactttgtaaa

ctccttaggggggccaaa------------------------------------------

------------------------------------------------

>CRF07BC.HZ1510809

cctcaaatcactctttggcaacgaccccttgttaccataaagatakgggggcaagtaaag

gaagctctattagatacaggagcagatgatacagtattagaagacatgaatttgccaggg

aaatggaaaccaaaaatgatagggggaattggaggttttatcaaagtaagacagtatgaa

cagatacccatagaaatctgtggrcataaagctataggtacagtattagtaggacctaca

cctgtcaacataattggaagaaatctgttgactcagcttggttgtactttaaattttcca

atcagtcctattgaaactgtaccagtaaaactaaagccaggaatggatggcccaaaggtt

aaacaatggccattgacaaaagagaaaatagaagcattaacagcaatttgtgacgaaatg

gaaaaggaaggaaaaattacaaaaattgggcctgaaaatccatacaacactccaatattt

gccataaaaaagaaagacagtactaagtggagaaaattagtagatttcagggaactcaat

aaaagaaccmaagatttttgggaagttcaattaggaataccacacccagcaggattaaaa

aagaaaaaatcagtgacagtgctggatgtgggggatgcatatttttcagttcctttatat

gaagacttcaggaaatatactgcattcaccatacctagtgtaaacaatgaaacaccaggg

actaggtatcagtacaatgtactcccacagggatggaarggatcaccagcaatatttcaa

agtagtatgacaaaaatcttagaaccttttagaaaacaaaatccagacatagtcatctat

caatacatggatgatttgtatgtakgatctgacttagagatagggcaacatagaacaaaa

atagaggaacwaagacaacatttgttgaggtggggatttaccacaccagacaagaaacat

cagaaagaacctccatttctttggatggggtatgaactccatcctgacaaatggacagta

cagcctatacagctgccagtacaagatagctggactgtcaatgatatacaaaagttagtg

ggaaaattaaactgggcaagtcagatttatcctggaattagagtaaggcaactgtgtaaa

ctccttagggggaccaaagcactaacagacatagtaccattaactgaa------------

------------------------------------------------

>CRF07BC.HZ1510829

cctcaaatcactctttggcaacgaccccttgttaccataaagataggggggcaattaaag

gaagctctattagatacaggagcagatgatacagtattagaagacatgaatttgccaggg

aaatggaaaccaaraatgatagggggaattggaggttttatcaaagtaagacagtatgaa

cagatacccatagaaatatgtggacataaagctataggtacagtattaataggacctaca

cctgtcaacataattggaagaaatctgttgactcagcttggttgtactttaaattttcca

atcagtcctattgaaactgtaccagtaaaactaaagccaggaatggatggcccaaaggtt

aaacaatggccattgacaaaagagaaaatagaagcattaacagcaatttgtgakgaaatg

gaaaaggaaggaaaaattacaaaaattgggcctgaaaatccatataacactccaatattt

gcyataaaaaagaaagacagtactaagtggagaaaattagtagatttcagagaactcaat

aaaagaactcaagatttttgggaagttcaattaggaataccacacccagcaggtttaaaa

aagaaaaaatcagtgacagtgctggatgtgggggatgcatatttttcagttcctttatat

gaagacttcaggaaatatactgcattcaccatacctagtataaataatgaaacaccaggg

attaggtatcagtacaatgtacttccacarggatggaaaggatcaccagcaatatttcaa

agtagcatgacaaaaatcttagagccttttagaaaacaaaatccagayatagtcatytat

caatacatggatgatttgtatgtaggatctgacttagagatagggcagcatagaacaaaa

atagaggaactgagacaacatttgttgagatggggatttaccacaccagacaagaaacat

cagaaagaacctccatttctttggatggggtatgaactccatcctgacaaatggacagta

cagcctatacagctgccagtacaagatagctggactgtcaatgatatacaaaagttagtg

ggaaaattaaactgggcaagccagatttatcctggaattaaagtaaggcaactttgtaaa

ctccttaggggggccaaagcactaacagacatagtaccactaactgaagaagcagaatta

------------------------------------------------

>CRF07BC.HZ1510835

cctcaaatcactctttggcaacgaccccttgttgccataaagataggggggcaattaaag

gaagctctattagatacaggagcagatgatacagtattagaagacatgaatttgccaggg

aaatggaaaccaaaaatgatagggggaattggaggttttatcaaagtaagacagtatgac

cagatacccatagaaatctgtggacataaaactataggtacagtattagtaggacctaca

cctgtcaacataattggaagaaatttgttgactcagcttggttgtactttaaattttcca

atcagtcctattgaaactgtaccagtgaaactaaagccaggaatggatggcccaaaggtt

agacaatggccactgacaaaagaaaagatagaagcattaacagcaatttgtgatgaaatg

gaaaaggaaggaaaaattacaagaattgggcctgaaaatccatacaacactccaatattt

gccataaaaaagaaagacagtactaagtggagaaaattagtagatttcagggaactcaat

aaaagaactcaagatttttgggaagttcaattaggaataccacacccagcaggcttaaaa

aagaaaaaatcagtgacagtgctggatgtgggggatgcatatttttcagttcctttatat

gaagacttcaggaaatatactgcattcaccatacctagtacaaacaatgaaacaccaggg

attaggtatcagtacaatgtacttccacagggatggaaaggatcaccagcaatatttcaa

agtagcatgacaagaatcttagagccttttagaaaacaaaatccagacataatcatctat

caatacatggatgatttgtatgtaggatctgacttagagatagggcagcatagaacaaaa

atagaggaactaagacaacatttgttaaggtggggatttaccacaccagacaagaaacat

cagaaagaacctccatttctttggatggggtatgaactccatcctgacaaatggacagta

cagcctatacagctgccagtacaagatagctggactgtcaatgatatacaaaagttagtg

ggaaaattaaactgggcaagtcagatttatcctggaattaaagtaaggcaactttgtaaa

ctccttaggggggccaaagcactaacagacatagtaccactaactgaagaagcagaatta

gaa---------------------------------------------

>CRF07BC.HZ1510848

cctcaaatcactctttggcaacgaccccttgtcacaataaagataggggggcaattaaag

gaagctctattagatacaggagcagatgatacagtattggaagacatgaatttgccaggg

aaatggaaaccaaaaatgatagggggaattggaggttttatcaaagtaagacagtatgaa

gagatacccatagaaatctgtggacataaagctataggtacagtattagtaggacctaca

cctgtcaacataattggaagaaatctgttgactcagcttggttgtactttaaattttcca

atcagtcctattgaaactgtaccagtaaaactaaagccagggatggatggcccaaaggtt

aaacaatggccattgacaaaagagaaaatagaagcattaacagcaatttgtgaagaaatg

gaaaaggaaggaaaaattacaaaaattgggcctgaaaatccatacaacactccaatattt

gccataaaaaagaaagacagtactaagtggagaaaattagtagatttcagggaactcaat

aaaagaactcaagatttttgggaagttcaattaggaataccacacccagcaggattaaaa

aagaaaaaatcaataacagtgctggatgtgggggatgcatatttttcagttcctttatat

gaagacttcaggaaatatactgcattcaccatacctagtataaacaatgaaacaccaggg

attaggtatcagtacaatgtacttccacaaggatggaaaggatcaccagcaatatttcaa

agtagcatgacaaaaatcttagagccttttagaaaacaaaatccagacatagtcatctat

caatacatggatgatttgtatgtaggatctgacctagagatagggcagcacagaacaaaa

atagaggaactgagacaacatttgttgaggtggggatttaccacaccagacaagaaacat

cagaaagaacctccgtttctttggatggggtatgaactccatcctgacaaatggacagta

cagcctatacagctgccagtacaagatagctggactgtcaatgatatacaaaagttagtg

ggaaaattaaactgggcaagtcagatttatcctggaattaaagtaaagcaactttgtaaa

cttcttaggggggccaaagcactaacagacatagtaccactaactgaagaagcagaatta

------------------------------------------------

>CRF07BC.HZ1510849

cctcaaatcactctttggcaacgaccccttgttaccataaagataggggggcaattaaag

gaagctctattagatacaggagcagatgatacagtaatagaagaaataaatttgccaggg

aaatggaaaccaaaaatgatagggggaattggaggttttatcaaagtaagacagtatgaa

cagatacccatagaaatctgtggacataaagctatmggtacagtattagtaggacctaca

cctgtcaacataattggaagaaatctgttgactcagcttggttgtacyttaaattttcca

atcagtcctattgaaactgtaccagtaaaactaaagccaggaatggatggcccaaaggtt

aaacaatggccattgacaaaagagaaaatagaagcattaacagcaatttgtgatgaaatg

gaaaaggaaggaaaaattacaaaaattgggcctgaaaatccatacaacactccaatattt

gccataaaaaagaaggacagtactaaatggagaaaattagtagatttcagggaactcaat

aaaagaactcaagatttttgggaagttcaattaggaataccgcacccagcaggattaaaa

aagaaaaaatcagtaacagtgctggatgtgggggatgcatatttttcagttcctttacat

gaagacttcaggaaatatactgcattcaccatacctagtataaacaatgaaacaccaggg

attaggtatcagtacaatgtacttccacaaggatggaaaggatcaccrgcaatatttcaa

agtagcatgacaaaaatcttagagccttttagaaaacaaaatccagrcatagttatctat

caatatatggatgatttgtatgtaggatctgacttagagataggacagcatagaacaaaa

atagaggaactgagacaacatttgttkrggtggggatttaccacaccagataagaaacat

caaaaagaacctccatttctttggatggggtatgaactccatcctgacaaatggacagta

cagcctatacatctgccagaacaagatagctggactgtcaatgatatacaaaagttagtg

gggaagttaaactgggcaagtcagatttatcctggaattaaagtaagacaactttgtaaa

ctccttaggggggccaaaggactaacagaaatagtaacactaactgaagaagcagaatta

------------------------------------------------

>CRF07BC.HZ1510893

cctcagatcactctttggcaacgacccttagttacaataaagataggggggcaattaaag

gaagctctattagatacaggagcagatgatacagtattagaagacatggatttgccagga

aaatggaaaccaaaaatgatagggggaattggaggttttatcaaagtaagacagtatgaa

caaatacccatagaaatttgtggacataaagctataggtacagtattaatagggcctaca

cctgtcaacataattggaagaaatctgttgactcagcttgggtgtactttaaattttcca

atcagtcctattgaaactgtaccagtaaaattaaagccaggaatggatggtccaaaggtt

aaacaatggccattgacaaaagagaaaatagaagcattaacagcaatttgtgatgaaatg

gaaaaggaaggaaaaatttcaaaaattgggcctgaaaacccatacaacactccaatattt

gccataaaaaagaaagacagtactaagtggagaaaattagtagatttcagggaactcaat

aaaagaactcaagatttttgggaagttcaattaggaataccacacccagcaggattaaaa

aagaaaaaatcagtaacagtgctggatgtgggggatgcatatttttcagttcctttatat

gaagacttcaggaaatatactgcgttcactatacctagtataaacaatgaaacaccaggg

attaggtatcagtacaatgtacttccacagggatggaaaggatcaccagcaatatttcaa

agtagcatgacaaaaatcttagagccttttagaaaacaaaatccagacatagtcatctat

caatacatggatgatttgtatgtaggatctgacttagagatagggcagcatagaacaaaa

atagaggaactgagacaacatttgttgaggtggggatttaccacaccagacaagaaacat

cagaaagaacctccatttctttggatggggtatgaactccatcctgacaaatggacagta

cagcctatacagttgccagtacaagatagctggactgtcaatgatatacaaaagttagtg

ggaaaattaaactgggcaagtcagatttatcctggaattaaaataaggcaactttgtaaa

cttcttaggggggccaaagcactaacagacatagtaccactaactgaagaagcagaa---

------------------------------------------------

>CRF07BC.HZ1510897

cctcaaatcactctttggcaacgaccccttgtctcaataaagataggggggcaactaaag

gaagccctattagatacaggagcagatgatacagtattagaagaaatgaatttgccaggg

aaatggaaaccaaaaatgatagggggaattggaggttttatcaaagtaagacagtatgaa

cagatacccatagaaatctgtggacataaaactataggtacagtattaataggacctaca

cctgtcaacataattggaagaaatctgttgactcagcttggttgtactttaaattttcca

atcagtcctattgaaactgtaccagtaaaactaaagccaggaatggatggcccaaaggtt

aaacaatggccattgacaagagaaaaaatagaagcattaacagcaatttgtgatgaaatg

gaaaaggaaggaaaaattacaaaaattgggcctgaaaatccatacaacactccaatattt

gctataaaaaagaaagacagtactaagtggagaaaattagtagatttcagggaactcaat

aaaagaactcaagatttttgggaagttcaattaggaataccacacccagcaggattaaaa

aagaaaaaatcagtgacagtgctggatgtgggggatgcatatttttcagttcctttacat

gaagacttcaggaaatatactgcatttaccatacctagtataaataatgaaacaccaggg

attaggtatcagtacaatgtacttccacagggatggaaaggctcaccagcaatatttcaa

agtagcatgacaaaaatcttagagccttttagaaaccaaaatccagatatagtcatctat

caatacatggatgatttgtatgtaggatctgacttagagatagggcaacatagaataaaa

atagaggaactgagacaacatttgttggggtggggatttaccacaccagacaagaaacat

cagaaagaacctccatttctttggatggggtatgaactccatcctgacaaatggacagta

cagcctatacagctgccagaacaagatagctggactgtcaatgatatacaaaagttagtg

ggaaaattaaactgggcaagtcaaatttatcctggaattaaagtaaggcaactttgtaaa

ctccttaggggggccaaagcactaacagacatagtaccactaactgaagaagcagaatta

------------------------------------------------

>CRF07BC.HZ1510913

cctcaaatcactctttggcaacgaccccttgttgccataaaggtaggggggcaagtaaag

gaggctctattagatacaggagcagatgatacagtattagaagacatgaatttgccagga

aaatggaaaccaaaaatgatagggggaattggaggttttatcaaagtgaaacagtatgaa

cagatacccatagaaatctgtggacataaagctataggtacagtattagtaggacctaca

cctgtcaacataattggaaggaacctgttgactcagcttggttgtactttaaatttccca

atcagccctattgaaactgtaccagtaaaactaaagccaggaatggatggcccaaaagtt

aaacaatggccactgacaaaagagaaaatagaagcattaacagcaatttgtgaggaaatg

gaaaaggaaggaaaaattacaaaaattggacctgaaaacccatacaacactccaatattt

gccataaaaaagaaagacagtactaagtggagaaaattagtagatttcagggaactcaat

aaaagaactcaagatttttgggaagttcaattaggaataccacacccagcaggattaaaa

aagaaaaaatcagtgacagtgttggatgtgggggatgcctatttttcagttcctttatat

gaagacttcaggaaatatactgcattcaccatacctagtataaacaatgaaacaccaggg

attaggtatcagtataatgtacttccacagggatggaaaggatcaccagcaatatttcaa

agtagcatgacaaaaatcttagagccttttagaaaacaaaatccagacatagtcatctat

caatacatggatgatttgtatgtaggatctgacttagagatagggcagcatagaacaaaa

atagaggaactgagacaacatttgttgaggtggggatttaccacaccagacaagaaacat

cagaaagaacctccatttctttggatgggatatgaactccatcccgacaaatggacagtg

cagcctatacagctgccagtacaagatagctggactgtcaatgatatacaaaagttagtg

ggaaaattaaactgggcaagtcagatttatcctggaattaaagtaaggcagctttgtaaa

ctccttaggggggtcaaaacactaacagacatagtaccactaactgaagaagcag-----

------------------------------------------------

>CRF07BC.HZ160007

cctcaaatcactctttggcagcgaccccttgttaccataaagataggggggcaattaaag

gaagctctattggatacaggagcagatgatacagtattagaagacatgaatttgccaggg

aaatggaaaccaaaaatgatagggggaattggaggttttatcaaagtaagacagtatgaa

cagatacccatagaaatttgtggacataaagctataggtacagtattagtaggacctaca

cctgtcaacataattggaagaaatctgttaactcagcttggttgtactctaaattttcca

atcagtcctattgaaactgtgccagtaaaactaaagccaggaatggatggcccgaaggtt

aaacaatggccattgacaaaagagaaaatagaagcattaacagcaatttgtgatgaaatg

gaaaaggaaggaaaaatcacaaaaattgggcctgaaaatccatacaatactccaatattt

gccataaaaaagaaagacagtactaagtggagaaaattagtagatttcagggaactcaat

aaaagaactcaagattttcgggaagttcaattaggaataccacacccagcagggttaaaa

aagaaaaaatcagtgacagtgctggatgtgggggatgcatatttttcagttcctttatat

gaagacttcaggaaatatactgcattcaccatacctagtataaacaatgaaacaccaggg

attaggtatcagtacaatgtacttccacaggggtggaaaggatcaccagcaatatttcaa

agtagcatgacaaaaatcttagagccttttaggaaacaaaatccagacatagtcatctat

caatacatggatgatttgtatgtaggatctgacttagagatagggcagcatagaacaaaa

atagaggaactgagacawcatttgttagggtggggatttaccacaccagacaaaaaacat

cagaaagaacctccatttctttggatggggtatgaactccatcctgacaaatggacagta

cagcctatacagctgccagtacaagatagctggactgtcaatgatatacaaaagttagtg

ggaaaattaaactgggcaagtcagatttatcctggaattaaagtaaggcaactttgtaaa

ctccttagggggaccaaagcactaacagacatagtaccactaactgaagaagcaga----

------------------------------------------------

>CRF07BC.HZ160068

cctcaaatcactctttggcaacgaccccttgttacaataaagataggggggcaattaaag

gaagctctattagatacaggagcagatgatacagtattagaagacatgaatttgccaggg

aaatggaaaccaaaaatgatagggggaattggaggttttatcaaagtaagacagtatgaa

gagatacccatagaaatctgtggacataaagctataggtacagtgttaataggacctaca

cctgtcaacataattggaagaaatctgttgactcagcttggttgtactttaaattttcca

atcagtcctattgaaactgtaccagtaaaactaaagccaggaatggatgggccaaaggtt

aaacaatggccattgacaaaagagaaaatagaagcattaacagcaatttgtgatgaaatg

gaaaaggaaggaaaaattacaaaaattgggcctgaaaatccatacaacactccaatattt

gccataaaaaagaaagacagtactaagtggagaaaattagtagacttcagggaactcaat

aaaagaactcaagatttttgggaagttcaattaggaataccacacccagcaggattaaaa

aagaaaaaatcagtgacagtgctggatgtgggggatgcatatttttcagttcctttatat

gaagacttcaggaaatatactgcattcaccatacctagtataaacaatgaaacaccaggg

attaggtatcagtacaatgtacttccacagggatggaaaggatcaccagcaatatttcaa

agtagcatgacaaaaatcttagagccttttagaaaacaaaatccagacatagtcatctat

caatacatggatgatttgtatgtaggatctgacctagagatagggcagcatagaacaaaa

atagaggaactgagacaacatttgttgaggtggggatttaccacaccagacaagaaacat

cagaaagaacctccatttctttggatggggtatgaactccatcctgacaaatggacagta

cagcctatacagctgccagtacaagatagctggactgtcaatgatatacaaaagttagtg

ggaaaattaaactgggcaagtcagatttatcctggaattaaagtaaggcaactttgtaaa

ctccttaggggggccaaagcactaacagacatagtaccactaactgaagaagcagaatta

gaaa--------------------------------------------

>CRF07BC.HZ160130

cctcaaatcactctttggcaacgacccatcgtttcagtaaagataggggggcaattaaaa

gaagctctattagatacaggagcagatgatacagtattagaagatatagatttgccaggg

aaatggaaaccaaaaatgatagggggaattggaggttttatcaaagtaagacagtatgaa

cagatacccatagaaatctgtggacataaagctataggtacagtattagtaggacctaca

cctgtcaacataattggaagaaatctgttgactcagattggttgtactttaaatttccca

attagtcctattgaaactgtaccagtaaaattaaagccagggatggatgggccaaaggtt

aaacaatggccattgtcagaagaaaaaataaaagcattaacagaaatttgtaaagaaatg

gaagaggaaggaaaaatctcaaaaattgggcctgaaaatccatacaatactccaatattt

gctataaagaaaaaagacagcaccaaatggaggaaattagtagacttcagagagctcaat

aaaagaactcaggacttttgggaggttcaattaggaataccacacccatcagggttaaaa

agaaaaaagtcagtgacagtactggatgtgggggatgcatatttttcagttcccttacat

gaagacttcaggaaatatactgcattcaccatacccagtacaaacaatgaaacaccaggg

gttaggtatcagtacaatgtacttccacagggatggaaaggatcaccagcaatattccaa

agtagcatgacaaaaatcttagaaccttttagaaaacaaaatccagacatagaaatctat

caatacatggatgatttgtatgtaggatctgacttagaaatagggcaacatagaacaaaa

atagaggagctgagacaacacttgttgaggtggggatttaccacaccagacaagaaacat

cagaaagaacccccatttctttggatggggtatgaactccatcctgacaaatggacagta

cagcctatacagctrccagagaaagatagctggactgtcaatgatatacaaaagttagtg

ggaaaattaaactgggcaagtcagatttatcctggaattaaagtaaggcaactttgtaaa

ctcattagaggggtcaaagcactaacagacatagtaccactaactgaagaagcagaatta

gaattggcaga-------------------------------------

>CRF07BC.HZ160135

cctcaaatcactctttggcaacgacccattgtcacagtaaggataggggggcaattaaag

gaagctctattagatacaggagcagatgatacagtattagaagacatgaatttgccaggg

aaatggaaaccaaaaatgatagggggaattggaggttttatcaaagtaagacagtatgaa

cagatacccatagaaatctgtggacatcaagctataggtacagtattggtaggacctaca

cctgtcaacataattggaagaaatctgttgactcagcttggttgtactctaaattttcca

atcagttctattgaaactgtaccagtaaaactaaagccaggaatggatggcccaaaggtt

aaacaatggccattgacaaaagagaaaatagaagcattaacagcaatttgtgatgaaatg

gaaaaggaaggaaaaattacaaaaattgggcctgaaaatccatacaacactccaatattt

gccataaaaaagaaagacagtactaagtggagaaaattagtagatttcagggaactcaat

aagagaactcaagatttttgggaagttcaattaggaataccacacccagcaggattaaaa

aagaaaaaatcagtgacagtgctggatgtgggggatgcatatttctcagttcctttatat

gaagacttcaggaaatatactgcattcaccatacctagtataaacaatgaaacaccaggg

attaggtatcagtacaatgtacttccacagggatggaaaggatcaccagcaatatttcaa

agtagcatgacaagaatcttagagccttttagaaaacaaaatccagacatagtcatctat

caatacatggatgatttgtatgtaggatctgacttagagatagggcagcatagaacaaaa

atagaggaactgagacaacatttgttgaggtggggatttaccacaccagacaagaaacat

cagaaagaacccccatttctttggatggggtatgaactccatcctgacaaatggacagta

cagcctatacagctgccagtacaagacagctggactgtcaatgatatacaaaagttagtg

ggaaaattaaactgggcaagtcagatttatcctggaattaaagtaaggcaactttgtaaa

ctccttagggggaccaaagcactaacagaaatagtaccactaactgaagaagcagaatta

gaaa--------------------------------------------

>CRF07BC.HZ160140

cctcaaatcactctttggcaacgaccccttgtcacaataaaggtaggggggcaattaaag

gaagctctattagatacaggagcagatgatacagtactagaagacataaatttgccaggg

aaatggaagccaagaatgatagggggaattggaggttttatcaaagtaagacagtatgaa

caggtacccatagaaatctgtggacatcaagctacaggcacagtgttagtaggaccaacc

cctgtcaacataattggaaggaatctgttgactcagcttggttgtactttaaattttcca

atcagtcctattgaaactgtgccagtaaaactaaagccagggatggatggcccaaaggtt

aaacaatggccattgacaaaagagaaaatagaagcattaacagcaatttgtgatgaaatg

gaaaaggaaggaaaaattacaaaaattgggcctgaaaatccatacaacactccaatattt

gccataaaaaagaaagacagtactaagtggagaaaattagtagatttcagggaactcaat

aaaagaactcaagatttttgggaagtacaattaggaataccacacccagcaggattaaaa

aagaaaaaatcagtgacagtgctggatgtgggagatgcatatttttcagttcctttacat

gaagactttaggaaatatactgcattcaccatacctagtataaacaatgaaacaccaggg

attaggtatcagtacaatgtacttccacagggatggaaaggatcaccagcaatatttcaa

agtagcatgacaagaatcttagaaccttttagaaaacaaaatccagacatagtcatctat

caatacatggatgatttgtatgtaggatctgacttagagatagggcagcatagaacaaaa

atagaggaactgagacaacatttgttgaggtggggatttaccacaccagacaagaaacat

cagaaagaacctccatttctttggatggggtatgaactccatcctgataaatggacagta

cagcctatacagctgccagtacaagatagctggactgtcaatgatatacaaaagttagtg

ggaaaattaaactgggcaagtcagatttatcctggaattaaagtaaggcaactttgtaaa

ctccttagggggaccaaagcactaacagacatagtaccactaactgaagaagcagaatta

gaa---------------------------------------------

>CRF07BC.HZ160155

cctcaaatcactctttggcaacgaccccttgtcacagtaaaaatagggggacagctgaaa

gaggctctattagatacaggagcagatgatacagtattagaagatataaatttaccagga

aaatggaaaccaaaaatgatagggggaattggaggttttatcaaagtaaggcaatatgat

cagatacttatagaaatttgtggaaaaaaggctataggtacagtattagtaggacctaca

cctgtcaacataattggacgaaatatgttgactcagattggttgtactttaaattttcca

attagtcctattgaaactgtaccagtaaaattaaagccaggaatggatggcccgaaggtt

aaacaatggccattaacaaaagagaaaatagaagcattaacagcaatttgtgatgaaatg

gaaaaggaaggaaaaattacaaaaattgggcctgaaaatccctacaacactccaatattt

gccataaaaaagaaagacagtactaagtggagaaaattagtagatttcagggaactcaat

aaaagaactcaagatttttgggaagttcaattaggaataccacatccagcaggattaaaa

aagaaaagatcagtgacagtgctggatgtgggggatgcatatttttcagttcctttatat

gaagacttcaggaaatatactgcattcaccatacctagtataaataatgaaacaccaggg

attaggtatcagtacaatgtacttccacagggatggaaaggatcaccagcaatatttcaa

agtagcatgacaagaatcttagagccttttagaaaacaaaatccagacatagtcatctat

caatacatggatgatttgtatgtaggatctgacttagagatagggcaacatagaacaaaa

atagaggaactgagacaacatttgttgaggtggggatttaccacaccagacaagaaacat

cagaaagagcctccatttctttggatggggtatgaactccatcctgacaaatggacagta

cagcctatacagctgccagtacaagatagctggactgtcaatgatatacaaaagttagtg

ggaaaattaaactgggcaagtcagatttatcctggaattaaagtaaggcaactttgtaaa

ctccttaggggggccaaagcactaacagacatagtaccactaactgaagaagcagaatta

------------------------------------------------

>CRF07BC.HZ160182

cctcaaatcactctttggcaacgacccctcgtcacaataaagataggggggcaattaaaa

gaagctctattagacacaggagcagatgatacagtattagaagacatgaatttaccaggg

aaatggagaccaaaaatgatagggggaattggaggttttatcaaagtaagacagtatgac

cagatacacatagaaatctgtggacacaaagttatgggtacagtattagtgggacctaca

cctgtcaacataattggaagaaatctgttgactcagcttggttgcaccttaaattttcca

atcagtcccattgaaactgtaccagtaaaattaaagccaggaatggatggcccaaaggtt

aaacaatggccgttgacagaagagaaaatagaagcattaaaagcaatttgtgatgaaatg

gagaaggaaggaaaaattacaaaaattgggcctgaaaatccatataacactccaatattt

gccataaaaaagaaggacagtactaagtggagaaaattagtagacttcagggaactcaat

aaaagaactcaagatttttgggaagttcaattaggaataccacacccagcagggttaaaa

aagaaaaaatcagtgacagtactagatgtgggggatgcatatttttcagttcctttatat

gaagacttcaggaaatatactgcattcaccatacctagtataaacaatgaaacaccaggg

attaggtatcagtacaatgtacttccacagggatggaaaggatcaccagcaatattccaa

agtagcatgacaagaatcttagagccttttagaaaacaaaatccagacatagttatttat

caatacatggatgatttgtatgtaggatctgatttagagataggacagcatagaacaaaa

atagaggaactgagagaacatttgttgaggtggggatttaccacaccagacaagaaacat

cagaaagaacctccatttctttggatggggtatgaactccatcctgacaagtggacagta

cagcctatacagctaccagaaaaagatagctggactgtcaatgatatacagaagttagtg

ggaaaattaaactgggccagtcagatttatcctggaattaaagtaaggcagctttgtaaa

ctccttaggggggccaaagcactaacagacatagtaccactaactgaagaagcagaa---

------------------------------------------------

>CRF07BC.HZ160185

cctcaaatcactctttggcaacgaccccttgttaccataaagataggggggcaattaaag

gaagctctattagatacaggagcagatgatacagtattagaagacatgaatttgccaggg

aaatggaaaccaaaaatgatagggggaattggaggttttatcaaagtaagacagtatgaa

cagatacccatagaaatctgtggacataaagctataggtacagtattagtaggacctaca

cctatcaacataattggaagaaatctgttgacccagcttggttgtactttaaattttcca

atcagtcctattgaaactgtaccagtaaaactaaagccaggaatggatggcccaaaagtt

aaacaatggccattgacaaaagaaaaaatagaagcattaacagcaatttgtactgaaatg

gaaaaggaaggaaaaattacaaaaattgggcctgaaaatccatacaacactccaatcttt

gccataaaaaagaaagacagtactaagtggagaaaattagtagatttcagggaactcaat

aaaagaactcaagatttttgggaagttcaattaggaataccacacccagcaggattaaaa

aagaaaaaatcagtgacagtgctggatgtgggggatgcatatttttcagttcctttatat

gaaaacttcaggaaatatactgcattcaccatacctagtataaacaatgaaacaccaggg

attaggtatcagtacaatgtacttccacagggatggaaaggatcaccagcaatatttcaa

agtagcatgacaaaaatcttagagccttttagaaaacaaaatccagacatagtcatctat

caatacatggatgatttgtatgtaggatctgacttagagatagggcagcatrgaacaaaa

atagaggaactgagacaacacttgttgaggtggggatttaccacaccagacaaaaaacat

caaaaagaacctccatttctgtggatggggtatgaactccatcctgacaaatggacagta

cagcctatacagctaccagtgcaagatagctggactgtcaatgatatacaaaagttagtg

ggaaaattaaactgggcaagtcagatttatcctggaattaaagtaaggcagctttgtaaa

ctccttagggggaccaaagcactaacagaagtagtaacacta------------------

------------------------------------------------

>CRF07BC.HZ160192

cctcaaatcactctttggcagcgacccttagtcacagtaaaaataggaggacagctaaaa

gaagctctattagatacaggagcagatgatacagtattagaagatataaatttgccagga

aaatggaaaccaaaaatgatagggggaattggaggttttatcaaagtgagacaatatgat

cagatacttatagaaatttgtggaaaaaaggctataggtacagtattagtaggacctaca

cctgtcaacataattggacgaaatatgttgactcagattggttgtactttaaatttccca

atcagttctattgaaactgtaccagtaaaactaaagccaggaatggatggcccaaaggtt

aaacaatggccattgacaaaagagaaaatagaagcattaacagcaatttgtgatgaaatg

gaaaaggaaggaaaaattacaaagattgggcctgaaaatccatacaacactccaatattt

gccataaaaaagaaggacagtactaagtggagaaaattagtagatttcagggagctcaat

aaaagaactcaagatttttgggaagttcaattaggaataccacacccagcaggattaaaa

aagaaaaaatcagtgacagtgctggatgtgggagatgcatatttttcagtccctttatat

gaagacttcaggaaatatactgcattcaccatacctagtataaacaatgaaacaccaggg

attaggtatcagtacaatgtacttccacagggatggaaaggatcaccagcaatatttcaa

agtagcatgacgagaatcttagatccttttagaaaacaaaatccagacatggtcatctat

caatacatggatgatttgtatgtaggatctgacttagagatagggcagcatagaataaaa

atagaggaactgagacaacatttgttgaggtggggatttaccacaccagacaagaaacat

cagaaagaacctccatttctttggatggggtatgaactccatcctgacaaatggacagta

cagcctatacagttgccagtacaagatagctggactgtcaatgatatacaaaagttagtg

ggaaaattaaactgggcaagtcagatttatgctggaattaaagtaaggcaactctgtaaa

ctccttaggggggccaaggcactaacagacatagtaccactaactgaagaagcagaatta

gaa---------------------------------------------

>CRF07BC.HZ160235

cctcagatcactctttggcagcgacccctcgtcacaataaagataggggggcaattaaaa

gaagctctattagatacaggagcagatgatacagtattagaagaaatgaatttgccaggg

aaatggaaaccaaaaatgatagggggaattggaggttttatcaaagtaaggcagtatgaa

caggtacccatagaagtatgtggacacaaagttataggtacagtattagtagggcctaca

cctgtcaacataattggaagaaatctgttgactcagcttggttgcactttaaattttcca

atcagtcccattgaaactataccagtaaaattaaagccaggaatggatggcccaaaggtt

aaacaatggccattgacagaagaraaaataaaagcattaacagaaatttgtaaggaaatg

gagaaggaaggaaaaattacaaaaattgggcctgaaaatccatataacactccaatattt

gccataaaaaagaaggacagtacaaagtggagaaaactagtagacttcagagaactcaat

aaaagaactcaagatttttgggaagttcaattaggaataccacacccagcaggattaaaa

aagaaaaaatcagtgacagtactggatgtgggggatgcatatttttcagttcctttacat

gaggacttcaggaaatatactgcattcaccatacctagtataaacaatgaaacaccaggg

attaggtatcagtataatgtacttccacaaggatggaaaggatcaccagcaatattccaa

agtagcatgacaaagatcctagagccttttagaaaacaaaatccagacatagttatctat

caatacatggatgatttatatgtgggatctgatttagagatagggcagcatagagcaaaa

atagaggaattaagagaacatttgttgaggtggggattcaccacaccagacaagaaacat

cagaaagaaccgccatttctttggatggggtatgaactccatcctgacaaatggacagta

cagcctataaagctgccagaaaaagatagctggactgtcaatgatatacaaaagttagtg

ggaaaattaaactgggcaagtcagatttatcctggaattaaagtaaggcaactttgtaaa

cttattaggggagtcaaagcactaacagacatagtaccactaactgaagaagcagaatta

------------------------------------------------

>CRF07BC.HZ160243

cctcaaatcactctttggcaacgaccccttgttaccataaagataggagggcaattaaag

gaagctctattagatacaggagcagatgatacagtattagaagagataaatttgccaggg

aaatggaaaccaaaaatgatagggggaattggaggttttatcaaagtaagacagtatgaa

caggtacccatagaaatatgtggacataaagctataggtacagtattagtagggcctaca

cctgtcaacataattggaagaaatctgttgactcagcttggttgtactttaaattttcca

atcagtcctattgaaactgtaccagtaaaactaaagccaggaatggatggcccaaaagtt

aaacaatggccattgacaaaagagaaaatagaagcattaacagcaatttgtgaygaaatg

gaaaaggaaggaaaaattacaaaaattgggcctgaaaatccatacaatactccaatattt

gctataaaaaagaaagacagtactaagtggagaaaattagtagatttcagggaactcaat

aaaagaactcaagatttttgggaagttcaattaggaataccacacccagcaggattraaa

aagaaaaaatcagtracagtgctggatgtgggggatgcatatttttcagttcctttagat

gaarayttcaggaaatatactgcattcaccatacctagtataaacaatgaaacaccaggg

attaggtatcagtacaatgtacttccacagggatggaaaggatcaccagcaatatttcaa

agtagcatgacaaaaatcttagagccttttagaaaacaaaatccagacatagtcatctat

caatacatggatgatttgtatgtaggatctgacttagagatagggcagcacagaacaaaa

atagaggaactgagacaacatttgttgaggtggggatttaccacaccagacaaaaaacat

cagaaagagccgccatttctttggatggggtatgagctccatcctgacaaatggacagta

caacctatacagctgccagtacaagatagctggactgtcaatgatatacaaaagttagtg

ggaaaattaaactgggcaagtcaaatttatcctggaattaaagtaaggcaactttgtaaa

ctccttagggggaccaaagcactaacagacatagtaccactaactgaagaagcagaatta

------------------------------------------------

>CRF07BC.HZ160282

cctcaaatcactctttggcaacgaccccttgtcacaataaagataggggggcaattaaag

gaagctctattagatacaggagcagatgatacagtattagaagacatgaatttgccaggg

aaatggaaaccaaaaatgatagggggaattggaggttttatcaaagtaagacagtatgaa

gagatacccatagaaatctgtggacataaagctataggtacagtattagtgggacctaca

cctgtcaacataattggaagaaatctgttgactcagcttggttgtactttaaattttcca

atcagtcctattgaaactgtaccagtaagactaaagccaggaatggatggcccaaaggtt

aaacaatggccattaacaaaagaraaaatagaagcattaacagcaatttgtgatgaaatg

gaaaaggaaggaaaaattacaaaaattgggcctgaaaatccctacaacactccaatattt

gccataaaaaagaaagacagtactaagtggagaaaattagtagatttcagggaactcaat

aaaagaactcaagatttttgggaagttcaattaggaataccacacccagcagggttaaaa

aagaaaaaatcagtgacagtgctggatgtgggggatgcatatttttcagttcctttatat

gaagatttcaggaaatatactgcattcaccatacctagtataaacaatgaaacaccaggg

attaggtatcagtacaatgtacttccacagggatggaaaggatcaccagccatattccaa

agtagcatgacaaaaatcttagagccttttagaaaacaaaatccagacatagtcatctac

caatacatggatgatttatatgtaggatctgatttagagatagggcagcatagagcaaaa

atagaggaactgagacaacatttgttgaggtggggatttaccacaccagacaagaaacat

cagaaagaacccccatttctttggatggggtatgaactccatcctgacaaatggacggta

cagcctatacagctgccagtacaggatagctggactgtcaatgatatacaaaagttagtg

ggaaaattaaactgggcaagtcagatctatcctggaattaaagtaaggcaactttgtaaa

ctccttaggggggccaaggc----------------------------------------

------------------------------------------------

>CRF07BC.HZ160289

cctcaaatcactctttggcaacgaccccttgttaccataaagataggggggcaattaaag

gaagctctattagatacaggagcagatgatacagtattagaagacatgaatttgccaggg

aaatggaaaccaaaaatgatagggggaattggaggttttatcaaagtaagacagtatgac

cagataaccatagaaatctgtggacataaagttataggtacagtcttggtaggacctaca

cctatcaatataattggaagaaatctgttgactcagcttggttgtactttaaattttcca

atcagtcctattgaaactgtaccagtaaaattaaagccaggaatggatggcccaaaggtt

aaacaatggccattgacaaaagagaaaatagaagcattaacagcaatttgtgaagaaatg

gaaaaggaaggaaaaattacaaaaattgggcctgaaaatccatacaacactccaatattt

gccataaaaaagaaagatagtactaagtggagaaaattagtagatttcagggaactcaat

aaaagaactcaagatttttgggaagtacaattaggaataccacacccagcaggattaaaa

aagaaaaaatcagtgaccgtgctggatgtgggggatgcatatttttcagttcctttatat

gaagacttcaggaaatatactgcattcaccatacctagtataaacaatgaaacaccaggg

attaggtatcagtacaatgtacttccacagggatggaaaggatcaccagcaatattccaa

agtagcatgacaaaaatcttagagccttttagaaagcaaaatccagacatggtcatctat

caatacatggatgatttgtatgtaggatctgacttagagatagggcagcatagaacaaaa

atagaggaactgagacaacatttgttgaggtggggatttaccacaccagacaagaaacat

cagaaagaacctccatttctttggatggggtatgaactccatcctgacaaatggacagta

cagcctatacagctgccagtacaagatagctggactgtcaatgatatacaaaagttagtg

ggaaaattaaactgggcaagtcagatttatcctggaattaaagtaaggcagctttgtaaa

ctccttagaggggcc---------------------------------------------

------------------------------------------------

>CRF07BC.HZ160319

cctcaaatcactctttggcaacgaccccttgtyacaataaagataggggggcaattaaag

gaagctctattagatacaggagcagatgatacagtattagaagacatgaatttgccaggg

aaatggaaaccaaaaatgatagggggaattggaggttttatcaaagtaagacagtatgaa

gagatacccatagaaatctgtggacataaagctataggtacagtgttaataggacctaca

cctgtcaacataattggaagaaatctgttgactcagcttggttgtactttaaattttcca

atcagtcctcttgaaactgtaccagtaaaactaaagccaggaatggatgggccaaaggtt

aaacaatggccattgacaaaagagaaaatagaagcattaacagcaatttgtgatgaaatg

gaaaaggaaggaaaaattacaaaaattgggcctgaaaatccatacaacactccaatattt

gccataaaaaagaaagacagtactaagtggagaaaattagtagacttcagggaactcaat

aaaagaactcaagatttttgggaagttcaattaggaataccacacccagcaggattaaaa

aagaaaaaatcagtgacagtgctggatgtgggggatgcatatttttcagttcctttatat

gaagacttcaggaaatatactgcattcaccatacctagtataaacaatgaaacaccaggg

attaggtatcagtacaatgtacttccacagggatggaaaggatcaccagcaatatttcaa

agtagcatgacaaaaatcttagagccttttagaaaacaaaatccagacatagtcatctat

caatacatggatgatttgtatgtaggatctgacctagagatagggcagcatagaacaaaa

atagaggaactgagacaacatttgttgaggtggggatttaccacaccagacaagaaacat

cagaaagaacctccatttctttggatggggtatgaactccatcctgacaaatggacagta

cagcctatacagctgccagtacaagatagctggactgtcaatgatatacaaaagttagtg

ggaaaattaaactgggcaagtcagatttatcctggaattaaagtaaggcaactttgtaaa

ctccttaggggggccaaagcactaacagacatagtaccactaactgaagaagcagaag--

------------------------------------------------

>CRF07BC.HZ160328

cctcaaatcactctttggcaacgaccccttgttaccataaagataggggggcaattaaag

gaagctctattagatacaggagcagatgatacagtattagaagacatgaatttgccaggg

aagtggaaaccaaaaatgatagggggaattggaggttttatcaaagtaagacagtatgaa

gaggtccccatagaaatctgtggacataaagctataggtacagtattagtaggacctaca

cctgtcaacataattggaagaaatctgttgactcagcttggttgtaccttaaattttcca

atcagtcctattgacactgtaccagtaaaactaaagccaggaatggatggcccaaaggtt

aaacaatggccattgacaaaagagaaaatagaagcattaacagcaatctgtgatgaaatg

gaaaaggaaggaaaaattacaaaaattgggcctgaaaatccatacaacactccaatattt

gccataaaaaagaaagacagtactaagtggagaaaattagtagatttcagggaactcaat

aaaagaactcaagatttttgggaagttcaattaggaataccacacccagcaggattaaaa

aagaaaaaatcagtgacagtgctggatgtgggggatgcatatttttcagttcctttacat

gaagacttcaggaaatatactgcattcaccatacctagtataaacaatgaaacaccaggg

attaggtatcagtacaatgtacttccacaggggtggaaaggatcaccagcaatatttcaa

agtagcatgacaaaaatcttagagccttttagaaaacaaaatccagacatagtcatctat

caatacatggatgatttgtatgtaggatctgacttagagatagggcagcatagaataaaa

atagaggaactgagacaacatttgttgaggtggggatttaccacaccagacaagaaacat

cagaaagaacctccatttctttggatggggtatgaactccatcctgacaaatggacagta

cagcctatacaactgccagtacaagatagctggactgtcaatgatatacaaaagttagtg

ggaaaattaaattgggcaagtcagatttatcctggaattaaagtaaggcaactttgtaaa

ctccttaggggggccaaagcactaacagacatagtaccactaactgaagaagcagaa---

------------------------------------------------

>CRF07BC.HZ161121

cctcaaatcactctttggcaacgaccccttgttaccataaagataggggggcaattaaag

gaagctctattagatacaggagcagatgatacagtattagaagatatggatttgccaggg

aaatggaaaccaaaaatgataggaggaattggaggttttatcaaagtacgacagtatgaa

cagatacccatagaaatctgtggacataaagctataggtacagtattagtagggcctacc

cctgtcaacataattggaagaaatctgttgactcagcttggttgtactttaaattttcca

atcagtcctattgaaactgtaccagtaaaactaaagccaggaatggatggcccaaaggtt

aaacaatggccattgacaaaagaaaaaatagaagcattaacagcaatttgtgatgaaatg

gaaaaggaaggaaaaattacaaaaattgggcctgaaaatccatacaacactccaatattt

gccataaaaaagaaagacagtactaagtggagaaaattagtagatttcagggaacttaat

aaaagaactcaagatttttgggaagttcaattaggaataccacacccagcaggattagaa

aagaacaaatcagtgacagtgctggatgtgggggatgcctatttttcagtgcctttatat

gaagatttcaggaaatatactgcatttaccatacctagtayaaacaatgaaacaccaggg

attaggtatcagtacaatgtacttccacagggatggaaaggatcaccagcaatatttcaa

agtagcatgacaaaaatcttagagccttttagaaaacaaaatccagacatagtcatctat

caataygtggatgatttgtatgtaggatctgacttagagatagggcarcatagagcaaaa

atagaggaactgagacaacatttgttgaggtggggatttaccacaccagacaagaaacat

cagaaagaacctccatttctttggatggggtatgaactccatcctgacaaatggacagta

cagcctatacagctgccagtgcaagatagctggactgtcaatgatatacaaaagttagtg

ggaaaattaaactgggcaagtcagatttatcctggaattaaagtaagrcarctttgtaaa

ctccttagggggaccaaagcactaacagacatagtaccactaactgaagaagcagaat--

------------------------------------------------

>CRF07BC.HZ161968

cctcagatcactctttggcaacgaccccttgttaccataaagataggggggcaattaaag

gaagctctattagatacaggagcagatgatacagtattagargacatgaatttgccaggg

aaatggaaaccaaaaatgatagggggaattggaggttttatcaaagtaagacagtatgaa

cagrtacccatagaaatttgtggacataaagctataggtacagtattaataggacctaca

cctgtcaacataattggaagaaatctgttgactcagcttggttgtactttaaattttcca

atcagtcctattgawactataccagtaaaactaaagccaggaatggatggcccaaaggtt

aaacaatggccattgacaaaagagaaaatagaagcattaacagcaatttgtgatgaaatg

gaaaaggaaggaaaaattacaaaaattgggcctgaaaatccatacaacactccaatattt

gcyataaaaaagaaagacagcactaagtggagaaaattagtagatttcagggaactcaat

aaaaggactcaagatttttgggaagttcaattaggaataccacacccagcaggattaaaa

aagaaaaaatcagtgacagtrctggatgtgggggatgcatatttttcagttcctttabat

gaagatttcaggaaatatactgcattcaccatacctagtatmaacaatgaaacaccaggg

attaggtatcagtacaatgtacttccacagggatggaaaggatcaccagcaatatttcaa

agtagcatgacaaaaatcttagagccttttagaaaacaaaatccagacatagtcatctat

caatacatggatgatttgtatgtaggatctgacttagagatagggcagcacagarcaaaa

atagaggaactgagacagcatttgttgaagtgggggtttaccacaccagacaagaaacat

cagaaagaacctccatttctttggatggggtatgaactccatcctgacaaatggacagta

cagcctatacagctgccagtacaagatagctggactgtcaatgatatacaaaagttagtg

ggaaaattaaactgggcaagtcagatttatcctggaattaaagtaaggcaactttgtaaa

ctccttagggggaccaaagcactaacagacatagtaccactaactgaagaagcaga----

------------------------------------------------

>CRF07BC.HZ162724

cctcaaatcactctttggcaacgacccmttgttaccataaagataggggggcaattaaag

gaagcyctattagatacaggrgcagatgatacagtattagaagacatgaatttgccaggg
[truncated: 557,836 more chars]
